# ﻿New polyketides from the liquid culture of *Diaporthebreyniae* sp. nov. (Diaporthales, Diaporthaceae)

**DOI:** 10.3897/mycokeys.90.82871

**Published:** 2022-06-14

**Authors:** Blondelle Matio Kemkuignou, Lena Schweizer, Christopher Lambert, Elodie Gisèle M. Anoumedem, Simeon F. Kouam, Marc Stadler, Yasmina Marin-Felix

**Affiliations:** 1 Department of Microbial Drugs, Helmholtz Centre for Infection Research (HZI) and German Centre for Infection Research (DZIF), Partner Site Hannover/Braunschweig, Inhoffenstrasse 7, 38124 Braunschweig, Germany Department of Microbial Drugs, Helmholtz Centre for Infection Research (HZI) and German Centre for Infection Research (DZIF) Braunschweig Germany; 2 Institute of Microbiology, Technische Universität Braunschweig, Spielmannstraße 7, 38106 Braunschweig, Germany Technische Universität Braunschweig Braunschweig Germany; 3 Department of Chemistry, Higher Teacher Training College, University of Yaoundé I, Yaoundé P.O. Box 47, Cameroon University of Yaoundé I Yaounde Cameroon

**Keywords:** Antimicrobial, cytotoxicity, *Diaporthe*, endophytic fungi, one new species, secondary metabolites

## Abstract

During the course of a study on the biodiversity of endophytes from Cameroon, a fungal strain was isolated. A multigene phylogenetic inference using five DNA loci revealed that this strain represents an undescribed species of *Diaporthe*, which is introduced here as *D.breyniae*. Investigation into the chemistry of this fungus led to the isolation of two previously undescribed secondary metabolites for which the trivial names fusaristatins G (**7**) and H (**8**) are proposed, together with eleven known compounds. The structures of all of the metabolites were established by using one-dimensional (1D) and two-dimensional (2D) Nuclear Magnetic Resonance (NMR) spectroscopic data in combination with High-Resolution ElectroSpray Ionization Mass Spectrometry (HR-ESIMS) data. The absolute configuration of phomopchalasin N (**4**), which was reported for the first time concurrently to the present publication, was determined by analysis of its Rotating frame Overhauser Effect SpectroscopY (ROESY) spectrum and by comparison of its Electronic Circular Dichroism (ECD) spectrum with that of related compounds. A selection of the isolated secondary metabolites were tested for antimicrobial and cytotoxic activities, and compounds **4** and **7** showed weak antifungal and antibacterial activity. On the other hand, compound **4** showed moderate cytotoxic activity against all tested cancer cell lines with IC_50_ values in the range of 5.8–45.9 µM. The latter was found to be less toxic than the other isolated cytochalasins (**1**–**3**) and gave hints in regards to the structure-activity relationship (SAR) of the studied cytochalasins. Fusaristatin H (**8**) also exhibited weak cytotoxicity against KB3.1 cell lines with an IC_50_ value of 30.3 µM.

﻿**Graphical abstract**

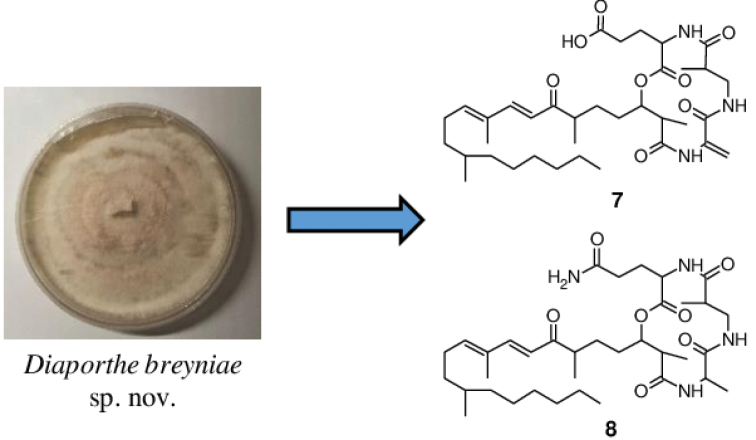

## ﻿Introduction

The genus *Diaporthe* (including their asexual states, which were previously referred to as *Phomopsis* spp.) comprises several hundred species mostly attributed to plant pathogens, non-pathogenic endophytes, or saprobes in terrestrial host plants (Chepkirui and Stadler 2017; Xu et al. 2021). The term “endophytic fungi” herein refers to a group of microorganisms that inhabit the internal parts of a plant, but typically cause no apparent symptoms of disease in the host plant (Stone et al. 2000). Fungal endophytes belonging to the genus *Diaporthe* have been widely investigated by natural product chemists and have proven to be a rich source of novel organic compounds with interesting biological activities and a high level of chemical diversity (Chepkirui and Stadler 2017). They have been shown to predominantly produce polyketides, but PKS/NRPS-derived hybrids like cytochalasins have also been frequently reported from *Diaporthe* (Jouda et al. 2016; Chepkirui and Stadler 2017). Initially, cytochalasins have been discovered for their potent cytotoxic effects, which are due to their interference with the actin cytoskeleton (Yahara et al. 1982) and have been targeted primarily as anticancer agents. However, not all cytochalasins are equally active on actin (Kretz et al. 2019), and they were even found to significantly inhibit biofilm formation of an important human pathogenic bacterium (Yuyama et al. 2018). The current paper supports the activities of an interdisciplinary consortium that aims at exploring the chemical space of the cytochalasins, in order to establish structure-activity relationships (SAR) and systematically explore their utility for application in various medical applications. Owing to the structural complexity of cytochalasins, their total synthesis remains tedious and requires several reaction steps with relatively low final yields (Zaghouani et al. 2016; Long et al. 2018). Moreover, most of the compounds that were reported previously have not been studied thoroughly for their biological effects; hence, it is worth obtaining them from the fungal producer organisms by *de novo* isolation and characterization.

We have recently isolated and studied a new endophytic species of *Diaporthe* from the twigs of *Breyniaoblongifolia*. We noted prominent antimicrobial effects in the extracts derived from this strain and decided to study its secondary metabolites. The current paper includes the description of the new species *D.breyniae* sp. nov., and reports details on the isolation and structure elucidation of its secondary metabolites, as well as an account of their biological properties.

## ﻿Materials and methods

### ﻿Fungal isolation

The fungus was isolated from fresh twigs of an apparently healthy plant belonging to *Breyniaoblongifolia* in Kala Mountain (Yaoundé, Cameroon). Fresh twigs (5 × 5 cm length) of *Breyniaoblongifolia* were thoroughly washed with running tap water, then disinfected in 75% ethanol for 1 min, in 3% sodium hypochlorite (NaClO) for 10 min, and finally in 75% ethanol for 30 s. These twigs were then rinsed three times in sterile distilled water and dried on sterile tissue paper under a laminar flow hood. Small segments of the twigs were transferred to Petri dishes containing potato dextrose agar (PDA, HiMedia, Mumbai, India) supplemented with 100 mg/mL penicillin and 100 µg/mL streptomycin sulphate and incubated at 28 °C. After 10 days, fungal colonies were examined and hyphal tips were transferred to PDA using a sterile needle and incubated at 28 °C.

Herbarium type material and the ex-type strain of the new species are maintained at the collection of the Westerdijk Fungal Biodiversity Institute (CBS), Utrecht, the Netherlands.

### ﻿Phenotypic study

For cultural characterization, the isolate was grown for 15 days on malt extract agar (MEA; HiMedia, Mumbai, India), oatmeal agar (OA; Sigma-Aldrich, St. Louis, Missouri, USA), and PDA at 21 °C in darkness (Guarnaccia et al. 2018). Color notations in parentheses are taken from the color chart of The Royal Horticultural Society London (1966). The fungus was grown in 2% tap water agar supplemented with sterile pine needles (PNA; Smith et al. 1996) to induce sporulation.

### ﻿Molecular study

DNA of the fungus was extracted and purified directly from colony growing in yeast malt agar (YM agar; malt extract 10 g/L, yeast extract 4 g/L, D-glucose 4 g/L, agar 20 g/L, pH 6.3 before autoclaving), following the Fungal gDNA Miniprep Kit EZ-10 Spin Column protocol (NBS Biologicals, Cambridgeshire, UK). The amplification of the ITS, *cal*, *his3*, *tef1* and *tub2* loci were performed according to White et al. (1990) (ITS), Carbone and Kohn (1999) (*cal* and *tef1*), Glass and Donaldson (1995) (*his3* and *tub2*) and Crous et al. (2004) (*his3*). PCR products were purified and sequenced using Sanger Cycle Sequencing method at Microsynth Seqlab GmbH (Göttingen, Germany), and the consensus sequences obtained employing the de-novo assembly feature of the Geneious 7.1.9 (http://www.geneious.com, Kearse et al. 2012) program package using a forward and reverse read.

In order to restrict the phylogenetic inference to the relevant species to compare with, a first phylogenetic analysis was carried out based on the combination of the five loci sequences (ITS, *cal*, *his3*, *tef1*, *tub2*) of our isolate and a selection of sequence data derived from type material or reference strains from all *Diaporthe* spp. available in NCBI. Each locus was aligned separately using MAFFT v. 7.017 (algorithm G-INS-I, gap open penalty set to 1.53, offset value 0.123 with options set for automatically determining sequence direction automatically and more accurately) as available as a Geneious 7.1.9 plugin (Katoh and Standley 2013) and manually adjusted in MEGA v. 10.2.4 (Kumar et al. 2018). Alignment errors were minimized by using gblocks (Talavera and Castresana 2007); with options set for allowed block positions ‘with half’, minimum length of a block set to 5 and a maximum of 10 contiguous nonconserved positions) and concatenated by employing the phylosuite v 1.2.2 program package (Zhang et al. 2020). Maximum-Likelihood tree inference followed using IQTree V2.1.3 (Minh et al. 2020) preceded by calculation and automatic selection of the appropriate nucleotide exchange model using ModelFinder (Chernomor et al. 2016; Kalyaanamoorthy et al. 2017) based on Bayesian inference criterion. Bootstrap support was calculated by parallelizing 10 independent maximum-likelihood (ML) tree searches with 100 bootstrap replicates each to minimize computational burden. The total 1000 bootstrap replicates were consequently mapped onto the ML tree with the best (highest) ML score. After selection of the core group related to the sequences derived from *D.breyniae* sp. nov., a second phylogenetic analysis was performed including all five sequenced loci, using *D.amygdali*CBS 126679^T^ and *D.eres*CBS 138594^T^ as outgroups. Sequence alignment and curation steps were identical, with exemption of a manual curation instead of employing automatic filtering for misaligned alignment sections using gblocks. ML trees using the supermatrix and single loci, respectively, were inferred using IQTree 2.1.3 with ModelFinder to determine optimal substitution models for each loci and partition, using 1000 bootstrap replicates to assign statistical support. The clade in which the sequences of the novel strain clustered, was checked visually for congruence among the single locus trees. Concurrently, a second tree was inferred following a Bayesian approach using MrBayes 3.2.7a (Ronquist et al. 2012) with nucleotide substitution models previously determined using PartitionFinder2 (Lanfear et al. 2016, options set for unlinked partitions, BIC, restricting models for Bayesian inference) and concatenated in Phylosuite V.1.2.2. Bayesian inference was done in Mr. Bayes v. 3.2.7 (Ronquist et al. 2012), using Markov Chain Monte Carlo (MCMC) with four incrementally heated chains (temperature parameter set to 0.15), starting from a random tree topology. Generations were set to 100.000.000 with convergence controlled by average standard deviation of split frequencies arriving below 0.01. Trees were sampled every 1000 generations with the first 25% of saved trees treated as “burn-in” phase. Posterior probabilities were mapped using the remaining trees. Bootstrap support (bs) ≥ 70 and posterior probability values (pp) ≥ 0.95 were considered significant (Alfaro et al. 2003). The sequences generated in this study are deposited in GenBank (Table 1) and the alignments used in the phylogenetic analysis are included in Supplementary material. Sequences retrieved from GenBank are indicated in Table 1 and Suppl. material 1: S4.

### ﻿Chromatography and spectral methods

Electrospray ionization mass (ESIMS) spectra were recorded with an UltiMate 3000 Series uHPLC (Thermo Fischer Scientific, Waltman, MA, USA) utilizing a C18 Acquity UPLC BEH column (2.1 × 50 mm, 1.7 µm; Waters, Milford, USA) connected to an amaZon speed ESI-Iontrap-MS (Bruker, Billerica, MA, USA). HPLC parameters were set as follows: solvent A: H_2_O + 0.1% formic acid, solvent B: acetonitrile (ACN) + 0.1% formic acid, gradient: 5% B for 0.5 min increasing to 100% B in 19.5 min, then isocratic condition at 100% B for 5 min, a flow rate of 0.6 mL/min, and Diode-Array Detection (DAD) of 210 nm and 190–600 nm.

High-resolution electrospray ionization mass spectrometry (HR-ESIMS) spectra were recorded with an Agilent 1200 Infinity Series HPLC-UV system (Agilent Technologies, Santa Clara, USA; column 2.1 × 50 mm, 1.7 µm, C18 Acquity UPLC BEH (waters), solvent A: H_2_O +0.1% formic acid; solvent B: ACN + 0.1% formic acid, gradient: 5% B for 0.5 min increasing to 100% B in 19.5 min and then maintaining 100% B for 5 min, flow rate 0.6 mL/min, UV/Vis detection 200–640 nm) connected to a MaXis ESI-TOF mass spectrometer (Bruker) (scan range 100–2500 *m/z*, capillary voltage 4500 V, dry temperature 200 °C).

Optical rotations were recorded in methanol (Uvasol, Merck, Darmstadt, Germany) by using an Anton Paar MCP-150 polarimeter (Seelze, Germany) at 20 °C. UV/Vis spectra were recorded using methanol (Uvasol, Merck, Darmstadt, Germany) with a Shimadzu UV/Vis 2450 spectrophotometer (Kyoto, Japan). ECD spectra were obtained on a J-815 spectropolarimeter (JASCO, Pfungstadt, Germany). Nuclear magnetic resonance (NMR) spectra were recorded at a temperature of 298 K with an Avance III 500 spectrometer (Bruker, Billerica, MA/USA, ^1^H-NMR: 500 MHz and ^13^C-NMR: 125 MHz) and an Ascend 700 spectrometer with 5 mm TCI cryoprobe (Bruker, Billerica, MA/USA, ^1^H-NMR: 700 MHz and ^13^C-NMR: 175 MHz).

**Table 1.** Isolated and reference strains of *Diaporthe* included in this study. # GenBank accession numbers in **bold** were newly generated in this study. The taxonomic novelty is indicated in ***bold italic***.

Species	Isolates^1^	GenBank accession numbers^2^					References
		ITS	*tub2*	*his3*	*tef1*	*cal*	
*Diaportheacaciarum*	CBS 138862^T^	KP004460	KP004509	KP004504	-	-	Crous et al. (2014)
*D.acericola*	MFLUCC 17-0956^T^	KY964224	KY964074	-	KY964180	KY964137	Dissanayake et al. (2017)
*D.alangii*	CFCC 52556^T^	MH121491	MH121573	MH121451	MH121533	MH121415	Yang et al. (2018)
*D.ambigua*	CBS 114015^T^	KC343010	KC343978	KC343494	KC343736	KC343252	Gomes et al. (2013)
*D.amygdali*	CBS 126679^T^	KC343022	KC343990	KC343506	KC343748	KC343264	Gomes et al. (2013)
*D.angelicae*	CBS 111592^T^	KC343026	KC343994	KC343511	KC343752	KC343268	Gomes et al. (2013)
*D.arctii*	CBS 136.25	KC343031	KC343999	KC343515	KC343757	KC343273	Gomes et al. (2013)
*D.arezzoensis*	MFLU 19-2880^T^	MT185503	MT454055	-	-	-	Li et al. (2020)
*D.batatas*	CBS 122.21	KC343040	KC344008	KC343524	KC343766	KC343282	Gomes et al. (2013)
*D.beilharziae*	BRIP 54792^T^	JX862529	KF170921	-	JX862535	-	Thompson et al. (2015)
*D.biguttulata*	ICMP 20657^T^	KJ490582	KJ490403	KJ490524	KJ490461	-	Huang et al. (2015)
***D.breyniae***	CBS 148910^T^	**ON400846**	**ON409186**	**ON409187**	**ON409188**	**ON409189**	Present study
*D.camporesii*	JZB 320143^T^	MN533805	MN561316	-	-	-	Hyde et al. (2020)
*D.caryae*	CFCC 52563^T^	MH121498	MH121580	MH121458	MH121540	MH121422	Yang et al. (2018)
*D.celtidis*	NCYU 19-0357^T^	MW114346	MW148266	-	MW192209	-	Tennakoon et al. (2021)
*D.cerradensis*	CMRP 4331^T^	MN173198	MW751671	MW751663	MT311685	MW751655	Iantas et al. (2021)
*D.chimonanthi*	SCHM 3614^T^	AY622993					Chang et al. (2005)
*D.chinensis*	MFLUCC 19-0101^T^	MW187324	MW245013	-	MW205017	MW294199	de Silva et al. (2021)
*D.chromolaenae*	MFLUCC 17-1422^T^	MH094275	-	-	-	-	Mapook et al. (2020)
*D.cichorii*	MFLUCC 17-1023^T^	KY964220	KY964104	-	KY964176	KY964133	Dissanayake et al. (2017)
*D.cinnamomi*	CFCC 52569^T^	MH121504	MH121586	MH121464	MH121546	-	Yang et al. (2018)
*D.citriasiana*	CBS 134240^T^	JQ954645	KC357459	MF418282	JQ954663	KC357491	Huang et al. (2013)
*D.compacta*	LC3083^T^	KP267854	KP293434	KP293508	KP267928	-	Gao et al. (2016)
*D.convolvuli*	CBS 124654	KC343054	KC344022	KC343538	KC343780	KC343296	Gomes et al. (2013)
*D.cucurbitae*	DAOM 42078^T^	KM453210	KP118848	KM453212	KM453211	-	Udayanga et al. (2015)
*D.cuppatea*	CBS 117499^T^	AY339322	JX275420	KC343541	AY339354	JX197414	Van Rensburg et al. (2006)
*D.discoidispora*	ICMP 20662^T^	KJ490624	KJ490445	KJ490566	KJ490503	-	Huang et al. (2015)
*D.durionigena*	VTCC 930005^T^	MN453530	MT276159	-	MT276157	-	Crous et al. (2020)
*D.endophytica*	CBS 133811^T^	KC343065	KC344033	KC343549	KC343791	KC343307	Gomes et al. (2013)
*D.eres*	CBS 138594^T^	KJ210529	KJ420799	KJ420850	KJ210550	KJ434999	Udayanga et al. (2014)
*D.fici-septicae*	MFLU 18-2588^T^	MW114348	MW148268	-	MW192211	-	Tennakoon et al. (2021)
*D.fructicola*	MAFF 246408^T^	LC342734	LC342736	LC342737	LC342735	LC342738	Crous et al. (2019)
*D.ganjae*	CBS 180.91^T^	KC343112	KC344080	KC343596	KC343838	KC343354	Gomes et al. (2013)
*D.glabrae*	SCHM 3622^T^	AY601918	-	-	-	-	Chang et al. (2005)
*D.goulteri*	BRIP 55657a^T^	KJ197290	KJ197270	-	KJ197252	-	Thompson et al. (2015)
*D.guangdongensis*	ZHKUCC20-0014^T^	MT355684	MT409292	-	MT409338	MT409314	Dong et al. (2021)
*D.gulyae*	BRIP 54025^T^	JF431299	KJ197271	-	JN645803	-	Thompson et al. (2015)
*D.guttulata*	CGMCC 3.20100^T^	MT385950	MT424705	MW022491	MT424685	MW022470	Dissanayake et al. (2020)
*D.helianthi*	CBS 592.81^T^	KC343115	KC344083	KC343599	KC343841	JX197454	Gomes et al. (2013)
*D.heterostemmatis*	SAUCC 194.85^T^	MT822613	MT855810	MT855581	MT855925	MT855692	Sun et al. (2021)
*D.hordei*	CBS 481.92	KC343120	KC344088	KC343604	KC343846	KC343362	Gomes et al. (2013)
*D.hubeiensis*	JZB 320123^T^	MK335809	MK500148	-	MK523570	MK500235	Manawasinghe et al. 2019
*D.infecunda*	CBS 133812^T^	KC343126	KC344094	KC343610	KC343852	KC343368	Gomes et al. (2013)
*D.infertilis*	CBS 230.52^T^	KC343052	KC344020	KC343536	KC343778	KC343294	Guarnaccia and Crous (2017)
*D.kochmanii*	BRIP 54033^T^	JF431295	-	-	JN645809	-	Thompson et al. (2011)
*D.kongii*	BRIP 54031^T^	JF431301	KJ197272	-	JN645797	-	Thompson et al. (2011)
*D.leucospermi*	CBS 111980^T^	JN712460	KY435673	KY435653	KY435632	KY435663	Crous et al. (2011c)
*D.longicolla*	FAU 599^T^	KJ590728	KJ610883	KJ659188	KJ590767	KJ612124	Udayanga et al. (2015)
*D.longispora*	CBS 194.36^T^	KC343135	KC344103	KC343619	KC343861	KC343377	Gomes et al. (2013)
*D.lusitanicae*	CBS 123212^T^	KC343136	KC344104	KC343620	KC343862	KC343378	Gomes et al. (2013)
*D.machili*	SAUCC 194.111^T^	MT822639	MT855836	MT855606	MT855951	MT855718	Huang et al. (2021)
*D.manihotia*	CBS 505.76	KC343138	KC344106	KC343622	KC343864	KC343380	Gomes et al. (2013)
*D.masirevicii*	BRIP 57892a^T^	KJ197277	KJ197257	-	KJ197239	-	Thompson et al. (2015)
*D.mayteni*	CBS 133185^T^	KC343139	KC344107	KC343623	KC343865	KC343381	Gomes et al. (2013)
*D.megalospora*	CBS 143.27	KC343140	KC344108	KC343624	KC343866	KC343382	Gomes et al. (2013)
*D.melonis*	CBS 507.78^T^	KC343142	KC344110	KC343626	KC343868	KC343384	Gomes et al. (2013)
*D.micheliae*	SCHM 3603	AY620820	-	-	-	-	Chang et al. (2005)
*D.middletonii*	BRIP 54884e^T^	KJ197286	KJ197266	-	KJ197248	-	Thompson et al. (2015)
*D.myracrodruonis*	URM 7972^T^	MK205289	MK205291	-	MK213408	MK205290	da Silva et al. (2019)
*D.neoarctii*	CBS 109490	KC343145	KC344113	KC343629	KC343871	KC343387	Gomes et al. (2013)
*D.neoraonikayaporum*	MFLUCC 14-1136^T^	KU712449	KU743988	-	KU749369	KU749356	Doilom et al. (2017)
*D.novem*	CBS 127271^T^	KC343157	KC344125	KC343641	KC343883	KC343399	Gomes et al. (2013)
*D.ovalispora*	ICMP 20659^T^	KJ490628	KJ490449	KJ490570	KJ490507	-	Huang et al. (2015)
*D.pachirae*	COAD 2074^T^	MG559537	MG559541	-	MG559539	MG559535	Milagres et al. (2018)
*D.passifloricola*	CBS 141329^T^	KX228292	KX228387	KX228367	-	-	Crous et al. (2016)
*D.phaseolorum*	CBS 113425	KC343174	KC344142	KC343658	KC343900	KC343416	Gomes et al. (2013)
*D.pseudolongicolla*	CBS 117165^T^	DQ286285	-	-	DQ286259	-	Petrović et al. (2018)
*D.pyracanthae*	CBS142384 ^T^	KY435635	KY435666	KY435645	KY435625	KY435656	Santos et al. (2017)
*D.racemosae*	CBS 143770^T^	MG600223	MG600227	MG600221	MG600225	MG600219	Marin-Felix et al. (2019)
*D.raonikayaporum*	CBS 133182^T^	KC343188	KC344156	KC343672	KC343914	KC343430	Gomes et al. (2013)
*D.rosae*	MFLUCC 17-2658^T^	MG828894	MG843878	-	-	MG829273	Wanasinghe et al. (2018)
*D.rosiphthora*	COAD 2913^T^	MT311196	-	-	MT313692	MT313690	Pereira et al. (2021)
*D.rossmaniae*	CAA 762^T^	MK792290	MK837914	MK871432	MK828063	MK883822	Hilário et al. (2020)
*D.sackstonii*	BRIP 54669b^T^	KJ197287	KJ197267	-	KJ197249	-	Thompson et al. (2015)
*D.sambucusii*	CFCC 51986^T^	KY852495	KY852511	KY852503	KY852507	KY852499	Yang et al. (2018)
*D.schini*	CBS 133181^T^	KC343191	KC344159	KC343675	KC343917	KC343433	Gomes et al. (2013)
*D.schoeni*	MFLU 15-1279^T^	KY964226	KY964109	-	KY964182	KY964139	Dissanayake et al. (2017a)
*D.sclerotioides*	CBS 296.67^T^	KC343193	KC344161	KC343677	KC343919	KC343435	Gomes et al. (2013)
*D.serafiniae*	BRIP 55665a^T^	KJ197274	KJ197254	-	KJ197236	-	Thompson et al. (2015)
*D.siamensis*	MFLUCC 10-0573a	JQ619879	JX275429	-	JX275393	-	Udayanga et al. (2012)
*D.sinensis*	CGMCC 3.19521^T^	MK637451	MK660447	-	MK660449	-	Feng et al. (2019)
*D.sojae*	CBS 139282^T^	KJ590719	KJ610875	KJ659208	KJ590762	KJ612116	Udayanga et al. (2015)
*D.stewartii*	CBS 193.36	FJ889448	-	-	GQ250324	-	Santos et al. (2010)
*D.subellipicola*	KUMCC 17-0153^T^	MG746632	MG746634	-	MG746633	-	Hyde et al. (2018)
*D.subordinaria*	CBS 101711	KC343213	KC344181	KC343697	KC343939	KC343455	Gomes et al. (2013)
*D.tecomae*	CBS 100547	KC343215	KC344183	KC343699	KC343941	KC343457	Gomes et al.(2013)
*D.tectonae*	MFLUCC 12-0777^T^	KU712430	KU743977	-	KU749359	KU749345	Doilom et al. (2017)
*D.tectonendophytica*	MFLUCC 13-0471^T^	KU712439	KU743986	-	KU749367	KU749354	Doilom et al. (2017)
*D.terebinthifolii*	CBS 133180^T^	KC343216	KC344184	KC343700	KC343942	KC343458	Gomes et al. (2013)
*D.thunbergiicola*	MFLUCC 12-0033^T^	KP715097	-	-	KP715098	-	Liu et al. (2015)
*D.tulliensis*	BRIP 62248a	KR936130	KR936132	-	KR936133	-	Crous et al. (2015)
*D.ueckeri*	FAU 656	KJ590726	KJ610881	KJ659215	KJ590747	KJ612122	Huang et al. (2015)
	BRIP 54736j (type of *D.miriciae*)	KJ197283	KJ197263	-	KJ197245	-	Thompson et al. (2015)
*D.unshiuensis*	CGMCC 3.17569^T^	KJ490587	KJ490408	KJ490529	KJ490466	-	Huang et al. (2015)
*D.vexans*	CBS 127.14	KC343229	KC344197	KC343713	KC343955	KC343471	Gomes et al.(2013)
*D.vitimegaspora*	STE-U 2675	AF230749	-	-	-	-	Mostert et al. (2001)
*D.vochysiae*	LGMF 1583^T^	MG976391	MK007527	MK033323	MK007526	MK007528	Noriler et al. (2019)
*D.yunnanensis*	CGMCC 3.18289^T^	KX986796	KX999228	KX999267	KX999188	KX999290	Gao et al. (2017)

^1^BRIP: Queensland Plant Pathology Herbarium, Brisbane, Australia; CBS: Westerdijk Fungal Biodiversity Institute, Utrecht, the Netherlands; CGMCC: Chinese General Microbiological Culture Collection Center, Beijing, China; COAD: Culture Collection of Octávio de Almeida Drumond. Universidade Federal de Viçosa, Viçosa, Brasil; FAU: Isolates in culture collection of Systematic Mycology and Microbiology Laboratory; ICMP: International Collection of Micro-organisms from Plants, Auckland, New Zealand; KUMCC: Kumming Institute of Botany, Kumming, China; LGMF, Laboratório de Genética de Microrganismos (LabGeM) culture collection, at the Federal University of Paraná, Brazil; MAFF: Ministry of Agriculture, Forestry and Fisheries, Tokyo, Japan; MFLUCC: Mae Fah Luang University Culture Collection, Chiang Rai, Thailand; SAUCC: Shandong Agricultural University Culture Collection, Shandong, China; STE-U: Department of Plant Pathology, Stellenbosch University, Stellenbosch, South Africa; URM: Culture Collection at the Universidade Federal de Pernambuco, Recife, Brazil; VTCC: Vietnam Type Culture Collection, Center of Biotechnology, Vietnam National University, Hanoi, Vietnam; ZHKUCC: Culture Collection of Zhongkai University of Agriculture and Engineering, Guangzhou, China. ^T^ indicates type material. ^2^ITS: internal transcribed spacers and intervening 5.8S nrDNA; *tub2*: partial β-tubulin gene; *his3*: partial histone H3 gene; *tef1*: partial elongation factor 1-alpha gene; *cal*: partial calmodulin gene.

### ﻿Small-scale fermentation and extraction

The fungus was cultivated in three different liquid media (YM 6.3 medium: 10g/mL malt extract, 4g/mL, yeast extract, 4g/mL, D-glucose and pH = 6.3, Q6 ½ medium: 10 g/mL glycerin, 2.5 g/mL D-glucose, 5 g/mL cotton seed flour and pH = 7.2; ZM ½ medium: 5 g/mL molasses, 5 g/mL oatmeal, 1.5 g/mL D-glucose, 4 g/mL saccharose, 4 g/mL mannitol, 0.5 g/mL edamin, ammonium sulphate 0.5 g/mL, 1.5 g/mL calcium carbonate and pH = 7.2) (Chepkirui et al. 2016). A well-grown 14-day-old mycelial culture grown on YM agar was cut into small pieces using a cork borer (7mm), and five pieces used for inoculation of 500 mL Erlenmeyer flasks containing 200 mL of media. The cultures were incubated at 23 °C on a rotary shaker at 140 rpm. The growth of the fungus was monitored by checking the amount of free glucose daily using Medi-Test glucose strips (Macherey Nagel, Düren, Germany). The fermentation was terminated three days after glucose depletion and the biomasses and supernatants were separated via vacuum filtration. Afterwards, the supernatants were extracted with equal amount of ethyl acetate (200 mL) and filtered through anhydrous sodium sulphate. The resulting ethyl acetate extracts were evaporated to dryness *in vacuo* (Rotary Evaporator: Heidolph Instruments GmbH & Co. KG, Schwabach, Germany; pump: Vacuubrand GmbH & Co. KG, Wertheim am Main, Germany) at 40 °C. The mycelia were extracted with 200 mL of acetone in an ultrasonic bath (Sonorex Digital 10 P, Bandelin Electronic GmH & Co. KG, Berlin, Germany) at 40 °C for 30 min, filtered and the organic phase evaporated. The volume of the remaining aqueous phase was adjusted with an equal amount of distilled water and subjected to the same procedure as described for the supernatants.

The small-scale cultivation of *Diaporthebreyniae* was also carried out on YM agar medium and rice solid medium (BRFT, brown rice 28 g as well as 0.1 L of base liquid (yeast extract 1 g/L, di-sodium tartrate di-hydrate 0.5 g/L, KH_2_PO_4_ 0.5 g/L) (Becker et al. 2020a). Briefly, the fungus was grown on a YM agar plate and the mycelia was extracted with 200 mL of ethyl acetate in an ultrasonic water bath at 40 °C for 30 min, filtered and the filtrate evaporated to dryness *in vacuo* at 40 °C. For BFRT medium, three small pieces of the mycelial culture grown on а YM agar plate were inoculated into a 250 ml Erlenmeyer flask containing 100 mL of YM 6.3 medium. The seed culture was incubated at 23 °C under shake condition at 140 rpm. After 5 days, 10 mL of this seed culture were transferred to a 500 mL Erlenmeyer flask containing BRFT medium and incubated for 28 days at 23 °C. Afterwards, extraction of the culture was performed following the same procedure as above mentioned for the mycelia obtained from the liquid cultures.

### ﻿Scale-up fermentation in shake flask batches and extraction

Preliminary results obtained from small-scale screening suggested that the fungus grew and produced best in ZM ½ medium (Suppl. material 1: Figs S1, S2). Moreover, the extracts obtained from the fungal culture in ZM ½ were active against *Bacillussubtilis* and *Mucorplumbeus*. Therefore, this medium was selected for scale-up fermentation. Three well-grown 14-day-old YM agar plate of the mycelial culture were cut into small pieces using a 7 mm cork borer and 5 pieces inoculated in 10 × 500 mL Erlenmeyer flasks containing 200 mL of ZM ½ medium. The culture was incubated at 23 °C on a rotary shaker at 140 rpm for 11 days. Fermentation was aborted 3 days after the depletion of free glucose. The mycelia and supernatant from the batch fermentation were separated *via* vacuum filtration. The mycelia were extracted with 3 × 500 mL of acetone in an ultrasonic water bath at 40 °C for 30 min. The extracts were combined and the solvent evaporated *in vacuo* (40 °C). The remaining water phase was subjected to the same procedure as previously described for the mycelial fraction in small-scale extraction, repeating the extraction step 3 times, yielding 955 mg dark brown solid-like extract. The supernatant (2 L) was extracted with equal amount of ethyl acetate and filtered through anhydrous sodium sulphate. The resulting ethyl acetate extract was evaporated to dryness *in vacuo* to afford 251 mg of extract.

### ﻿Isolation of secondary metabolites

The mycelial and the supernatant extracts from shake flask batch fermentation dissolved in methanol were centrifuged by means of a centrifuge (Hettich Rotofix 32 A, Tuttlingen, Germany) for 10 min at 4000 rpm. Afterwards, the mycelia and supernatant extracts were fractionated separately using preparative reverse phase HPLC (Büchi, Pure C-850, 2020, Switzerland). VP Nucleodur 100-5 C18ec column (150 × 40 mm, 7 µm: Machery-Nagel, Düren, Germany) was used as stationary phase. Deionized water (Milli-Q, Millipore, Schwalbach, Germany) supplemented with 0.1% formic acid (FA) (solvent A) and acetonitrile (ACN) with 0.1% FA (solvent B) were used as the mobile phase. The elution gradient used for fractionation was 5–35% solvent B for 20 min, 35–80% B for 30 min, 80–100% B for 10 min and thereafter isocratic condition at 100% solvent B for 15 min. The flow rate was set to 30 mL/min and UV detection was carried out at 210, 320 and 350 nm. For the supernatant extract, 13 fractions (F1-F13) were selected according to the observed peaks, and further analysis of the fractions using HPLC-MS revealed that four of the obtained fractions constituted pure compounds. Using the same elution conditions as mentioned, the mycelia extract afforded 17 fractions (F1–F17) selected from the observed peaks. HPLC-MS analysis of the obtained fractions revealed that seven fractions constituted pure compounds. The compounds obtained from mycelial and supernatant extracts were combined according to their respective HPLC-ESIMS retention time and molecular weight. Compound **1** (55.2 mg, *t_R_* = 7.80 min) was obtained from both the mycelium and supernatant extracts as well as compounds **2** (10.9 mg, *t_R_* = 6.27 min), **3** (2.6 mg, *t_R_* = 11.42 min) and **4** (5.6 mg, *t_R_* = 9.49 min). Compounds **5** (3.6 mg, *t_R_* = 13.46 min), **11** (0.7 mg, *t_R_* = 12.11 min) and **12** (2.0 mg, *t_R_* = 3.83 min) were only isolated from the mycelial extract. Fractions F4 from both the mycelium and supernatant extracts were combined and purified using an Agilent Technologies 1200 Infinity Series semi-preparative HPLC instrument (Waldbronn, Germany). The elution gradient used was 20–30% solvent B for 5 min followed by isocratic condition at 30% B for 25 min and thereafter increased gradient from 30–100% B for 5 min. VP Nucleodur 100-5 C18ec column (250 × 10 mm, 5 µm: Machery-Nagel, Düren, Germany) was used as stationary phase and the flow rate was 3 mL/min. These fractions afforded compound **13** (2.34 mg, *t_R_* = 5.13 min). Fractions F13 and F14 from the mycelial extract were combined with F12 from the supernatant as they contained the same compounds. The pooled fractions were purified by preparative reverse phase HPLC (Büchi, Pure C-850, 2020, Switzerland). VP Nucleodur 100-5 C18ec column (250 × 21 mm, 5 µm: Machery-Nagel, Düren, Germany) was used as stationary phase with a flow rate of 15 mL/min and an elution gradient of 5–70% solvent B for 5 min, followed by isocratic conditions at 70% B for 25min, and thereafter increased gradient from 70–100% B for 5 min. These fractions afforded compound **9** (10.5 mg, *t_R_* = 13.02 min) and sub-fraction G1. Sub-fraction G1 was further purified using an Agilent Technologies 1200 Infinity Series semi-preparative HPLC with the elution gradient starting from 65–70% B for 5 min followed by isocratic condition at 70% B for 25 min and thereafter increased gradient from 70–100% B for 5 min to afford compounds **7** (1.4 mg, *t_R_* = 13.91 min) and **8** (0.52 mg, *t_R_* = 13.56 min). Fraction F15 from the mycelium were also purified using the same instrument and same elution conditions as described for sub-fraction G1. This fraction afforded compounds **6** (1.1 mg, *t_R_* = 14.02 min) and **10** (1.7 mg, *t_R_* = 13.58 min).

Note: The given retention times were obtained from HPLC-ESIMS following the HPLC parameters as described in the general experimental procedures.

### ﻿Antimicrobial assay

The antifungal and antibacterial activities (Minimum Inhibition Concentration, MIC) of all extracts obtained from small-scale fermentation were determined in serial dilution assays as described previously (Chepkirui et al. 2016; Becker et al. 2020b) against *Bacillussubtilis*, *Candidatenuis*, *Escherichiacoli and Mucorplumbeus*. The assays were carried out in 96-well microtiter plates in YM 6.3 medium for filamentous fungi and yeast and MHB medium (Müller-Hinton Broth: SN X927.1, Carl Roth GmbH, Karlsruhe, Germany) for bacteria. Starting concentration for all extracts were 300 µg/mL. In addition, the antimicrobial activity of the isolated pure compounds was also assessed as previously described (Matio Kemkuignou et al. 2020) against a panel of bacteria and fungi including *Pichiaanomala* DSM 6766, *Schizosaccharomycespombe* DSM 70572, *Mucorhiemalis* DSM 2656, *Candidaalbicans* DSM 1665, and *Rhodotorulaglutinis* DSM 10134 for fungal microorganisms, *Bacillussubtilis* DSM 10, *Staphylococcusaureus* DSM 346 and *Mycobacteriumsmegmatis* ATCC 700084 for Gram-positive bacteria, *Acinetobacterbaumannii* DSM 30008, *Chromobacteriumviolaceum* DSM 30191, *Escherichiacoli* DSM 1116 and *Pseudomonasaeruginosa* for Gram-negative bacteria. Starting concentration for tested compounds was adjusted to 66.7 µg/mL.

### ﻿Cytotoxicity assay

The *in vitro* cytotoxicity (IC_50_) of the isolated metabolites against several mammalian cell lines (human endocervical adenocarcinoma KB 3.1, mouse fibroblasts L929, squamous cancer A431, breast cancer MCF-7, lung cancer A549, ovary cancer SK-OV-3 and prostate cancer PC-3) was determined by colorimetric tetrazolium dye MTT assay using epothilone B as a positive control in accordance to our previously reported experimental procedure (Becker et al. 2020b).

## ﻿Results and discussion

### ﻿Phylogenetic study

**Figure 1.**
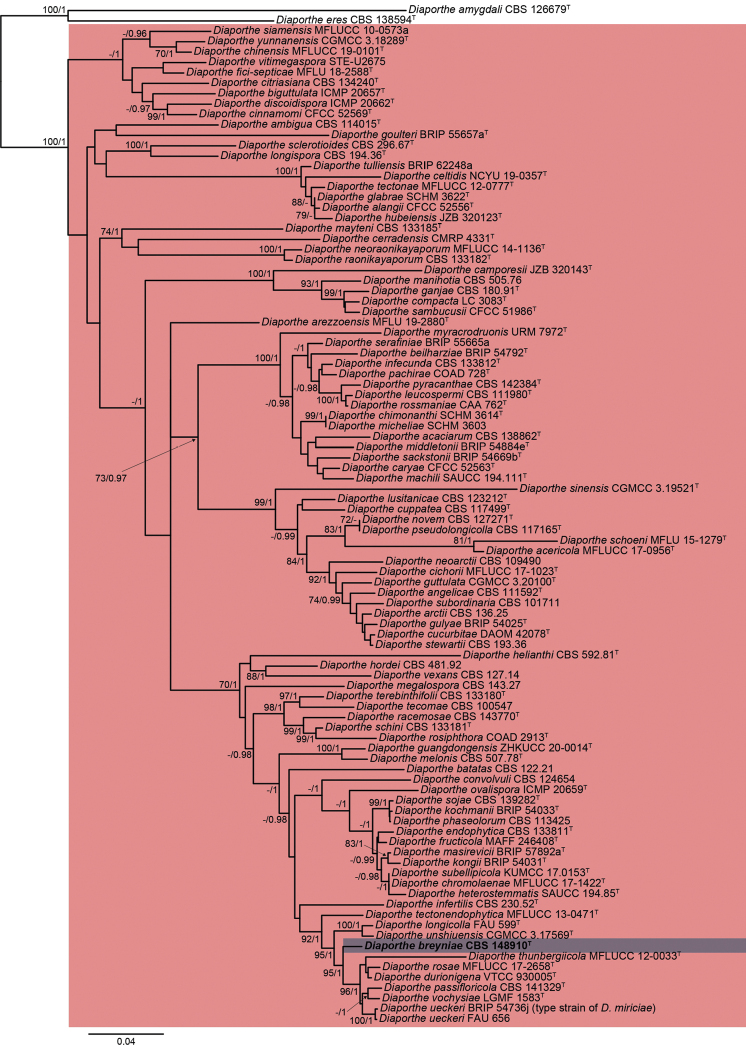
ML (lnL = -28100.2019) phylogram obtained from the combined ITS, *cal*, *his3*, *tef1* and *tub2* sequences of our strain and related *Diaporthe* spp. *Diaportheamygdali*CBS 126679^T^ and *D.eres*CBS 138594^T^ were used as an outgroup. Bootstrap support values ≥ 70/Bayesian posterior probability scores ≥ 0.95 are indicated along branches. Branch lengths are proportional to distance. New taxon is indicated in bold. Type material of the different species is indicated with ^T^.

The lengths of the fragments of the first phylogenetic inference using the five previously mentioned loci used in the combined dataset for the tree including all *Diaporthe* spp. were 454 bp (ITS), 318 bp (*cal*), 296 bp (*his3*), 153 bp (*tef1*) and 487 bp (*tub2*), comprising in total 341 taxa. The length of the final alignment was 1708 bp. The inferred phylogeny with the best maximum likelihood score with bootstrap support (bs) values mapped onto branch bipartitions is shown in Suppl. material 1: Fig. S100. The here studied strain was located in a clade with 92% bs including 341 taxa, including species belonging to the *D.sojae* complex. A second molecular phylogeny was inferred including sequences of the same loci, but restricted to the aforementioned clade, including 98 taxa. The lengths of the fragments used in the combined dataset were 572 bp (ITS), 449 bp (*cal*), 373 bp (*his3*), 452 bp (*tef1*) and 862 bp (*tub2*), totaling 2708 bp for the final alignment. Fig. 1 shows the consensus ML tree, including bs and Bayesian posterior probability (pp) values at the nodes. Our strain was located in an independent branch distant from other species of *Diaporthe*, demonstrating that this represented a new species, which is introduced here as *D.breyniae*. Unfortunately, the new species lacked sporulation in all media tested in the present study. Therefore, the introduction of it is based only on molecular data.

### ﻿Taxonomy

#### ﻿ Diaporthe breyniae

Taxon classificationFungi

Y. Marín & C. Lamb. sp. nov.

F343AAD4-1A66-5164-A82C-57BFBA0E28B9

843243

##### Etymology.

Name refers to the host genus that this fungus was isolated from, *Breynia*.

##### Description.

Not sporulated. *Diaporthebreyniae* differs from its closest phylogenetic neighbour, *D.durionigena* by unique fixed alleles in three loci based on alignments of the separate loci included in the supplementary material: ITS positions 93 (indel), 159 (G), 436 (T), 437 (C), 451 (G), 453 (A), 485 (C); *tef1* positions 46 (A), 62 (G), 80 (T), 100 (G), 146 (T), 274 (indel), 304 (A), 310 (G), 313 (C), 339 (T), 343 (A), 385 (G); *tub2* positions 393 (A), 402 (indel), 426 (A), 565 (C), 675 (T), 713 (G), 770 (T).

##### Culture characters.

Colonies on PDA reaching 55–70 mm in 2 weeks, greyed yellow (161A) with a white ring and transparent margins, lobate, cottony, raised, margins filamentous to fimbriate; reverse greyed yellow (161A–D) with transparent margins. Colonies on MEA covering the surface of the Petri dish in 2 weeks, white with greyed yellow center (161A), velvety to cottony, flat to raised in some zones, margins filamentous to fimbriate; reverse greyed yellow (162A–B). Colonies on OA covering the surface of the Petri dish in 2 weeks, white with greyed yellow ring (161D), velvety, flat, margins filamentous to fimbriate; reverse grey brown (199D).

##### Specimen examined.

Cameroon, Kala mountain, on leaves of *Breyniaoblongifolia*, 02 Jan. 2019, *S.C.N. Wouamba* (holotype: CBS H-24920, culture ex-type CBS 148910 = STMA 18284).

##### Notes.

*Diaporthebreyniae* is introduced based only on molecular data since sporulation could not be induced in any media used. This species is located in a well-supported clade (97% bs / 1 pp) together with *D.durionigena*, *D.passifloricola*, *D.rosae*, *D.thunbergiicola*, *D.ueckeri* and *D.vochysiae*. The latter species has only been reported from Brazil occurring on different hosts, i.e. *Stryphnodendronadstringens* (Fabaceae, Fabales) and *Vochysiadivergens* (Vochysiaceae, Myrtales) (Noriler et al. 2019). *Diaporthedurionigena* has been only isolated from *Duriozibethinus* (Malvaceae, Malvales) in Vietnam (Crous et al. 2020, 2021). *Diaporthepassifloricola* has been found on *Passiflorafoetida* (Passifloraceae, Malpighiales) and *Citrus* spp. (Rutaceae, Sapindales) in China and Malaysia (Crous et al. 2016; Chaisiri et al. 2021; Dong et al. 2021), while *D.rosae* has been isolated from *Rosa* sp. (Rosaceae, Rosales), *Magnoliachampaca* (Magnoliaceae, Magnoliales) and *Sennasiamea* (Fabaceae, Fabales) in Thailand (Perera et al. 2018; Wanasinghe et al. 2018). *Diaportheueckeri* (syn. *D.miriciae*, Gao et al. 2016) has been reported in Australia, Colombia and the USA, on *Cucumismelo* (Cucurbitaceae, Cucurbitales), *Glycinemax* (Fabaceae, Fabales) and *Helianthusannuus* (Asteraceae, Asterales) (Thompson et al. 2015; Udayanga et al. 2015; López-Cardona et al. 2021). *Diaporthethunbergiicola* has been only isolated from *Thunbergialaurifolia* (Acanthaceae, Lamiales) in Thailand (Liu et al. 2015). The new species *D.breyniae* is the only of these species reported on *Breynia* (Phyllanthaceae, Malpighiales) in Africa. In fact, to the best of our knowledge, this is the first species of *Diaporthe* reported in Cameroon and occurring in this host.

### ﻿Structure elucidation of compounds 1–13

**Figure 2.**
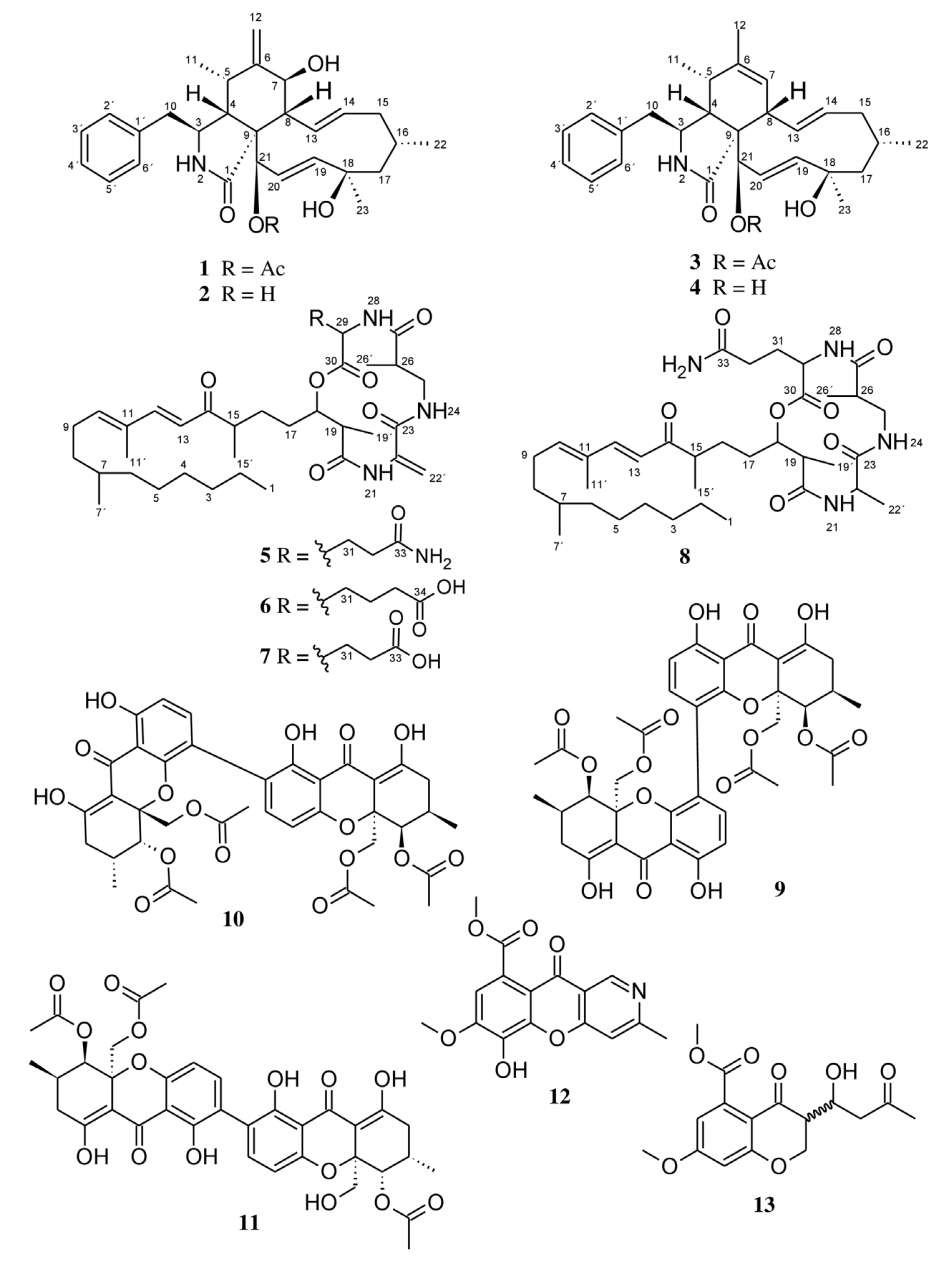
Chemical structures of compounds **1**–**13** isolated from *Diaporthebreyniae*.

Cultivation trials carried out on *Diaporthebreyniae* in different culture media including YM 6.3, Q6 ½, ZM ½, rice solid and YM agar highlighted its potential for producing secondary metabolites. During antimicrobial screening of the extracts, the fungus revealed significant antifungal and antibacterial activity against *Mucorhiemalis* and *Bacillussubtilis* respectively, especially when cultured in ZM ½ medium, encouraging more detailed examination. Investigation into the chemistry of *Diaporthebreyniae* led to the isolation of two new secondary metabolites (**7**, **8**) together with eleven known compounds (**1**–**4**, **5**, **6**, **9**–**13**) from the EtOAc extracts of a 2 L scale-up ZM ½ liquid medium of the fungus (Fig. 2). The structure elucidation of **1**–**13** was determined by detailed spectroscopic analysis of their 1D and 2D NMR data in combination with their HR-ESIMS data.

**Figure 3.**
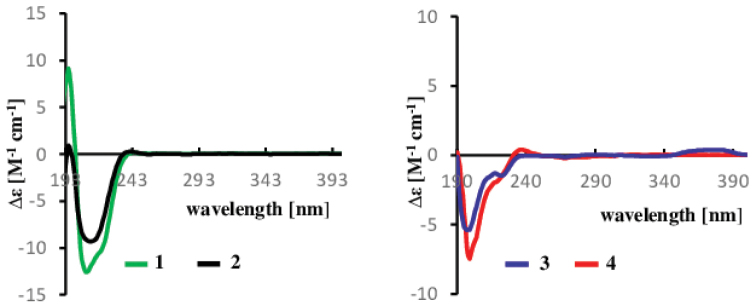
ECD spectra of compounds **1**–**4** in MeOH.

HR-ESI(+)MS and NMR spectroscopic analysis identified compounds **1**–**3** as cytochalasin H (**1**) (Suppl. material 1: Figs S3–S10) (Beno et al. 1977; Shang et al. 2017), deacetylcytochalasin H or cytochalasin J (**2**) (Suppl. material 1: Figs S11–S17) (Cole et al. 1981; Shang et al. 2017) and cytochalasin RKS-1778 (**3**) (Suppl. material 1: Figs S18–S24) (Kakeya et al. 1997) respectively. The absolute configuration of cytochalasins H (**1**) and J (**2**) was confirmed by comparing their optical rotation values ([α]^20^_D_ +55.7 (c 0.158, MeOH) for 1 and [α]^20^_D_ +35.3 (c 0.394, MeOH) for **2**) and ECD spectrum (Fig. 3) with those reported in the literature (Shang et al. 2017; Ma et al. 2021). The literature reports only the relative configuration of compound **3** (*rel*- (3*S*, 4*R*, 5*S*, 8*S*, 9*S*, 13*E*, 16*S*, 18*R*, 19*E*, 21*R*)) (Kakeya et al. 1997), therefore, its absolute configuration was investigated by comparison of its ECD spectrum with that of cytochalasins H (**1**) and J (**2**) (Fig. 3). The ECD spectrum of **3** showed negative (~ 200 nm) cotton effect, the shape of which matched with that of compounds **1** and **2**. Thus, the hitherto unestablished absolute configuration of cytochalasin RKS-1778 (**3**) was confirmed to be 3*S*, 4*R*, 5*S*, 8*R*, 9*R*, 13*E*, 16*S*, 18*R*, 19*E*, 21*R*.

**Figure 4.**
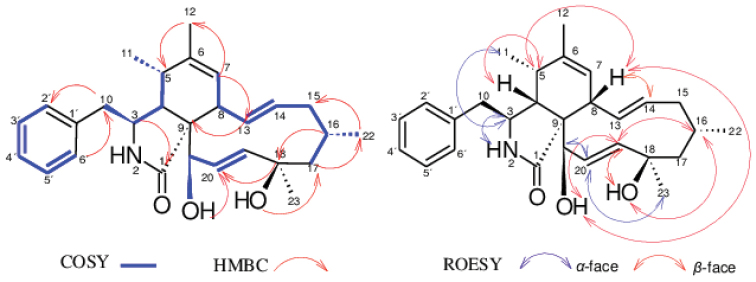
Selected ^1^H–^1^H COSY, NOESY and HMBC correlations of 4.

HR-ESI (+) MS analysis of **4** isolated as a yellowish oil afforded pseudo-molecular ion peaks [M+H]^+^ at *m/z* 436.2852 and [M+Na]^+^ at *m/z* 458.2665 attributed to the molecular formula C_28_H_37_NO_3_ (11 degrees of unsaturation). Comparison of the 1D and 2D NMR spectroscopic data for 4 (DMSO-d_*6*_) with those for **3** (Table 2) revealed that both compounds are closely related, with compound **4** being the deacetylated derivative of **3**. This was confirmed on the ^1^H NMR spectrum of compound **4** by the absence of the methyl group H_3_-25 and on its ^13^C NMR spectrum by the absence of both C-24 carbonyl group and C-25 methyl group as visible on the NMR data recorded for compound **3** (Table 2). The relative configuration of compound **4** was determined by analysis of the coupling constants and NOESY correlations. The E-geometry of the ∆^13,14^ and ∆^19,20^ double bonds in the macrocyclic ring was determined based on the large coupling constants *J* = 15.3 and 16.7 Hz observed between H-13 and H-14 and between H-19 and H-20 respectively. The small coupling constant *J* = 4.4 Hz observed between H-4 and H-5 confirmed their *cis* relationship (Kakeya et al. 1997). The NOESY spectrum arbitrarily suggested *α*-orientation of H-3, H-11, H-21 and H-23 based on the observed correlations between H-3/H-11, H-20/H-21 and H-20/H-23,while the *β*-orientation of H-4, H-5, H-8, H-16, 18-OH and 21-OH were apparent from a network NOESY correlations between H-4/H-5, H-5/H-8, H-8/21-OH, 21-OH/H-19, H-19/H-16 and H-16/18-OH (Fig. 4). These correlations allowed the assignment of the relative configuration of compound **4** as either *rel*- (3*S*, 4*R*, 5*S*, 8*S*, 9*S*, 13*E*, 16*S*, 18*R*, 19*E*, 21*R*) or *rel*- (3*R*, 4*S*, 5*R*, 8*R*, 9*R*, 13*E*, 16*R*, 18*S*, 19*E*, 21*S*). In addition, the optical rotation value of **4** ([α]^20^_D_ -17.6 (c 0.278, MeOH)) approximating that reported in the literature for **3** ([α]^20^_D_ -20 (c 0.05, MeOH, Kakeya et al. 1997) revealed that both compounds are levorotatory, and this suggested the stereochemistry of **4** to be identical to that of **3**. The latter assumption was confirmed by comparing the ECD spectrum of **4** with those of compounds **1**, **2** and **3**. The same negative Cotton effect (~ 200 nm) observed for all those compounds unambiguously certified the absolute configuration of compound **4** established as 3*S*, 4*R*, 5*S*, 8*S*, 9*S*, 13*E*, 16*S*, 18*R*, 19*E*, 21*R*. Thus, the structure of **4** was determined. This compound was regarded new while the current study has been under review, but concurrently it was published as phomopchalasin N by Chen et al. (2022). Interestingly, the authors also isolated it from a member of the genus *Diaporthe*, but inadvertently referred to their producer organism under the outdated name “*Phomopsis*”. We have decided to leave our complete data on the structure elucidation in the manuscript, so they can be compared with those of Chen et al. (2022) by other scientists, but the compounds are indeed identical.

**Table 2.** ^13^C (125 MHz) and ^1^H-NMR (500 MHz) spectroscopic data (DMSO-*d_6_*, *δ* in ppm) of compounds **3**, **4**.

	3		4	
No.	*δ*_C_, type	*δ*_H_ (*J* in Hz)	*δ*_C_, type	*δ*_H_ (*J* in Hz)
1	174.3, C	-	175.9, C	-
2-NH	-	7.89, s	-	7.57, s
3	53.9, CH	3.16, m	53.8, CH	3.14, q (4.9)
4	50.5, CH	2.02, t (4.1)	50.9, CH	2.47, t (4.4)
5	34.1, CH	2.18, m*	34.3, CH	2.3, m
6	137.3, C	-	137.1, C	-
7	126.8, CH	5.21*	127.4, CH	5.17, br s
8	42.3, CH	3.06 br d (9.9)	40.9, CH	3.04, br d (9.8)
9	55.5, C	-	57.2, C	-
10	44.0, CH_2_	2.59, dd (13.2, 7.4) 2.74, dd (13.1, 5.3)	43.6, CH_2_	2.65, dd (13.6, 5.2) 2.70, dd (13.6, 5.2)
11	12.8, CH_3_	0.64, d (7.2)	13.0, CH_3_	0.84, d (7.3)
12	19.2, CH_3_	1.62, s	19.3, CH_3_	1.63, s
13	129.2, CH	5.73, dd (15.7, 10.1)	129.7, CH	5.66, dd (15.3, 10.1)
14	133.5, CH	5.08, ddd (15.3, 10.9, 4.5)	132.8, CH	5.02, ddd (15.3, 11.0, 4.4)
15	42.1, CH_2_	1.57, m* 1.89, br dd (12.4, 4.3)	42.3, CH_2_	1.52, q (12.5) 1.84, br dd (12.5, 4.2)
16	27.6, CH	1.69, m	27.7, CH	1.69, m
17	53.1, CH	1.37, br dd (13.6, 3.2) 1.59, m*	53.1, CH_2_	1.34, br dd (13.4, 3.3) 1.60, dd (13.6, 3.3)
18	72.1, C	-	72.2, C	-
19	137.3, CH	5.36, dd (16.6, 2.3)	136.2, CH	5.61, dd (16.7, 2.4)
20	125.1, CH	5.71, dd (16.9, 2.4)	130.7, CH	5.76, dd (16.7, 2.4)
21	75.7, CH	5.23*	73.7, CH	3.63, br s
22	25.8, CH_3_	0.94, d (7.3)	25.9, CH_3_	0.93, d (7.1)
23	31.0, CH_3_	1.13, s	31.5, CH_3_	1.12, s
24	169.3, C	-	-	-
25	20.2, CH_3_	2.18, s	-	-
1´	136.8, C	-	136.9, C	-
2´/6´	129.6, CH (x2)	7.12, d (7.0)	129.8, CH (x2)	7.21*
3´/5´	127.9, CH (x2)	7.29, t (7.5)	127.7, CH (x2)	7.29, t (7.7)
4´	126.0, CH	7.21, t (7.5)	126.0, CH	7.21*
18-OH	-	4.36, s	-	4.17, s
21-OH	-	-	-	4.88, br d (5.6)

*overlapping signals, assignments were supported by HSQC and HMBC

Compounds **5** and **6** were readily identified as the known fusaristatins A and B respectively, after careful analysis of their HR-ESI (+) MS and NMR spectroscopic data (Suppl. material 1: Figs S34–S47). Fusaristatins A (**5**) and B (**6**) were first reported in 2007 from an endophytic *Fusarium* sp. (Shiono et al. 2007) and so far, only fusaristatin A (**5**) has been isolated from *D.phaeseolorum* and *D.longicolla* (syn: *Phomopsislongicolla*) (Santos et al. 2011; Choi et al. 2013; Cui et al. 2017). Therefore, this is the first report for the isolation of fusaristatin B (**6**) from the genus *Diaporthe*. In addition, two new derivatives of fusaristatin A (**7**, **8**) were isolated from *Diaporthebreyniae* and their structures were established by intensive analysis of their 1D and 2D NMR spectroscopic data in combination with HR-ESIMS data and by comparison with the data reported in the literature for fusaristatins A (**5**) and B (**6**) (Shiono et al. 2007).

**Figure 5.**
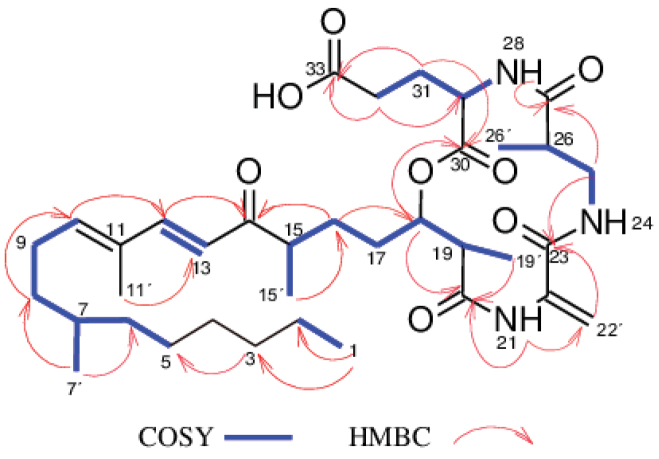
Selected ^1^H–^1^H COSY and HMBC correlations of **7**.

The molecular formula of compound **7**, isolated as a colorless oil, was determined to be C_36_H_57_N_3_O_8_ from the HR-ESIMS (positive mode) which showed pseudo-molecular ion peaks [M+H]^+^ at *m/z* 660.4219 and [M+Na]^+^ at *m/z* 682.4024, indicating 10 degrees of unsaturation. Inspection of the molecular formula of **7** (C_36_H_57_N_3_O_8_) in comparison to that of **5** (C_36_H_58_N_4_O_7_) suggested that an amino group (-NH_2_) in compound **5** could probably have been replaced by a hydroxyl group (-OH) in compound **7**. Intensive analysis of 1D and 2D NMR spectroscopic data (C_5_D_5_N) of compound **7** in comparison to that of **5** indicated that most signals in **7** were the same as those for **5** (Table 3), implying that **7** and **5** are closely related. The only difference was observed on the ^1^H NMR spectrum where the signal corresponding to the amino group 34-NH_2_ (*δ*_H_ 8.34) in compound **5** was absent in compound **7** (Table 3). Moreover, in the HMBC spectrum of **7**, correlations from H-31 to C-30, H-31/H-32 to C-33 suggested the presence of a glutamic acid residue instead of a glutamine residue as observed in **5**. Based on ^1^H-^1^H COSY, ^1^H-^13^C HSQC and ^1^H-^13^C HMBC experiments (Fig. 5), the signals of all protons and carbons in the molecule were unambiguously assigned and compound **7** was identified as a new derivative of fusaristatin A named fusaristatin G.

**Table 3.** ^13^C and ^1^H-NMR spectroscopic data (pyridine-*d_5_*, *δ* in ppm) of compounds **5**, **7, 8**.

	5^a^		7^b^		8^b^	
No.	*δ*_C_, type	*δ*_H_ (*J* in Hz)	*δ*_C_, type	*δ*_H_ (*J* in Hz)	*δ*_C_, type	*δ*_H_ (*J* in Hz)
1	14.7, CH_3_	0.88*	14.7, CH_3_	0.87*	14.5, CH_3_	0.87, t (6.9)*
2	23.4, CH_2_	1.20 ~ 1.31, m*	23.4, CH_2_	1.20 ~ 1.31, m*	23.1, CH_2_	1.20 ~ 1.31, m*
3	32.6, CH_2_	1.20 ~ 1.31, m*	32.6, CH_2_	1.20 ~ 1.31, m*	32.3, CH_2_	1.20 ~ 1.31, m*
4	27.7, CH_2_	1.20 ~ 1.31, m*	27.7, CH_2_	1.20 ~ 1.31, m*	27.4, CH_2_	1.20 ~ 1.31, m*
5	30.3, CH_2_	1.20 ~ 1.31, m*	30.3, CH_2_	1.20 ~ 1.31, m*	30.1, CH_2_	1.20 ~ 1.31, m*
6	37.5, CH_2_	1.09, m* 1.20-1.31, m*	37.5, CH_2_	1.09, m* 1.20 ~ 1.31, m*	37.3, CH_2_	1.09, m* 1.20 ~ 1.31, m*
7	33.2, CH	1.39, m*	33.2, CH	1.40, m*	32.9, CH	1.38, m*
7´	20.0, CH_3_	0.88*	20.0, CH_3_	0.88*	19.8, CH_3_	0.87, d (6.9)*
8	36.8, CH_2_	1.20 ~ 1.31* 1.40, m*	36.9, CH_2_	1.20 ~ 1.31, m* 1.40, m*	36.6, CH_2_	1.20 ~ 1.31, m* 1.40, m*
9	27.2, CH_2_	2.19, m*	27.2, CH_2_	2.18, m	27.0, CH_2_	2.21, m*
10	144.5, CH	6.03, br t (7.4)	144.5, CH	6.03, br t (7.2)	144.3, CH	6.01, t (7.4)
11	133.9, C	-	140.0, C	-	133.9, C	-
11´	12.6, CH_3_	1.83, s	12.7, CH_3_	1.83, s	12.5, CH_3_	1.85, s
12	148.4, CH	7.54, d (15.7)	148.3, CH	7.56, d (15.7)	148.2, CH	7.55, d (15.7)
13	123.7, CH	6.40, d (15.7)	123.8, CH	6.40, d (15.7)	123.6, CH	6.45, d (15.7)
14	203.8, C	-	203.6, C	-	204.1, C	-
15	44.5, CH	2.84, m	44.6, CH	2.80 ~ 2.88, m*	44.6, CH	2.88, m
15´	17.7, CH_3_	1.10, d (6.9)	17.6, CH_3_	1.10, d (6.9)	17.1, CH_3_	1.13, d (6.9)
16	28.5, CH_2_	1.57, m 1.93 ~ 2.00, m*	28.3, CH_2_	1.54, m 1.93 ~ 2.00, m*	29.1, CH_2_	1.66, m 2.04, m*
17	30.3, CH_2_	1.87, m 1.93 ~ 2.00, m*	30.2, CH_2_	1.84, m 1.93 ~ 2.00, m*	31.3, CH_2_	1.97, m 2.04, m*
18	77.3, CH	5.44, m	77.2, CH	5.48, m	77.6, CH	5.45, m
19	44.6, CH	3.03, quin (7.0)	44.5, CH	3.05, quin (7,0)	45.6, CH	2.95, m
19´	15.8, CH_3_	1.30, d (7.0)*	15.9, CH_3_	1.33, d (7.3)*	14.9, CH_3_	1.35, d (7.3)
20	173.9, C	-	174.0, C	-	173.5, C	-
21-NH	-	10.43, s	-	10.55, s	-	8.15, br s
22	139.6, C	-	139.8, C	-	50.9, CH	4.89, m
22´	114.6, CH_2_	5.60, s 6.24, s	114.3, CH_2_	5.59, s 6.22, s	17.3, CH_3_	1.65, d (7.1)
23	165.2, C	-	165.3, C		173.9, C	-
24-NH	-	7.81, br s	-	7.88, br t (6.1)	-	7.96, br s
25	43.0, CH_2_	3.81, dt (13.5, 6.9) 3.92, dt (13.3, 4.9)	43.0, CH_2_	3.78, dt (13.5, 6.7) 3.94, m	42.1, CH_2_	3.49, dt (13.6, 3.8) 4.04, dt (13.5, 7.9)
26	42.7, CH	2.87, m	42.7, CH	2.92, m	42.8, CH	2.85, m
26´	15.5, CH_3_	1.30, d (7.0)*	15.8, CH_3_	1.33, d (7.3)*	14.9, CH_3_	1.22, d (7.3)
27	175.0, C	-	175.1, C	-	175.4, C	-
28-NH	-	9.06, br d (7.5)	-	9.11, br d (7.7)	-	8.90, br d (7.7)
29	53.6, CH	5.13, dd (14.3, 7.6)	53.4, CH	5.18, m*	53.6, CH	5.06, dd (12.9, 6.2)
30	172.3, C	-	172.4, C	-	172.5, C	-
31	27.6, CH_2_	2.63, dt (13.7, 7.0) 2.69 ~ 2.77, m*	27.5, CH_2_	2.62, dt (13.8, 6.9) 2.71, tt (13.8, 6.9)	27.3, CH_2_	2.51, m 2.68 ~ 2.74, m*
32	32.8, CH_2_	2.69 ~ 2.77, m*	32.1, CH_2_	2.80 ~ 2.88, m*	32.7, CH_2_	2.68 ~ 2.74, m*
33	175.7, C	-	176.1, C	-	176.7, C	-
34-NH_2_	-	8.34, s	-	-	-	8.32, br s

*overlapping signals: assignments were supported by HSQC and HMBC, ^a 1^H 500 MH_Z_, ^13^C 125 MHz; ^b 1^H 700 MH_Z_, ^13^C 175 MH_Z._

Compound **8** was obtained as a white amorphous solid. The molecular formula was established as C_36_H_60_N_4_O_7_ on the basis of the pseudo-molecular ion peaks [M+H]^+^ at *m/z* 661.4542 and [M+Na]^+^ at *m/z* 683.4354 observed in the HR-ESI(+)MS, indicating 9 double bond equivalents. The molecular formula of **8** (C_36_H_60_N_4_O_7_) compared to that of **5** (C_36_H_58_N_4_O_7_) showed an increase of 2 Da suggesting that a reduction occurred in compound **5** to afford compound **8**. This assumption was confirmed on the ^1^H NMR spectrum of **8** where the signals in the downfield region corresponding to H_a_-22´ (δ_H_ 5.60) and H_b_-22´ (δ_H_ 6.24) as observed in **5** were missing, but instead the signal in the upfield region corresponding to a methyl group H_3_-22´ at δ_H_ 1.65 was recorded (Table 3). Moreover, an additional signal observed on the ^1^H NMR of **8** attributable to the methine H-22 (δ_H_ 4.89) further confirmed this assumption, indicating that the reduction of **5** occurred on the ∆^22-22´^ double bond to afford **8**. The reduction of the double bond ∆^22-22´^ further justified the upfield shift of the nitrogen-bearing proton 21-NH, which resonated at *δ*_H_ 8.15 in compound 8 instead of *δ*_H_ 10.43 as in compound 5. In the HMBC spectrum, the correlations observed between H-22´ and C-22/C-23, H-22 and C-22´/C-23 confirmed the presence of an alanine residue instead of dehydroalanine residue as previously reported for **5** (Shiono et al. 2007). Finally, the unambiguous assignment of all proton and carbon signals in metabolite **8** was achieved based on ^1^H-^13^C HSQC and ^1^H-^13^C HMBC experiments, thus identifying compound **8** as a new derivative of fusaristatin A, for which the trivial name fusaristatin H was assigned.

Compounds **9**–**13** were respectively identified as phomoxanthones A (**9**) and B (**10**) (Isaka et al. 2001), dicerandrol B (**11**) (Wagenaar and Clardy 2001), phomochromenone C (**12**) (Ding et al. 2017; Wei et al. 2021), and diaporchromanone C (**13**) (Wei et al. 2021) by comparison of their HR-ESIMS and 1D and 2 D NMR spectroscopic data (Suppl. material 1: Figs S65–S99) with those reported in the literature.

### ﻿Physico-chemical characteristic of compounds 4, 7 and 8

Phomopchalasin N (**4**): Yellowish oil. [α]^20^_D_ -17.6 (c 0.278, MeOH), UV (MeOH, c = 0.013 mg/mL) λ_max_ (log ε) 202 (4.32) nm. CD (c = 2.83 × 10^-3^ M, MeOH) λ_max_ (Δε) 200 (-7.66) nm. HR-ESIMS*m/z* 458.2665 [M + Na]^+^, *m/z* 893.5440 [2M + Na]^+^, *m/z* 871.5621 [2M + H]^+^, *m/z* 418.2746 [M + H - H_2_O]^+^, *m/z* 436.2852 [M + H]^+^ (Calcd for C_28_H_38_NO_3_^+^ 436.2846), *t*_R_ = 10.47 min. For NMR data (^1^H: 500 MHz, ^13^C: 125 MHz, DMSO-*d_6_*), see Table 2.

Fusaristatin G (**7**): colorless oil. [α]^20^_D_ -8 (*c* 0.1, MeOH), UV (MeOH, c = 0.02 mg/mL) λ_max_ (log ε) 201 (4.21), 283 (3.96) nm. HR-ESIMS*m/z* 682.4024 [M + Na]^+^, *m/z* 1341.8157 [2M + Na]^+^, *m/z* 1319.8354 [2M + H]^+^, *m/z* 642.4102 [M + H - H_2_O]^+^, *m/z* 660.4219 [M + H]^+^ (Calcd for C_36_H_58_N_3_O_8_^+^ 660.4218), *t*_R_ = 14.80 min. For NMR data (^1^H: 700 MHz, ^13^C: 175 MHz, C_5_H_5_N-*d_5_*), see Table 3.

Fusaristatin H (**8**): White amorphous solid. [α]^20^_D_ +14 (*c* 0.03, MeOH), UV (MeOH, c = 0.02 mg/mL) λ_max_ (log ε) 201 (4.24), 283 (4.20) nm. HR-ESIMS*m/z* 683.4354 [M + Na]^+^, *m/z* 1343.8820 [2M + Na]^+^, *m/z* 1321.9000 [2M + H]^+^, *m/z* 661.4542 [M + H]^+^ (Calcd for C_36_H_61_N_4_O_7_^+^ 661.4535), *t*_R_ = 14.46 min. For NMR data (^1^H: 700 MHz, ^13^C: 175 MHz, C_5_H_5_N-*d_5_*), see Table 3.

### ﻿Biological activity

The extracts obtained from the fungal culture in ZM ½ exhibited activities against *Bacillussubtilis* with MIC values of 75 µg/mL for the supernatant´s extract and 2.3 µg/mL for the mycelial extract. These extracts were also active against *Mucorplumbeus* with respective MIC values of 150 and 37.5 µg/mL. Moreover, the purified compounds **1**–**7**, **9**, **10**, **12**, and **13** were subjected to antimicrobial assays against a panel of bacteria and fungi. The minimum inhibitory concentration (MIC) values showed that all compounds were active against at least one of the tested micro-organisms at concentration of 66.7 μg/mL (Table 4). Overall, the majority of the tested compounds exhibited weak to moderate activity. However, significant activity was noted for phomoxanthones A (**9**) and B (**10**) against *Bacillussubtilis*. Both compounds inhibited the growth of the latter bacterium with a MIC value of 1.7 μg/mL, which turned out to be 5 times stronger than that of oxytetracyclin used as positive control. In addition, their MIC value of 4.2 μg/mL against the Gram-positive bacterium *S.aureus* was quite considerable in comparison to that of the other tested compounds. This finding concurs well with previously published data which reported the antimicrobial activity of xanthone derivatives isolated from *Diaporthe* spp. (Wagenaar and Clardy 2001; Elsässer et al. 2005; Lim et al. 2010). The antimicrobial activity of dicerandrol B (**11**), a closely related congener of phomoxanthones A (**9**) and B (**10**) was not investigated in the present work due to the low amount of available sample, however, its activity against *B.subtilis* and *S.aureus* has previously been reported (Wagenaar and Clardy 2001). The antimicrobial activity of compound 8 was not assessed due to the paucity of the sample.

**Table 4.** Minimum Inhibitory Concentrations (MIC) of compounds **1**–**7**, **9**–**10**, **12**–**13** against tested microorganisms.

MIC (μg/mL)												
Test organisms	1	2	3	4	5	6	7	9	10	12	13	References
*Acinetobacterbaumannii*	-	-	-	-	-	-	-	-	-	-	-	0.26^c^
*Bacillussubtilis*	-	-	16.7	66.7	16.7		16.7	1.7	1.7	66.7		8.3°
*Candidaalbicans*	-	-	-	-	-	-		66.7	-	-	-	16.6^n^
*Chromobacteriumviolaceum*	-	-	-	-	-	-	-	-	-	-	-	0.83°
*Escherichiacoli*	-	-	-	-	-	-	-	-	-	-		1.7°
*Mucorhiemalis*	66.7	-	66.7	66.7	66.7	66.7	66.7	16.7	66.7	66.7	66.7	8.3^n^
*Mycobacteriumsmegmatis*	-	-	-	-	-	-	-	66.7	-	-	-	1.7^k^
*Pichiaanomala*	-	-	-	-	-	-	-	-	-	-	-	8.3^n^
*Pseudomonasaeruginosa*	-	-	-	-	-	-	-	-	-	-	-	0.21^g^
*Rhodotorulaglutinis*	66.7	-	-	-	-	-	-	-	-	-	-	4.2^n^
*Schizosaccharomycespombe*	16.7	66.7	66.7	66.7	-	-	-	-	66.7	-	-	8.3^n^
*Staphylococcusaureus*	-	-	66.7	66.7	66.7		66.7	4.2	4.2	66.7	-	0.83°

(-): No inhibition, ^c^Ciprobay 2.54 mg/mL, ^g^Gentamycin 1 mg/mL, ^k^Kanamycin 1 mg/mL, ^n^Nystatin 1 mg/mL, °Oxytetracyclin 1 mg/mL. Starting concentration for antimicrobial assay were 66.7 μg/mL.

The cytotoxicity of all the isolated compounds except **11** was evaluated against a panel of mammalian cell lines. Eight compounds, **1**–**5** and **8**–**10** showed activity in this assay whereas the other isolated metabolites were inactive under test conditions (Table 5). The very significant activity exhibited by compounds **1**–**4** against all tested cancer cell lines were in agreement with previous studies which have reported cytochalasins as potent cytotoxins (Shang et al. 2017). However, when comparing the activity of the cytochalasin **4**, which is the deacetylated derivative of **3**, it was quite interesting to notice that **4** is significantly less toxic than **3** leading to the hypothesis that the presence of the acetyl group in **3** is an important structural element in the biological activity of the studied cytochalasins. The aforementioned assumption, was also observed when comparing the cytotoxicity of compound **1** and **2**. In effect, **2** is the deacetylated derivative of **1**, and the latter was also found to be less toxic than **1**. These results therefore give some hints in regards to the structure activity relationship (SAR) of the isolated cytochalasins, which will be tested further for their inhibitory effect on actin. In the same assay, compound **5** and **8** were found to be active against KB3.1 cell line with IC_50_ value of 10.63 and 30.3 µM respectively whereas compound **6** and **7** bearing the same core skeleton did not show any activity. These results indicated that the cytotoxicity of this class of compounds might possibly be enhanced by the presence of an amide group (C-33) as shown in **5** and **8** instead of a carboxylic acid as observed in **6** (C-34) and 7 (C-33). In addition, phomoxanthones A (**9**) and B (**10**), exhibited strong cytotoxic activities with half-maximal inhibitory concentrations (IC_50_) in the range 0.02 – 9.7 µM. These results were in accordance with previous published cytotoxicity of dimeric tetrahydroxanthone derivatives against human epidermoid carcinoma (KB), human breast cancer (BC-1), mouse lymphoma (L5178Y), human ovarian carcinoma (A2780), and African monkey kidney fibroblast (Vero) cell lines among others (Isaka et al. 2001; Rönsberg et al. 2013).

**Table 5.** Cytotoxic activity of compounds **1**–**10**, **12**–**13**.

IC_50_ (µM)													
Cell lines	1	2	3	4	5	6	7	8	9	10	12	13	Epothilone B
KB3.1	0.064	0.33	1.7	5.8	10.6	-	-	30.3	0.36	0.91	-	-	6.5×10^-5^
L929	0.19	1.5	1.3	10.8	>30.4	-	-	-	1.06	5.6	-	-	6.5×10^-4^
A431	0.085	0.33	14.3	11.0	12.0	n.t	n.t	n.t	0.04	0.17	n.t	n.t	1.2×10^-4^
MCF-7	0.14	3.1	7.3	19.3	7.44	n.t	n.t	n.t	0.02	0.36	n.t	n.t	8.2×10^-5^
A549	0.16	0.73	3.1	10.3	19.7	n.t	n.t	n.t	0.43	1.0	n.t	n.t	6.1×10^-5^
SKOV-3	0.073	0.33	13.6	45.9	13.9	n.t	n.t	n.t	0.15	0.65	n.t	n.t	2.9×10^-4^
PC-3	0.14	0.29	4.2	9.4	7.3	n.t	n.t	n.t	1.1	9.7	n.t	n.t	9.5×10^-4^

n.t: not tested, (-): no activity. Starting concentration for cytotoxicity assay was 37 µg/mL

## ﻿Conclusion

The genus *Diaporthe* has been regarded for decades as a potential source for the production of diverse bioactive secondary metabolites. In the present study, we suggest the introduction of the new species *D.breyniae* isolated from the twigs of *Breyniaoblongifolia* in Cameroon. From the liquid culture of this fungus, two previously undescribed polyketides were isolated together with eleven known compounds. The isolated compounds showed weak to strong antimicrobial activities as well as moderate cytotoxic activities overall. These results demonstrated that it should certainly be worthwhile to explore untapped geographic area like the African tropics in general and Cameroon in particular for the discovery of new fungi and the isolation of novel secondary metabolites produced by these with significant biological activities.

## Supplementary Material

XML Treatment for Diaporthe breyniae

## Supplementary materials

Supplementary material 1 Figures S1–S100, Tables S1–S5 Data type Docx file. Explanation note The following are available online: 1D, 2D NMR, ESIMS and HR-ESIMS spectra of compounds **1**–**13**; Fig. S100, ML phylogram including our strain and type and reference strains of *Diaporthe* spp.; Table S1–S4, Information of the phylogenetic study; Alignment of the ITS, *cal*, *his3*, *tef1*, *tub2* sequences used in the second phylogenetic study. https://binary.pensoft.net/file/696069This dataset is made available under the Open Database License (http://opendatacommons.org/licenses/odbl/1.0/). The Open Database License (ODbL) is a license agreement intended to allow users to freely share, modify, and use this Dataset while maintaining this same freedom for others, provided that the original source and author(s) are credited.Blondelle Matio Kemkuignou, Lena Schweizer, Christopher Lambert, Elodie Gisèle M. Anoumedem, Simeon F. Kouam, Marc Stadler, Yasmina Marin-Felix
