# Supplementary material for: ﻿New polyketides from the liquid culture of Diaporthebreyniae sp. nov. (Diaporthales, Diaporthaceae)
Source: MycoKeys. 2022 Jun 14;90:85–118. doi: 10.3897/mycokeys.90.82871 (PMC9849082; doi:10.3897/mycokeys.90.82871)
Supplement: Supplementary material 1 — Figures S1–S100, Tables S1–S5 [file mycokeys-90-085-s001.docx]

**New polyketides from the liquid culture of *Diaporthe* *breyniae* sp. nov.**

Blondelle Matio Kemkuignou^1,2^, Lena Schweizer^1^, Christopher Lambert^1,2^, Elodie Gisèle M. Anoumedem^3^, Simeon F. Kouam^3^, Marc Stadler^1,2^, Yasmina Marin-Felix^1,2^

^1^Department of Microbial Drugs, Helmholtz Centre for Infection Research (HZI) and German Centre for Infection Research (DZIF), Partner Site Hannover/Braunschweig, Inhoffenstrasse 7, 38124 Braunschweig, Germany.

^2^Institute of Microbiology, Technische Universität Braunschweig, Spielmannstraße 7, 38106 Braunschweig, Germany.

^3^Department of Chemistry, Higher Teacher Training College, University of Yaoundé I, Yaoundé P.O. Box 47, Cameroon

Corresponding author: Yasmina Marin Felix (Yasmina.marinfelix@helmholtz-hzi.de)

Contents

[Figure S1: HPLC-UV chromatogram of the small-scale crude extracts of *Diaporthe breyniae* in YM agar (a), YM 6.3 (b, c), Q6 ½ (d, e) and solid rice (f) (detection at 210 nm). 6](#_Toc97744600)

[Figure S2: HPLC-UV chromatogram of the crude extracts of *Diaporthe* *breyniae* sp. in ZM ½ medium (detection at 210 nm) 7](#_Toc97744601)

[Figure S3: HR-ESI (+) MS data for cytochalasin H (1) 8](#_Toc97744603)

[Figure S4: ESIMS data for cytochalasin H (1) 9](#_Toc97744604)

**Figure S5: ^1^H NMR spectrum (DMSO-*d_6_*, 500 MHz) of cytochalasin H (1)…………………………………………….10**

[Figure S6: ^13^C NMR spectrum (DMSO-*d_6_*, 125MHz) of cytochalasin H (1) 11](#_Toc97744605)

[Figure S7: ^1^H-^1^H COSY NMR spectrum (DMSO-*d_6_*, 500 MHz) of cytochalasin H (1) 12](#_Toc97744606)

**Figure S8: ^1^H-^13^C HSQC NMR spectrum (DMSO-*d_6_*, 500 MHz) of cytochalasin H (1)……………………………..13**

[Figure S9: ^1^H-^13^C HMBC NMR spectrum (DMSO-*d_6_*, 500 MHz) of cytochalasin H (1) 14](#_Toc97744607)

[Figure S10: ^1^H-^1^H NOESY NMR spectrum (DMSO-*d_6_*, 500 MHz) of cytochalasin H (1) 15](#_Toc97744608)

[Figure S11: HR-ESI (+) MS data for cytochalasin J (2) 16](#_Toc97744609)

[Figure S12: ESIMS data for cytochalasin J (2) 17](#_Toc97744610)

[Figure S13: ^1^H NMR spectrum (DMSO-*d6*, 500 MHz) of cytochalasin J (2) 18](#_Toc97744611)

[Figure S14: ^1^H-^1^H COSY NMR spectrum (DMSO-*d6*, 500 MHz) of cytochalasin J (2) 19](#_Toc97744612)

[Figure S15: ^1^H-^13^C HSQC NMR spectrum (DMSO-*d6*, 500 MHz) of cytochalasin J (2) 20](#_Toc97744613)

[Figure S16: ^1^H-^13^C HMBC NMR spectrum (DMSO-*d6*, 500 MHz) of cytochalasin J (2) 21](#_Toc97744614)

[Figure S17: ^1^H-^1^H NOESY NMR spectrum (DMSO-*d6*, 500 MHz) of cytochalasin J (2) 22](#_Toc97744615)

[Figure S18: HR-ESI (+) MS data for cytochalasin RKS-1778 (3) 23](#_Toc97744616)

[Figure S19: ESIMS data for cytochalasin RKS-1778 (3) 24](#_Toc97744617)

[Figure S20: ^1^H NMR spectrum (DMSO-*d6*, 500 MHz) of cytochalasin RKS-1778 (3) 25](#_Toc97744618)

[Figure S21: ^1^H-^1^H COSY NMR spectrum (DMSO-*d6*, 500 MHz) of cytochalasin RKS-1778 (3) 26](#_Toc97744619)

[Figure S22: ^1^H-^13^C HSQC NMR spectrum (DMSO-*d6*, 500 MHz) of cytochalasin RKS-1778 (3) 27](#_Toc97744620)

[Figure S23: ^1^H-^13^C HMBC NMR spectrum (DMSO-*d6*, 500 MHz) of cytochalasin RKS-1778 (3) 28](#_Toc97744621)

[Figure S24: ^1^H-^1^H NOESY NMR spectrum (DMSO-*d6*, 500 MHz) of cytochalasin RKS-1778 (3) 29](#_Toc97744622)

[Figure S25: HR-ESI (+) MS data for phomopchalasin N (4) 30](#_Toc97744623)

**Figure S26: ESIMS data for phomopchalasin N (4)………………………………………………………………………........31**

[Figure S27: ^1^H NMR spectrum (DMSO-*d6*, 500 MHz) of phomopchalasin N (4) 32](#_Toc97744624)

[Figure S28: ^13^C NMR spectrum (DMSO-*d6*, 125 MHz) of phomopchalasin N (4) 33](#_Toc97744625)

[Figure S29: ^1^H-^1^H COSY NMR spectrum (DMSO-*d6*, 500 MHz) of phomopchalasin N (4) 34](#_Toc97744626)

[Figure S30: ^1^H-^13^C HSQC NMR spectrum (DMSO-*d6*, 500 MHz) of phomopchalasin N (4) 35](#_Toc97744627)

[Figure S31: ^1^H-^13^C HMBC NMR spectrum (DMSO-*d6*, 500 MHz) of phomopchalasin N (4) 36](#_Toc97744628)

[Figure S32: ^1^H-^1^H NOESY NMR spectrum (DMSO-*d6*, 500 MHz) of phomopchalasin N (4) 37](#_Toc97744629)

**Figure S33: UV/vis spectrum of phomopchalasin N (4) in MeOH………………………………………………………..38**

[Figure S34: HR-ESI (+) MS data for fusaristatin A (5) 39](#_Toc97744630)

[Figure S35: ESIMS data for fusaristatin A (5) 40](#_Toc97744631)

[Figure S36: ^1^H NMR spectrum (Pyridin-*d_5_*, 500 MHz) of fusaristatin A (5) 41](#_Toc97744632)

[Figure S37: ^13^C NMR spectrum (Pyridin-*d_5_*, 125 MHz) of fusaristatin A (5) 42](#_Toc97744633)

[Figure S38: ^1^H-^1^H COSY NMR spectrum (Pyridin-*d_5_*, 500 MHz) of fusaristatin A (5) 43](#_Toc97744634)

[Figure S39: ^1^H-^13^C HSQC NMR spectrum (Pyridin-*d_5_*, 500 MHz) of fusaristatin A (5) 44](#_Toc97744635)

[Figure S40: ^1^H-^13^C HMBC NMR spectrum (Pyridin-*d_5_*, 500 MHz) of fusaristatin A (5) 45](#_Toc97744636)

[Figure S41: ^1^H-^1^H NOESY NMR spectrum (Pyridin-*d_5_*, 500 MHz) of fusaristatin A (5) 46](#_Toc97744637)

[Figure S42: HR-ESI (+) MS data for fusaristatin B (6) 47](#_Toc97744638)

[Figure S43: ESIMS data for fusaristatin B (6) 48](#_Toc97744639)

[Figure S44: ^1^H NMR spectrum (Pyridin-*d_5_*, 700 MHz) of fusaristatin B (6) 49](#_Toc97744640)

[Figure S45: ^1^H-^1^H COSY NMR spectrum (Pyridin-*d_5_*, 700 MHz) of fusaristatin B (6) 50](#_Toc97744641)

[Figure S46: ^1^H-^13^C HSQC NMR spectrum (Pyridin-*d_5_*, 700 MHz) of fusaristatin B (6) 51](#_Toc97744642)

[Figure S47: ^1^H-^13^C HMBC NMR spectrum (Pyridin-*d_5_*, 700 MHz) of fusaristatin B (6) 52](#_Toc97744643)

[Figure S48: HR-ESI (+) MS data for fusaristatin G (7) 53](#_Toc97744644)

[Figure S49: ESIMS data for fusaristatin G (7) 54](#_Toc97744645)

[Figure S50: ^1^H NMR spectrum (Pyridin-*d_5_*,700 MHz) of fusaristatin G (7) 55](#_Toc97744646)

[Figure S51: ^13^C NMR spectrum (Pyridin-*d_5_*, 175 MHz) of fusaristatin G (7) 56](#_Toc97744647)

[Figure S52: ^1^H-^1^H COSY NMR spectrum (Pyridin-*d*_5_, 700 MHz) of fusaristatin G (7) 57](#_Toc97744648)

[Figure S53: ^1^H-^13^C HSQC NMR spectrum (Pyridin-*d_5_*, 700 MHz) of fusaristatin G (7) 58](#_Toc97744649)

[Figure S54: ^1^H-^13^C HMBC NMR spectrum (Pyridin-*d_5_*, 700 MHz) of fusaristatin G (7) 59](#_Toc97744650)

[Figure S55: ^1^H-^1^H NOESY NMR spectrum (Pyridin-*d_5_*, 700 MHz) of fusaristatin G (7) 60](#_Toc97744651)

[Figure S56: UV/vis spectrum of fusaristatin G (7) in MeOH 61](#_Toc97744652)

[Figure S57: HR-ESI (+) MS data for fusaristatin H (8) 62](#_Toc97744653)

[Figure S58: ESIMS data for fusaristatin H (8) 63](#_Toc97744654)

[Figure S59: ^1^H NMR spectrum (Pyridin-*d_5_*, 700 MHz) of fusaristatin H (8) 64](#_Toc97744655)

[Figure S60: ^1^H-^1^H COSY NMR spectrum (Pyridin-*d_5_*, 700 MHz) of fusaristatin H (8) 65](#_Toc97744656)

[Figure S61: ^1^H-^13^C HSQC NMR spectrum (Pyridin-*d_5_*, 700 MHz) of fusaristatin H (8) 66](#_Toc97744657)

[Figure S62: ^1^H-^13^C HMBC NMR spectrum (Pyridin-*d_5_*, 700 MHz) of fusaristatin H (8) 67](#_Toc97744658)

[Figure S63: ^1^H-^1^H NOESY NMR spectrum (Pyridin-*d_5_*, 700 MHz) of fusaristatin H (8) 68](#_Toc97744659)

[Figure S64: UV/vis spectrum of fusaristatin H (8)in MeOH 69](#_Toc97744660)

[Figure S65: HR-ESI (+) MS data for phomoxanthone A (9) 70](#_Toc97744661)

[Figure S66: ESIMS data for phomoxanthone A (9) 71](#_Toc97744662)

[Figure S67: ^1^H NMR spectrum (chloroform-*d*, 500 MHz) of phomoxanthone A (9) 72](#_Toc97744663)

[Figure S68: ^13^C NMR spectrum (chloroform-*d*, 125 MHz) of phomoxanthone A (9) 73](#_Toc97744664)

[Figure S69: ^1^H-^1^H COSY NMR spectrum (chloroform-*d*, 500 MHz) of phomoxanthone A (9) 74](#_Toc97744665)

[Figure S70: ^1^H-^13^C HSQC NMR spectrum (chloroform-*d*, 500 MHz) of phomoxanthone A (9) 75](#_Toc97744666)

[Figure S71: ^1^H-^13^C HMBC NMR spectrum (chloroform-*d*, 500 MHz) of phomoxanthone A (9) 76](#_Toc97744667)

[Figure S72: ^1^H-^1^H NOESY NMR spectrum (chloroform-*d*, 500 MHz) of phomoxanthone A (9) 77](#_Toc97744668)

[Figure S73: HR-ESI (+) MS data for phomoxanthone B (10) 78](#_Toc97744669)

[Figure 74: ESIMS data for phomoxanthone B (10) 79](#_Toc97744670)

[Figure S75: ^1^H NMR spectrum (chloroform-*d*, 700 MHz) of phomoxanthone B (10) 80](#_Toc97744671)

[Figure S76: ^1^H-^1^H COSY NMR spectrum (chloroform-*d*, 700 MHz) of phomoxanthone B (10) 81](#_Toc97744672)

[Figure S77: ^1^H-^13^C HSQC NMR spectrum (chloroform-*d*, 700 MHz) of phomoxanthone B (10) 82](#_Toc97744673)

[Figure S78: ^1^H-^13^C HMBC NMR spectrum (chloroform-*d*, 700 MHz) of phomoxanthone B (10) 83](#_Toc97744674)

[Figure S79: ^1^H-^1^H NOESY NMR spectrum (chloroform-*d*, 700 MHz) of phomoxanthone B (10) 84](#_Toc97744675)

[Figure S80: HR-ESI (+) MS data for dicerandrol B (11) 85](#_Toc97744676)

[Figure S81: ESIMS data for dicerandrol B (11) 86](#_Toc97744677)

[Figure S82: ^1^H NMR spectrum (chloroform-*d*, 700 MHz) of dicerandrol B (11) 87](#_Toc97744678)

[Figure S83: ^1^H-^1^H COSY NMR spectrum (chloroform-*d*, 700 MHz) of dicerandrol B (11) 88](#_Toc97744679)

[Figure S84: ^1^H-^13^C HSQC NMR spectrum (chloroform-*d*, 700 MHz) of dicerandrol B (11) 89](#_Toc97744680)

[Figure S85: ^1^H-^13^C HMBC NMR spectrum (chloroform-*d*, 700 MHz) of dicerandrol B (11) 90](#_Toc97744681)

[Figure S86: ^1^H-^1^H NOESY NMR spectrum (chloroform-*d*, 700 MHz) of dicerandrol B (11) 91](#_Toc97744682)

[Figure S87: HR-ESI (+) MS data for phomochromenone C (12) 92](#_Toc97744683)

[Figure S88: ESIMS data for phomochromenone C (12) 93](#_Toc97744684)

[Figure S89: ^1^H NMR spectrum (chloroform-*d*, 500 MHz) of phomochromenone C (12) 94](#_Toc97744685)

[Figure S90: ^1^H-^1^H COSY NMR spectrum (chloroform-*d*, 500 MHz) of phomochromenone C (12) 95](#_Toc97744686)

[Figure S91: ^1^H-^13^C HSQC NMR spectrum (chloroform-*d*, 500 MHz) of phomochromenone C (12) 96](#_Toc97744687)

[Figure S92: ^1^H-^13^C HMBC NMR spectrum (chloroform-*d*, 500 MHz) of phomochromenone C (12) 97](#_Toc97744688)

[Figure S93: ^1^H-^1^H NOESY NMR spectrum (chloroform-*d*, 500 MHz) of phomochromenone C (12) 98](#_Toc97744689)

[Figure S94: HR-ESI (+) MS data for diapochromanone C (13) 99](#_Toc97744690)

[Figure S95: ESIMS data for diapochromanone C (13) 100](#_Toc97744691)

[Figure S96: ^1^H NMR spectrum (DMSO-*d6*, 500 MHz) of diapochromanone C (13) 101](#_Toc97744692)

[Figure S97: ^1^H-^1^H COSY NMR spectrum (DMSO-*d6*, 500 MHz) of diapochromanone C (13) 102](#_Toc97744693)

[Figure S98: ^1^H-^13^C HSQC NMR spectrum (DMSO-*d6*, 500 MHz) of diapochromanone C (13) 103](#_Toc97744694)

[Figure S99: ^1^H-^13^C HMBC NMR spectrum (DMSO-*d6*, 500 MHz) of diapochromanone C (13) 104](#_Toc97744695)

[Table S1: Flanking positions of gblocks curated MAFFT alignments of the first phylogenetic analysis. Characteristics of the alignments subsequently used for multigene phylogenetic inference using IQTree 2. 105](#_Toc97744696)

[Table S2: Selected edge-linked proportional partition substitution models subjected to IQTree2 calculated with ModelFinder using Bayesian information criterion (BIC). 106](#_Toc97744697)

[Table S3: Characteristics of the restricted MAFFT alignments following the first phylogenetic analysis using IQTree 2 for phylogenetic inference. 106](#_Toc97744698)

[Table S4: Selected edge-linked proportional partition substitution models for the restricted phylogenetic analysis subjected to IQTree2 calculated with ModelFinder using Bayesian information criterion (BIC). 106](#_Toc97744699)

[Figure S100. ML (lLN=-50994.2709) phylogram obtained from the combined curated ITS, *cal*, *his3*, *tef1* and *tub2* sequences of our strain and type and reference strains of *Diaporthe* spp. *Diaporthella corylina* CBS 121124 was used as outgroup. Bootstrap support values ≥70/are indicated along branches. Branch lengths are proportional to distance. Alignment deposited in TreeBase (S29473). 108](#_Toc97744700)

[Table S5. Strains of *Diaporthe* spp. included in the first phylogenetic study. 112](#_Toc97744701)

[⮚ Alignment of the ITS sequences used in the second phylogenetic study 127](#_Toc97744702)

[⮚ Alignment of the *cal* sequences used in the second phylogenetic study 154](#_Toc97744703)

[⮚ Alignment of the *his3* sequences used in the second phylogenetic study 168](#_Toc97744704)

[⮚ Alignment of the *tef1* sequences used in the second phylogenetic study 177](#_Toc97744705)

[⮚ Alignment of the *tub2* sequences used in the second phylogenetic study 192](#_Toc97744706)

YM Agar, UV Chromatogram, 210 nm

YM 6.3 mycelium, UV Chromatogram, 210 nm

YM 6.3 supernatant, UV Chromatogram, 210 nm

Q6 ½ mycelium, UV Chromatogram, 210 nm

Q6 ½ supernatant, UV Chromatogram, 210 nm

Solid rice, UV Chromatogram, 210 nm

0

200

400

600

Intens.

[mAU]

0

100

200

300

Intens.

[mAU]

0

250

500

750

1000

Intens.

[mAU]

0

250

500

750

1000

1250

Intens.

[mAU]

0

100

200

300

Intens.

[mAU]

0

100

200

300

Intens.

[mAU]

2.5

5.0

7.5

10.0

12.5

15.0

17.5

20.0

22.5

Time [min]

**a**

**b**

**c**

**d**

**e**

**f**

# **Figure S1**: HPLC-UV chromatogram of the small-scale crude extracts of *Diaporthe breyniae* in YM agar (**a**), YM 6.3 (**b**, **c**), Q6 ½ (**d**, **e**) and solid rice (**f**) (detection at 210 nm).

Mycelium ZM ½ *D. breyniae* , UV Chromatogram, 210 nm

Supernatant ZM ½ *D. breyniae* , UV Chromatogram, 210 nm

0

200

400

600

800

Intens.

[mAU]

0

200

400

600

800

1000

Intens.

[mAU]

2

4

6

8

10

12

14

16

Time [min]

**8**

**9**

7

**1**

**2**

**5**

**12**

**13**

**4**

**3**

**11**

**10**

**6**

**2**

**1**

**9**

**13**

**4**

**3**

# **Figure S2**: HPLC-UV chromatogram of the crude extracts of *Diaporthe* *breyniae* sp. in ZM ½ medium (detection at 210 nm)


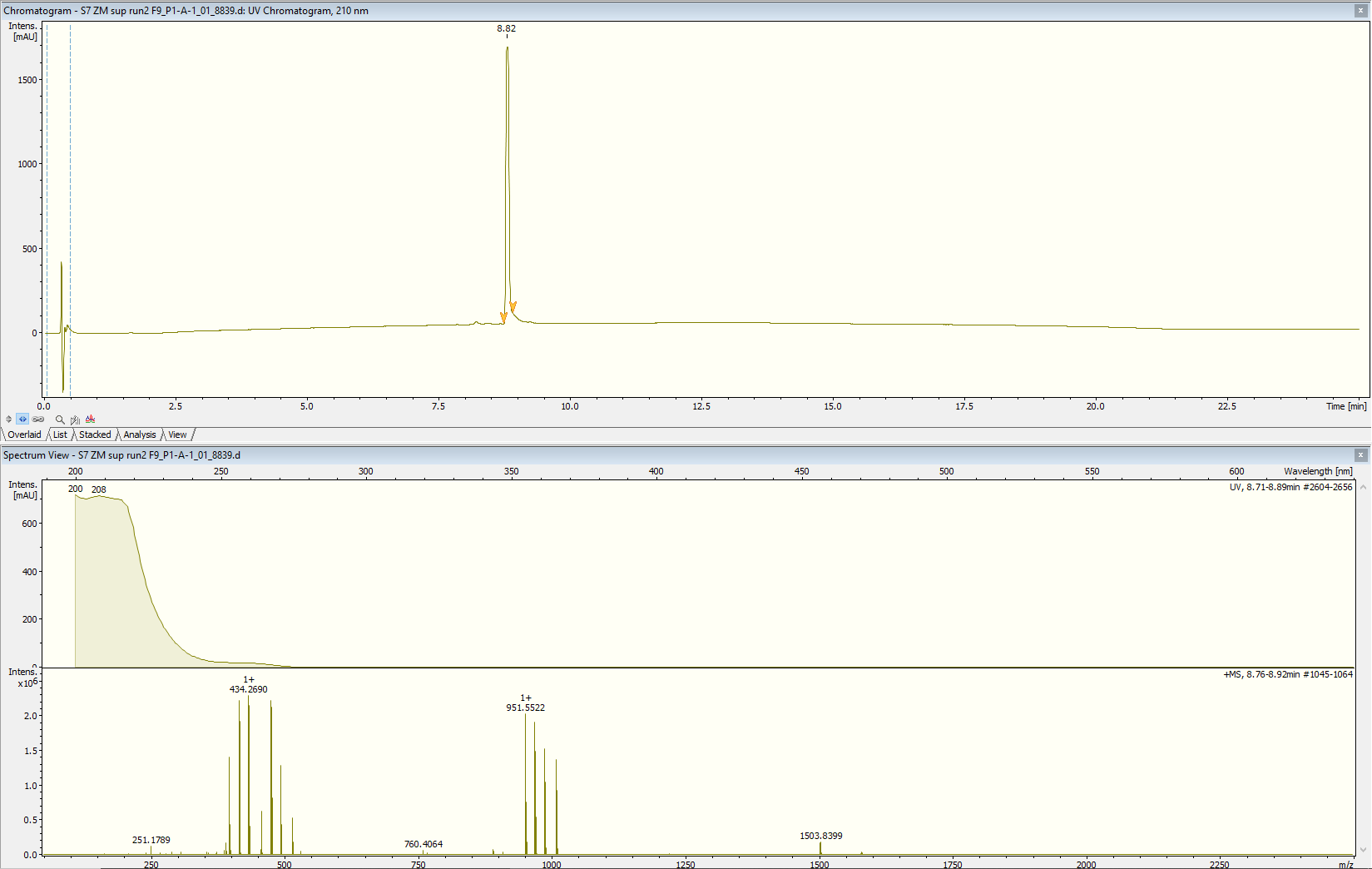


**Figure S3**: HR-ESI (+) MS data for cytochalasin H (**1**)


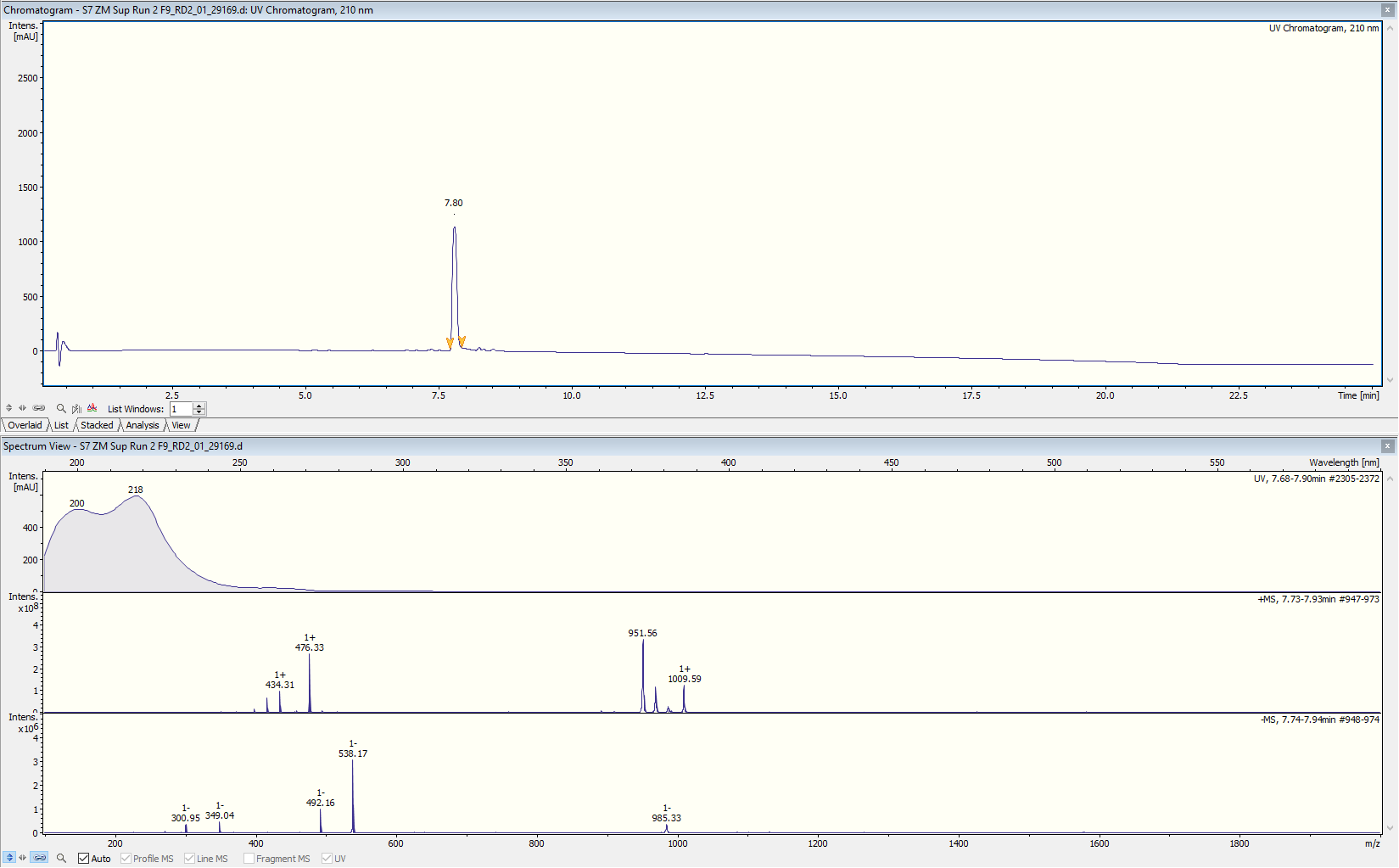


# **Figure S4**: ESIMS data for cytochalasin H (**1**)

**Figure S5**: ^1^H NMR spectrum (DMSO-*d_6_*, 500 MHz) of cytochalasin H (**1**)

# **Figure S6**: ^13^C NMR spectrum (DMSO-*d_6_*, 125MHz) of cytochalasin H (**1**)

# **Figure S7**: ^1^H-^1^H COSY NMR spectrum (DMSO-*d_6_*, 500 MHz) of cytochalasin H (**1**)

**Figure S8**: ^1^H-^13^C HSQC NMR spectrum (DMSO-*d_6_*, 500 MHz) of cytochalasin H (**1**)

# **Figure S9**: ^1^H-^13^C HMBC NMR spectrum (DMSO-*d_6_*, 500 MHz) of cytochalasin H (**1**)

# **Figure S10**: ^1^H-^1^H NOESY NMR spectrum (DMSO-*d_6_*, 500 MHz) of cytochalasin H (**1**)


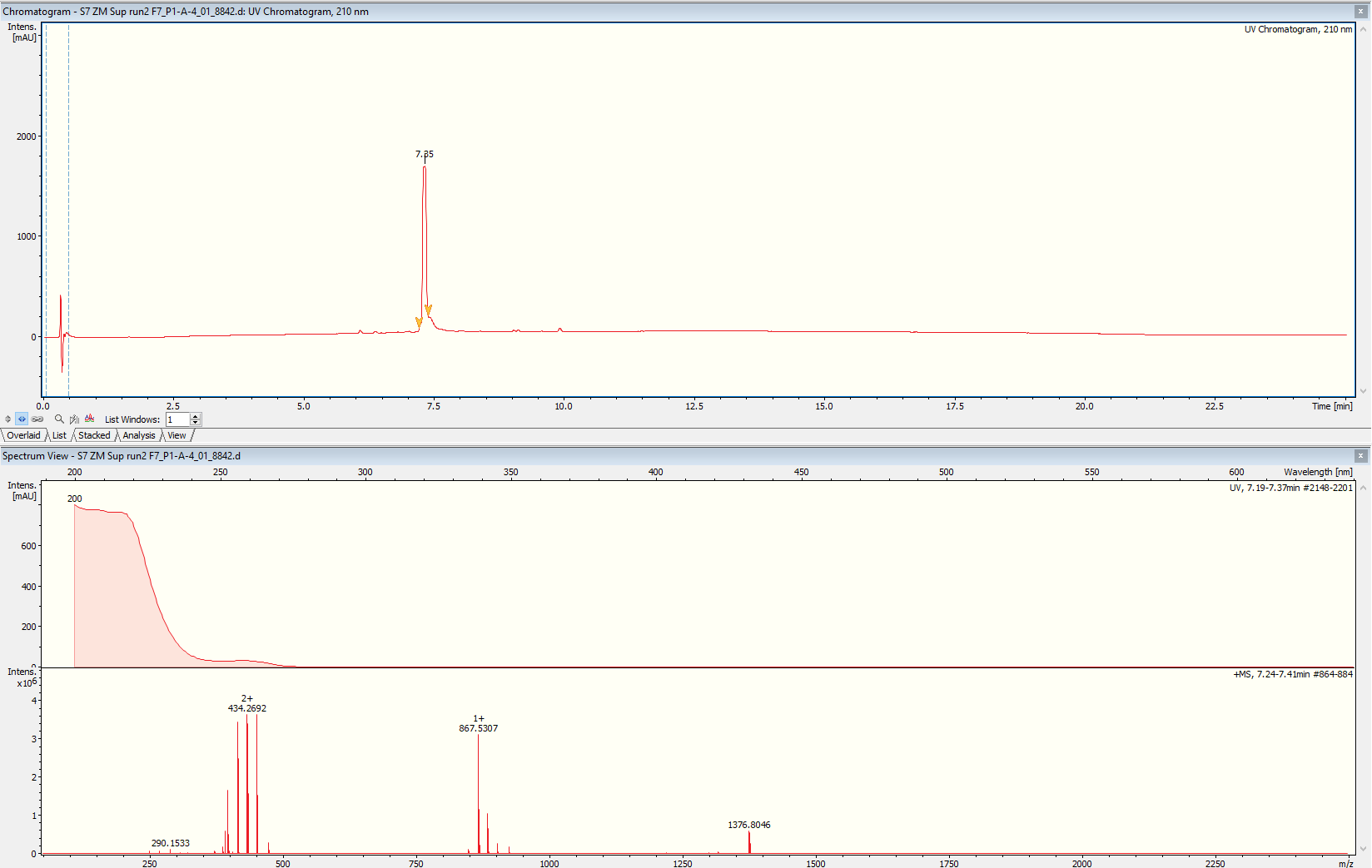


# **Figure S11**: HR-ESI (+) MS data for cytochalasin J (**2**)


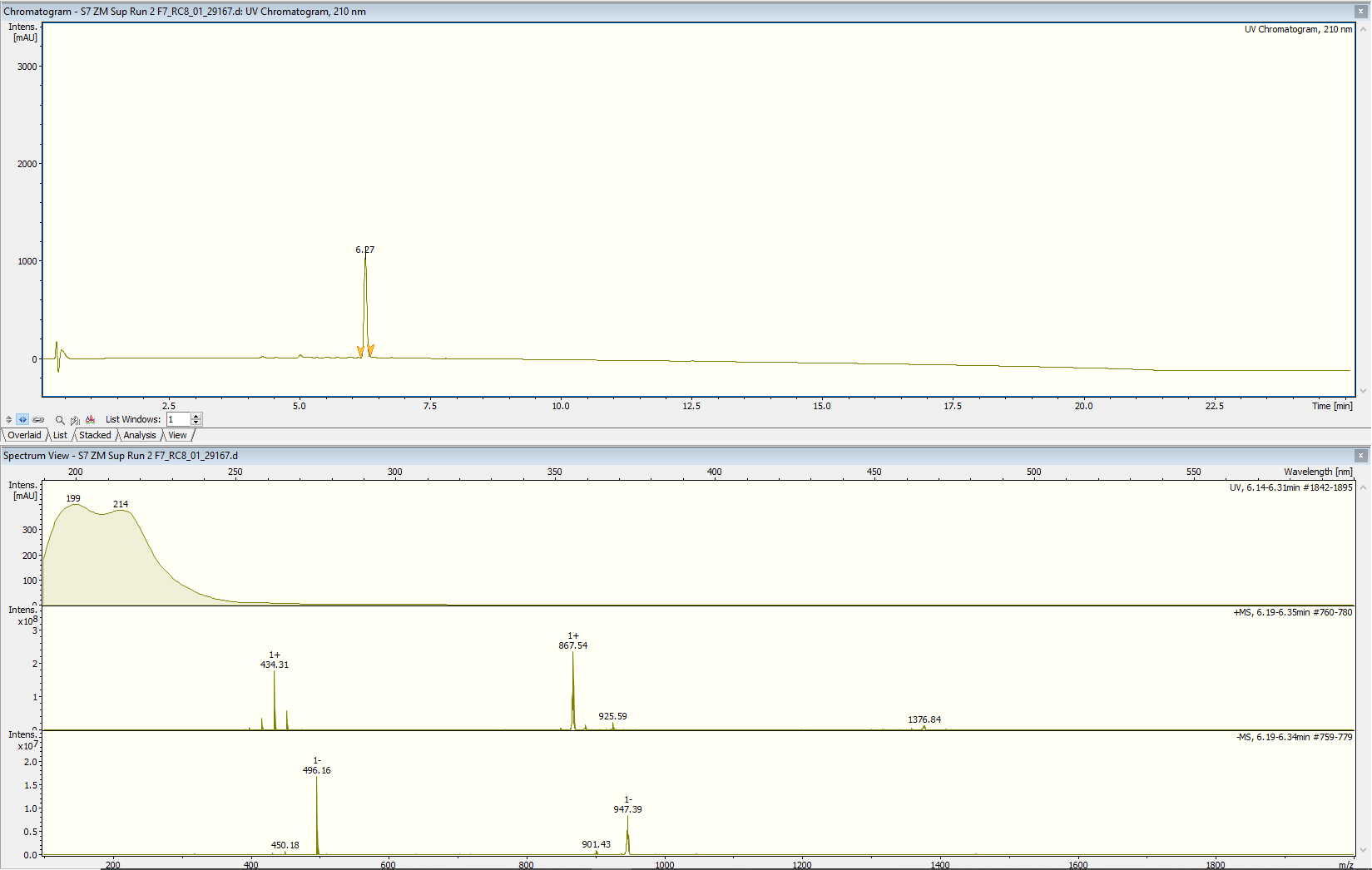


# **Figure S12**: ESIMS data for cytochalasin J (**2**)

# **Figure S13**: ^1^H NMR spectrum (DMSO-*d_6_*, 500 MHz) of cytochalasin J (**2**)

# **Figure S14**: ^1^H-^1^H COSY NMR spectrum (DMSO-*d_6_*, 500 MHz) of cytochalasin J (**2**)

# **Figure S15**: ^1^H-^13^C HSQC NMR spectrum (DMSO-*d_6_*, 500 MHz) of cytochalasin J (**2**)

# **Figure S16**: ^1^H-^13^C HMBC NMR spectrum (DMSO-*d_6_*, 500 MHz) of cytochalasin J (**2**)

# **Figure S17**: ^1^H-^1^H NOESY NMR spectrum (DMSO-*d_6_*, 500 MHz) of cytochalasin J (**2**)


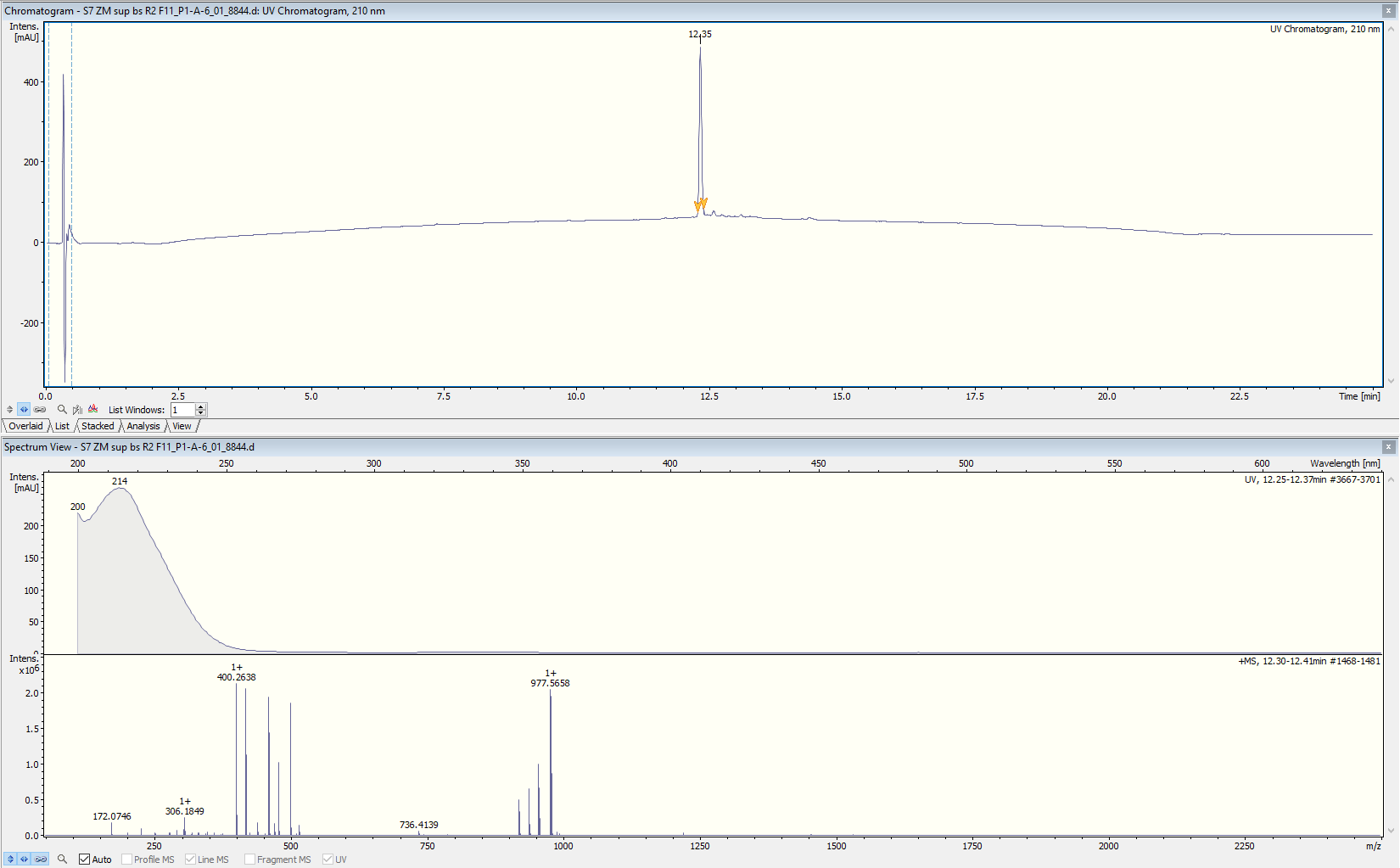


# **Figure S18**: HR-ESI (+) MS data for cytochalasin RKS-1778 (**3**)


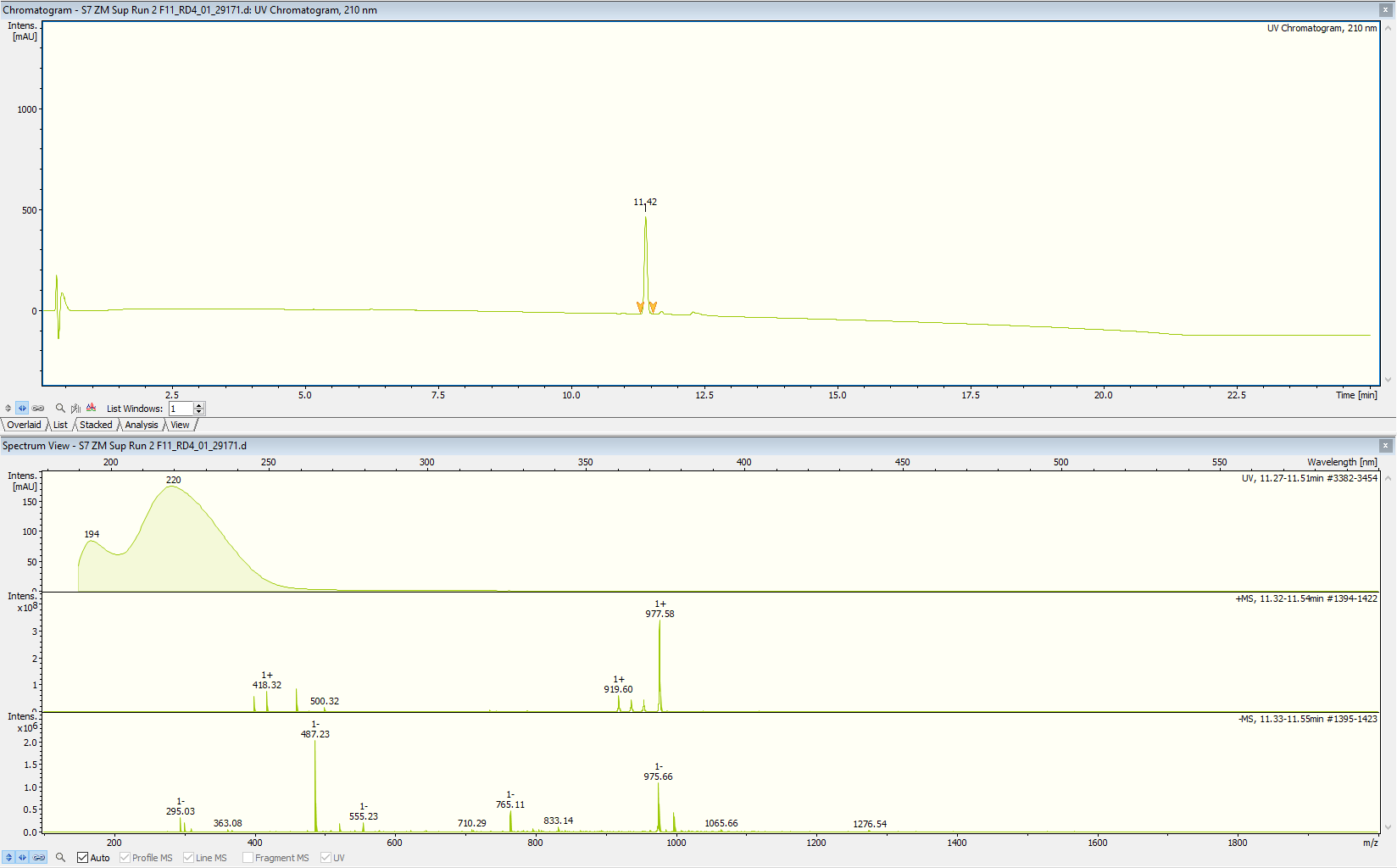


# **Figure S19**: ESIMS data for cytochalasin RKS-1778 (**3**)

# **Figure S20**: ^1^H NMR spectrum (DMSO-*d_6_*, 500 MHz) of cytochalasin RKS-1778 (**3**)

# **Figure S21**: ^1^H-^1^H COSY NMR spectrum (DMSO-*d_6_*, 500 MHz) of cytochalasin RKS-1778 (**3**)

# **Figure S22**: ^1^H-^13^C HSQC NMR spectrum (DMSO-*d_6_*, 500 MHz) of cytochalasin RKS-1778 (**3**)

# **Figure S23**: ^1^H-^13^C HMBC NMR spectrum (DMSO-*d_6_*, 500 MHz) of cytochalasin RKS-1778 (**3**)

# **Figure S24**: ^1^H-^1^H NOESY NMR spectrum (DMSO-*d_6_*, 500 MHz) of cytochalasin RKS-1778 (**3**)


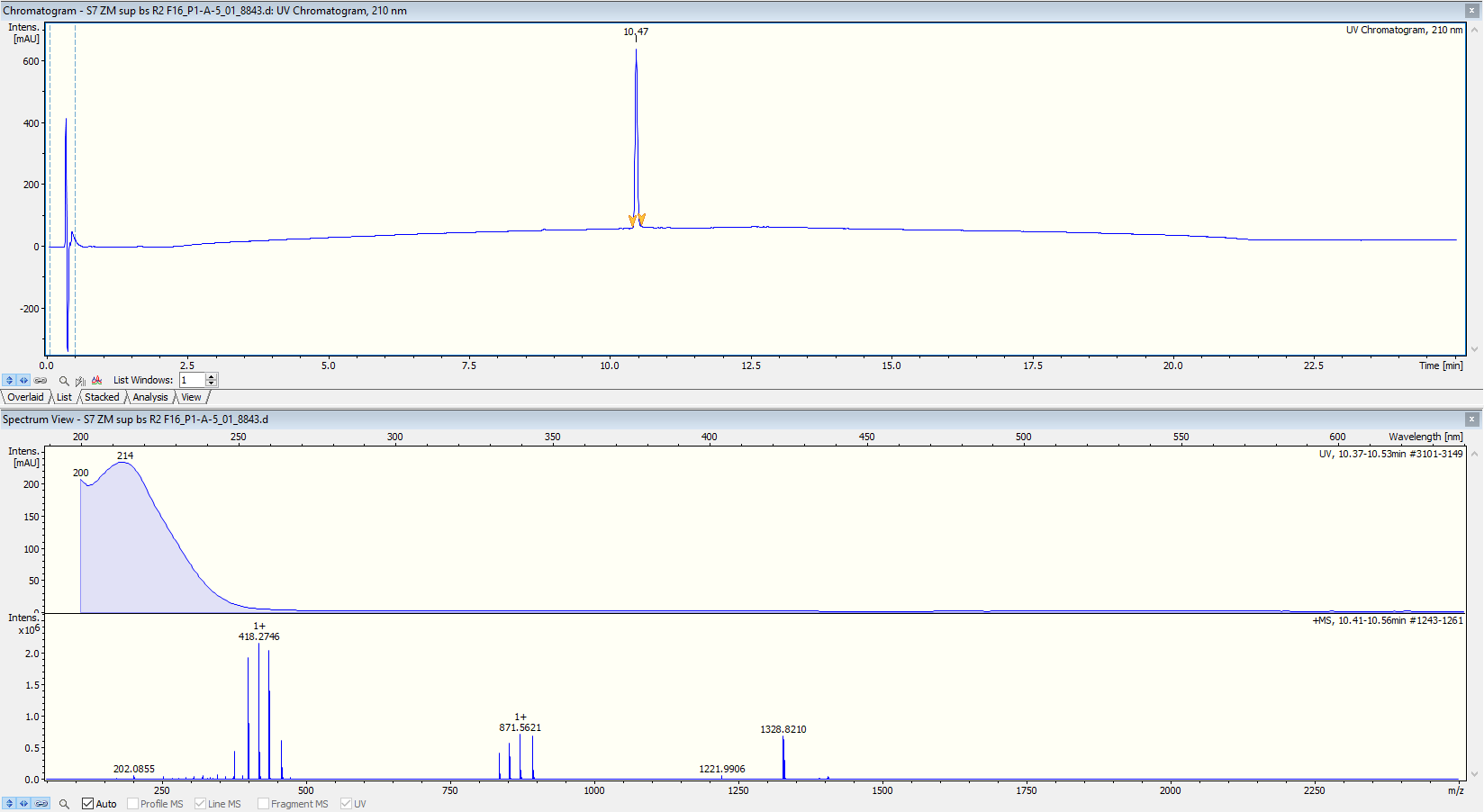


# **Figure S25**: HR-ESI (+) MS data for phomopchalasin N (**4**)


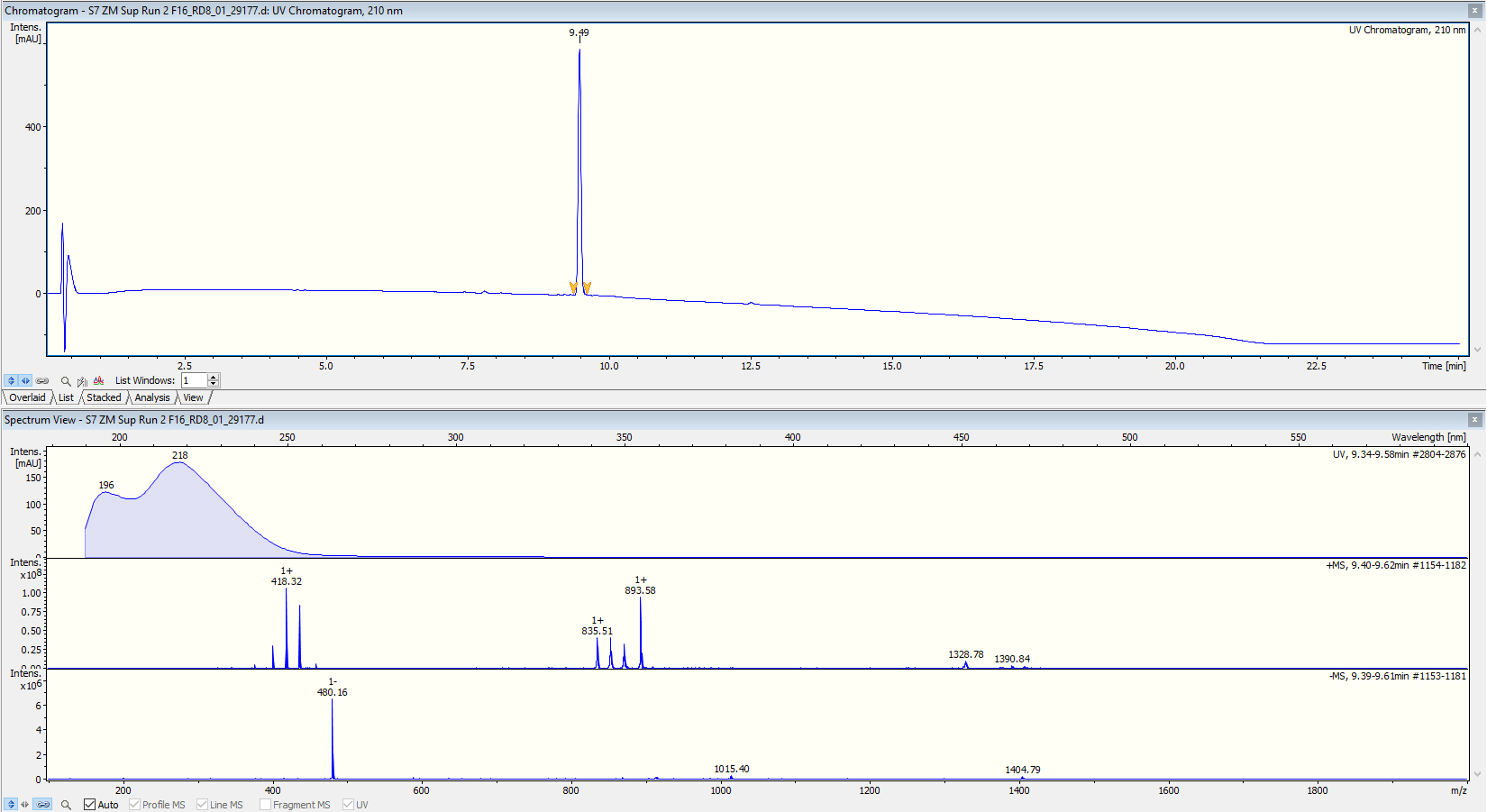


**Figure S26**: ESIMS data for phomopchalasin N (**4**)

# **Figure S27**: ^1^H NMR spectrum (DMSO-*d_6_*, 500 MHz) of phomopchalasin N (**4**)

# **Figure S28**: ^13^C NMR spectrum (DMSO-*d_6_*, 125 MHz) of phomopchalasin N (**4**)

# **Figure S29**: ^1^H-^1^H COSY NMR spectrum (DMSO-*d_6_*, 500 MHz) of phomopchalasin N (**4**)

# **Figure S30**: ^1^H-^13^C HSQC NMR spectrum (DMSO-*d_6_*, 500 MHz) of phomopchalasin N (**4**)

# **Figure S31**: ^1^H-^13^C HMBC NMR spectrum (DMSO-*d_6_*, 500 MHz) of phomopchalasin N (**4**)

# **Figure S32**: ^1^H-^1^H NOESY NMR spectrum (DMSO-*d_6_*, 500 MHz) of phomopchalasin N (**4**)


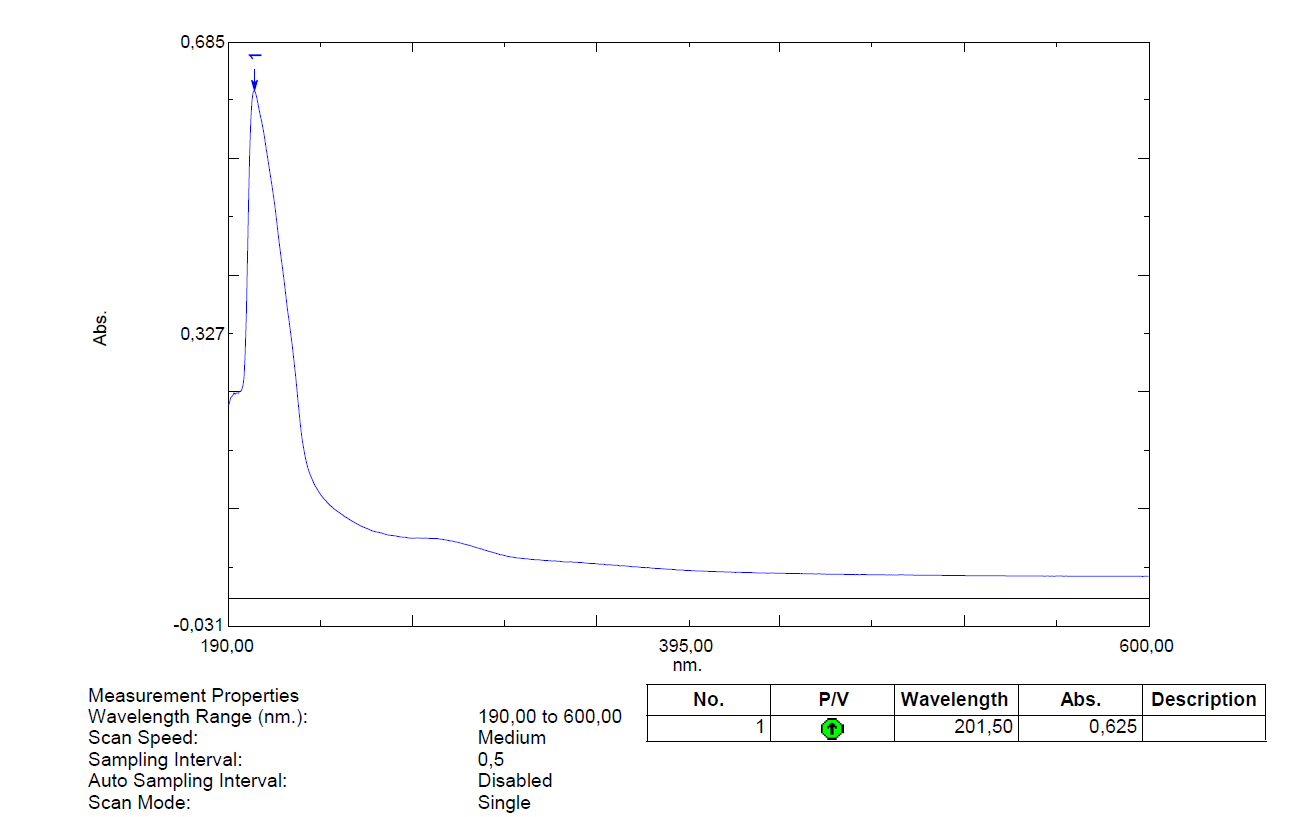


**Figure S33**: UV/vis spectrum of phomopchalasin N (**4**) in MeOH


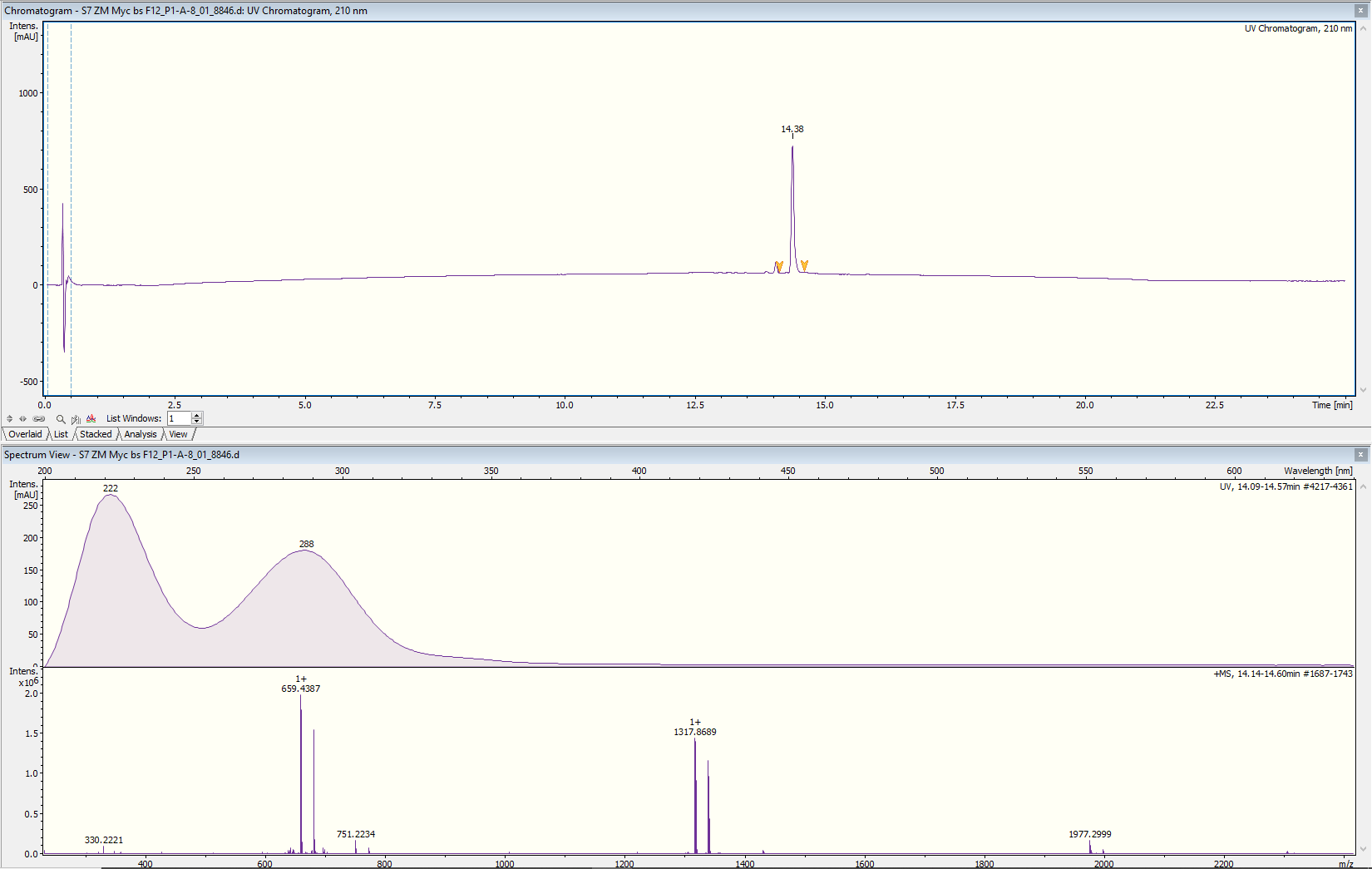


# **Figure S34**: HR-ESI (+) MS data for fusaristatin A (**5**)


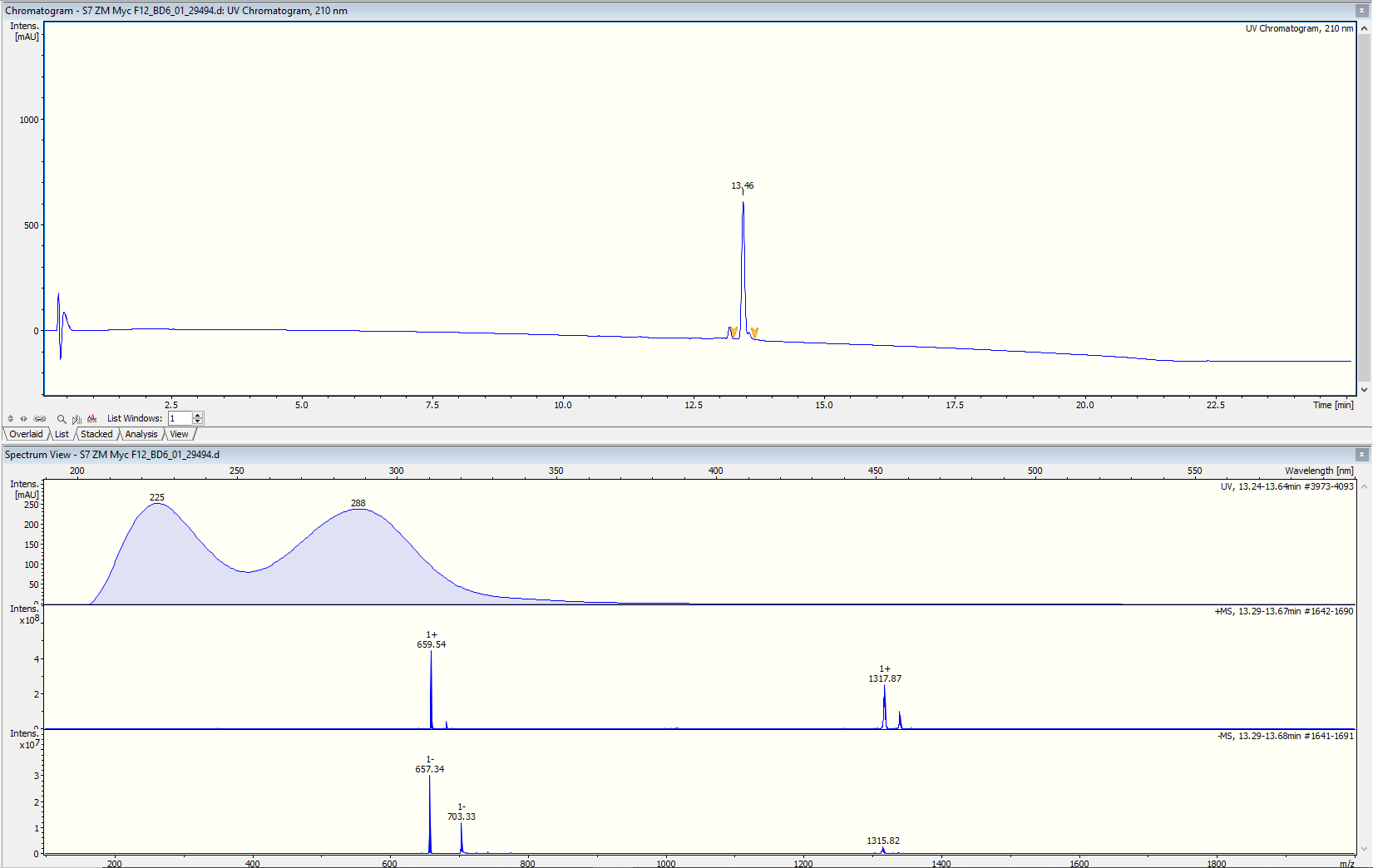


# **Figure S35**: ESIMS data for fusaristatin A (**5**)

# **Figure S36**: ^1^H NMR spectrum (Pyridin-*d_5_*, 500 MHz) of fusaristatin A (**5**)

# **Figure S37**: ^13^C NMR spectrum (Pyridin-*d_5_*, 125 MHz) of fusaristatin A (**5**)

# **Figure S38**: ^1^H-^1^H COSY NMR spectrum (Pyridin-*d_5_*, 500 MHz) of fusaristatin A (**5**)

# **Figure S39**: ^1^H-^13^C HSQC NMR spectrum (Pyridin-*d_5_*, 500 MHz) of fusaristatin A (**5**)

# **Figure S40**: ^1^H-^13^C HMBC NMR spectrum (Pyridin-*d_5_*, 500 MHz) of fusaristatin A (**5**)

# **Figure S41**: ^1^H-^1^H NOESY NMR spectrum (Pyridin-*d_5_*, 500 MHz) of fusaristatin A (**5**)


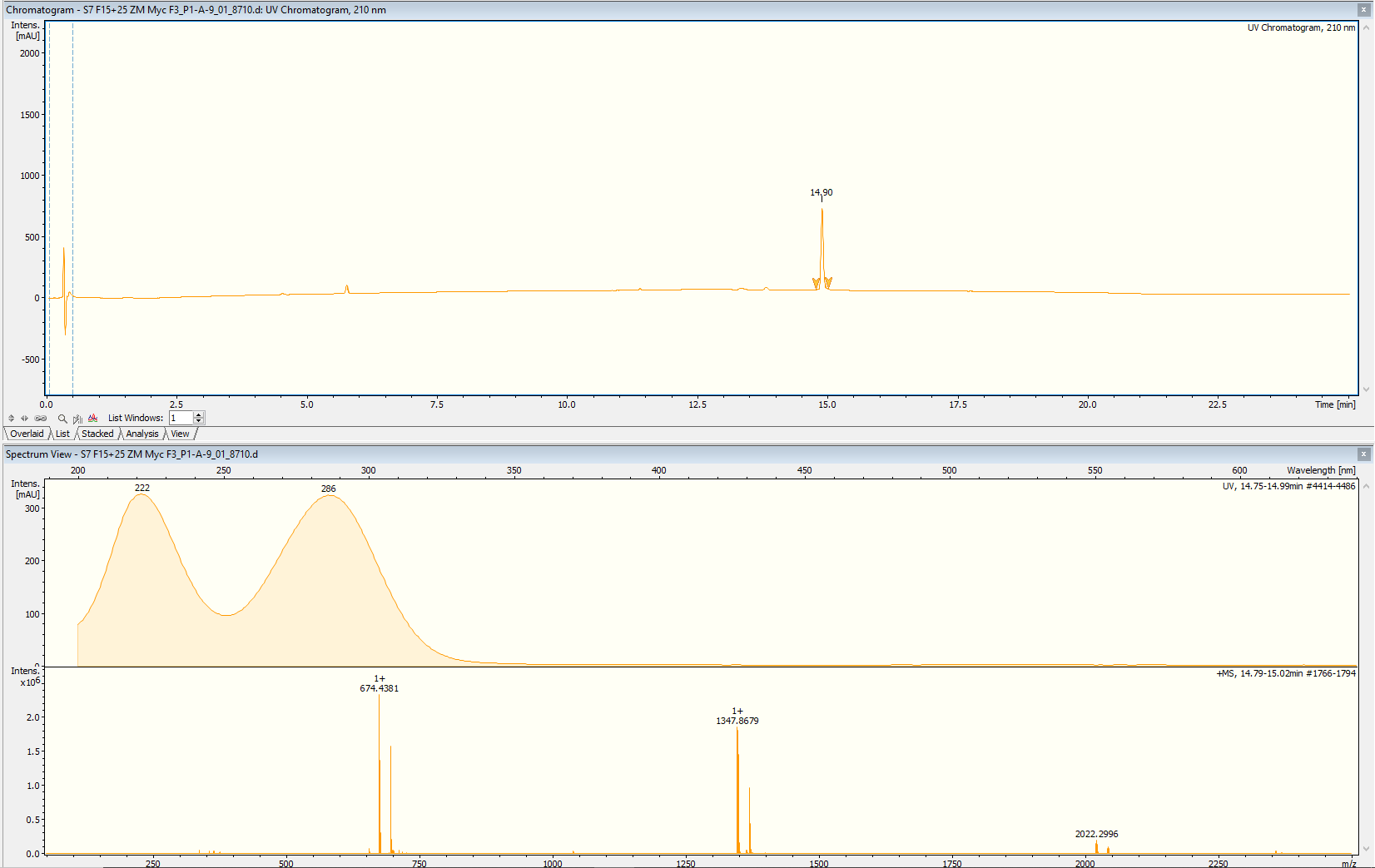


# **Figure S42**: HR-ESI (+) MS data for fusaristatin B (**6**)


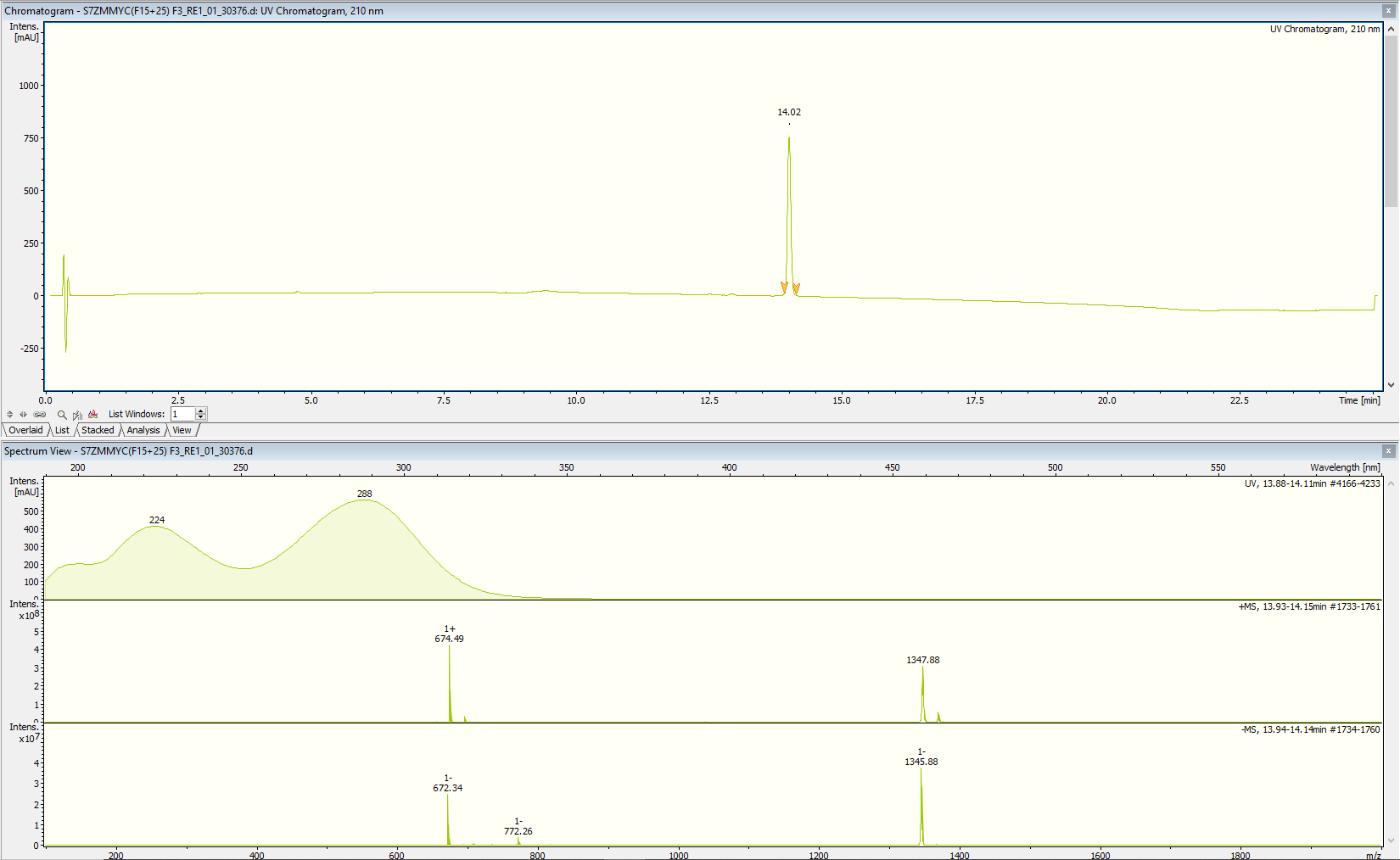


# **Figure S43**: ESIMS data for fusaristatin B (**6**)

# **Figure S44**: ^1^H NMR spectrum (Pyridin-*d_5_*, 700 MHz) of fusaristatin B (**6**)

# **Figure S45**: ^1^H-^1^H COSY NMR spectrum (Pyridin-*d_5_*, 700 MHz) of fusaristatin B (**6**)

# **Figure S46**: ^1^H-^13^C HSQC NMR spectrum (Pyridin-*d_5_*, 700 MHz) of fusaristatin B (**6**)

# **Figure S47**: ^1^H-^13^C HMBC NMR spectrum (Pyridin-*d_5_*, 700 MHz) of fusaristatin B (**6**)


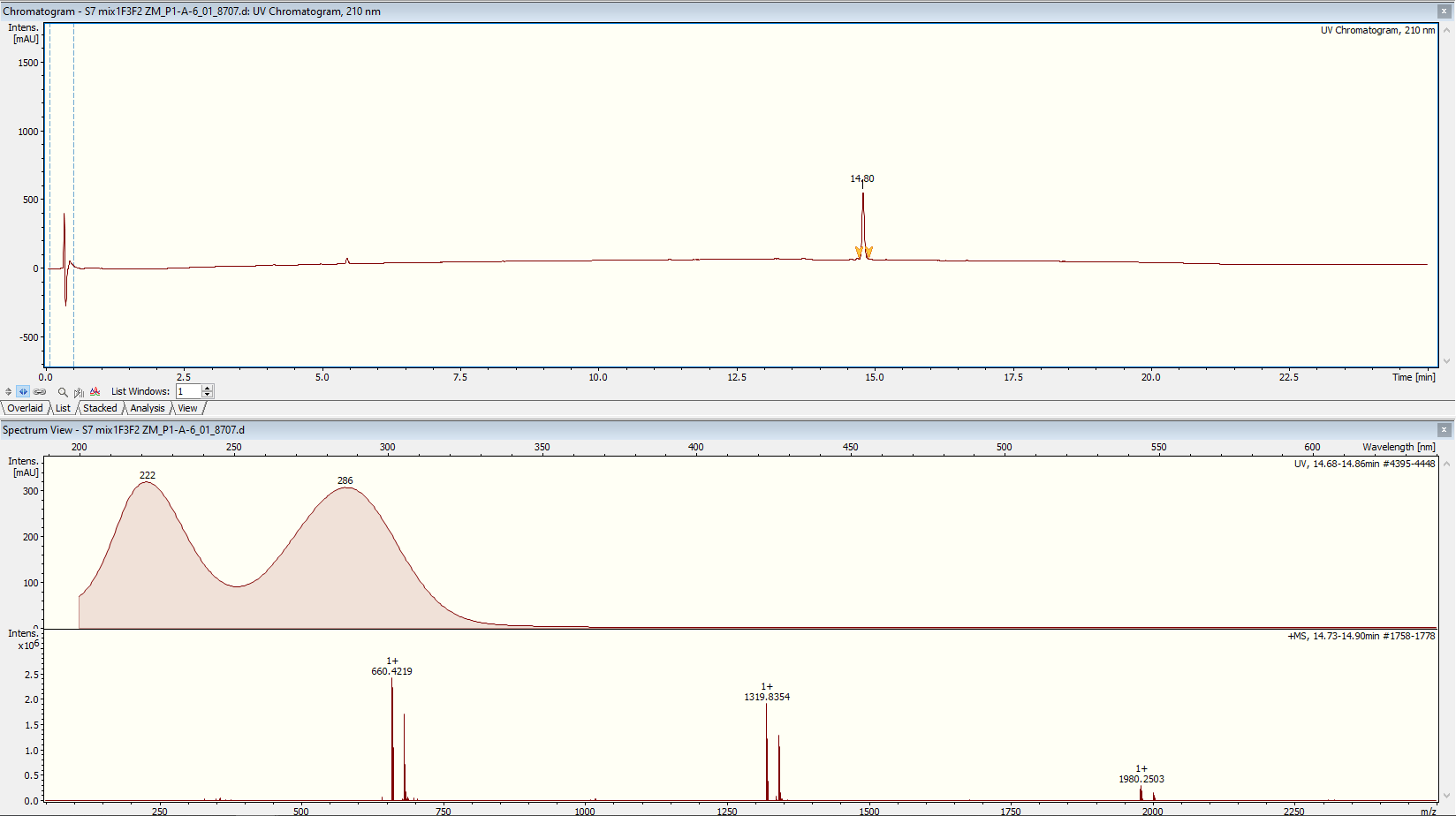


# **Figure S48**: HR-ESI (+) MS data for fusaristatin G (**7**)


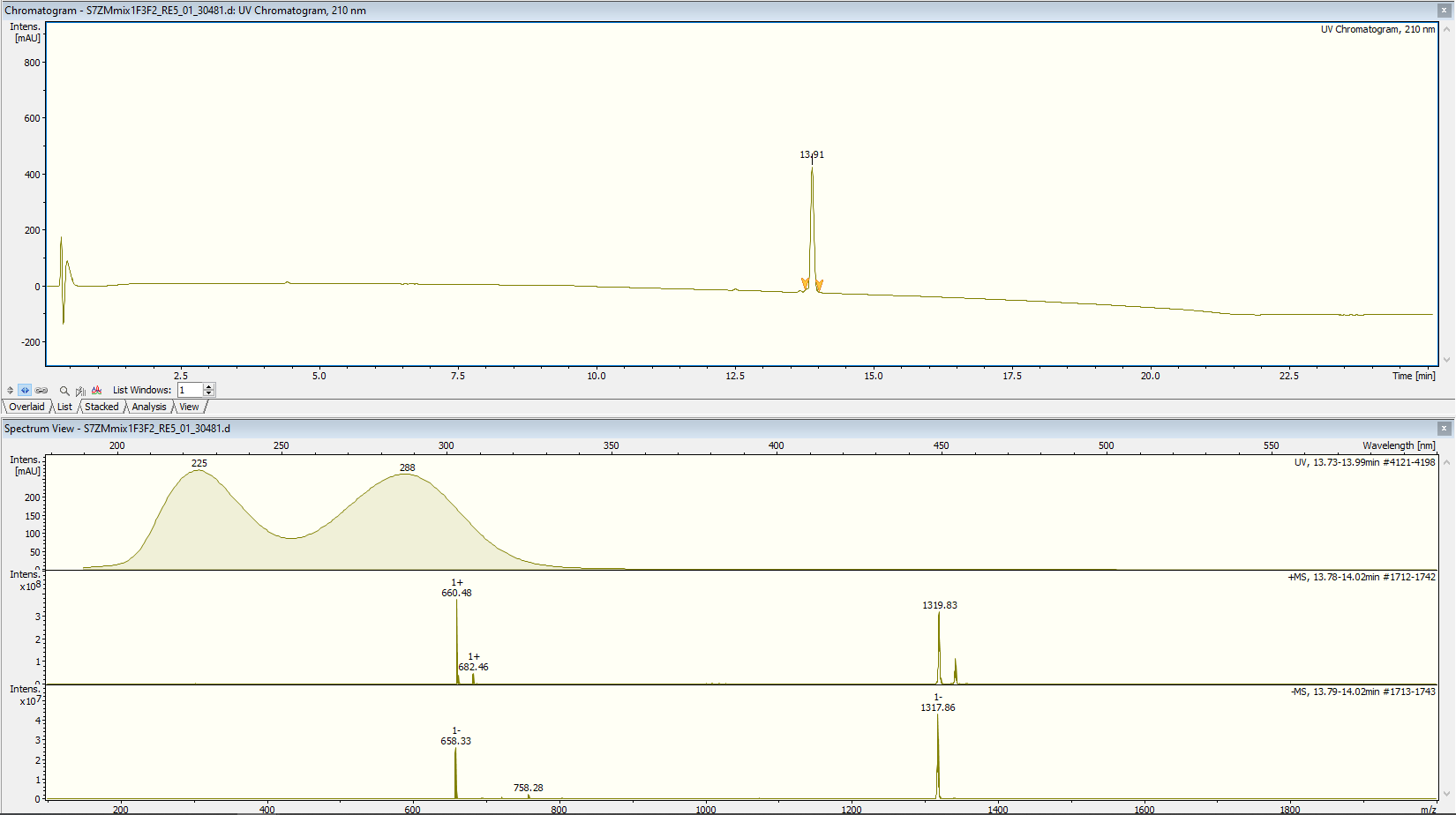


# **Figure S49**: ESIMS data for fusaristatin G (**7**)

# **Figure S50**: ^1^H NMR spectrum (Pyridin-*d_5_*, 700 MHz) of fusaristatin G (**7**)

# **Figure S51**: ^13^C NMR spectrum (Pyridin-*d_5_*, 175 MHz) of fusaristatin G (**7**)

# **Figure S52**: ^1^H-^1^H COSY NMR spectrum (Pyridin-*d_5_*, 700 MHz) of fusaristatin G (**7**)

# **Figure S53**: ^1^H-^13^C HSQC NMR spectrum (Pyridin-*d_5_*, 700 MHz) of fusaristatin G (**7**)

# **Figure S54**: ^1^H-^13^C HMBC NMR spectrum (Pyridin-*d_5_*, 700 MHz) of fusaristatin G (**7**)

# **Figure S55**: ^1^H-^1^H NOESY NMR spectrum (Pyridin-*d_5_*, 700 MHz) of fusaristatin G (**7**)


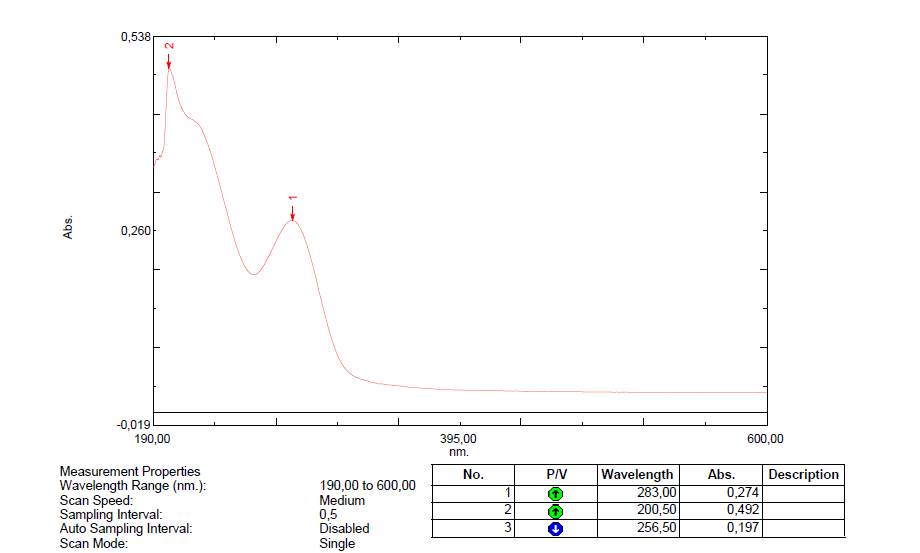


# **Figure S56**: UV/vis spectrum of fusaristatin G (**7**) in MeOH


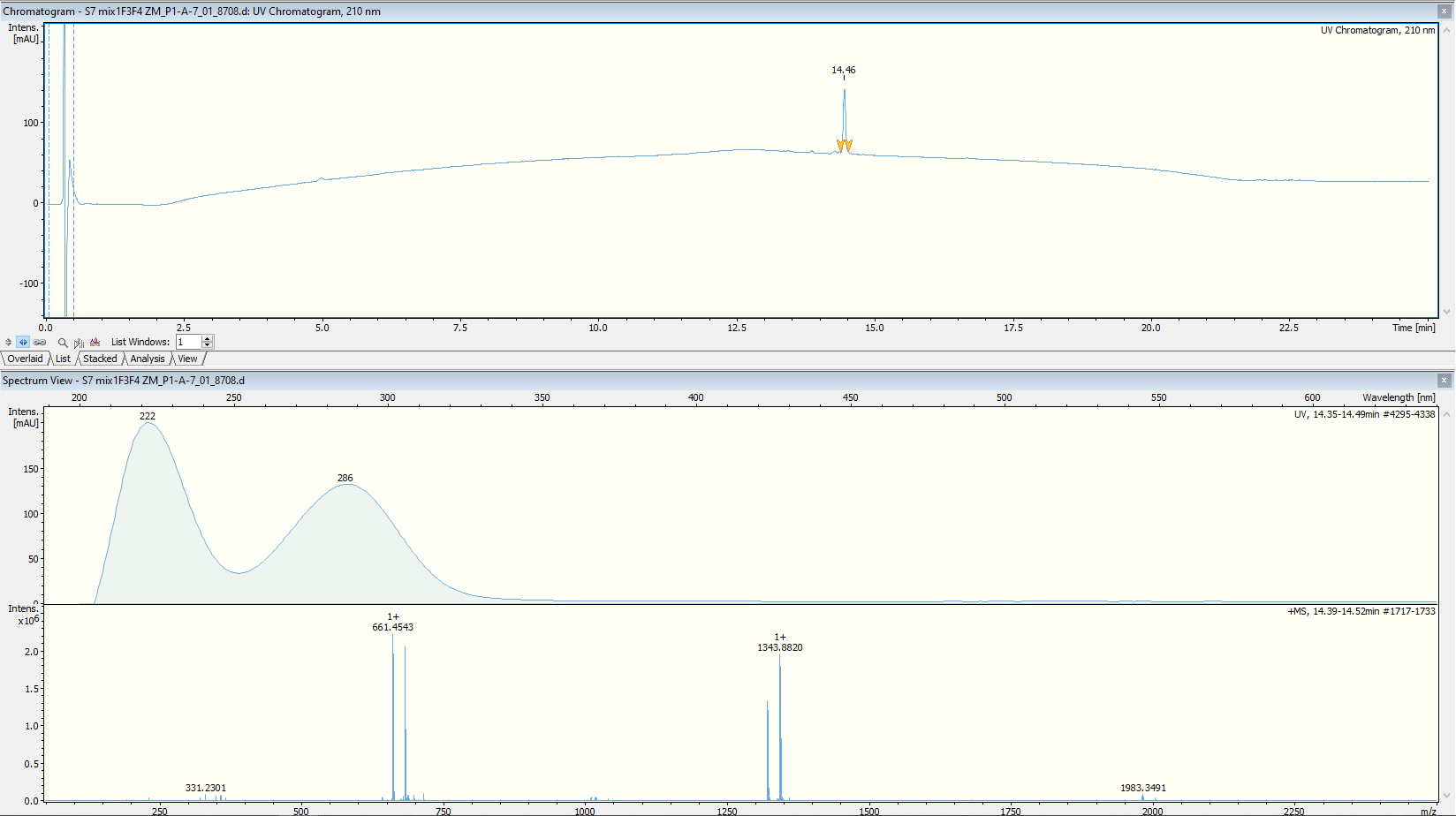


# **Figure S57**: HR-ESI (+) MS data for fusaristatin H (**8**)


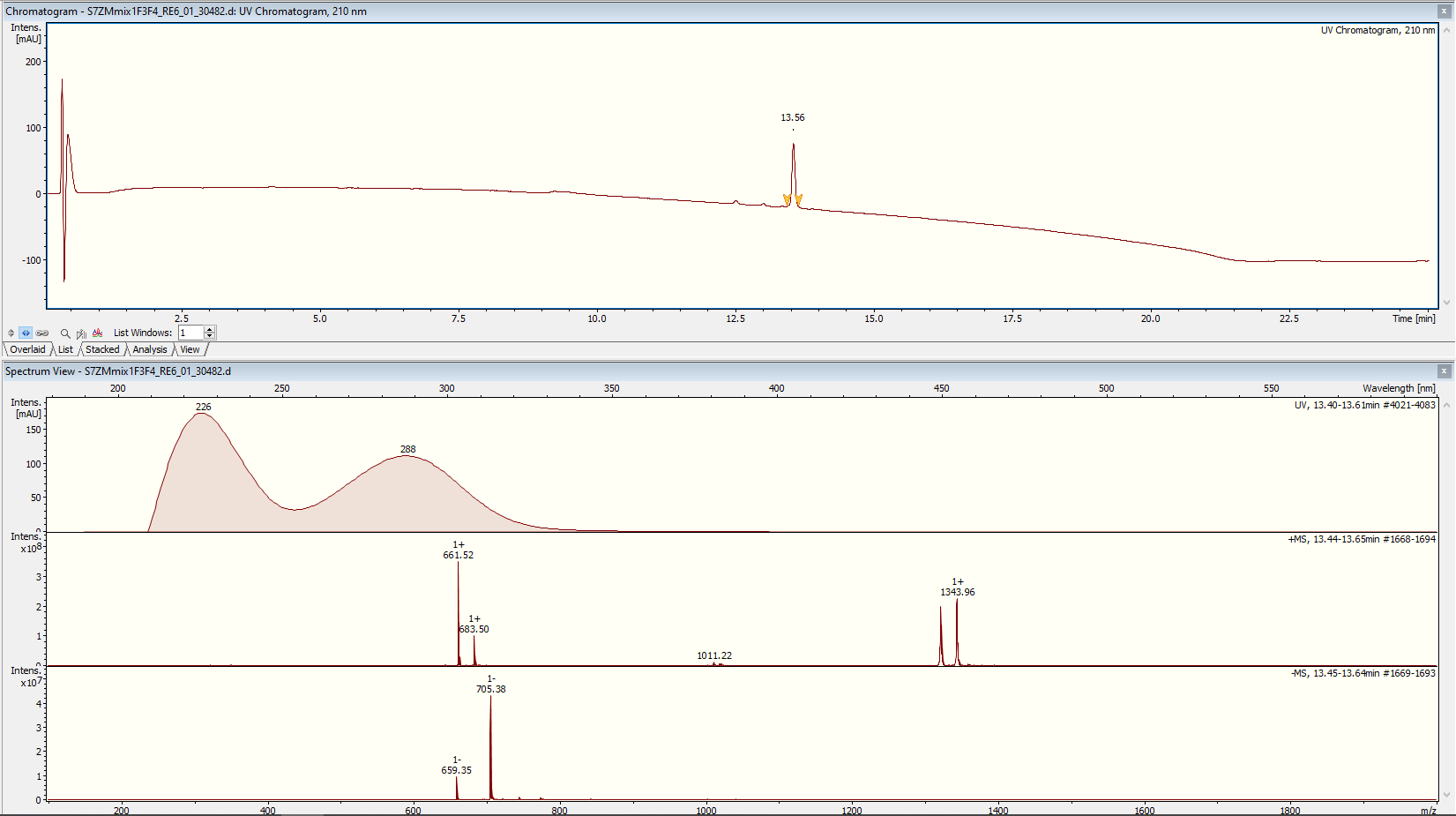


# **Figure S58**: ESIMS data for fusaristatin H (**8**)

# **Figure S59**: ^1^H NMR spectrum (Pyridin-*d_5_*, 700 MHz) of fusaristatin H (**8**)

# **Figure S60**: ^1^H-^1^H COSY NMR spectrum (Pyridin-*d_5_*, 700 MHz) of fusaristatin H (**8**)

# **Figure S61**: ^1^H-^13^C HSQC NMR spectrum (Pyridin-*d_5_*, 700 MHz) of fusaristatin H (**8**)

# **Figure S62**: ^1^H-^13^C HMBC NMR spectrum (Pyridin-*d_5_*, 700 MHz) of fusaristatin H (**8**)

# **Figure S63**: ^1^H-^1^H NOESY NMR spectrum (Pyridin-*d_5_*, 700 MHz) of fusaristatin H (**8**)

**
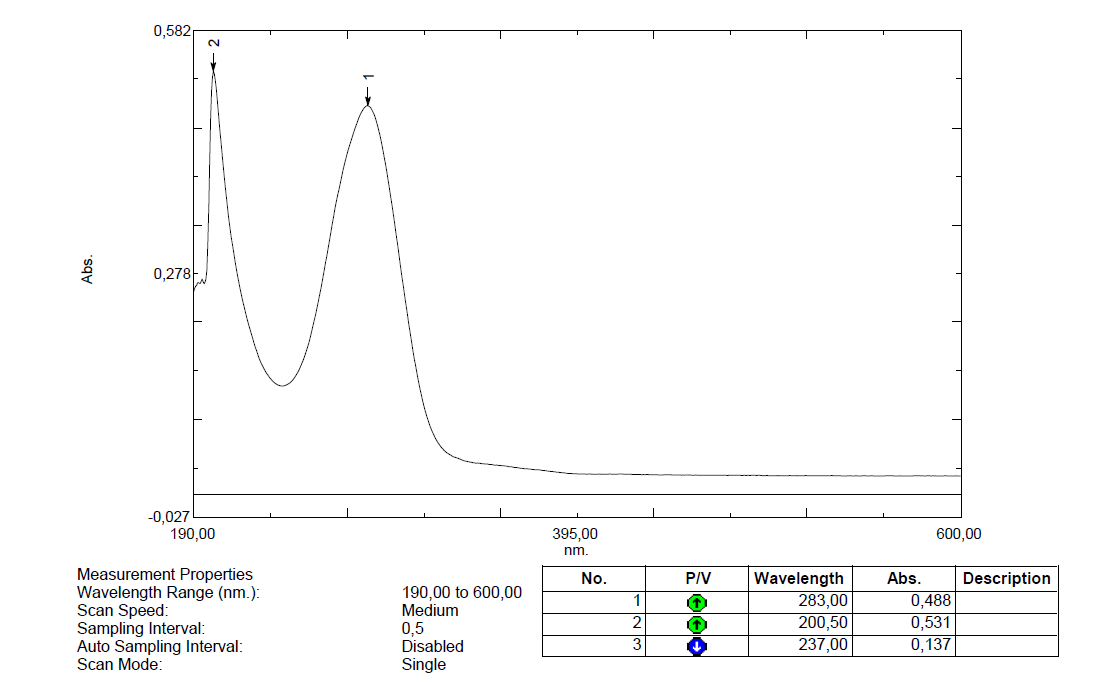
**

# **Figure S64**: UV/vis spectrum of fusaristatin H (**8**)in MeOH

**
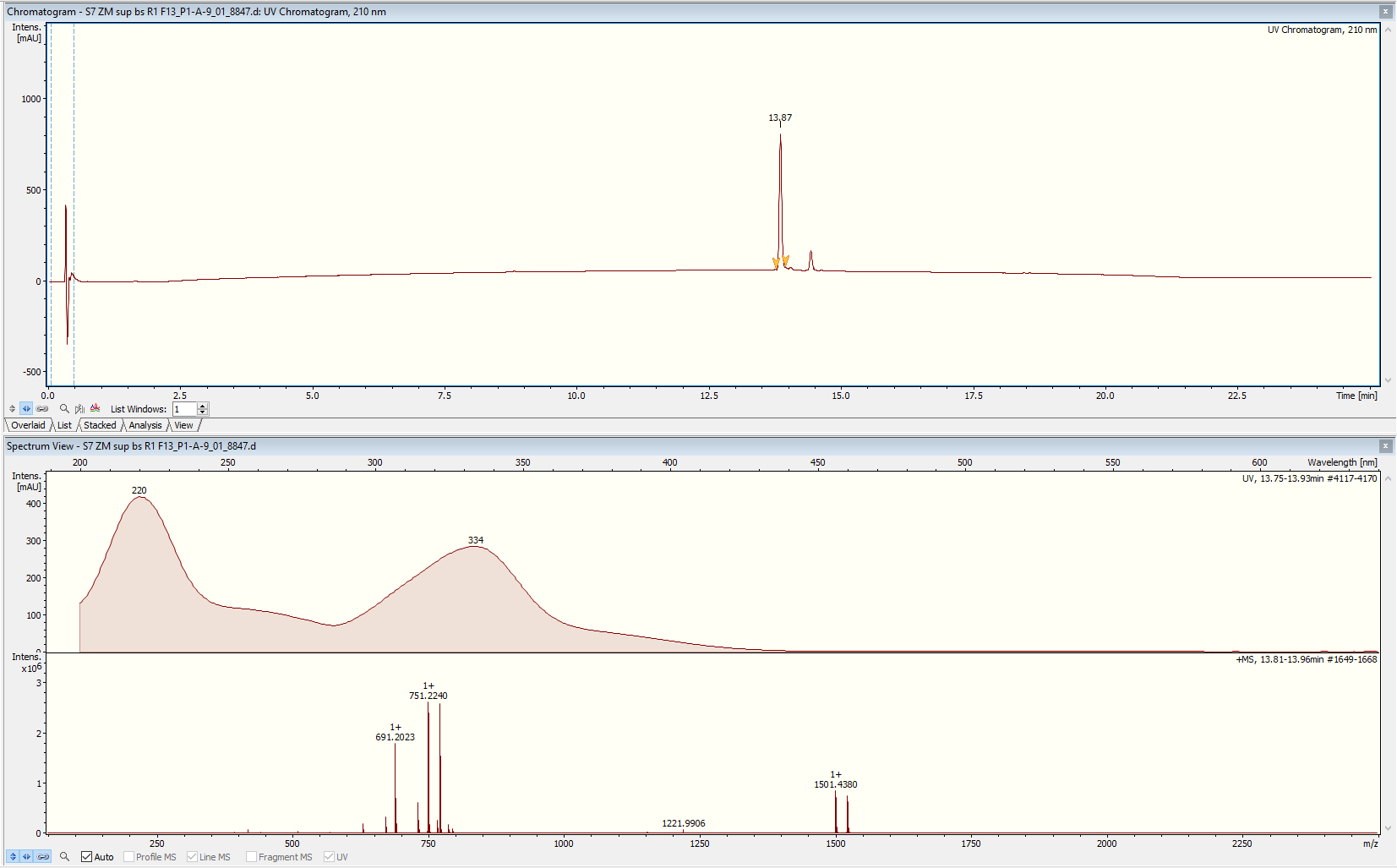
**

# **Figure S65**: HR-ESI (+) MS data for phomoxanthone A (**9**)

**
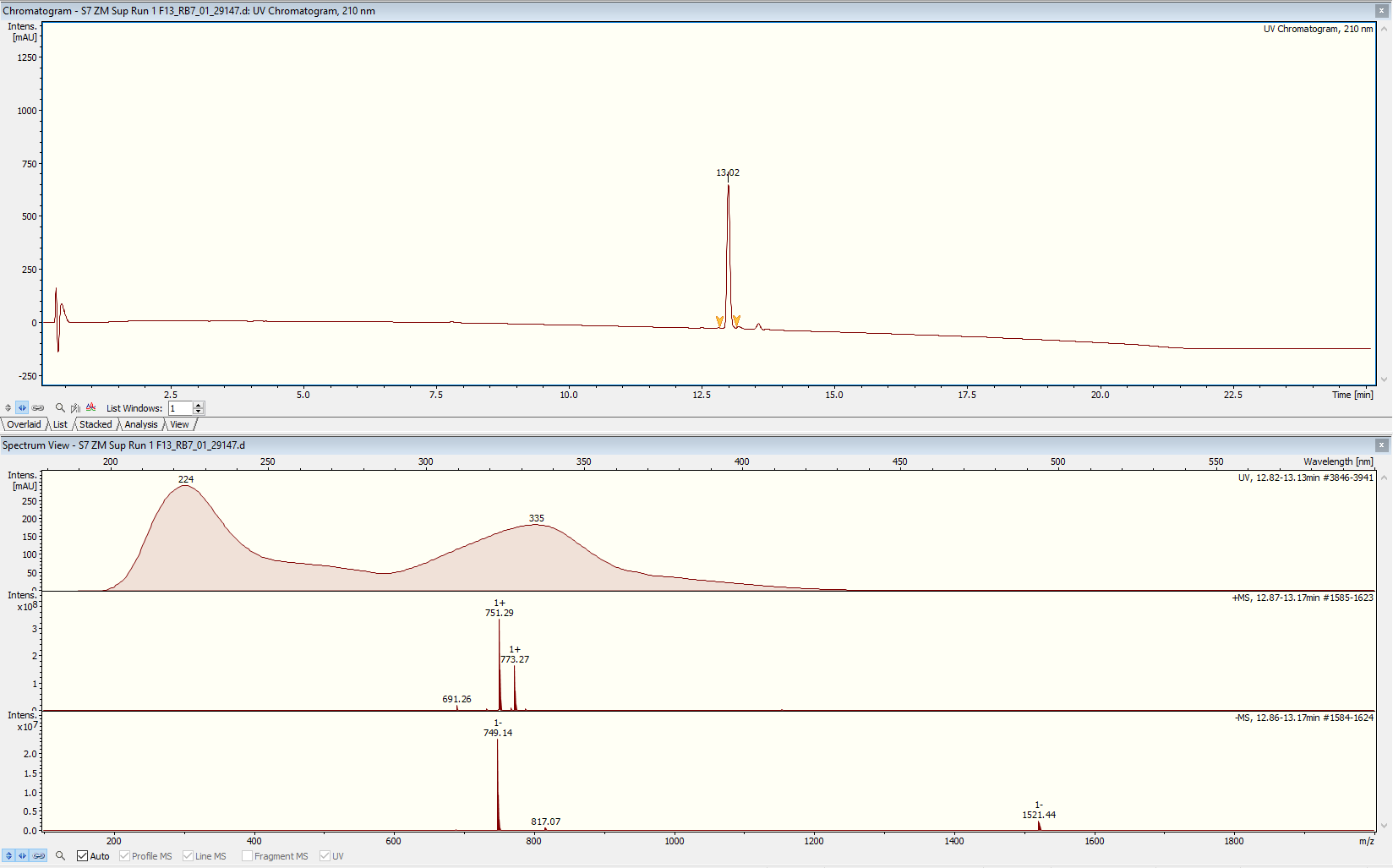
**

# **Figure S66**: ESIMS data for phomoxanthone A (**9**)

# **Figure S67**: ^1^H NMR spectrum (chloroform-*d,* 500 MHz) of phomoxanthone A (**9**)

# **Figure S68**: ^13^C NMR spectrum (chloroform-*d,* 125 MHz) of phomoxanthone A (**9**)

# **Figure S69**: ^1^H-^1^H COSY NMR spectrum (chloroform-*d,* 500 MHz) of phomoxanthone A (**9**)

# **Figure S70**: ^1^H-^13^C HSQC NMR spectrum (chloroform-*d,* 500 MHz) of phomoxanthone A (**9**)

# **Figure S71**: ^1^H-^13^C HMBC NMR spectrum (chloroform-*d,* 500 MHz) of phomoxanthone A (**9**)

# **Figure S72**: ^1^H-^1^H NOESY NMR spectrum (chloroform-*d,* 500 MHz) of phomoxanthone A (**9**)

**
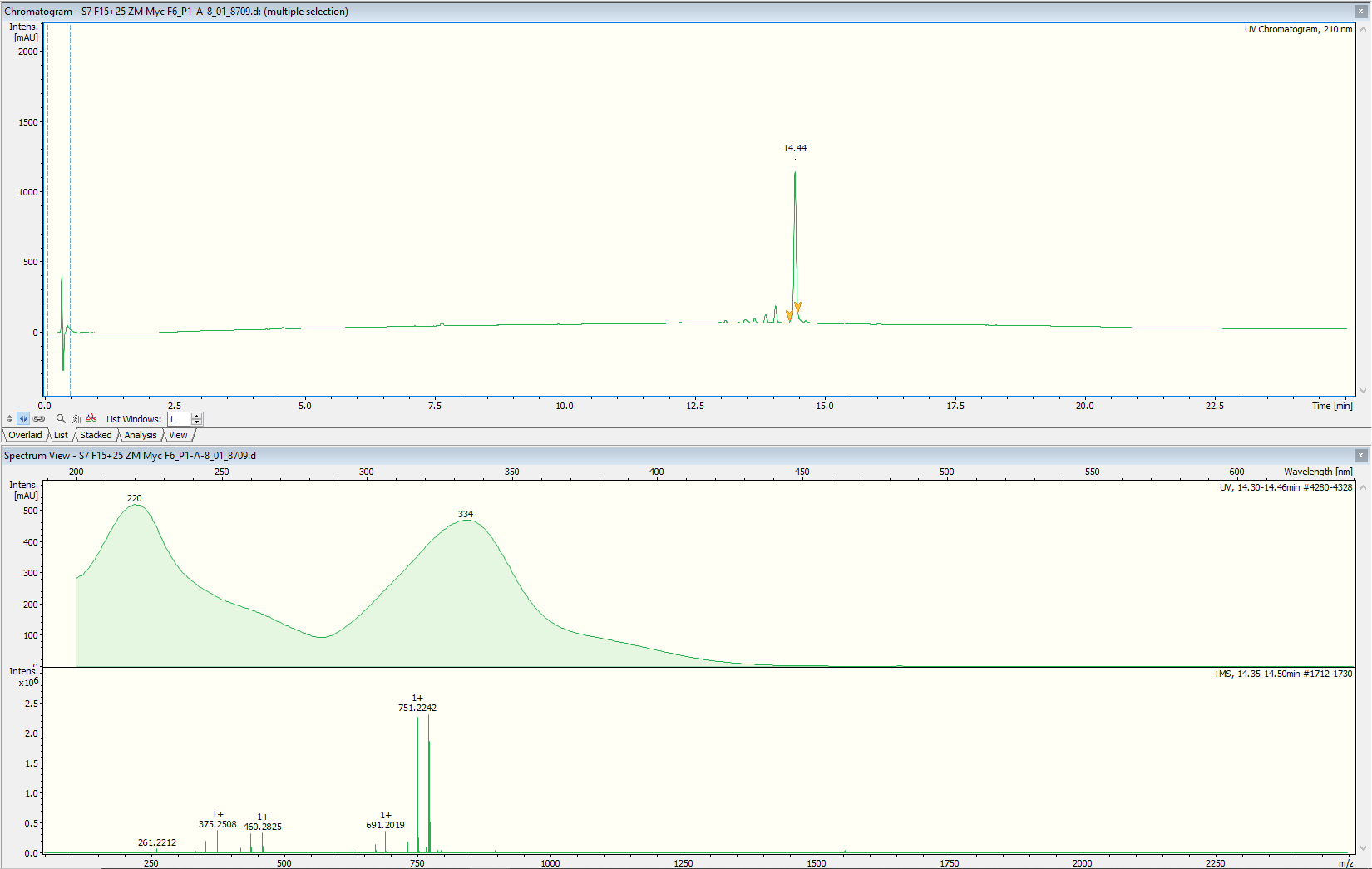
**

# **Figure S73**: HR-ESI (+) MS data for phomoxanthone B (**10**)

**
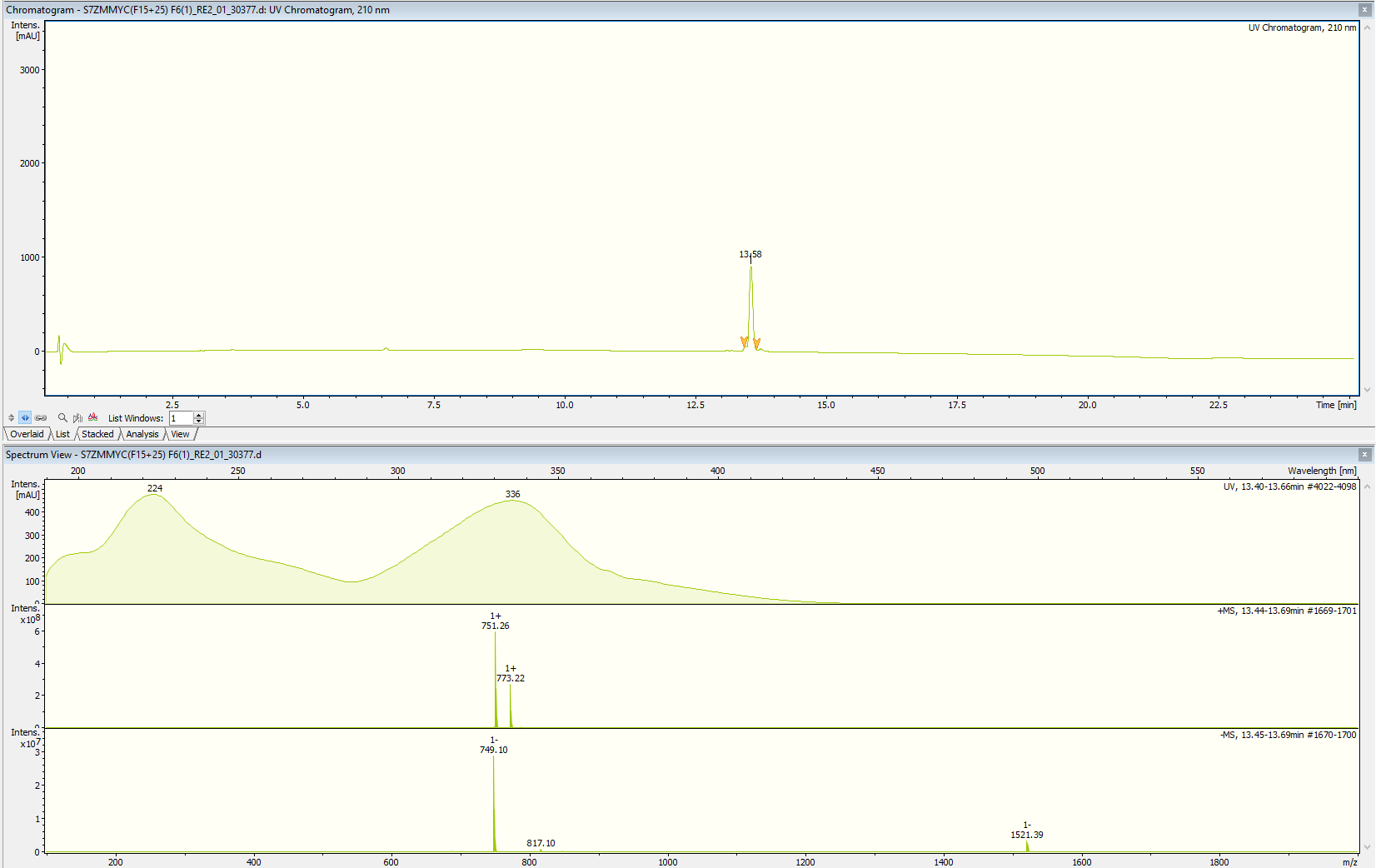
**

# **Figure 74**: ESIMS data for phomoxanthone B (**10**)

# **Figure S75**: ^1^H NMR spectrum (chloroform-*d,* 700 MHz) of phomoxanthone B (**10**)

# **Figure S76**: ^1^H-^1^H COSY NMR spectrum (chloroform-*d,* 700 MHz) of phomoxanthone B (**10**)

# **Figure S77**: ^1^H-^13^C HSQC NMR spectrum (chloroform-*d,* 700 MHz) of phomoxanthone B (**10**)

# **Figure S78**: ^1^H-^13^C HMBC NMR spectrum (chloroform-*d,* 700 MHz) of phomoxanthone B (**10**)

# **Figure S79**: ^1^H-^1^H NOESY NMR spectrum (chloroform-*d,* 700 MHz) of phomoxanthone B (**10**)

**
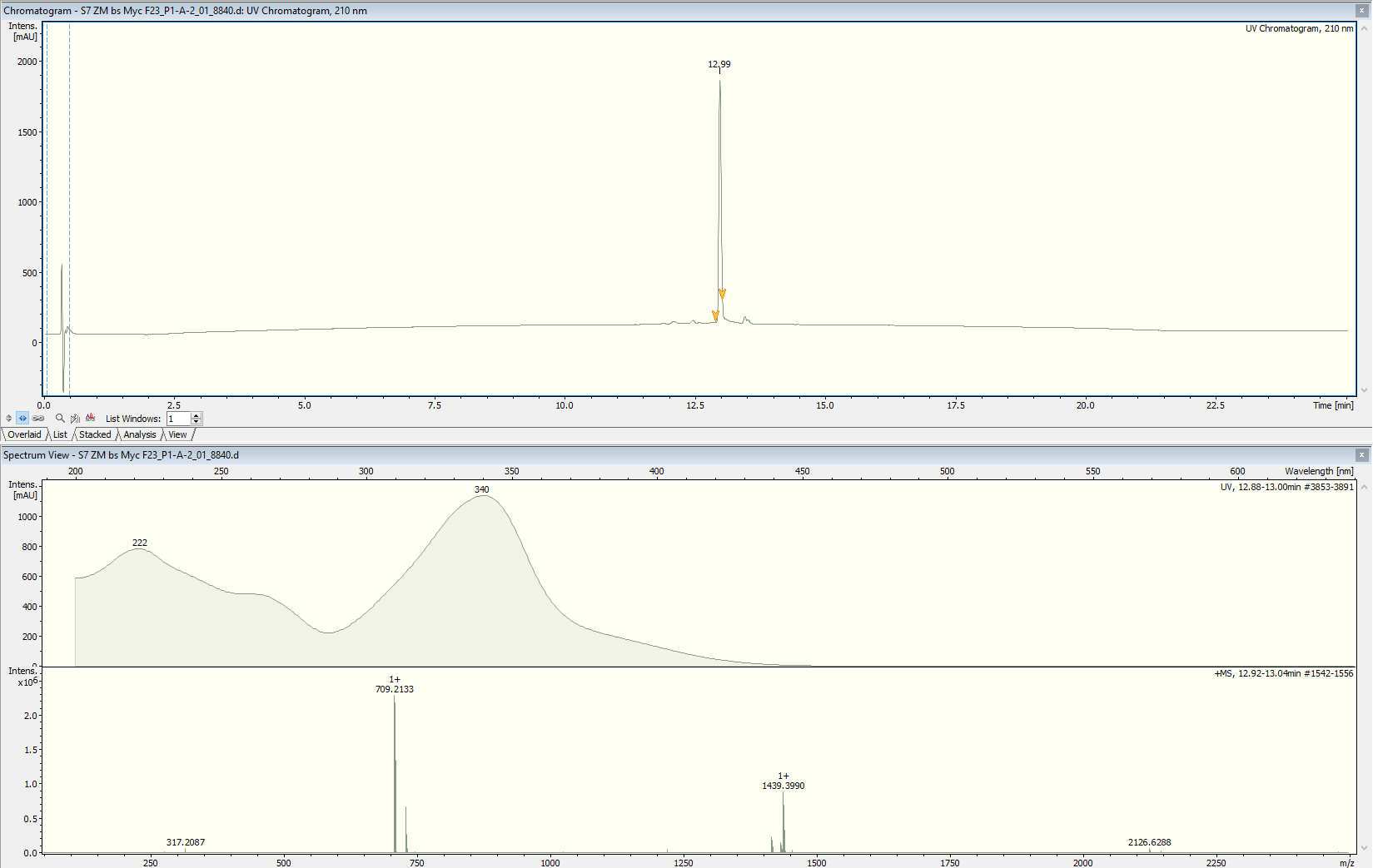
**

# **Figure S80**: HR-ESI (+) MS data for dicerandrol B (**11**)

**
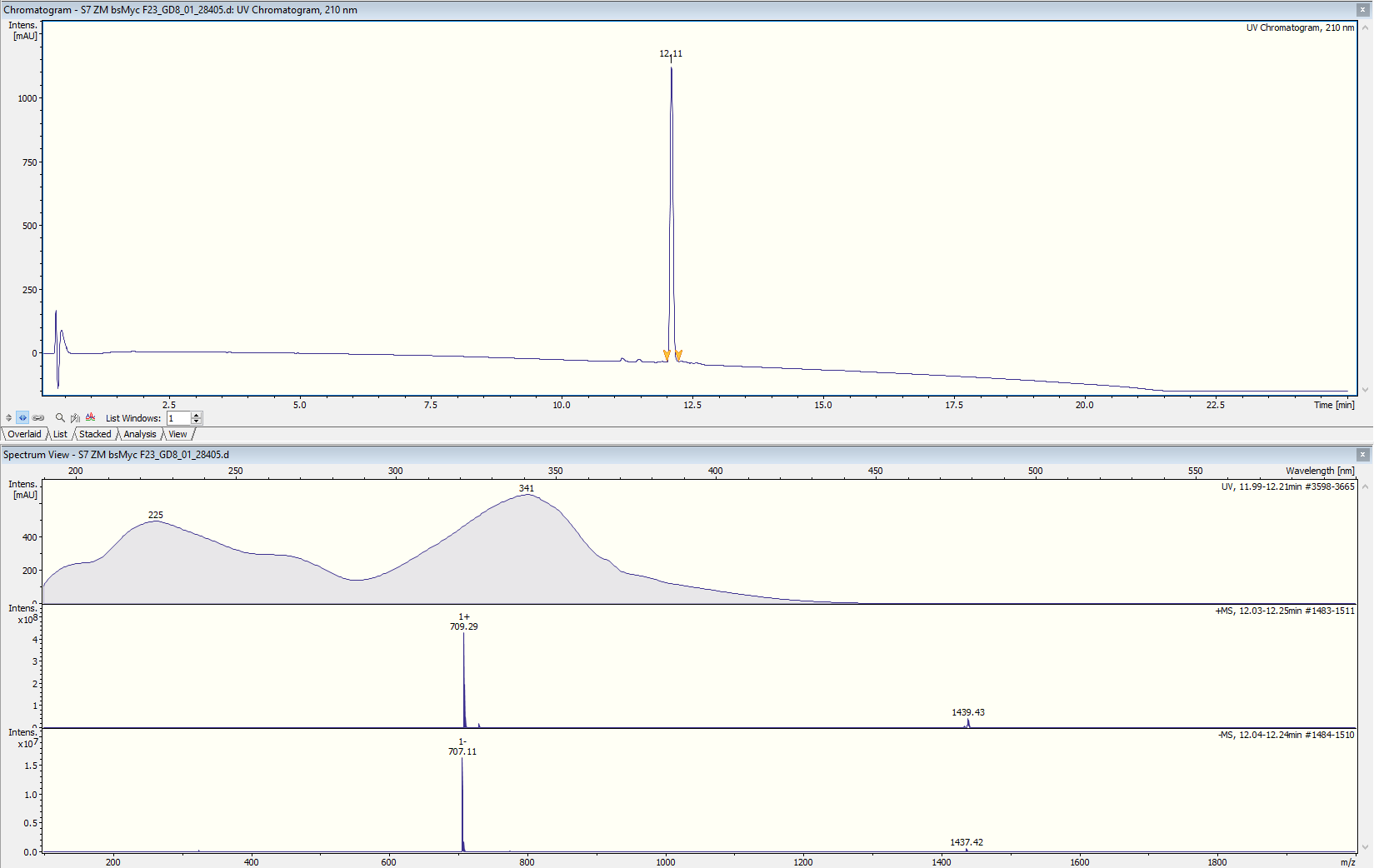
**

# **Figure S81**: ESIMS data for dicerandrol B (**11**)

# **Figure S82**: ^1^H NMR spectrum (chloroform-*d,* 700 MHz) of dicerandrol B (**11**)

# **Figure S83**: ^1^H-^1^H COSY NMR spectrum (chloroform-*d,* 700 MHz) of dicerandrol B (**11**)

# **Figure S84**: ^1^H-^13^C HSQC NMR spectrum (chloroform-*d,* 700 MHz) of dicerandrol B (**11**)

# **Figure S85**: ^1^H-^13^C HMBC NMR spectrum (chloroform-*d,* 700 MHz) of dicerandrol B (**11**)

# **Figure S86**: ^1^H-^1^H NOESY NMR spectrum (chloroform-*d,* 700 MHz) of dicerandrol B (**11**)

**
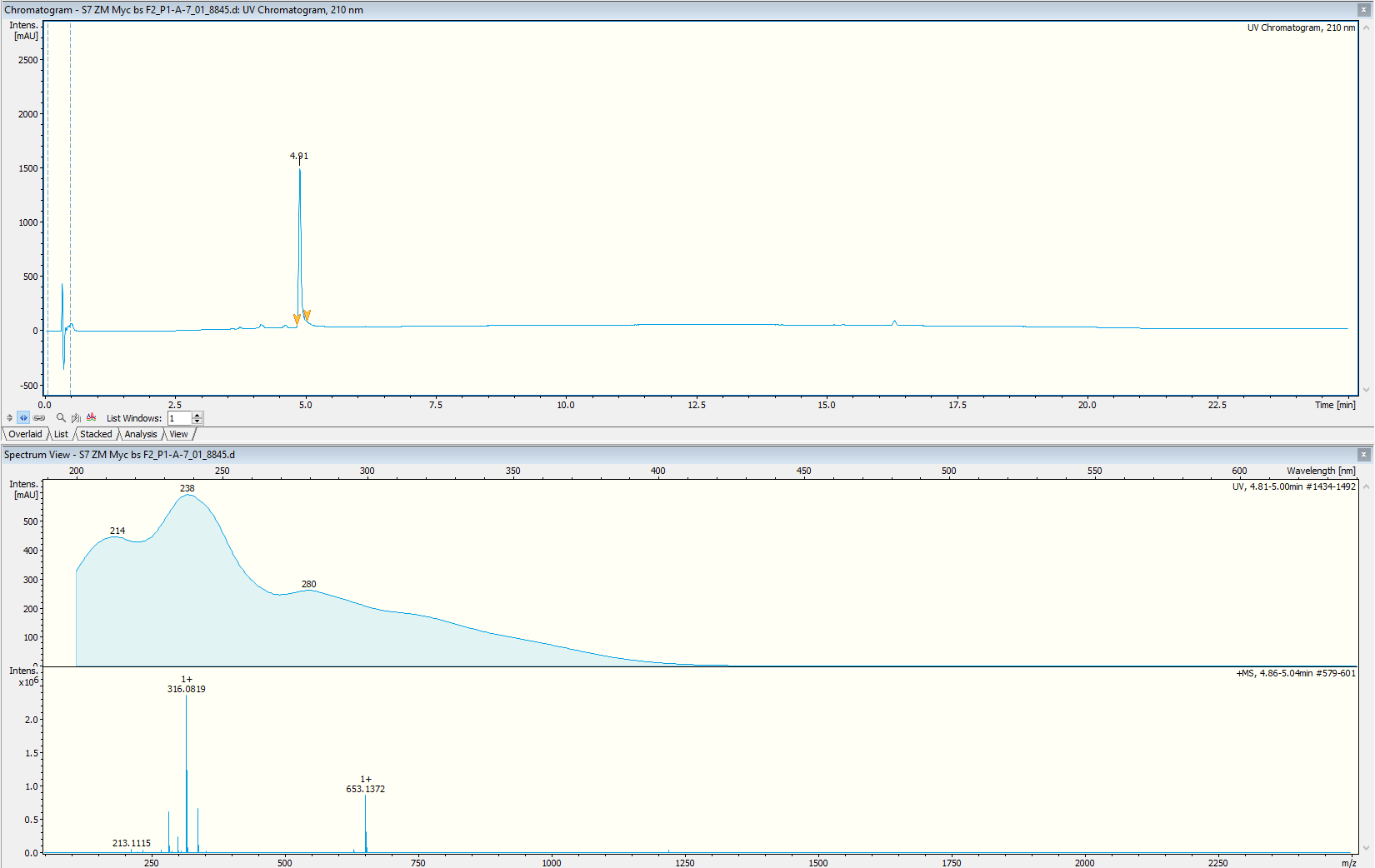
**

# **Figure S87**: HR-ESI (+) MS data for phomochromenone C (**12**)

**
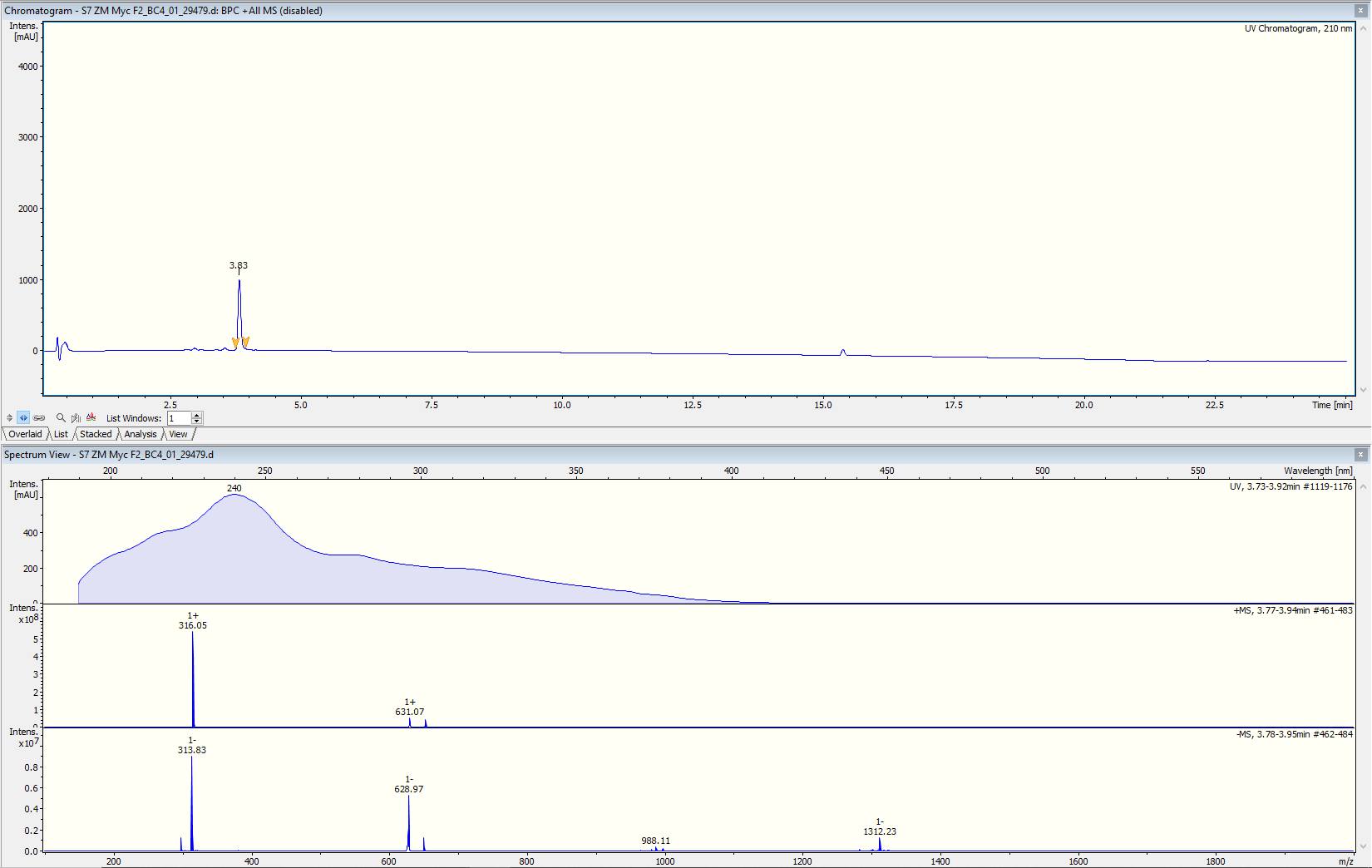
**

# **Figure S88**: ESIMS data for phomochromenone C (**12**)

# **Figure S89**: ^1^H NMR spectrum (chloroform-*d,* 500 MHz) of phomochromenone C (**12**)

# **Figure S90**: ^1^H-^1^H COSY NMR spectrum (chloroform-*d,* 500 MHz) of phomochromenone C (**12**)

# **Figure S91**: ^1^H-^13^C HSQC NMR spectrum (chloroform-*d,* 500 MHz) of phomochromenone C (**12**)

# **Figure S92**: ^1^H-^13^C HMBC NMR spectrum (chloroform-*d,* 500 MHz) of phomochromenone C (**12**)

# **Figure S93**: ^1^H-^1^H NOESY NMR spectrum (chloroform-*d,* 500 MHz) of phomochromenone C (**12**)

**
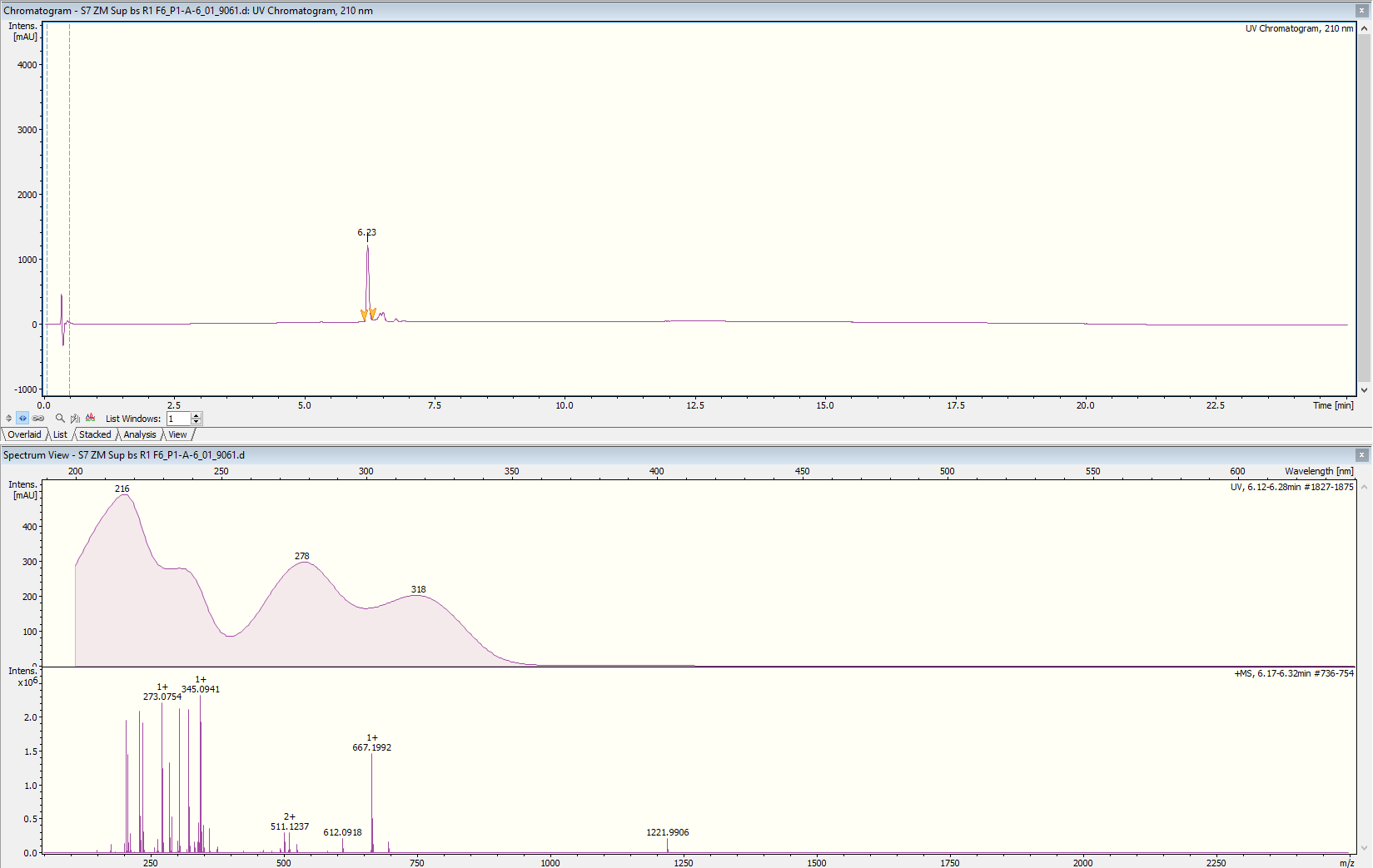
**

# **Figure S94**: HR-ESI (+) MS data for diapochromanone C (**13**)

**
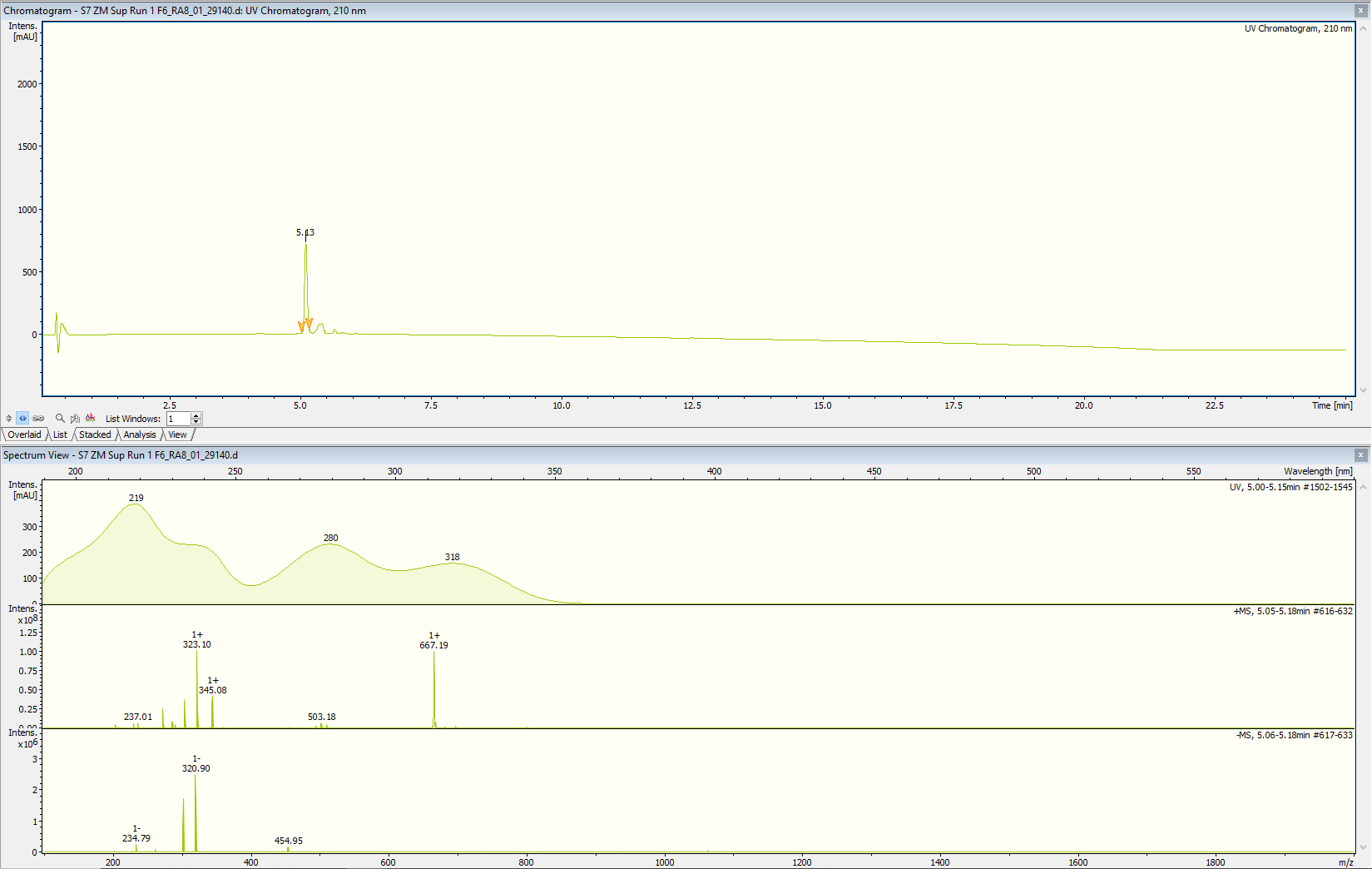
**

# **Figure S95**: ESIMS data for diapochromanone C (**13**)

# **Figure S96**: ^1^H NMR spectrum (DMSO-*d_6_*, 500 MHz) of diapochromanone C (**13**)

# **Figure S97**: ^1^H-^1^H COSY NMR spectrum (DMSO-*d_6_*, 500 MHz) of diapochromanone C (**13**)

# **Figure S98**: ^1^H-^13^C HSQC NMR spectrum (DMSO-*d_6_*, 500 MHz) of diapochromanone C (**13**)

# **Figure S99**: ^1^H-^13^C HMBC NMR spectrum (DMSO-*d_6_*, 500 MHz) of diapochromanone C (**13**)

# **Table S1: Flanking positions of gblocks curated MAFFT alignments of the first phylogenetic analysis. Characteristics of the alignments subsequently used for multigene phylogenetic inference using IQTree 2.**

| **DNA locus** | **Flanking positions** | **Proportion of selected positions** | **Unique** | **Infor** | **Invar** | **Const** |
| --- | --- | --- | --- | --- | --- | --- |
| **ITS** | [160-194], [212-217], [224-228], [242-246], [248-252], [256-264], [272-284], [289-296], [306-310], [321-331], [345-370], [372-380], [382-387], [389-406], [432-581], [722-752], [756-763], [765-779], [810-833], [848-860], [862-868], [878-884], [894-906], [908-912], [914-918], [935-940], [1023-1031] | 454/1090 (41%) | 298 | 182 | 173 | 173 |
| ***cal*** | [136-144], [147-154], [200-207], [215-219], [222-227], [239-246], [248-254], [257-290], [292-299], [341-350], [356-363], [367-379], [435-446], [449-453], [505-510], [544-553], [570-580], [588-714], [750-757], [777-784], [786-792] | 318/1130 (28%) | 245 | 194 | 60 | 60 |
| ***his3*** | [41-174], [264-349], [371-408], [430-467] | 296/556 (53%) | 118 | 61 | 198 | 198 |
| ***tef1*** | [72-93], [120-125], [209-213], [234-250], [253-257], [279-283], [285-304], [355-363], [376-380], [395-400], [496-504], [609-614], [618-625], [647-676] | 153/690 (22%) | 144 | 107 | 24 | 24 |
| ***tub2*** | [350-360], [410-416], [422-430], [441-449], [453-463], [478-521], [535-540], [562-574], [578-583], [602-645], [698-703], [724-730], [734-739], [773-784], [786-794], [809-818], [828-877], [881-887], [892-897], [904-909], [920-926], [943-948], [954-958], [972-979], [981-1009], [1019-1038], [1051-1069], [1074-1078], [1084-1091], [1206-1210], [1220-1224], [1281-1294], [1300-1308], [1355-1387], [1439-1473] | 487/1573 (30%) | 409 | 277 | 69 | 69 |

# **Table S2: Selected edge-linked proportional partition substitution models subjected to IQTree2 calculated with ModelFinder using Bayesian information criterion (BIC).**

| **DNA Locus** | **Model** | **Speed** | **Parameters** |
| --- | --- | --- | --- |
| **ITS** | TIM2+F+R6 | 0.8790 | TIM2{1.44477,4.51416,8.70391}+F{0.234449,0.269379,0.263033,0.233138}+R6{0.536554,0.0546432,0.238083,0.451239,0.113658,1.30845,0.0648552,3.5264,0.0317098,7.68286,0.0151409,15.9969} |
| ***cal*** | TIM3e+R4 | 1.0758 | TIM3e{1.44434,3.75679,6.4024}+FQ+R4{0.419846,0.11732,0.277667,0.843042,0.256309,2.047,0.046178,4.15774} |
| ***his3*** | TIM+F+I+G4 | 0.5075 | TIM{1.58487,2.25529,13.4591}+F{0.176178,0.366201,0.248433,0.209188}+I{0.495632}+G4{0.405926} |
| ***tef1*** | TIM3e+R4 | 1.9209 | TIM3e{1.2577,3.14666,5.61429}+FQ+R4{0.340331,0.0628809,0.246864,0.568165,0.273029,1.58956,0.139776,2.89281} |
| ***tub2*** | TPM3+F+R4 | 1.0733 | TPM3{0.787922,3.467}+F{0.202505,0.348345,0.224092,0.225058}+R4{0.447488,0.13288,0.285347,0.844767,0.235179,2.32065,0.0319855,4.80584} |

# **Table S3: Characteristics of the restricted MAFFT alignments following the first phylogenetic analysis using IQTree 2 for phylogenetic inference.**

| **DNA Locus** | **Sequences** | **Sites** | **Unique** | **Informative** | **Invariant** | **Constant** |
| --- | --- | --- | --- | --- | --- | --- |
| **ITS** | 98 | 572 | 261 | 125 | 370 | 370 |
| ***cal*** | 62 | 449 | 265 | 180 | 212 | 212 |
| ***his3*** | 57 | 373 | 136 | 76 | 255 | 255 |
| ***tef1*** | 88 | 452 | 376 | 249 | 155 | 155 |
| ***tub2*** | 88 | 862 | 548 | 300 | 437 | 437 |

# **Table S4: Selected edge-linked proportional partition substitution models for the restricted phylogenetic analysis subjected to IQTree2 calculated with ModelFinder using Bayesian information criterion (BIC).**

| Locus | **Model** | **Speed** | **Parameters** |
| --- | --- | --- | --- |
| **ITS** | TNe+R4 | 0.7720 | TNe{4.25924,8.29414}+FQ+R4{0.724063,0.0614901,0.17382,1.08852,0.0593564,4.38521,0.0427603,11.8329} |
| ***cal*** | HKY+F+I+G4 | 0.7970 | HKY{4.76727}+F{0.211049,0.311217,0.248678,0.229056}+I{0.327434}+G4{1.57157} |
| ***his3*** | TN+F+I+G4 | 1.0162 | TN{0.830644,5.81824}+F{0.174453,0.372547,0.242232,0.210767}+I{0.449487}+G4{0.369166} |
| ***tef1*** | TIM2+F+I+G4 | 1.8195 | TIM2{1.36894,2.88353,3.67547}+F{0.215419,0.328149,0.21603,0.240402}+I{0.176832}+G4{2.05415} |
| ***tub2*** | HKY+F+I+G4 | 0.8203 | HKY{4.03403}+F{0.199137,0.35834,0.226556,0.215968}+I{0.308603}+G4{1.2599} |

# **Figure S100.** ML (lLN=-50994.2709) phylogram obtained from the combined curated ITS, *cal*, *his3*, *tef1* and *tub2* sequences of our strain and type and reference strains of *Diaporthe* spp. *Diaporthella corylina* CBS 121124 was used as outgroup. Bootstrap support values ≥70/are indicated along branches. Branch lengths are proportional to distance. Alignment deposited in TreeBase (S29473).


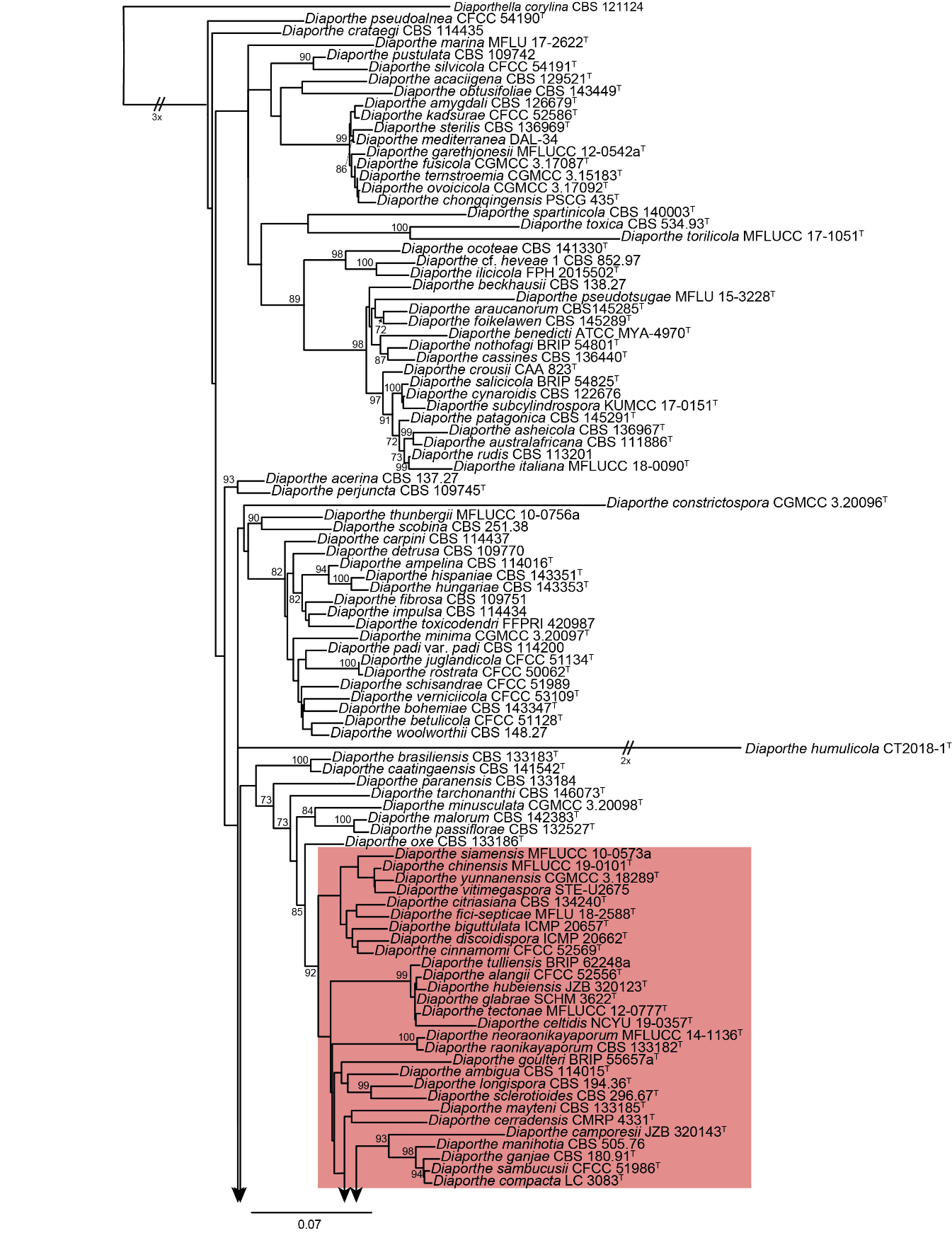


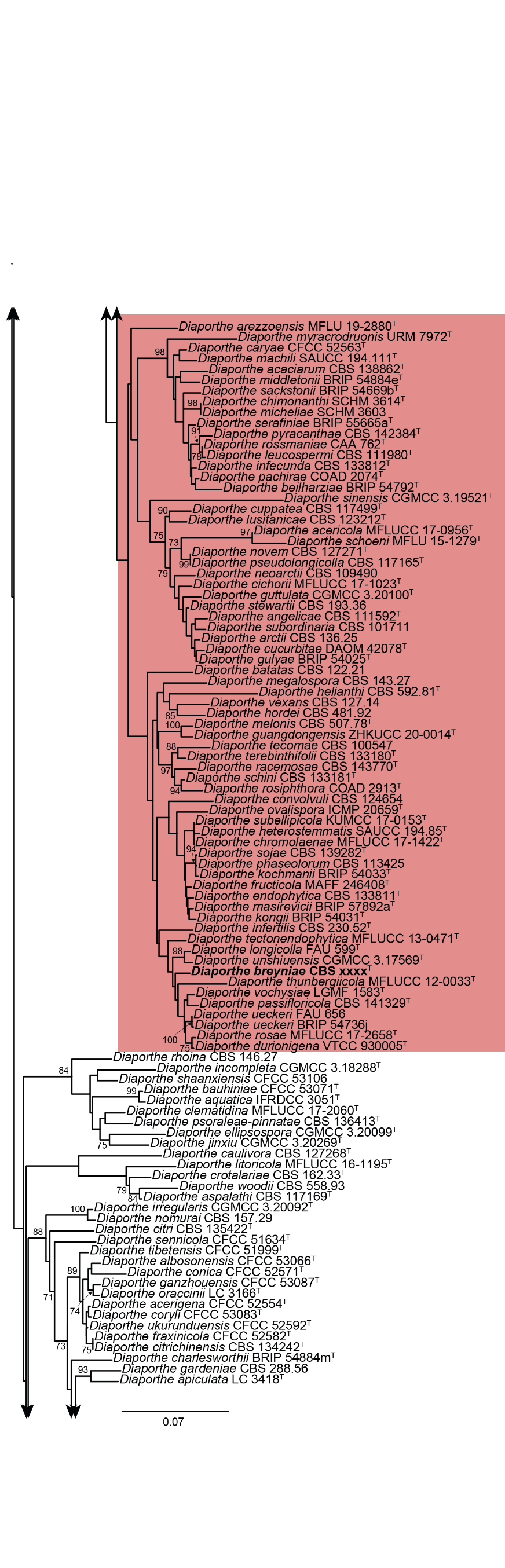


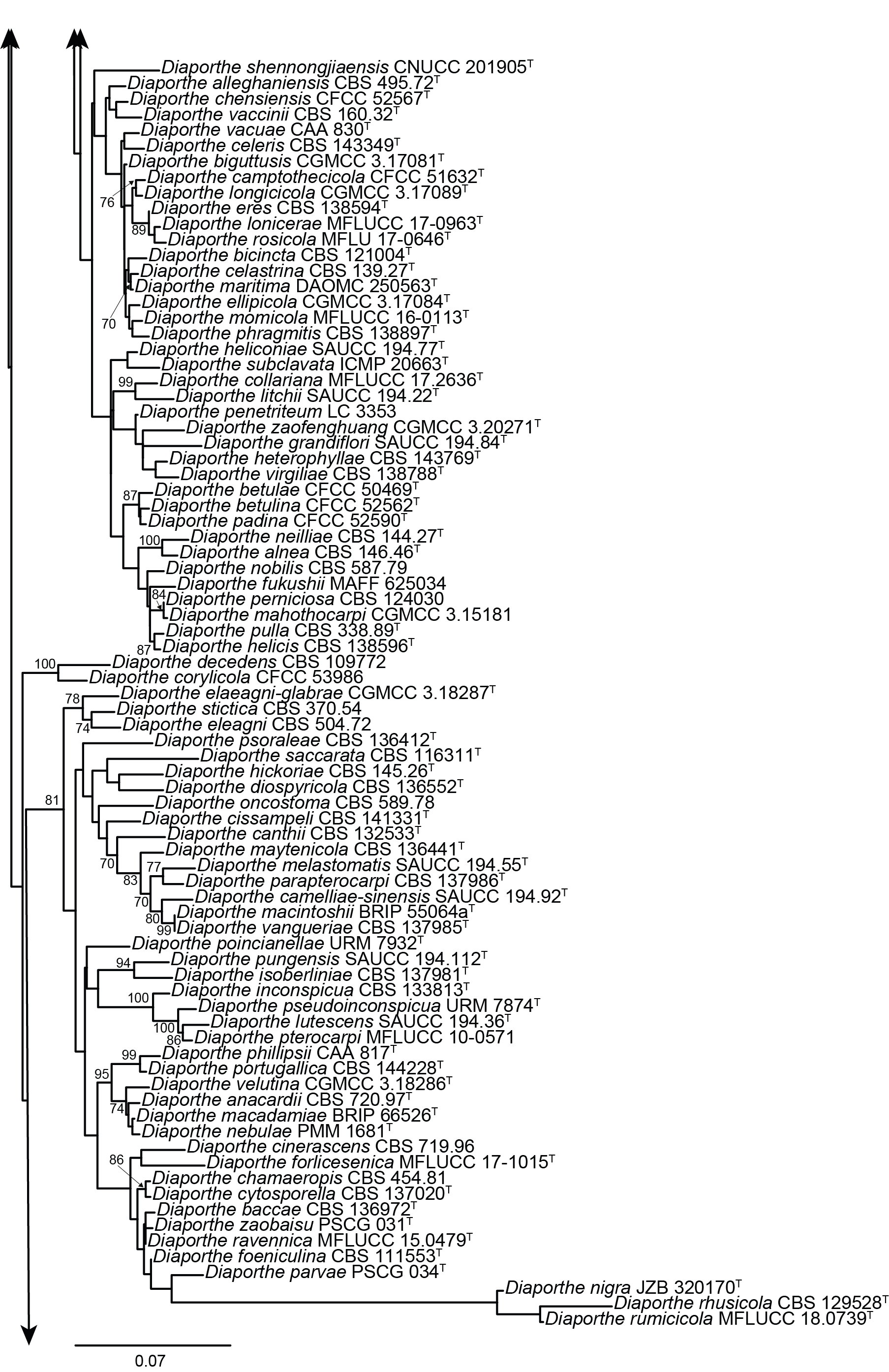


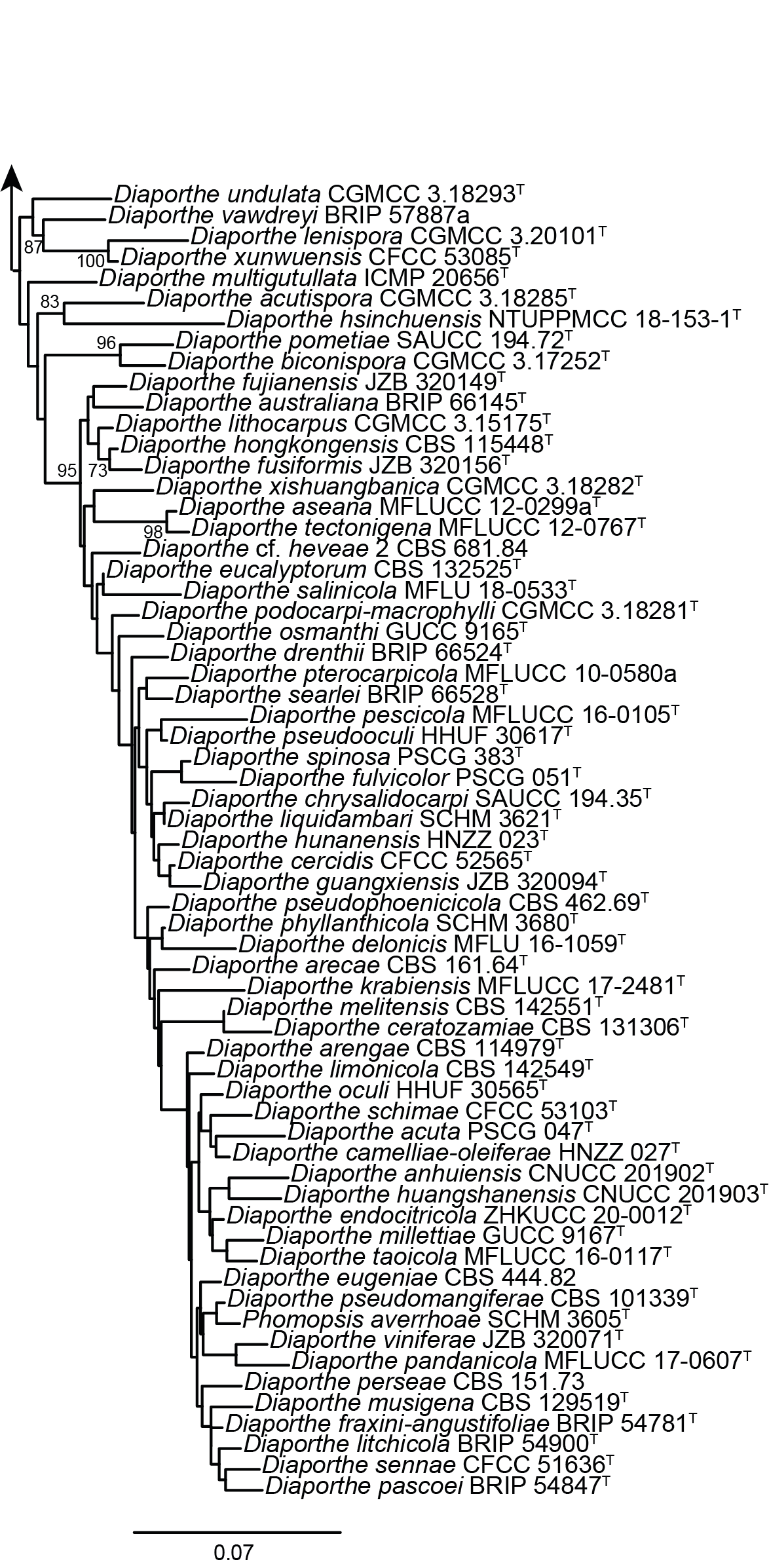


# **Table S5. Strains of *Diaporthe* spp. included in the first phylogenetic study.**

| **Species** | **Isolates^1^** | **GenBank accession numbers^2^** | | | | | **References** |
| --- | --- | --- | --- | --- | --- | --- | --- |
|  |  | **ITS** | ***tub2*** | ***his3*** | ***tef1*** | ***cal*** |  |
| *Diaporthe acaciarum* | CBS 138862^T^ | KP004460 | KP004509 | KP004504 | - | - | Crous et al. (2014b) |
| *D. acaciigena* | CBS 129521^T^ | KC343005 | KC343973 | KC343489 | KC343731 | KC343247 | Gomes et al. (2013) |
| *D. acericola* | MFLUCC 17-0956^T^ | KY964224 | KY964074 | - | KY964180 | KY964137 | Dissanayake et al. (2017a) |
| *D. acerigena* | CFCC 52554^T^ | MH121489 | - | MH121449 | MH121531 | MH121413 | Yang et al. (2018) |
| *D. acerina* | CBS 137.27 | KC343006 | KC343974 | KC343490 | KC343732 | KC343248 | Gomes et al. (2013) |
| *D. acuta* | PSCG 047^T^ | MK626957 | MK691225 | MK726161 | MK654802 | MK691125 | Guo et al. (2020) |
| *D. acutispora* | CGMCC 3.18285^T^ | KX986764 | KX999195 | KX999235 | KX999155 | KX999274 | Gao et al. (2017) |
| *D. alangii* | CFCC 52556^T^ | MH121491 | MH121573 | MH121451 | MH121533 | MH121415 | Yang et al. (2018) |
| *D. albosonensis* | CFCC 53066^T^ | MK432659 | MK578059 | MK443004 | MK578133 | MK442979 | Yang et al. (2020) |
| *D. alleghaniensis* | CBS 495.72^T^ | FJ889444 | KC843228 | KC343491 | GQ250298 | KC343249 | Gomes et al. (2013) |
| *D. alnea* | CBS 146.46^T^ | KC343008 | KC343976 | KC343492 | KC343734 | KC343250 | Gomes et al. (2013) |
| *D. ambigua* | CBS 114015^T^ | KC343010 | KC343978 | KC343494 | KC343736 | KC343252 | Gomes et al. (2013) |
| *D. ampelina* | CBS 114016^T^ | AF230751 | JX275452 | - | GQ250351 | JX197443 | Gomes et al. (2013) |
| *D. amygdali* | CBS 126679^T^ | KC343022 | KC343990 | KC343506 | KC343748 | KC343264 | Gomes et al. (2013) |
| *D. anacardii* | CBS 720.97^T^ | KC343024 | KC343992 | KC343508 | KC343750 | KC343266 | Gomes et al. (2013) |
| *D. angelicae* | CBS 111592^T^ | KC343026 | KC343994 | KC343511 | KC343752 | KC343268 | Gomes et al. (2013) |
| *D. anhuiensis* | CNUCC 201902^T^ | MN219727 | MN227009 | MN224550 | MN224669 | MN224556 | Zhou and Hou (2019) |
| *D. apiculata* | LC 3418^T^ | KP267896 | KP293476 | KP293550 | KP267970 | - | Gao et al. (2016) |
| *D. aquatica* | IFRDCC 3051^T^ | JQ797437 | - | - | - | - | Hu et al. (2012) |
| *D. araucanorum* | CBS 145285^T^ | MN509711 | MN509722 | - | MN509733 | MN974277 | Zapata et al. (2020) |
| *D. arctii* | CBS 136.25 | KC343031 | KC343999 | KC343515 | KC343757 | KC343273 | Gomes et al. (2013) |
| *D. arecae* | CBS 161.64^T^ | KC343032 | KC344000 | KC343516 | KC343758 | KC343274 | Gomes et al. (2013) |
| *D. arengae* | CBS 114979^T^ | KC343034 | KC344002 | KC343518 | KC343760 | KC343276 | Gomes et al. (2013) |
| *D. arezzoensis* | MFLU 19-2880^T^ | MT185503 | MT454055 | - | - | - | Li et al. (2020) |
| *D. aseana* | MFLUCC 12-0299a^T^ | KT459414 | KT459432 | - | KT459448 | KT459464 | Hyde et al. (2016) |
| *D. asheicola* | CBS 136967^T^ | KJ160562 | KJ160518 | - | KJ160594 | KJ160542 | Lombard et al. (2014) |
| *D. aspalathi* | CBS 117169^T^ | KC343036 | KC344004 | KC343520 | KC343762 | KC343278 | Van Rensburg *et al*. (2006) |
| *D. australafricana* | CBS 111886^T^ | KC343038 | KC344006 | KC343522 | KC343764 | KC343280 | Gomes et al. (2013) |
| *D. australiana* | BRIP 66145^T^ | MN708222 | MN696530 | - | MN696522 | - | Wrona et al. (2020) |
| *D. averrhoae* | SCHM 3605^T^ | AY618930 | - | - | - | - | Chang et al. (2005) |
| *D. baccae* | CBS 136972^T^ | KJ160565 | MF418509 | MF418264 | KJ160597 | - | Lombard et al. (2014) |
| *D. batatas* | CBS 122.21 | KC343040 | KC344008 | KC343524 | KC343766 | KC343282 | Gomes et al. (2013) |
| *D. bauhiniae* | CFCC 53071^T^ | MK432648 | MK578051 | MK442995 | MK578124 | MK442970 | Yang et al. (2021a) |
| *D. beckhausii* | CBS 138.27 | KC343041 | KC344009 | KC343525 | KC343767 | KC343283 | Gomes et al. (2013) |
| *D. beilharziae* | BRIP 54792^T^ | JX862529 | KF170921 | - | JX862535 | - | Thompson et al. (2015) |
| *D. benedicti* | ATCC MYA-4970^T^ | KM669929 | - | - | KM669785, | KM669862 | Lawrence *et al*. (2015) |
| *D. betulae* | CFCC 50469^T^ | KT732950 | KT733020 | KT732999 | KT733016 | KT732997 | Du et al. (2016) |
| *D. betulicola* | CFCC 51128^T^ | KX024653 | KX024657 | KX024661 | KX024655 | KX024659 | Du et al. (2016) |
| *D. betulina* | CFCC 52562^T^ | MH121497 | MH121579 | MH121457 | MH121539 | MH121421 | Yang et al. (2018) |
| *D. bicincta* | CBS 121004^T^ | KC343134 | KC344102 | KC343618 | KC343860 | KC343376 | Gomes et al. (2013) |
| *D. biconispora* | CGMCC 3.17252^T^ | KJ490597 | KJ490418 | KJ490539 | KJ490476 | - | Huang et al. (2015) |
| *D. biguttulata* | ICMP 20657^T^ | KJ490582 | KJ490403 | KJ490524 | KJ490461 | - | Huang et al. (2015) |
| *D. biguttusis* | CGMCC 3.17081^T^ | KF576282 | KF576306 | - | KF576257 | - | Gao et al. (2015) |
| *D. bohemiae* | CBS 143347^T^ | MG281015 | MG281188 | MG281361 | MG281536 | MG281710 | Guarnaccia et al. (2018) |
| *D. brasiliensis* | CBS 133183^T^ | KC343042 | KC344010 | KC343526 | KC343768 | KC343284 | Gomes et al. (2013) |
| ***D. breyniae*** | CBS 148910^T^ | **ON400846** | **ON409186** | **ON409187** | **ON409188** | **ON409189** | Present study |
| *D. caatingaensis* | CBS 141542^T^ | KY085927 | KY115600 | KY115605 | KY115603 | KY115597 | Crous et al. (2016a) |
| *D. camelliae-oleiferae* | HNZZ 027^T^ | MZ509555 | MZ504718 | MZ504696 | MZ504707 | MZ504685 | Yang et al. (2021b) |
| *D. camelliae-sinensis* | SAUCC 194.92^T^ | MT822620 | MT855817 | MT855588 | MT855932 | MT855699 | Sun et al. (2021) |
| *D. camporesii* | JZB 320143^T^ | MN533805 | MN561316 | - | - | - | Hyde et al. (2020) |
| *D. camptothecicola* | CFCC 51632^T^ | KY203726 | KY228893 | KY228881 | KY228887 | KY228877 | Yang et al. (2017b) |
| *D. canthii* | CBS 132533^T^ | JX069864 | KC843230 | - | KC843120 | KC843174 | Crous et al. (2012) |
| *D. carpini* | CBS 114437 | KC343044 | KC344012 | KC343528 | KC343770 | KC343286 | Gomes et al. (2013) |
| *D. caryae* | CFCC 52563^T^ | MH121498 | MH121580 | MH121458 | MH121540 | MH121422 | Yang et al. (2018) |
| *D. cassines* | CBS 136440^T^ | KF777155 | - | - | KF777244 | - | Crous et al. (2013) |
| *D. caulivora* | CBS 127268^T^ | KC343045 | KC344013 | KC343529 | KC343771 | KC343287 | Gomes et al. (2013) |
| *D. celastrina* | CBS 139.27^T^ | KC343047 | KC344015 | KC343531 | KC343773 | KC343289 | Gomes et al. (2013) |
| *D. celeris* | CBS 143349^T^ | MG281017 | MG281190 | MG281363 | MG281538 | MG281712 | Guarnaccia et al. (2018) |
| *D. celtidis* | NCYU 19-0357^T^ | MW114346 | MW148266 | - | MW192209 | - | Tennakoon et al. (2021) |
| *D. ceratozamiae* | CBS 131306^T^ | JQ044420 | - | - | - | - | Crous et al. (2011b) |
| *D. cercidis* | CFCC 52565^T^ | MH121500 | MH121582 | MH121460 | MH121542 | MH121424 | Yang et al. (2018) |
| *D. cerradensis* | CMRP 4331^T^ | MN173198 | MW751671 | MW751663 | MT311685 | MW751655 | Iantas et al. (2021) |
| *D.* cf. *heveae* 1 | CBS 852.97 | KC343116 | KC344084 | KC343600 | KC343842 | KC343358 | Gomes et al. (2013) |
| *D.* cf. *heveae* 2 | CBS 681.84 | KC343117 | KC344085 | KC343601 | KC343843 | KC343359 | Gomes et al. (2013) |
| *D. chamaeropis* | CBS 454.81 | KC343048 | KC344016 | KC343532 | KC343774 | KC343290 | Gomes et al. (2013) |
| *D. charlesworthii* | BRIP 54884m^T^ | KJ197288 | KJ197268 | - | KJ197250 | - | Thompson et al. (2015) |
| *D. chensiensis* | CFCC 52567^T^ | MH121502 | MH121584 | MH121462 | MH121544 | MH121426 | Yang et al. (2018) |
| *D. chimonanthi* | SCHM 3614^T^ | AY622993 |  |  |  |  | Chang et al. (2005) |
| *D. chinensis* | MFLUCC 19-0101^T^ | MW187324 | MW245013 | - | MW205017 | MW294199 | de Silva et al. (2021) |
| *D. chongqingensis* | PSCG 435^T^ | MK626916 | MK691321 | MK726257 | MK654866 | MK691209 | Guo et al. (2020) |
| *D. chromolaenae* | MFLUCC 17-1422^T^ | MH094275 | - | - | - | - | Mapook et al. (2020) |
| *D. chrysalidocarpi* | SAUCC 194.35^T^ | MT822563 | MT855760 | MT855532 | MT855876 | MT855646 | Huang et al. (2021) |
| *D. cichorii* | MFLUCC 17-1023^T^ | KY964220 | KY964104 | - | KY964176 | KY964133 | Dissanayake et al. (2017a) |
| *D. cinnamomi* | CFCC 52569^T^ | MH121504 | MH121586 | MH121464 | MH121546 | - | Yang et al. (2018) |
| *D. cinerascens* | CBS 719.96 | KC343050 | KC344018 | KC343534 | KC343776 | KC343292 | Gomes et al. (2013) |
| *D. cissampeli* | CBS 141331^T^ | KX228273 | KX228384 | KX228366 | - | - | Crous et al. (2016b) |
| *D. citri* | CBS 135422^T^ | KC843311 | KC843187 | MF418281 | KC843071 | KC843157 | Udayanga et al. (2014b) |
| *D. citriasiana* | CBS 134240^T^ | JQ954645 | KC357459 | MF418282 | JQ954663 | KC357491 | Huang et al. (2013) |
| *D. citrichinensis* | CBS 134242^T^ | JQ954648 | MF418524 | KJ420880 | JQ954666 | KC357494 | Huang et al. (2013) |
| *D. clematidina* | MFLUCC 17-2060^T^ | MT310657 | MT394623 | - | MT394669 | MT394624 | Phukhamsakda et al. (2020) |
| *D. collariana* | MFLUCC 17-2636^T^ | MG806115 | MG783041 | - | MG783040 | MG783042 | Perera et al. (2018) |
| *D. compacta* | LC3083^T^ | KP267854 | KP293434 | KP293508 | KP267928 | - | Gao et al. (2016) |
| *D. conica* | CFCC 52571^T^ | MH121506 | MH121588 | MH121466 | MH121548 | MH121428 | Yang et al. (2018) |
| *D. constrictospora* | CGMCC 3.20096^T^ | MT385947 | MT424702 | MW022487 | - | MT424718 | Dissanayake et al. (2020) |
| *D. convolvuli* | CBS 124654 | KC343054 | KC344022 | KC343538 | KC343780 | KC343296 | Gomes et al. (2013) |
| *D. coryli* | CFCC 53083^T^ | MK432661 | MK578061 | MK443006 | MK578135 | MK442981 | Yang et al. (2020) |
| *D. corylicola* | CFCC 53986 | MW839880 | MW883977 | MW836717 | MW815894 | MW836684 | Gao et al. (2021) |
| *D. crataegi* | CBS 114435 | KC343055 | KC344023 | KC343539 | KC343781 | KC343297 | Gomes et al. (2013) |
| *D. crotalariae* | CBS 162.33^T^ | KC343056 | KC344024 | KC343540 | KC343782 | KC343298 | Gomes et al. (2013) |
| *D. crousii* | CAA823^T^ | MK792311 | MK837932 | MK871450 | MK828081 | MK883835 | Hilário et al. (2020) |
| *D. cucurbitae* | DAOM 42078^T^ | KM453210 | KP118848 | KM453212 | KM453211 | - | Udayanga et al. (2015) |
| *D. cuppatea* | CBS 117499^T^ | AY339322 | JX275420 | KC343541 | AY339354 | JX197414 | Van Rensburg et al. (2006) |
| *D. cynaroidis* | CBS 122676 | KC343058 | KC344026 | KC343542 | KC343784 | KC343300 | Gomes et al. (2013) |
| *D. cytosporella* | CBS 137020^T^ | KC843307 | KC843221 | MF418283 | KC843116 | KC843141 | Udayanga et al. (2014b) |
| *D. decedens* | CBS 109772 | KC343059 | KC344027 | KC343543 | KC343785 | KC343301 | Gomes et al. (2013) |
| *D. delonicis* | MFLU 16-1059^T^ | MT215490 | MT212209 | - | - | - | Perera et al. (2020) |
| *D. detrusa* | CBS 109770 | KC343061 | KC344029 | KC343545 | KC343787 | KC343303 | Gomes et al. (2013) |
| *D. diospyricola* | CBS 136552^T^ | KF777156 | - | - | - | - | Crous et al. (2013) |
| *D. discoidispora* | ICMP 20662^T^ | KJ490624 | KJ490445 | KJ490566 | KJ490503 | - | Huang et al. (2015) |
| *D. drenthii* | BRIP 66524^T^ | MN708229 | MN696537 | - | MN696526 | - | Wrona et al. (2020) |
| *D. durionigena* | VTCC 930005^T^ | MN453530 | MT276159 | - | MT276157 | - | Crous et al. (2021) |
| *D. elaeagni-glabrae* | CGMCC 3.18287^T^ | KX986779 | KX999212 | KX999251 | KX999171 | KX999281 | Gao et al. (2017) |
| *D. eleagni* | CBS 504.72 | KC343064 | KC344032 | KC343548 | KC343790 | KC343306 | Gomes et al. (2013) |
| *D. ellipicola* | CGMCC 3.17084^T^ | KF576270 | KF576291 | - | KF576245 | - | Gao et al. (2015) |
| *D. ellipsospora* | CGMCC 3.20099^T^ | MT385949 | MT424704 | MW022488 | MT424684 | MT424720 | Dissanayake et al. (2020) |
| *D. endocitricola* | ZHKUCC 20-0012 ^T^ | MT355682 | MT409290 | - | MT409336 | MT409312 | Dong et al. (2021) |
| *D. endophytica* | CBS 133811^T^ | KC343065 | KC344033 | KC343549 | KC343791 | KC343307 | Gomes et al. (2013) |
| *D. eres* | CBS 138594^T^ | KJ210529 | KJ420799 | KJ420850 | KJ210550 | KJ434999 | Udayanga et al. (2014a) |
| *D. eucalyptorum* | CBS 132525^T^ | JX069862 | - | - | - | - | Crous et al. (2012) |
| *D. eugeniae* | CBS 444.82 | KC343098 | KC344066 | KC343582 | KC343824 | KC343340 | Gomes et al. (2013) |
| *D. fibrosa* | CBS 109751 | KC343099 | KC344067 | KC343583 | KC343825 | KC343341 | Gomes et al. (2013) |
| *D. fici-septicae* | MFLU 18-2588^T^ | MW114348 | MW148268 | - | MW192211 | - | Tennakoon et al. (2021) |
| *D. foeniculina* | CBS 111553^T^ | KC343101 | KC344069 | KC343585 | KC343827 | KC343343 | Gomes et al. (2013) |
| *D. foikelawen* | CBS 145289^T^ | MN509713 | MN509724 | - | MN509735 | MN974278 | Zapata et al. (2020) |
| *D. forlicesenica* | MFLUCC 17-1015^T^ | KY964215 | KY964099 | - | KY964171 | - | Dissanayake et al. (2017a) |
| *D. fraxini-angustifoliae* | BRIP 54781^T^ | JX862528 | KF170920 | - | JX852534 | - | Tan et al. (2013) |
| *D. fraxinicola* | CFCC 52582^T^ | MH121517 | - | - | MH121559 | MH121435 | Yang et al. (2018) |
| *D. fructicola* | MAFF 246408^T^ | LC342734 | LC342736 | LC342737 | LC342735 | LC342738 | Crous et al. (2019) |
| *D. fujianensis* | JZB 320149^T^ | MW010212 | MW056008 | - | MW20523 | MW205212 | Manawasinghe et al. (2021) |
| *D. fukushii* | MAFF 625034 | JQ807469 | - | - | JQ807418 | - | Baumgartner et al. (2013) |
| *D. fulvicolor* | PSCG 051^T^ | MK626859 | MK691236 | MK726163 | MK654806 | MK691132 | Guo et al. (2020) |
| *D. fusicola* | CGMCC 3.17087^T^ | KF576281 | KF576305 | - | KF576256 | KF576233 | Gao et al. (2015) |
| *D. fusiformis* | JZB 320156^T^ | MW010218 | MW056014 | - | MW205234 | MW205218 | Manawasinghe et al. (2021) |
| *D. ganjae* | CBS 180.91^T^ | KC343112 | KC344080 | KC343596 | KC343838 | KC343354 | Gomes et al. (2013) |
| *D. ganzhouensis* | CFCC 53087^T^ | MK432665 | MK578065 | MK443010 | MK578139 | MK442985 | Yang et al. (2021a) |
| *D. gardeniae* | CBS 288.56 | KC343113 | KC344081 | KC343597 | KC343839 | KC343355 | Gomes et al. (2013) |
| *D. garethjonesii* | MFLUCC 12-0542a^T^ | KT459423 | KT459441 | - | KT459457 | KT459470 | Hyde et al. (2016) |
| *D. glabrae* | SCHM 3622^T^ | AY601918 | - | - | - | - | Chang et al. (2005) |
| *D. goulteri* | BRIP 55657a^T^ | KJ197290 | KJ197270 | - | KJ197252 | - | Thompson et al. (2015) |
| *D. grandiflori* | SAUCC194.84^T^ | MT822612 | MT855809 | MT85558 | MT855924 | MT855691 | Sun et al. (2021) |
| *D. guangdongensis* | ZHKUCC 20-0014^T^ | MT355684 | MT409292 | - | MT409338 | MT409314 | Dong et al. (2021) |
| *D. guangxiensis* | JZB 320094^T^ | MK335772 | MK500168 | - | MK523566 | MK736727 | Manawasinghe et al. (2019) |
| *D. gulyae* | BRIP 54025^T^ | JF431299 | KJ197271 | - | JN645803 | - | Thompson et al. (2015) |
| *D. guttulata* | CGMCC 3.20100^T^ | MT385950 | MT424705 | MW022491 | MT424685 | MW022470 | Dissanayake et al. (2020) |
| *D. helianthi* | CBS 592.81^T^ | KC343115 | KC344083 | KC343599 | KC343841 | JX197454 | Gomes et al. (2013) |
| *D. helicis* | CBS 138596^T^ | KJ210538 | KJ420828 | KJ420875 | KJ210559 | KJ435043 | Udayanga et al. (2014a) |
| *D. heliconiae* | SAUCC 194.77^T^ | MT822605 | MT855802 | MT855573 | MT855917 | MT855684 | Sun et al. (2021) |
| *D. heterophyllae* | CBS 143769^T^ | MG600222 | MG600226 | MG600220 | MG600224 | MG600218 | Marin-Felix et al. (2019) |
| *D. heterostemmatis* | SAUCC 194.85^T^ | MT822613 | MT855810 | MT855581 | MT855925 | MT855692 | Sun et al. (2021) |
| *D. hickoriae* | CBS 145.26^T^ | KC343118 | KC344086 | KC343602 | KC343844 | KC343360 | Gomes et al. (2013) |
| *D. hispaniae* | CBS 143351^T^ | MG281123 | MG281296 | MG281471 | MG281644 | MG281820 | Guarnaccia et al. (2018) |
| *D. hongkongensis* | CBS 115448^T^ | KC343119 | KC344087 | KC343603 | KC343845 | KC343361 | Gomes et al. (2013) |
| *D. hordei* | CBS 481.92 | KC343120 | KC344088 | KC343604 | KC343846 | KC343362 | Gomes et al. (2013) |
| *D. hsinchuensis* | NTUPPMCC 18-153-1^T^ | MZ268409 | MZ268430 | MZ268493 | MZ268472 | MZ268451 | Ariyawansa et al. (2021) |
| *D. huangshanensis* | CNUCC 201903^T^ | MN219730 | MN227011 | MN224558 | MN224678 | - | Zhou and Hou (2019) |
| *D. hubeiensis* | JZB 320123^T^ | MK335809 | MK500148 | - | MK523570 | MK500235 | Manawasinghe et al. 2019 |
| *D. humulicola* | CT2018-1^T^ | MN152927 | - | MN180213 | MN180207 | MN180204 | Allan-Perkins et al. (2020) |
| *D. hunanensis* | HNZZ 023^T^ | MZ509550 | MZ504713 | MZ504691 | MZ504702 | MZ504680 | Yang et al. (2021b) |
| *D. hungariae* | CBS 143353^T^ | MG281126 | MG281299 | MG281474 | MG281647 | MG281823 | Guarnaccia *et al*. (2018) |
| *D. ilicicola* | FPH 2015502^T^ | MH171064 | MH171074 | MH171084 | - | - | Lin et al. (2018) |
| *D. impulsa* | CBS 114434 | KC343121 | KC344089 | KC343605 | KC343847 | KC343363 | Gomes et al. (2013) |
| *D. incompleta* | CGMCC 3.18288^T^ | KX986794 | KX999226 | KX999265 | KX999186 | KX999289 | Gao et al. (2017) |
| *D. inconspicua* | CBS 133813^T^ | KC343123 | KC344091 | KC343607 | KC343849 | KC343365 | Gomes et al. (2013) |
| *D. infecunda* | CBS 133812^T^ | KC343126 | KC344094 | KC343610 | KC343852 | KC343368 | Gomes et al. (2013) |
| *D. infertilis* | CBS 230.52^T^ | KC343052 | KC344020 | KC343536 | KC343778 | KC343294 | Guarnaccia & Crous (2017) |
| *D. irregularis* | CGMCC 3.20092^T^ | MT385951 | MT424706 | - | MT424686 | MT424721 | Dissanayake et al. (2020) |
| *D. isoberliniae* | CBS 137981^T^ | KJ869133 | KJ869245 | - | - | - | Crous et al. (2014a) |
| *D. italiana* | MFLUCC 18-0090^T^ | MH846237 | MH853688 | - | MH853686 | MH853690 | Hyde et al. (2019) |
| *D. jinxiu* | CGMCC3.20269^T^ | MW477881 | MW480877 | MW480865 | MW480873 | MW480869 | Wang et al. (2021) |
| *D. juglandicola* | CFCC 51134^T^ | KU985101 | KX024634 | - | KX024628 | KX024616 | Yang et al. (2017a) |
| *D. kadsurae* | CFCC 52586^T^ | MH121521 | MH121600 | MH121479 | MH121563 | MH121439 | Yang et al. (2018) |
| *D. kochmanii* | BRIP 54033^T^ | JF431295 | - | - | JN645809 | - | Thompson et al. (2011) |
| *D. kongii* | BRIP 54031^T^ | JF431301 | KJ197272 | - | JN645797 | - | Thompson et al. (2011) |
| *D. krabiensis* | MFLUCC 17-2481^T^ | MN047101 | MN431495 | - | MN433215 |  | Dayarathne et al. (2020) |
| *D. lenispora* | CGMCC 3.20101^T^ | MT385952 | MT424707 | MW022493 | MT424687 | MW022472 | Dissanayake et al. (2020) |
| *D. leucospermi* | CBS 111980^T^ | JN712460 | KY435673 | KY435653 | KY435632 | KY435663 | Crous et al. (2011) |
| *D. limonicola* | CBS 142549^T^ | MF418422 | MF418582 | MF418342 | MF418501 | MF418256 | Guarnaccia & Crous (2017) |
| *D. liquidambari* | SCHM 3621^T^ | AY601919 | - | - | - | - | Chang et al. (2005) |
| *D. litchicola* | BRIP 54900^T^ | JX862533 | KF170925 | - | JX862539 | - | Tan et al. (2013) |
| *D. litchii* | SAUCC 194.22^T^ | MT822550 | MT855747 | MT855519 | MT855863 | MT855635 | Sun et al. (2021) |
| *D. lithocarpus* | CGMCC 3.15175^T^ | KC153104 | KF576311 | - | KC153095 | - | Gao et al. (2014) |
| *D. litoricola* | MFLUCC 16-1195^T^ | MF190139 | - | - | - | - | Senanayake *et al*. (2017) |
| *D. longicicola* | CGMCC 3.17089^T^ | KF576267 | KF576291 | - | KF576242 | - | Gao et al. (2015) |
| *D. longicolla* | FAU 599^T^ | KJ590728 | KJ610883 | KJ659188 | KJ590767 | KJ612124 | Udayanga et al. (2015) |
| *D. longispora* | CBS 194.36^T^ | KC343135 | KC344103 | KC343619 | KC343861 | KC343377 | Gomes et al. (2013) |
| *D. lonicerae* | MFLUCC 17-0963^T^ | KY964190 | KY964073 | - | KY964146 | KY964116 | Dissanayake et al. (2017a) |
| *D. lusitanicae* | CBS 123212^T^ | KC343136 | KC344104 | KC343620 | KC343862 | KC343378 | Gomes et al. (2013) |
| *D. lutescens* | SAUCC 194.36^T^ | MT822564 | MT855761 | MT855533 | MT855877 | MT855647 | Sun et al. (2021) |
| *D. macadamiae* | BRIP 66526^T^ | MN708230 | MN696539 | - | MN696528 | - | Wrona et al. (2020) |
| *D. machili* | SAUCC 194.111^T^ | MT822639 | MT855836 | MT855606 | MT855951 | MT855718 | Huang et al. (2021) |
| *D. macintoshii* | BRIP 55064a^T^ | KJ197289 | KJ197269 | - | KJ197251 | - | Thompson et al. (2015) |
| *D. mahothocarpi* | CGMCC 3.15181 | KC153096 | - | - | KC153087 | - | Gao et al. (2014) |
| *D. malorum* | CBS142383^T^ | KY435638 | KY435668 | KY435648 | KY435627 | KY435658 | Santos et al. (2017) |
| *D. manihotia* | CBS 505.76 | KC343138 | KC344106 | KC343622 | KC343864 | KC343380 | Gomes et al. (2013) |
| *D. marina* | MFLU 17-2622^T^ | MN047102 | - | - | - | - | Dayarathne et al. (2020) |
| *D. maritima* | DAOMC 250563^T^ | KU552025 | KU574615 | - | KU552023 | - | Tanney et al. (2016) |
| *D. masirevicii* | BRIP 57892a^T^ | KJ197277 | KJ197257 | - | KJ197239 | - | Thompson et al. (2015) |
| *D. mayteni* | CBS 133185^T^ | KC343139 | KC344107 | KC343623 | KC343865 | KC343381 | Gomes et al. (2013) |
| *D. maytenicola* | CBS 136441^T^ | KF777157 | KF777250 | - | - | - | Crous et al. (2013) |
| *D. mediterranea* | DAL-34 | MT007489 | MT006686 | MT007095 | MT006989 | MT006761 | Beluzán et al. (2021) |
| *D. megalospora* | CBS 143.27 | KC343140 | KC344108 | KC343624 | KC343866 | KC343382 | Gomes et al. (2013) |
| *D. melastomatis* | SAUCC 194.55^T^ | MT822583 | MT855780 | MT855551 | MT855896 | MT855664 | Sun et al. (2021) |
| *D. melitensis* | CBS 142551^T^ | MF418424 | MF418584 | MF418344 | MF418503 | MF418258 | Guarnaccia & Crous (2017) |
| *D. melonis* | CBS 507.78^T^ | KC343142 | KC344110 | KC343626 | KC343868 | KC343384 | Gomes et al. (2013) |
| *D. micheliae* | SCHM 3603 | AY620820 | - | - | - | - | Chang et al. (2005) |
| *D. middletonii* | BRIP 54884e^T^ | KJ197286 | KJ197266 | - | KJ197248 | - | Thompson et al. (2015) |
| *D. millettiae* | GUCC 9167^T^ | MK398674 | MK502089 | - | MK480609 | MK502086 | Long et al. (2019) |
| *D. minima* | CGMCC 3.20097^T^ | MT385953 | MT424708 | MW022496 | MT424688 | MT424722 | Dissanayake et al. (2020) |
| *D. minusculata* | CGMCC 3.20098^T^ | MT385957 | MT424712 | MW022499 | MT424692 | MW022475 | Dissanayake et al. (2020) |
| *D. momicola* | MFLUCC 16-0113^T^ | KU557563 | KU557587 | - | KU557631 | KU557611 | Dissanayake et al. (2017b) |
| *D. multigutullata* | ICMP 20656^T^ | KJ490633 | KJ490454 | KJ490575 | KJ490512 | - | Huang et al. (2015) |
| *D. musigena* | CBS 129519^T^ | KC343143 | KC344111 | KC343627 | KC343869 | KC343385 | Gomes et al. (2013) |
| *D. myracrodruonis* | URM 7972^T^ | MK205289 | MK205291 | - | MK213408 | MK205290 | da Silva et al. (2019) |
| *D. nebulae* | PMM 1681^T^ | KY511337 | KY511369 | - | MH708552 | - | Lesuthu et al. (2019) |
| *D. neilliae* | CBS 144.27^T^ | KC343144 | KC344112 | KC343628 | KC343870 | KC343386 | Gomes et al. (2013) |
| *D. neoarctii* | CBS 109490 | KC343145 | KC344113 | KC343629 | KC343871 | KC343387 | Gomes et al. (2013) |
| *D. neoraonikayaporum* | MFLUCC 14-1136^T^ | KU712449 | KU743988 | - | KU749369 | KU749356 | Doilom et al. (2017) |
| *D. nigra* | JZB 320170^T^ | MN653009 | MN887113 | - | MN892277 | - | Hyde et al. (2020) |
| *D. nobilis* | CBS 587.79 | KC343153 | KC344121 | KC343637 | KC343879 | KC343395 | Gomes et al. (2013) |
| *D. nomurai* | CBS 157.29 | KC343154 | KC344122 | KC343638 | KC343880 | KC343396 | Gomes et al. (2013) |
| *D. nothofagi* | BRIP 54801^T^ | JX862530 | KF170922 | - | JX862536 | - | Tan et al. (2013) |
| *D. novem* | CBS 127271^T^ | KC343157 | KC344125 | KC343641 | KC343883 | KC343399 | Gomes et al. (2013) |
| *D. obtusifoliae* | CBS 143449^T^ | MG386072 | - | MG386137 | - | - | Crous et al. (2017) |
| *D. ocoteae* | CBS 141330^T^ | KX228293 | KX228388 | - | - | - | Crous et al. (2016b) |
| *D. oculi* | HHUF 30565^T^ | LC373515 | LC373519 | - | LC373517 | - | Ozawa et al. (2019) |
| *D. oncostoma* | CBS 589.78 | KC343162 | KC344130 | KC343646 | KC343888 | KC343404 | Gomes et al. (2013) |
| *D. oraccinii* | LC 3166^T^ | KP267863 | KP293443 | KP293517 | KP267937 | - | Gao et al. (2016) |
| *D. osmanthi* | GUCC 9165^T^ | MK398675 | MK502090 | - | MK480610 | MK502087 | Long et al. (2019) |
| *D. ovalispora* | ICMP 20659^T^ | KJ490628 | KJ490449 | KJ490570 | KJ490507 | - | Huang et al. (2015) |
| *D. ovoicicola* | CGMCC 3.17092^T^ | KF576264 | KF576288 | - | KF576239 | KF576222 | Gao et al. (2015) |
| *D. oxe* | CBS 133186^T^ | KC343164 | KC344132 | KC343648 | KC343890 | KC343406 | Gomes et al. (2013) |
| *D. pachirae* | COAD 2074^T^ | MG559537 | MG559541 | - | MG559539 | MG559535 | Milagres et al. (2018) |
| *D. padi* var. *padi* | CBS 114200 | KC343169 | KC344137 | KC343653 | KC343895 | KC343411 | Gomes et al. (2013) |
| *D. padina* | CFCC 52590^T^ | MH121525 | MH121604 | MH121483 | MH121567 | MH121443 | Yang et al. (2018) |
| *D. pandanicola* | MFLUCC 17-0607^T^ | MG646974 | MG646930 | - | - | - | Tibpromma et al. (2018) |
| *D. paranensis* | CBS 133184 | KC343171 | KC344139 | KC343655 | KC343897 | KC343413 | Gomes et al. (2013) |
| *D. parapterocarpi* | CBS 137986^T^ | KJ869138 | KJ869248 | - | - | - | Crous et al. (2014a) |
| *D. parvae* | PSCG 034^T^ | MK626919 | MK691248 | MK726210 | MK654858 | - | Guo et al. (2020) |
| *D. pascoei* | BRIP 54847^T^ | JX862532 | KF170924 | - | JX862538 | - | Tan et al. (2013) |
| *D. passiflorae* | CBS 132527^T^ | JX069860 | KY435674 | KY435654 | KY435633 | KY435664 | Crous et al. (2012) |
| *D. passifloricola* | CBS 141329^T^ | KX228292 | KX228387 | KX228367 | - | - | Crous et al. (2016b) |
| *D. patagonica* | CBS 145291^T^ | MN509717 | MN509728 | - | MN509739 | MN974279 | Zapata et al. (2020) |
| *D. penetriteum* | LC 3353 | KP714505 | KP714529 | KP714493 | KP714517 | - | Gao et al. (2016) |
| *D. perjuncta* | CBS 109745^T^ | KC343172 | KC344140 | KC343656 | KC343898 | KC343414 | Gomes et al. (2013) |
| *D. perniciosa* | CBS 124030 | KC343149 | KC344117 | KC343633 | KC343875 | KC343391 | Gomes et al. (2013) |
| *D. perseae* | CBS 151.73 | KC343173 | KC344141 | KC343657 | KC343899 | KC343415 | Gomes et al. (2013) |
| *D. pescicola* | MFLUCC 16-0105^T^ | KU557555 | KU557579 | - | KU557623 | KU557603 | Dissanayake et al. (2017b) |
| *D. phaseolorum* | CBS 113425 | KC343174 | KC344142 | KC343658 | KC343900 | KC343416 | Gomes et al. (2013) |
| *D. phragmitis* | CBS 138897^T^ | KP004445 | KP004507 | KP004503 | - | - | Crous et al. (2014b) |
| *D. phillipsii* | CAA 817^T^ | MK792305 | MN000351 | MK871445 | MK828076 | MK883831 | Hilário et al. (2020) |
| *D. phyllanthicola* | SCHM 3680^T^ | AY620819 | - | - | - | - | Chang et al. (2005) |
| *D. podocarpi-macrophylli* | CGMCC 3.18281^T^ | KX986774 | KX999207 | KX999246 | KX999167 | KX999278 | Gao et al. (2017) |
| *D. poincianellae* | URM 7932^T^ | MH989509 | MH989537 | MH989539 | MH989538 | MH989540 | Crous et al. (2018a) |
| *D. pometiae* | SAUCC 194.72^T^ | MT822600 | MT855797 | MT855568 | MT855912 | MT855679 | Huang et al. (2021) |
| *D. portugallica* | CBS 144228^T^ | MH063905 | MH063917 | MH063899 | MH063911 | MH063893 | Guarnaccia & Crous (2018) |
| *D. pseudoalnea* | CFCC 54190^T^ | MZ727037 | MZ753487 | MZ781302 | MZ816343 | MZ753468 | Jiang et al. (2021) |
| *D. pseudoinconspicua* | URM 7874^T^ | MH122538 | MH122524 | MH122517 | MH122533 | MH122528 | Crous et al. (2018b) |
| *D. pseudolongicolla* | CBS 117165^T^ | DQ286285 | - | - | DQ286259 | - | Petrović et al. (2018) |
| *D. pseudomangiferae* | CBS 101339^T^ | KC343181 | KC344149 | KC343665 | KC343907 | KC343423 | Gomes et al. (2013) |
| *D. pseudooculi* | HHUF 30617^T^ | LC373515 | LC373519 | - | LC373517 | - | Ozawa et al. (2019) |
| *D. pseudophoenicicola* | CBS 462.69^T^ | KC343184 | KC344152 | KC343668 | KC343910 | KC343426 | Gomes et al. (2013) |
| *D. pseudotsugae* | MFLU 15-3228^T^ | KY964225 | KY964108 | - | KY964181 | KY964138 | Dissanayake et al. (2017a) |
| *D. psoraleae* | CBS 136412^T^ | KF777158 | KF777251 | - | KF777245 | - | Crous et al. (2013) |
| *D. psoraleae-pinnatae* | CBS 136413^T^ | KF777159 | KF777252 | - | - | - | Crous et al. (2013) |
| *D. pterocarpi* | MFLUCC 10-0571 | JQ619899 | JX275460 | - | JX275416 | JX197451 | Udayanga et al. (2012) |
| *D. pterocarpicola* | MFLUCC 10-0580a | JQ619887 | JX275441 | - | JX275403 | JX197433 | Udayanga et al. (2012) |
| *D. pulla* | CBS 338.89^T^ | KC343152 | KC344120 | KC343636 | KC343878 | KC343394 | Gomes et al. (2013) |
| *D. pungensis* | SAUCC 194.112^T^ | MT822640 | MT855837 | MT855607 | MT855952 | MT855719 | Sun et al. (2021) |
| *D. pustulata* | CBS 109742 | KC343185 | KC344153 | KC343669 | KC343911 | KC343427 | Gomes et al. (2013) |
| *D. pyracanthae* | CBS142384^T^ | KY435635 | KY435666 | KY435645 | KY435625 | KY435656 | Santos et al. (2017) |
| *D. racemosae* | CBS 143770^T^ | MG600223 | MG600227 | MG600221 | MG600225 | MG600219 | Marin-Felix et al. (2019) |
| *D. raonikayaporum* | CBS 133182^T^ | KC343188 | KC344156 | KC343672 | KC343914 | KC343430 | Gomes et al. (2013) |
| *D. ravennica* | MFLUCC 15–0479^T^ | KU900335 | KX432254 | - | KX365197 | - | Dissanayake et al. (2017a) |
| *D. rhoina* | CBS 146.27 | KC343189 | KC344157 | KC343673 | KC343915 | KC343431 | Gomes et al. (2013) |
| *D. rhusicola* | CBS 129528^T^ | JF951146 | KC843205 | - | KC843100 | KC843124 | Crous et al. (2011a), Udayanga et al. (2014b) |
| *D. rosae* | MFLUCC 17-2658^T^ | MG828894 | MG843878 | - | - | MG829273 | Wanasinghe et al. (2018) |
| *D. rosicola* | MFLU 17-0646^T^ | MG828895 | MG843877 | - | MG829270 | MG829274 | Wanasinghe et al. (2018) |
| *D. rosiphthora* | COAD 2913^T^ | MT311196 | - | - | MT313692 | MT313690 | Pereira et al. (2021) |
| *D. rossmaniae* | CAA 762^T^ | MK792290 | MK837914 | MK871432 | MK828063 | MK883822 | Hilário et al. (2020) |
| *D. rostrata* | CFCC 50062^T^ | KP208847 | KP208855 | KP208851 | KP208853 | KP208849 | Fan et al. (2015) |
| *D. rudis* | CBS 113201 | KC343234 | KC344202 | KC343718 | KC343960 | KC343476 | Udayanga et al. (2014b) |
| *D. rumicicola* | MFLUCC 18-0739^T^ | MH84623 | MK049555 | - | MK049554 | - | Hyde et al. (2019) |
| *D. saccarata* | CBS 116311^T^ | KC343190 | KC344158 | KC343674 | KC343916 | KC343432 | Gomes et al. (2013) |
| *D. sackstonii* | BRIP 54669b^T^ | KJ197287 | KJ197267 | - | KJ197249 | - | Thompson et al. (2015) |
| *D. salicicola* | BRIP 54825^T^ | JX862531 | KF170923 | - | JX862537 | - | Tan et al. (2013) |
| *D. salinicola* | MFLU 18-0553^T^ | MN047098 | - | - | MN077073 | - | Dayarathne et al. (2020) |
| *D. sambucusii* | CFCC 51986^T^ | KY852495 | KY852511 | KY852503 | KY852507 | KY852499 | Yang *et al*. (2018) |
| *D. schimae* | CFCC 53103^T^ | MK432640 | MK578043 | MK442987 | MK578116 | MK442962 | Yang et al. (2021a) |
| *D. schini* | CBS 133181^T^ | KC343191 | KC344159 | KC343675 | KC343917 | KC343433 | Gomes et al. (2013) |
| *D. schisandrae* | CFCC 51988^T^ | KY852497 | KY852513 | KY852505 | KY852509 | KY852501 | Yang *et al*. (2018) |
| *D. schoeni* | MFLU 15-1279^T^ | KY964226 | KY964109 | - | KY964182 | KY964139 | Dissanayake et al. (2017a) |
| *D. sclerotioides* | CBS 296.67^T^ | KC343193 | KC344161 | KC343677 | KC343919 | KC343435 | Gomes et al. (2013) |
| *D. scobina* | CBS 251.38 | KC343195 | KC344163 | KC343679 | KC343921 | KC343437 | Gomes et al. (2013) |
| *D. searlei* | BRIP 66528^T^ | MN708231 | MN696540 | - | - | - | Wrona et al. (2020) |
| *D. sennae* | CFCC 51636^T^ | KY203724 | KY228891 | - | KY228885 | KY228875 | Yang et al. (2017c) |
| *D. sennicola* | CFCC 51634^T^ | KY203722 | KY228889 | - | KY228883 | KY228873 | Yang et al. (2017c) |
| *D. serafiniae* | BRIP 55665a^T^ | KJ197274 | KJ197254 | - | KJ197236 | - | Thompson et al. (2015) |
| *D. shaanxiensis* | CFCC 53106 | MK432654 | - | MK443001 | MK578130 | MK442976 | Yang et al. (2020) |
| *D. shennongjiaensis* | CNUCC201905^T^ | MN216229 | MN227012 | MN224559 | MN224672 | MN224551 | Zhou and Hou (2019) |
| *D. siamensis* | MFLUCC 10-0573a | JQ619879 | JX275429 | - | JX275393 | - | Udayanga et al. (2012) |
| *D. silvicola* | CFCC 54191^T^ | MZ727041 | MZ753491 | MZ753481 | MZ816347 | MZ753472 | Jiang et al. (2021) |
| *D. sinensis* | CGMCC 3.19521^T^ | MK637451 | MK660447 | - | MK660449 | - | Feng et al. (2019) |
| *D. sojae* | CBS 139282^T^ | KJ590719 | KJ610875 | KJ659208 | KJ590762 | KJ612116 | Udayanga et al. (2015) |
| *D. spartinicola* | CBS 140003^T^ | KR611879 | KR857695 | KR857696 | - | - | Crous et al. (2015a) |
| *D. spinosa* | PSCG 383^T^ | MK626849 | MK691234 | MK726156 | MK654811 | MK691129 | Guo et al. (2020) |
| *D. sterilis* | CBS 136969^T^ | KJ160579 | KJ160528 | MF418350 | KJ160611 | KJ160548 | Lombard et al. (2014) |
| *D. stewartii* | CBS 193.36 | FJ889448 | - | - | GQ250324 | - | Santos et al. (2010) |
| *D. stictica* | CBS 370.54 | KC343212 | KC344180 | KC343696 | KC343938 | KC343454 | Gomes et al. (2013) |
| *D. subclavata* | ICMP 20663^T^ | KJ490630 | KJ490451 | KJ490572 | KJ490509 | - | Huang et al. (2015) |
| *D. subcylindrospora* | KUMCC 17-0151^T^ | MG746629 | MG746631 | - | MG746630 | - | Hyde et al. (2018) |
| *D. subellipicola* | KUMCC 17-0153^T^ | MG746632 | MG746634 | - | MG746633 | - | Hyde et al. (2018) |
| *D. subordinaria* | CBS 101711 | KC343213 | KC344181 | KC343697 | KC343939 | KC343455 | Gomes et al. (2013) |
| *D. taoicola* | MFLUCC 16-0117^T^ | KU557567 | KU557591 | - | KU557635 | - | Dissanayake et al. (2017b) |
| *D. tarchonanthi* | CBS 146073^T^ | MT223794 | MT223733 | MT223759 | - | - | Crous et al. (2020) |
| *D. tecomae* | CBS 100547 | KC343215 | KC344183 | KC343699 | KC343941 | KC343457 | Gomes et al.(2013) |
| *D. tectonae* | MFLUCC 12-0777^T^ | KU712430 | KU743977 | - | KU749359 | KU749345 | Doilom et al. (2017) |
| *D. tectonendophytica* | MFLUCC 13-0471^T^ | KU712439 | KU743986 | - | KU749367 | KU749354 | Doilom et al. (2017) |
| *D. tectonigena* | MFLUCC 12-0767^T^ | KU712429 | KU743976 | - | KU749371 | KU749358 | Doilom et al. (2017) |
| *D. terebinthifolii* | CBS 133180^T^ | KC343216 | KC344184 | KC343700 | KC343942 | KC343458 | Gomes et al. (2013) |
| *D. ternstroemia* | CGMCC 3.15183^T^ | KC153098 | - | - | KC153089 | - | Gao et al. (2014) |
| *D. thunbergii* | MFLUCC 10-0756a | JQ619893 | JX275449 | - | JX275409 | JX197440 | Udayanga et al. (2012) |
| *D. thunbergiicola* | MFLUCC 12-0033^T^ | KP715097 | - | - | KP715098 | - | Liu et al. (2015) |
| *D. tibetensis* | CFCC 51999^T^ | MF279843 | MF279873 | MF279828 | MF279858 | MF279888 | Fan et al. (2018) |
| *D. torilicola* | MFLUCC 17-1051^T^ | KY964212 | KY964096 | - | KY964168 | KY964127 | Dissanayake et al. (2017a) |
| *D. toxica* | CBS 534.93^T^ | KC343220 | KC344188 | KC343704 | KC343946 | KC343462 | Gomes et al.(2013) |
| *D. toxicodendri* | FFPRI 420987 | LC275192 | LC275224 | LC275216 | LC275216 | LC275200 | Ando et al. (2017) |
| *D. tulliensis* | BRIP 62248a | KR936130 | KR936132 | - | KR936133 | - | Crous et al. (2015b) |
| *D. ueckeri* | FAU 656 | KJ590726 | KJ610881 | KJ659215 | KJ590747 | KJ612122 | Huang et al. (2015) |
|  | BRIP 54736j (type of *D. miriciae*) | KJ197283 | KJ197263 | - | KJ197245 | - | Thompson et al. (2015) |
| *D. ukurunduensis* | CFCC 52592^T^ | MH121527 | - | MH121485 | MH121569 | MH121445 | Yang et al. (2018) |
| *D. undulata* | CGMCC 3.18293^T^ | KX986798 | KX999230 | KX999269 | KX999190 | - | Gao et al. (2017) |
| *D. unshiuensis* | CGMCC3.17569^T^ | KJ490587 | KJ490408 | KJ490529 | KJ490466 | - | Huang et al. (2015) |
| *D. vaccinii* | CBS 160.32^T^ | AF317578 | KC344196 | KC343712 | GQ250326 | KC343470 | Gomes et al. (2013) |
| *D. vacuae* | CAA 830^T^ | MK792309 | MK837931 | MK871449 | MK828080 | MK883834 | Hilário et al. (2020) |
| *D. vangueriae* | CBS 137985^T^ | KJ869137 | KJ869247 | - | - | - | Crous et al. (2014a) |
| *D. vawdreyi* | BRIP 57887a | KR936126 | KR936128 | - | KR936129 | - | Crous et al. (2015b) |
| *D. velutina* | CGMCC 3.18286^T^ | KX986790 | KX999223 | KX999261 | KX999182 | - | Gao et al. (2017) |
| *D. verniciicola* | CFCC 53109^T^ | MK573944 | MK574639 | MK574599 | MK574619 | MK574583 | Yang et al. (2021a) |
| *D. vexans* | CBS 127.14 | KC343229 | KC344197 | KC343713 | KC343955 | KC343471 | Gomes et al.(2013) |
| *D. viniferae* | JZB 320071^T^ | MK341551 | MK500112 | - | MK500107 | MK500119 | Manawasinghe et al. 2019 |
| *D. virgiliae* | CBS 138788^T^ | KP247573 | KP247582 | - | - | - | Machingambi et al. (2015) |
| *D. vitimegaspora* | STE-U 2675 | AF230749 | - | - | - | - | Mostert et al. (2001) |
| *D. vochysiae* | LGMF 1583^T^ | MG976391 | MK007527 | MK033323 | MK007526 | MK007528 | Noriler et al. (2019) |
| *D. woodii* | CBS 558.93 | KC343244 | KC344212 | KC343728 | KC343970 | KC343486 | Gomes et al. (2013) |
| *D. woolworthii* | CBS 148.27 | KC343245 | KC344213 | KC343729 | KC343971 | KC343487 | Gomes et al. (2013) |
| *D. xishuangbanica* | CGMCC 3.18282^T^ | KX986783 | KX999216 | KX999255 | KX999175 | - | Gao et al. (2017) |
| *D. xunwuensis* | CFCC 53085^T^ | MK432663 | MK578063 | MK443008 | MK578137 | MK442983 | Yang et al. (2021a) |
| *D. yunnanensis* | CGMCC 3.18289^T^ | KX986796 | KX999228 | KX999267 | KX999188 | KX999290 | Gao et al. (2017) |
| *D. zaobaisu* | PSCG 031^T^ | MK626922 | MK691245 | MK726207 | MK654855 | - | Guo et al. (2020) |
| *D. zaofenghuang* | CGMCC3.20271^T^ | MW477883 | MW480875 | - | MW480871 | MW480867 | Wang et al. (2021) |
| *Diaporthella corylina* | CBS 121124 | KC343004 | KC343972 | KC343488 | KC343730 | KC343246 | Gomes et al. (2013) |

^1^ATCC: American Type Culture Collection, Virginia, USA; BRIP: Queensland Plant Pathology Herbarium, Brisbane, Australia; CAA: Collection of Artur Alves housed at Department of Biology, University of Aveiro, Portugal; CBS: Westerdijk Fungal Biodiversity Institute, Utrecht, the Netherlands; CFCC: China Forestry Culture Collection Center, Beijing, China; CGMCC: Chinese General Microbiological Culture Collection Center, Beijing, China; CMRP: Taxonline Microbiological Collections of Paraná Network, at the Federal University of Paraná, Brazil; CNUCC: Capital Normal University Culture Collection Center, Beijing, China; COAD: Culture Collection of Octávio de Almeida Drumond. Universidade Federal de Viçosa, Viçosa, Brasil; CPC: Culture collection of Pedro Crous, housed at Westerdijk Fungal Biodiversity Institute; DAOM: Plant Research Institute, Department of Agriculture (Mycology), Ottawa, Canada; DAL: strains deposited in fungal collection of the Instituto Agroforestal Mediterráneo–Universitat Politècnica de València, Valencia, Spain; DAOMC: Canadian Collection of Fungal Cultures, Ottawa, Canada; FPH: personal collection of Francesca Peduto Hand, Department of Plant Pathology, The Ohio State University, Columbus; GUCC: Culture Collection at the Department of Plant Pathology, Agriculture College, Guizhou University, China; FAU: Isolates in culture collection of Systematic Mycology and Microbiology Laboratory; FFPRI: the Forestry and Forest Products Research Institute culture collection, Tsukuba, Japan; HNZZ: Central South University of Forestry and Technology, Changsha, China; ICMP: International Collection of Micro-organisms from Plants, Landcare Research, Private Bag 92170, Auckland, New Zealand; IFRDCC: International Fungal Research and Development Culture Collection; KUMCC: Kumming Institute of Botany, Kumming, China; JZB: Culture collection of Institute of Plant and Environment Protection, Beijing, China; LC: Working collection of Lei Cai, housed at Institute of Microbiology, Chinese Academy of Sciences, Beijing, China; LGMF, Laboratório de Genética de Microrganismos (LabGeM) culture collection, at the Federal University of Paraná, Brazil; MAFF: Ministry of Agriculture, Forestry and Fisheries, Tokyo, Japan; MFLU: Mae Fah Luang University herbarium, Thailand; MFLUCC: Mae Fah Luang University Culture Collection, Chiang Rai, Thailand; NCYU: Department of Plant Medicine, National Chiayi University, Chiayi, Taiwan; NTUPPMCC: Department of Plant Pathology and Microbiology, National Taiwan University Culture Collection,

PMM: collection of Providence Moyo at the University of Stellenbosch, Stellenbosch, South Africa; PSCG: Personal Culture Collection Y.S. Guo, China; SAUCC: Shandong Agricultural University Culture Collection, Shandong, China; SCHM: Mycological Herbarium of South China Agricultural University, Guangzhou, China; URM: Culture Collection at the Universidade Federal de Pernambuco, Recife, Brazil; VTCC: Vietnam Type Culture Collection, Center of Biotechnology, Vietnam National University, Hanoi, Vietnam; ZHKUCC: Culture Collection of Zhongkai University of Agriculture and Engineering, Guangzhou, China. ^T^ indicates ex-type material.

^2^ITS: internal transcribed spacers and intervening 5.8S nrDNA; *tub2*: partial β-tubulin gene; *his3*: partial histone H3 gene; *tef1*: partial elongation factor 1-alpha gene; *cal*: partial calmodulin gene.

# Alignment of the ITS sequences used in the second phylogenetic study

>Diaporthe_acaciarum_CBS_138862

NNNNNNNNNCCGTTGGTGAACCAGCGGAGGGATCATTGCTGGAACGCG-CTTC-GGCGCA-CCCAGAAACCCTTTGTGAACTTATACCT--ACTGTTGCCTCGGCGTCAGGCCGGCCTCC--TC----------ACCGAGGCC-----CCCCG---GAGACGGGGAGCA-GCCCGCCGGCGGCCAA-CTAAACTCTTGTTTCTACAGTGAATCTCTGAGT--AAAAAACATAAATGAATCAAAACTTTCAACAACGGATCTCTTGGTTCTGGCATCGATGAAGAACGCAGCGAAATGCGATAAGTAATGTGAATTGCAGAATTCAGTGAATCATCGAATCTTTGAACGCACATTGCGCCCCCTGGTATTCCGGGGGGCATGCCTGTTCGAGCGTCATTTCAACCCTCAAGCCTGGCTTGG-TGATGGGGCACTACTCCCTC------GCGGGAGTAGGCCCTGAAATTCAGTGGCGAGCT-CGCCAGGAC-CCCGAGCGTAGTAG-TTATA-TCTCGCTTTGG-AAGGCCCTGGCGGTGCCCTGCCGTTAAA-CCCCCAACTTCTGAAAATT

>Diaporthe_middletonii_BRIP_54884e

ACAAGGTCTCCGTTGGTGAACCAGCGGAGGGATCATTGCTGGAACGCG-CTTC-GGCGCA-CCCAGAAACCCTTTGTGAACTTATACCT--ATTGTTGCCTCGGCGTTAGGCCGGCCTCC--TC----------ACTGAGGCC-----CCCTG---GAAACAGGGAGCA-GCCCGCCGGCGGCCAA-CTAAACTCTTGTTTCTATAGTGAATCTCTGAGT--AAAAAACATAAATGAATCAAAACTTTCAACAACGGATCTCTTGGTTCTGGCATCGATGAAGAACGCAGCGAAATGCGATAAGTAATGTGAATTGCAGAATTCAGTGAATCATCGAATCTTTGAACGCACATTGCGCCCCCTGGTATTCCGGGGGGCATGCCTGTTCGAGCGTCATTTCAACCCTCAAGCCTGGCTTGG-TGATGGGGCACTACTTCCTC------ACGGGAGTAGGCCCTGAAATTCAGTGGCGAGCT-CGCCAGGAC-CCCGAGCGTAGTAG-TTATA-TCTCGCTTTGG-AAGGCCCTGGCGGTGCCCTGCCGTTAAA-ACCCCAACTTCTGAAAATT

>Diaporthe_beilharziae_BRIP_54792

ACAAGGTCTCCGTTGGTGAACCAGCGGAGGGATCATTGCTGGAACGCG-CCTC-GGCGCA-CCCAGAAACCCTTTGTGAACTTATACCT--ATCGTTGCCTCGGCGTTCGGCCGGCCTCT--TC----------ACTGAGGCC-----CCCTG---GAAACGGGGAGCA-GCCCGCCGGCGGCCAA-CCAAACTCTTGTTTCTATAGTGAATCTCTGAGT--GAAAAACATAAATGAATCAAAACTTTCAACAACGGATCTCTTGGTTCTGGCATCGATGAAGAACGCAGCGAAATGCGATAAGTAATGTGAATTGCAGAATTCAGTGAATCATCGAATCTTTGAACGCACATTGCGCCCCCTGGTATTCCGGGGGGCATGCCTGTTCGAGCGTCATTTCAACCCTCAAGCCTGGCTTGG-TGATGGGGCACTACTTCCTC------ACGGGAGTAGGCCCTGAAATTCAGTGGCGAGCT-CGCCAGGAC-CCCGAGCGTAGTAG-TTACA-TCTCGCTCTGG-AAGGCCCTGGCGGTGCCCTGCCGTTAAA-CCCCCAACTTCTGAAAATT

>Diaporthe_caryae_CFCC_52563

NNNNNNNNNNNNNNNNNNNNNNNNNNNNNNNNNNNNNNNNNNNNNNNNNNNNNNNNNNNNNNNNNNNNNCCCTTTGTGAACTTATACCT--ACTGTTGCCTCGGCGTCAGGCCGGCCCCT--TC----------ACTGGGGCC-----CCCCG---GAGACGGGGAGCA-GCCCGCCGGCGGCCAA-CTAAACTCTTGTTTCTATAGTGAATCTCTGAGT--AAAAAACATAAATGAATCAAAACTTTCAACAACGGATCTCTTGGTTCTGGCATCGATGAAGAACGCAGCGAAATGCGATAAGTAATGTGAATTGCAGAATTCAGTGAATCATCGAATCTTTGAACGCACATTGCGCCCCCTGGTATTCCGGGGGGCATGCCTGTTCGAGCGTCATTTCAACCCTCAAGCCTGGCTTGG-TGATGGGGCACTGCTTCG--------AGAGGAGCAGGCCCTGAAATTCAGTGGCGAGCT-CGCCAGGAC-CCCGAGCGTAGTAG-TTATA-TCTCGCTTTGG-AAGGCCCTGGCGGTGCCCTGCCGTTAAA-CCCCCAACTTCTGAAANNN

>Phomopsis_chimonanthi

NNNNNNNNNNNNNNNTGTGACCAGCGGAGGGATCATTGCTGGAACGCG-CTTC-GGCGCA-CCCAGAAACCCTTTGTGAACTTATACCT--ATTGTTGCCTCGGCGTCAGGCCGGCCTCT--TC----------ACTGAGGCC-----CCCCG---GAGACGGGGAGCA-GCCCGCCGGCGGCCAA-CTAAACTCTTGTTTCTATAGTGAATCTCTGAGT--AAAAAACATAAATGAATCAAAACTTTCAACAACGGATCTCTTGGTTCTGGCATCGATGAAGAACGCAGCGAAATGCGATAAGTAATGTGAATTGCAGAATTCAGTGAATCATCGAATCTTTGAACGCACATTGCGCCCCCTGGTATTCCGGGGGGCATGCCTGTTCGAGCGTCATTTCAACCCTCAAGCCTGGCTTGG-TGATGGGGCACTGCTTCG--------AAAGGAGCAGGCCCTGAAATTCAGTGGCGAGCT-CGCCAGGAC-CCCGAGCGTAGTAG-TTATA-TCTCGCTTTGG-AAGGCCCTGGCGGTGCCCTGCCGTTAAA-CCCCCAACTTCTGAAAATT

>Phomopsis_micheliae

NNNNNNNNNNNNNNNTGTGACCAGCGGAGGGATCATTGCTGGAACGCG-CTTC-GGCGCA-CCCAGAAACCCTTTGTGAACTTATACCT--ATTGTTGCCTCGGCGTCAGGCCGGCCTCT--TC----------ACTGAGGCC-----CCCCG---GAGACGGGGAGCA-GCCCGCCGGCGGCCAA-CTAAACTCTTGTTTCTATAGTGAATCTCTGAGT--AAAAAACATAAATGAATCAAAACTTTCAACAACGGATCTCTTGGTTCTGGCATCGATGAAGAACGCAGCGAAATGCGATAAGTAATGTGAATTGCAGAATTCAGTGAATCATCGAATCTTTGAACGCACATTGCGCCCCCTGGTATTCCGGGGGGCATGCCTGTTCGAGCGTCATTTCAACCCTCAAGCCTGGCTTGG-TGATGGGGCACTGCTTCG--------AAAGGAGCAGGCCCTGAAATTCAGTGGCGAGCT-CGCCAGGAC-CCCGAGCGTAGTAG-TTATA-TCTCGCTTTGG-AAGGCCCTGGCGGTGCCCTGCCGTTAAA-CCCCCAACTTCTGAAAATT

>Diaporthe_machili_SAUCC194_111

NNNNNNTCTCCGTTGGTGAACCAGCGGAGGGATCATTGCTGGAACGCG-CTTC-GGCGCA-CCCAGAAACCCTTTGTGAACTTATACCT--ACTGTTGCCTCGGCGTCAGGCCGGCCTCC--TC----------ACCGAGGCC-----CCCCG---GAGACGGGGAGCA-GCCCGCCGGCGGCCAA-CTAAACTCTTGTTTCTATAGTGAATCTCTGAGT--AAAAAACATAAATGAATCAAAACTTTCAACAACGGATCTCTTGGTTCTGGCATCGATGAAGAACGCAGCGAAATGCGATAAGTAATGTGAATTGCAGAATTCAGTGAATCATCGAATCTTTGAACGCACATTGCGCCCCCTGGTATTCCGGGGGGCATGCCTGTTCGAGCGTCATTTCAACCCTCAAGCCTGGCTTGG-TGATGGGGCACTGCTTCG--------AGAGGAGCAGGCCCTGAAATTCAGTGGCGAGCT-CGCCAGGAC-CCCGAGCGTAGTAG-TTATA-TCTCGCTCTGG-AAGGCCCTGGCGGTGCCCTGCCGTTAAA-CCCCCAACTTCTGAAAATT

>Diaporthe_sackstonii_BRIP_54669b

ACAAGGTCTCCGTTGGTGAACCAGCGGAGGGATCATTGCTGGAACGCG-CTTC-GGCGCA-CCCAGAAACCCTTTGTGAACTTATACCT--ATTGTTGCCTCGGCGTCAGGCCGGCCTCT--TC----------ACTGAGGCC-----CCCCG---GAGACGGGGAGCA-GCCCGCCGGCGGCCAA-CTAAACTCTTGTTTCTATAGTGAATCTCTGAGT--AAAAAACATAAATGAATCAAAACTTTCAACAACGGATCTCTTGGTTCTGGCATCGATGAAGAACGCAGCGAAATGCGATAAGTAATGTGAATTGCAGAATTCAGTGAATCATCGAATCTTTGAACGCACATTGCGCCCCCTGGTATTCCGGGGGGCATGCCTGTTCGAGCGTCATTTCAACCCTCAAGCCTGGCTTGG-TGATGGGGCACTGCTTCG--------AGAGGAGCAGGCCCTGAAATTCAGTGGCGAGCT-CGCCAGGAC-CCCGAGCGTAGTAG-TTATA-TCTCGCTCTGG-AAGGCCCTGGCGGTGCCCTGCCGTTAAA-CCCCCAACTTCTGAAAATT

>Diaporthe_infecunda_CBS_133812

ACAAGGTCTCCGTTGGTGAACCAGCGGAGGGATCATTGCTGGAACGCG-CTTC-GGCGCA-CCCAGAAACCCTTTGTGAACTTATACCT--ATTGTTGCCTCGGCGTTAGGCCGGCCTCT--TC----------ACCGAGGCC-----CCCTG---GAAACAGGGAGCA-GCCCGCCGGCGGCCAA-CTAAACTCTTGTTTCTATAGTGAATCTCTGAGT--AAAAAACATAAATGAATCAAAACTTTCAACAACGGATCTCTTGGTTCTGGCATCGATGAAGAACGCAGCGAAATGCGATAAGTAATGTGAATTGCAGAATTCAGTGAATCATCGAATCTTTGAACGCACATTGCGCCCCCTGGTATTCCGGGGGGCATGCCTGTTCGAGCGTCATTTCAACCCTCAAGCCTGGCTTGG-TGATGGGGCACTGCTTCG--------AAAGGAGCAGGCCCTGAAATTCAGTGGCGAGCT-CGCCAGGAC-CCCGAGCGTAGTAG-TTATA-TCTCGCTTTGG-AAGGCCCTGGCGGTGCCCTGCCGTTAAA-CCCCCAACTTCTGAAAATT

>Diaporthe_serafiniae_BRIP_55665a

ACAAGGTCTCCGTTGGTGAACCAGCGGAGGGATCATTGCTGGAACGCG-CTTC-GGCGCA-CCCAGAAACCCTTTGTGAACTTATACCT--ATTGTTGCCTCGGCGTTAGGCCGGCCTCT--TC----------ACTGAGGCC-----CCCTG---GAAACGGGGAGCA-GCCCGCCGGCGGCCAA-CTAAACTCTTGTTTCTATAGTGAATCTCTGAGT--AAAAAACATAAATGAATCAAAACTTTCAACAACGGATCTCTTGGTTCTGGCATCGATGAAGAACGCAGCGAAATGCGATAAGTAATGTGAATTGCAGAATTCAGTGAATCATCGAATCTTTGAACGCACATTGCGCCCCCTGGTATTCCGGGGGGCATGCCTGTTCGAGCGTCATTTCAACCCTCAAGCCTGGCTTGG-TGATGGGGCACTGCTTCG--------AGAGGAGCAGGCCCTGAAATTCAGTGGCGAGCT-CGCCAGGAC-CCCGAGCGTAGTAG-TTATA-TCTCGCT-TAA-AGGGCCCTGGCGGTGCCCTGCCGTTAAA-CCCCCAACTNNNNNNNNNN

>Diaporthe_pachirae_CDA_728

NNNNNNNNNNNNNNNNNNNNNNNNCGGAGGGATCATTGCTGGAACGCG-CTTC-GGCGCA-CCCAGAAACCCTTTGTGAACTTATACCT--ATTGTTGCCTCGGCGTCAGGCCGGCCTCT--TC----------ACTGAGGCC-----CCCTG---GAGACAGGGAGCA-GCCCGCCGGCGGCCAA-CTAAACTCTTGTTTCTATAGTGAATCTCTGAGT--AAAAAACATAAATGAATCAAAACTTTCAACAACGGATCTCTTGGTTCTGGCATCGATGAAGAACGCAGCGAAATGCGATAAGTAATGTGAATTGCAGAATTCAGTGAATCATCGAATCTTTGAACGCACATTGCGCCCCCTGGTATTCCGGGGGGCATGCCTGTTCGAGCGTCATTTCAACCCTCAAGCCTGGCTTGG-TGATGGGGCACTACTTCCTC------ACGGGAGTAGGCCCTGAAATTCAGTGGCGAGCT-CGCCAGGAC-CCCGAGCGTAGTAG-TTATA-TCTCGCTTTGG-AAGGCCCTGGCGGTGCCCTGCCGTTAAA-CCCCCAACTTCTGAAAATT

>Diaporthe_leucospermi_CBS_111980

ACAAGGTCTCCGTTGGTGAACCAGCGGAGGGATCATTGCTGGAACGCG-CTTC-GGCGCA-CCCAGAAACCCTTTGTGAACTTATACCT--ACTGTTGCCTCGGCG-TAAGCTGGCTTTG--AA----------ATATAAGCC-----CCCTG---GAAACGGGGAGCA-GCCCGCCGGCGGCCAA-CTAAACTCTTGTTTCTATAGTGAATCTCTGAGT--AAAAAACATAAATGAATCAAAACTTTCAACAACGGATCTCTTGGTTCTGGCATCGATGAAGAACGCAGCGAAATGCGATAAGTAATGTGAATTGCAGAATTCAGTGAATCATCGAATCTTTGAACGCACATTGCGCCCCCTGGTATTCCGGGGGGCATGCCTGTTCGAGCGTCATTTCAACCCTCAAGCCTGGCTTGG-TGATGGGGCACTGCTTCG--------AAAGGAGCAGGCCCTGAAATTCAGTGGCGAGCT-CGCCAGGAC-CCCGAGCGTAGTAG-TTATA-TCTCGCTTTGG-AAGGCCCTGGCGGTGCCCTGCCGTTAAA-CCCCCAACTTCTGAAAATT

>Diaporthe_pyracanthae_CBS142384

NNNNNNNNNNNNNNNNNNNNNNNNNNNAGGGATCATTGCTGGAACGCG-CTTC-GGCGCA-CCCAGAAACCCTTTGTGAACTTATACCT--ATTGTTGCCTCGGCG-TAAGCTGGCTTTG--AA----------ATATAAGCC-----CCCTG---GAAACGGGGAGCA-GCCCGCCGGCGGCCAA-CTAAACTCTTGTTTCTATAGTGAATCTCTGAGT--AAAAAACATAAATGAATCAAAACTTTCAACAACGGATCTCTTGGTTCTGGCATCGATGAAGAACGCAGCGAAATGCGATAAGTAATGTGAATTGCAGAATTCAGTGAATCATCGAATCTTTGAACGCACATTGCGCCCCCTGGTATTCCGGGGGGCATGCCTGTTCGAGCGTCATTTCAACCCTCAAGCCTGGCTTGG-TGATGGGGCACTGCTTCG--------AAAGGAGCAGGCCCTGAAATTCAGTGGCGAGCT-CGCCAGGAC-CCCGAGCGTAGTAG-TTATA-TCTCGCTCTGG-AAGGCCCTGGCGGCGCCCTGCCGTTAAA-CCCCCAACTTCTGAAAATT

>Diaporthe_rossmaniae_CAA762

NNNNNNNNNNNNNNNNNNNNNNNNNNNAGGGATCATTGCTGGAACGCG-CTTC-GGCGCA-CCCAGAAACCCTTTGTGAACTTATACCT--ATTGTTGCCTCGGCG-TAAGCTGGCTTTG--AA----------ATATAAGCC-----CCCTG---GAAACGGGGAGCA-GCCCGCCGGCGGCCAA-CTAAACTCTTGTTTCTATAGTGAATCTCTGAGT--AAAAAACATAAATGAATCAAAACTTTCAACAACGGATCTCTTGGTTCTGGCATCGATGAAGAACGCAGCGAAATGCGATAAGTAATGTGAATTGCAGAATTCAGTGAATCATCGAATCTTTGAACGCACATTGCGCCCCCTGGTATTCCGGGGGGCATGCCTGTTCGAGCGTCATTTCAACCCTCAAGCCTGGCTTGG-TGATGGGGCACTGCTTCG--------AAAGGAGCAGGCCCTGAAATTCAGTGGCGAGCT-CGCCAGGAC-CCCGAGCGTAGTAG-TTATA-TCTCGCTCTGG-AAGGCCCTGGCGGCGCCCTGCCGTTAAA-CCCCCAACTTCTGAAAATT

>Diaporthe_novem_CBS_127271

ACAAGGTCTCCGTTGGTGAACCAGCGGAGGGATCATTGCTGGAACGCG-CTTC-GGCGCA-CCCAGAAACCCTTTGTGAACTTATACCC--ACTGTTGCCTCGGCG-CAGGCCGGCCTCT--TC----------ACTGAGGCC-----CCCTG---GAAACAGGGAGCA-GCCCGCCGGTGGCCAA-CTAAACTC-TGTTTCTATAGTGAATCTCTGAGT--AAAAAACATAAATGAATCAAAACTTTCAACAACGGATCTCTTGGTTCTGGCATCGATGAAGAACGCAGCGAAATGCGATAAGTAATGTGAATTGCAGAATTCAGTGAATCATCGAATCTTTGAACGCACATTGCGCCCTCTGGTATTCCGGAGGGCATGCCTGTTCGAGCGTCATTTCAACCCTCAAGCCTGGCTTGG-TGATGGGGCACTGCCTGT--------AAAAGGGCAGGCCCTGAAATCTAGTGGCGAGCT-CGCCAGGAC-CCCGAGCGTAGTAG-TTATA-TCTCGCTCTGG-AAGGCCCTGGCGGTGCCCTGCCGTTAAA-CCCCCAACTTCTGAAAATT

>Diaporthe_pseudolongicolla_CBS_117165

NNNNNNNNNNNNNNNNNNNNNNNNNNNNNNNNTCATTGCTGGAACGCG-CTTC-GGCGCA-CCCAGAAACCCTTTGTGAACTTATACCC--ACTGTTGCCTCGGCG-CAGGCCGGCCTCT--TC----------ACTGAGGCC-----CCCTG---GAAACAGGGAGCA-GCCCGCCGGTGGCCAA-CTAAACTC-TGTTTCTATAGTGAATCTCTGAGT--AAAAAACATAAATGAATCAAAACTTTCAACAACGGATCTCTTGGTTCTGGCATCGATGAAGAACGCAGCGAAATGCGATAAGTAATGTGAATTGCAGAATTCAGTGAATCATCGAATCTTTGAACGCACATTGCGCCCTCTGGTATTCCGGAGGGCATGCCTGTTCGAGCGTCATTTCAACCCTCAAGCCTGGCTTGG-TGATGGGGCACTGCCTGT--------AAAAGGGCAGGCCCTGAAATCTAGTGGCGAGCT-CGCCAGGAC-CCCGAGCGTAGTAG-TTATA-TCTCGCTCTGG-AAGGCCCTGGCGGTGCCCTGCCGTTAAA-CCCCCAACTTCTGAAAATT

>Diaporthe_lusitanicae_CBS_123212

ACAAGGTCTCCGTTGGTGAACCAGCGGAGGGATCATTGCTGGAACGCG-CTTC-GGCGCA-CCCAGAAACCCTTTGTGAACTTATACCC--ACTGTTGCCTCGGCG-CAGGCCGGCCTCT--TC----------ACTGAGGCC-----CCCCC---GAAAGGGGGAGCA-GCCCGCCGGCGGCCAA-CTAAACTC-TGTTTCTATAGTGAATCTCTGAGT-AAAAAAACATAAATGAATCAAAACTTTCAACAACGGATCTCTTGGTTCTGGCATCGATGAAGAACGCAGCGAAATGCGATAAGTAATGTGAATTGCAGAATTCAGTGAATCATCGAATCTTTGAACGCACATTGCGCCCTCTGGTATTCCGGAGGGCATGCCTGTTCGAGCGTCATTTCAACCCTCAAGCCTGGCTTGG-TGATGGGGCACTGCCTGT--------AAAAGGGCAGGCCCTGAAATCTAGTGGCGAGCT-CGCCAGGAC-CCCGAGCGTAGTAG-TTACA-TCTCGCTCTGG-AAGGCCCTGGCGGTGCCCTGCCGTTAAA-CCCCCAACTTCTGAAAATT

>Diaporthe_yunnanensis_CGMCC_3_18289

NNNNNNNNNNNNNNNNNNNNNNNGCGGAGGGATCATTGCTGGAACGCG-CTTC-GGCGCA-CCCAGAAACCCTTTGTGAACTTATACCT--ATTGTTGCCTCGGCG-CAGGCCGGCCTCT--TC----------ACTGAGGCC-----CCCTG---GAAACAGGGAGCA-GCCCGCCGGCGGCCAA-CCAAACTCTTGTTTCTATAGTGAATCTCTGAGT-AAAAAAACATAAATGAATCAAAACTTTCAACAACGGATCTCTTGGTTCTGGCATCGATGAAGAACGCAGCGAAATGCGATAAGTAATGTGAATTGCAGAATTCAGTGAATCATCGAATCTTTGAACGCACATTGCGCCCTCTGGTATTCCGGAGGGCATGCCTGTTCGAGCGTCATTTCAACCCTCAAGCCTGGCTTGG-TGATGGGGCACTGCCTGT--------AATAGGGCAGGCCCTGAAATCTAGTGGCGAGCT-CGCCAGGAC-CCCGAGCGTAGTAG-TTATA-TCTCGCTCTGG-AAGGCCCTGGCGGTGCCCTGCCGTTAAA-CCCCCAACTTCTGAAAATT

>Diaporthe_angelicae_CBS_111592

ACAAGGTCTCCGTTGGTGAACCAGCGGAGGGATCATTGCTGGAACGCG-CCTC-GGCGCA-CCCAGAAACCCTTTGTGAACTTATACCTATACTGTTGCCTCGGCG-CAGGCCGGCCTTT--CT--------CGGTAAAGGCC-----CCCTG---GAGACAGGGAGCA-GCCCGCCGGCGGCCAG-CCAAACTC-TGTTTCTATAGTGGATCTCTGAGT--AAAAAACATAAATGAATCAAAACTTTCAACAACGGATCTCTTGGTTCTGGCATCGATGAAGAACGCAGCGAAATGCGATAAGTAATGTGAATTGCAGAATTCAGTGAATCATCGAATCTTTGAACGCACATTGCGCCCTCTGGTATTCCGGAGGGCATGCCTGTTCGAGCGTCATTTCAACCCTCAAGCCTGGCTTGG-TGATGGGGCACTGCCTGT--------GAAAGGGCAGGCCCTGAAATCTAGTGGCGAGCT-CGCCAGGAC-CCCGAGCGCAGTAG-TTACA-TCTCGCTCTGG-GAGGCCCTGGCGGTGCCCTGCCGTTAAA-CCCCCAACTTCTGAAAATT

>Diaporthe_subordinaria_CBS_101711

ACAAGGTCTCCGTTGGTGAACCAGCGGAGGGATCATTGCTGGAACGCG-CCTC-GGCGCA-CCCAGAAACCCTTTGTGAACTTATACCCATACTGTTGCCTCGGCG-CAGGCCGGCCTTT--CT--------CGGTAAAGGCC-----CCCTG---GAGACAGGGAGCA-GCCCGCCGGCGGCCAG-CCAAACTC-TGTTTCTATAGTGGATCTCTGAGT--AAAAAACATAAATGAATCAAAACTTTCAACAACGGATCTCTTGGTTCTGGCATCGATGAAGAACGCAGCGAAATGCGATAAGTAATGTGAATTGCAGAATTCAGTGAATCATCGAATCTTTGAACGCACATTGCGCCCTCTGGTATTCCGGAGGGCATGCCTGTTCGAGCGTCATTTCAACCCTCAAGCCTGGCTTGG-TGATGGGGCACTGCCTGT--------GAAAGGGCAGGCCCTGAAATCTAGTGGCGAGCT-CGCCAGGAC-CCCGAGCGTAGTAG-TTACA-TCTCGCTCTGG-GAGGCCCTGGCGGTGCCCTGCCGTTAAA-CCCCCAACTTCTGAAAATT

>Diaporthe_arctii_CBS_136_25

ACAAGGTCTCCGTTGGTGAACCAGCGGAGGGATCATTGCTGGAACGCG-CCTC-GGCGCA-CCCAGAAACCCTTTGTGAACTCATACCCATACTGTTGCCTCGGCG-CAGGCCGGCCTTT--TT--------CGACAAAGGCC-----CCCTG---GAGACAGGGAGCA-GCCCGCCGGCGGCCAA-CCAAACTC-TGTTTCTATAGTGGATCTCTGAGT--AAAAAACATAAATGAATCAAAACTTTCAACAACGGATCTCTTGGTTCTGGCATCGATGAAGAACGCAGCGAAATGCGATAAGTAATGTGAATTGCAGAATTCAGTGAATCATCGAATCTTTGAACGCACATTGCGCCCTCTGGTATTCCGGAGGGCATGCCTGTTCGAGCGTCATTTCAACCCTCAAGCCTGGCTTGG-TGATGGGGCACTGCCTGT--------GAAAGGGCAGGCCCTGAAATCTAGTGGCGAGCT-CGCCAGGAC-CCCGAGCGTAGTAG-TTACA-TCTCGCTCTGG-AAGGCCCTGGCGGTGCCCTGCCGTTAAA-CCCCCAACTTCTGAAAATT

>Diaporthe_gulyae_BRIP_54025

NNNNNNNNNNNNNNNNNNNNNNNNNNNAGGGATCATTGCTGGAACGCG-CCTC-GGCGCA-CCCAGAAACCCTTTGTGAACTTATACCCATACTGTTGCCTCGGCG-CAGGCCGGCCTTT--TT--------CGACAAAGGCC-----CCCTG---GAGACAGGGAGCA-GCCCGCCGGCGGCCAA-CCAAACTC-TGTTTCTATAGTGAATCTCTGAGT--AAAAAACATAAATGAATCAAAACTTTCAACAACGGATCTCTTGGTTCTGGCATCGATGAAGAACGCAGCGAAATGCGATAAGTAATGTGAATTGCAGAATTCAGTGAATCATCGAATCTTTGAACGCACATTGCGCCCTCTGGTATTCCGGAGGGCATGCCTGTTCGAGCGTCATTTCAACCCTCAAGCCTGGCTTGG-TGATGGGGCACTGCCTGT--------GAAAGGGCAGGCCCTGAAATCTAGTGGCGAGCT-CGCCAGGAC-CCCGAGCGTAGTAG-TTACA-TCTCGCTCTGG-AAGGCCCTGGCGGTGCCCTGCCGTTAAA-CCCCCAACTTCTGAAAATT

>Diaporthe_cucurbitae_DAOM_42078

ACAAGGTCTCCGTTGGTGAACCAGCGGAGGGATCATTGCTGGAACGCG-CCCC-GGCGCA-CCCAGAAACCCTTTGTGAACTTATACCCATACTGTTGCCTCGGCG-CAGGCCGGCCTTT--TT--------CGATAGAGGCC-----CCCTG---GAGACAGGGAGCA-GCCCGCCGGCGGCCAA-CCAAACTC-TGTTTCTATAGTGAATCTCTGAGT--AAAAAACATAAATGAATCAAAACTTTCAACAACGGATCTCTTGGTTCTGGCATCGATGAAGAACGCAGCGAAATGCGATAAGTAATGTGAATTGCAGAATTCAGTGAATCATCGAATCTTTGAACGCACATTGCGCCCTCTGGTATTCCGGAGGGCATGCCTGTTCGAGCGTCATTTCAACCCTCAAGCCTGGCTTGG-TGATGGGGCACTGCCTGT--------GAAAGGGCAGGCCCTGAAATCTAGTGGCGAGCT-CGCCAGGAC-CCCGAGCGTAGTAG-TTACA-TCTCGCTCTGG-AAGGCCCTGGCGGTGCCCTGCCGTTAAA-CCCCCAACTTCTGAAAATT

>Diaporthe_stewartii_CBS_193_36

NNNNNNNNNNNNNNNNNNNNNNNNNNNAGGGATCATTGCTGGAACGCG-CCCC-GGCGCA-CCCAGAAACCCTTTGTGAACTTATACCCATACTGTTGCCTCGGCG-CAGGCCGGCCTTT--TT--------CGATAAGGGCC-----CCCTG---GAGACAGGGAGCA-GCCCGCCGGCGGCCAA-CCAAACTC-TGTTTCTATAGTGAATCTCTGAGT--AAAAAACATAAATGAATCAAAACTTTCAACAACGGATCTCTTGGTTCTGGCATCGATGAAGAACGCAGCGAAATGCGATAAGTAATGTGAATTGCAGAATTCAGTGAATCATCGAATCTTTGAACGCACATTGCGCCCTCTGGTATTCCGGAGGGCATGCCTGTTCGAGCGTCATTTCAACCCTCAAGCCTGGCTTGG-TGATGGGGCACTGCCTGT--------GAAAGGGCAGGCCCTGAAATCTAGTGGCGAGCT-CGCCAGGAC-CCCGAGCGTAGTAG-TTACA-TCTCGCTCTGG-AAGGCCCTGGCGGTGCCCTGCCGTTAAA-CCCCCAACTTCTGAAAATT

>Diaporthe_neoarctii_CBS_109490

ACAAGGTCTCCGTTGGTGAACCAGCGGAGGGATCATTGCTGGAACGCG-CCTC-GGCGCA-CCCAGAAACCCTTTGTGAACTTATACCCAAACTGTTGCCTCGGCG-CAGGCCGGCCCCT--CT--------CGTTAGGGGCC-----CCCTG---GAGACAGGGAGCA-GCCCGCCGGCGGCCGA-CCAAACTC-TGTTTCTATAGTGAATCTCTGAGT--AAAAAACATAAATGAATCAAAACTTTCAACAACGGATCTCTTGGTTCTGGCATCGATGAAGAACGCAGCGAAATGCGATAAGTAATGTGAATTGCAGAATTCAGTGAATCATCGAATCTTTGAACGCACATTGCGCCCTCTGGTATTCCGGAGGGCATGCCTGTTCGAGCGTCATTTCAACCCTCAAGCCTGGCTTGG-TGATGGGGCACTGCCTGT--------TAAAGGGCAGGCCCTGAAATCTAGTGGCGAGCT-CGCCAGGAC-CCCGAGCGTAGTAG-TTACA-TCTCGCTCTGG-AAGGCCCTGGCGGTGCCCTGCCGTTAAA-CCCCCAACTTCTGAAAATT

>Diaporthe_cuppatea_CBS_117499

NNNNNNNNNNNNNNNNNNNNNNNNNNNNNGGATCATTGCTGGAACGCG-CTTT-GGCGCA-CCCAGAAACCCTTTGTGAACTTATACCC--ACTGTTGCCTCGGCG-CAGGCCGGCCTTT--GT--------TGCAAAAGGCC-----CCCTG---GAAACAGGGAGCA-GCCCGCCGGCGGCCAA-CCAAACTC-TGTTTCTATAGTGAATCTCTGAGT--AAAAAACATAAATGAATCAAAACTTTCAACAACGGATCTCTTGGTTCTGGCATCGATGAAGAACGCAGCGAAATGCGATAAGTAATGTGAATTGCAGAATTCAGTGAATCATCGAATCTTTGAACGCACATTGCGCCCTCTGGTATTCCGGAGGGCATGCCTGTTCGAGCGTCATTTCAACCCTCAAGCCCGGCTTGG-TGATGGGGCACTGCCTGT--------AAAAGGGCAGGCCCTGAAATCTAGTGGCGAGCT-CGCCAGGAC-CCCGAGCGTAGTAG-TCATA-TCTCGCTCTGG-AAGGCCCTGGCGGTGCCCTGCCGTTAAA-CCCCCAACTTCTGAAAATT

>Diaporthe_arezzoensis_MFLU_19_2880

ACAAGGTCTCCGTTGGTGAACCAGCGGAGGGATCATTGCTGGAACGCG-CCCC-GGCGCA-CCCAGAAACCCTTTGTGAACTCATACCTAT-CTGTTGCCTCGGCG-CAGGCCGGCCCCC--TC----------ACAGGGGCC-----CCCCG---GAGACGGGGAGCA-GCCCGCCGGCGGCCAA-CCAAACTCTTGTTTCTACAGTGTATCTCTGAGA-GAAAAAACATAAATGAATCAAAACTTTCAACAACGGATCTCTTGGTTCTGGCATCGATGAAGAACGCAGCGAAATGCGATAAGTAATGTGAATTGCAGAATTCAGTGAATCATCGAATCTTTGAACGCACATTGCGCCCTCTGGTATTCCGGAGGGCATGCCTGTTCGAGCGTCATTTCAACCCTCAAGCCTGGCTTGG-TGATGGGGCACTGCCCGT--------AGAAGGGCAGGCCCTGAAATCTAGTGGCGAGCT-CGCCAGGAC-CCCGAGCGTAGTAG-TTATA-TCTCGCTCCGG-AAGGCCCTGGCGGTGCCCTGCCGTTAAA-CCCCCAACTTCTGAAAATT

>Diaporthe_biguttulata_ICMP20657

NNNNNNNNNNNNNNNNNNNNNNNNNNNNNNNNNNNNNNCTGGAACGCG-CCTC-GGCGCA-CCCAGAAACCCTTTGTGAACTTATACCT-TACTGTTGCCTCGGCG-CAGGCCGGCCTCT--TA----------GCTGAGGCC-----CCCCG---GAGACGGGGAGCA-GCCCGCCGGCGGCCAA-CCTAACTCTTGTTTTTACACTGTATCTCTGAGC-AAAAAAACATAAATGAATCAAAACTTTCAACAACGGATCTCTTGGTTCTGGCATCGATGAAGAACGCAGCGAAATGCGATAAGTAATGTGAATTGCAGAATTCAGTGAATCATCGAATCTTTGAACGCACATTGCGCCCTCTGGTATTCCGGAGGGCATGCCTGTTCGAGCGTCATTTCAACCCTCAAGCCTGGCTTGG-TGTTGGGGCACTGCTTCG--------AGAGAAGCAGGCCCTGAAATCTAGTGGCGAGCT-CGCCAGGAC-CCCGAGCGTAGTAG-TTATA-TCTCGTTCTGG-AAGGCCCTGGCGGTGCCCTGCCGTTAAA-CCCCCAACTTCTGAAAATT

>Diaporthe_discoidispora_ICMP20662

NNNNNNNNNNNNNNNNNNNNNNNNNNNNNNNNNNNNNNCTGGAACGCG-CCTC-GGCGCA-CCCAGAAACCCTTTGTGAACTTATACCT-TACTGTTGCCTCGGCG-CAGGCCGGCCTCT--TA----------GCTGAGGCC-----CCCCG---GAGACGGGGAGCA-GCCCGCCGGCGGCCAA-GTTAACTCTTGTTTCTACCATGAATCTCTGAGT-AAAAAAACATAAATGAATCAAAACTTTCAACAACGGATCTCTTGGTTCTGGCATCGATGAAGAACGCAGCGAAATGCGATAAGTAATGTGAATTGCAGAATTCAGTGAATCATCGAATCTTTGAACGCACATTGCGCCCTCTGGTATTCCGGAGGGCATGCCTGTTCGAGCGTCATTTCAACCCTCAAGCCTGGCTTGG-TGTTGGGGCACTGCTTCG--------AGAGAAGCAGGCCCTGAAATCTAGTGGCGAGCT-CGCTAGGAC-CCCGAGCGTAGTAG-TTATA-TCTCGTTCTGG-AAGGCCCTGGCGGTGCCCTGCCGTTAAA-CCCCCAACTTCTGAAAATT

>Diaporthe_guttulata_CGMCC_3_20100

NNNNNNNNNNNNNNNNNNNNNNNNNNNNNNNNNNNNNNNNNNNNNNNNNNNTC-GGCGCA-CCCAGAAACCCTTTGTGAACTTATACCCATACTGTTGCCTCGGCG-CAGGCCGGCCCTT--TT--------CGATAAGGGCC-----CCCTG---GAGACAGGGAGCA-GCCCGCCGGCGGCCAA-CCAAACTC-TGTTTCTATAGTGAATCTCTGAGT--AAAAAACATAAATGAATCAAAACTTTCAACAACGGATCTCTTGGTTCTGGCATCGATGAAGAACGCAGCGAAATGCGATAAGTAATGTGAATTGCAGAATTCAGTGAATCATCGAATCTTTGAACGCACATTGCGCCCTCTGGTATTCCGGAGGGCATGCCTGTTCGAGCGTCATTTCAACCCTCAAGCCTGGCTTGG-TGATGGGGCACTGCCTGT--------GAAAGGGCAGGCCCTGAAATCTAGTGGCGAGCT-CGCCAGGAC-CCCGAGCGTAGTAG-TTACA-TCTCGCTCTGG-AAGGCCCTGGCGGTGCCCTGCCGTTAAA-CCCCCAACTNNNNNNNNNN

>Diaporthe_schoeni_MFLU_15_1279

NNNNNNNNNNNNNNNNNNNNNNNNNNNNNNNNNNNNNNNNNNNNNNNNNNNNNNNNNNNNNNCCAG-AACCCTTTGTG-ACTTATACCCATACTGTTGCCTCGGCG-CAGGCCGGCCTTT--TT--------CGATAAGGGCC-----CCCTG---GAGACAGGGAGCA-GCCCGCCGGCGGCCAA-CCCAAACTCTGTTTCTATAGTGAATCTCTGAGT--AAAAAACATAAATGAATCAAAACTTTCAACAACGGATCTCTTGGTTCTGGCATCGATGAAGAACGCAGCGAAATGCGATAAGTAATGTGAATTGCAGAATTCAGTGAATCATCGAATCTTTGAACGCACATTGCGCCCTCTGGTATTCCGGAGGGCATGCCTGTTCGAGCGTCATTTCAACCCTCAAGCCTGGCTTGG-TGATGGGGCACTGCCTGT--------AAAAGGGCAGGCCCTGAAATCTAGTGGCGAGCT-CGCCAGGAC-CCCGAGCGTAGTAG-TTACA-TCTCGCTCTGG-AAGGCCCTGGCGGTGCCCTGCCGTTAAA-CCCCCAACTTCTGAAAATT

>Diaporthe_cichorii_MFLUCC_17_1023

NNNNNNNNNNNNNNNNNNNNNNNNNNNNNNNNNNNNNNNNNNNNNNNNNNNNNNNNNNNNNNNCCAGAACCCTTTGTGAACTTATACCCATACTGTTGCCTCGGCG-CAGGCCGGCCCCT--GC---------TGCAGGGGCC-----CCCTG---GGGACAGGGAGCA-GCCCGCCGGCGGCCAA-CCAAACTC-TGTTTCTATAGTGGATCTCTGAGT-AAAAAAACATAAATGAATCAAAACTTTCAACAACGGATCTCTTGGTTCTGGCATCGATGAAGAACGCAGCGAAATGCGATAAGTAATGTGAATTGCAGAATTCAGTGAATCATCGAATCTTTGAACGCACATTGCGCCCTCTGGTATTCCGGAGGGCATGCCTGTTCGAGCGTCATTTCAACCCTCAAGCCTGGCTTGG-TGATGGGGCACTGCCTGT--------GAAAGGGCAGGCCCTGAAATCTAGTGGCGAGCT-CGCCAGGAC-CCCGAGCGTAGTAG-TTACA-TCTCGCTCTGG-AAGGCCCTGGCGGTGCCCTGCCGTTAAA-CCCCCAACTTCTGAAAATT

>Diaporthe_acericola_MFLUCC_17_0956

NNNNNNNNNNNNNNNNNNNNNNNNNNNNNNNNNNNNNNNNNNNNNNNNNNNNNNNNNNNNNNNCCAGAACCCTTTGTG-ACTTATACCC--ACTGTTGCCTCGGCG-CAGGCCGGCCTCT--TC----------GCTGAGGCC-----CCCTG---GAAACAGGGAGCA-GCCCGCCGGTGGCCAA-CTAAACTC-TGTTTCTATAGTGAATCTCTGAGT--AAAAAACATAAATGAATCAAAACTTTCAACAACGGATCTCTTGGTTCTGGCATCGATGAAGAACGCAGCGAAATGCGATAAGTAATGTGAATTGCAGAATTCAGTGAATCATCGAATCTTTGAACGCACATTGCGCCCTCTGGTATTCCGGAGGGCATGCCTGTTCGAGCGTCATTTCAACCCTCAAGCCTGGCTTGG-TGATGGGGCACTGCCTGT--------AAAAGGGCAGGCCCTGAAATCTAGTGGCGAGCT-CGCCAGGAC-CCCGAGCGTAGTAG-TTATA-TCTCGCTTTGG-AAGGCCCTGGCGGTGCCCTGCCGTTAAA-CCCCCAACTTCTGAAAATT

>Diaporthe_cinnamomi_CFCC_52569

NNNNNNNNNNNNNNNNNNNNNNNNNNNNNNNNNNNNNNNNNNNNNNNNNNNNNNNNNNNNNNNNNNNNNCCCTTTGTGAACTTATACCT-TACTGTTGCCTCGGCG-CAGGCCGGCCTCT--TA----------GCTGAGGCC-----CCCCG---GAGACGGGGAGCA-GCCCGCCGGCGGCCAA-GTTAACTCTTGTTTCTACCCTGAATCTCTGAGT--AAAAAACATAAATGAATCAAAACTTTCAACAACGGATCTCTTGGTTCTGGCATCGATGAAGAACGCAGCGAAATGCGATAAGTAATGTGAATTGCAGAATTCAGTGAATCATCGAATCTTTGAACGCACATTGCGCCCTCTGGTATTCCGGAGGGCATGCCTGTTCGAGCGTCATTTCAACCCTCAAGCCTGGCTTGG-TGCTGGGGCACTGCTTCG--------AGAGAAGCAGGCCCTGAAATCTAGTGGCGAGCT-CGCTAGGAC-CCCGAGCGTAGTAA-TTATA-TCTCGTTCTGG-AAGGCCCTGGCGGTGCCCTGCCGTTAAA-CCCCCAACTTCTGAAANNN

>Diaporthe_siamensis_MFLUCC_10_0573a

NNNNNNNNNNNNNNNNNNNNNNNNNNNNNNNNNNNNNNNNNNNNNNNNNNNNNNNNNNNNNNNNNNNNNCCCTTTGTGAACTTATACCT-TACTGTTGCCTCGGCG-CAGGCCGGCCTCT--TA----------GCTGAGGCC-----CCCCG---GAGACGGGGAGCA-GCCCGCCGGCGGCCAA-CCAAACTCTTGTTTCTACAGTGAATCTCTGAGT--AAAAAACATAAATGAATCAAAACTTTCAACAACGGATCTCTTGGTTCTGGCATCGATGAAGAACGCAGCGAAATGCGATAAGTAATGTGAATTGCAGAATTCAGTGAATCATCGAATCTTTGAACGCACATTGCGCCCTCTGGTATTCCGGAGGGCATGCCTGTTCGAGCGTCATTTCAACCCTCAAGCCTGGCTTGG-TGTTGGGGCACTGCTTCG--------AGAGAAGCAGGCCCTGAAATCTAGTGGCGAGCT-CGCTAGGAC-CCCGAGCGTAGTAG-TTATA-TCTCGTTCTGG-AAGGCCCTGGCGGTGCCCTGCCGTTAAA-CCCCCAACTTCTGAAATTT

>Phomopsis_vitimegaspora_STE-U2675

NNNNNNNNNNNNNNNNNNNNNNNNNNNNNNNNNNNNNNNNNNNNNNNNNNNNNNNNNNNNNNNNNNNAACCCTTTGTGAACTCATACCT-TACTGTTGCCTCGGCG-CAGGCCGGCCTCT--CA----------GCTGAGGCC-----CCCCG---GAGACGGGGAGCA-GCCCGCCGGCGGCCAG-CCAAACTCTTGTTTCTACAGTGAATCTCTGAGT-AAGAAAACATAAATGAATCAAAACTTTCAACAACGGATCTCTTGGTTCTGGCATCGATGAAGAACGCAGCGAAATGCGATAAGTAATGTGAATTGCAGAATTCAGTGAATCATCGAATCTTTGAACGCACATTGCGCCCTCTGGTATTCCGGAGGGCATGCCTGTTCGAGCGTCATTTCAACCCTCAAGCCTGGCTTGG-TGTTGGGGCACTGCCTGT--------GAGAGGGCAGGCCCTGAAATCTAGTGGCGAGCT-CGCCAGGAC-CCCGAGCGTAGTAG-TTATA-TCTCGTTCTGG-AAGGCCCTGGCGGCGCCCTGCCGTTAAA-CCCCCAACTTCTGAAATTT

>Diaporthe_citriasiana_CBS_134240

ACAAGGTCTCCGTTGGTGAACCAGCGGAGGGATCATTGCTGGAACGCG-CCCC-GGCGCA-CCCAGAAACCCTTTGTGAACTCATACCT-TACTGTTGCCTCGGCG-CAGGCCGGCCTCT--AC---------TGCTGAGGCC-----CCCCG---GGGACGGGGAGCA-GCCCGCCGGCGGCCAA-GCCAACTCTTGTTTCTACAGTGAATCTCTGAGC--AAAAAACATAAATGAATCAAAACTTTCAACAACGGATCTCTTGGTTCTGGCATCGATGAAGAACGCAGCGAAATGCGATAAGTAATGTGAATTGCAGAATTCAGTGAATCATCGAATCTTTGAACGCACATTGCGCCCTCTGGTATTCCGGAGGGCATGCCTGTTCGAGCGTCATTTCAACCCTCAAGCCTGGCTTGG-TGCTGGGGCACTGCTCCG--------AGAGGAGCAGGCCCTGAAATCTAGTGGCGAGCT-CGCCAGGAC-CCCGAGCGCAGTAG-TTACA-TCTCGTTCTGG-AAGGCCCTGGCGGTGCCCTGCCGTTAAA-CCCCCAACTTCTGAAATTT

>Diaporthe_chinensis_MFLUCC_19_0101

NNNNNNNNTCCGTTGGTGAACCAGCGGAGGGATCATTGCTGGAACGCG-CTTC-GGCGCA-CCCAGAAACCCTTTGTGAACTTATACCT-TACTGTTGCCTCGGCG-CAGGCCGGCCTCT--AC---------TGCTGAGGCC-----CCCCG---GAGACGGGGAGCA-GCCCGCCGGCGGCCAA-CCAAACTCTTGTTTCTACAGTGAATCTCTGAGT--AAAAAACATAAATGAATCAAAACTTTCAACAACGGATCTCTTGGTTCTGGCATCGATGAAGAACGCAGCGAAATGCGATAAGTAATGTGAATTGCAGAATTCAGTGAATCATCGAATCTTTGAACGCACATTGCGCCCTCTGGTATTCCGGAGGGCATGCCTGTTCGAGCGTCATTTCAACCCTCAAGCCTGGCTTGG-TGTTGGGGCACTGCCTGT--------AAAAGGGCAGGCCCTGAAATCTAGTGGCGAGCT-CGCTAGGAC-CCCGAGCGTAGTAG-TTATA-TCTCGTTCTGG-AAGGCCCTGGCGGTGCCCTGCCGTTAAA-CCCCCNNNNNNNNNNNNNN

>Diaporthe_fici_septicae_MFLU_18_2588

NNNNNNNNTCCGTTGGTGAACCAGCGGAGGGA-CATTGCTGGAACGCG-CCCC-GGCGCA-CCCAGAAACCCTTTGTGAACTTATACCT-TACTGTTGCCTCGGCG-CAGGCCGGCCTCC--CA----------GCTGAGGCC-----CCCCG---GAGACGGGGAGCA-GCCCGCCGGCGGCCAA-CTAAACTCTTGTTTCTACAGTGGATCTCTGAGTTAAAAAAACATAAATGAATCAAAACTTTCAACAACGGATCTCTTGGTTCTGGCATCGATGAAGAACGCAGCGAAATGCGATAAGTAATGTGAATTGCAGAATTCAGTGAATCATCGAATCTTTGAACGCACATTGCGCCCTCTGGTATTCCGGAGGGCATGCCTGTTCGAGCGTCATTTCAACCCTCAAGCCTGGCTTGG-TGTTGGGGCACTGCCTGT--------AAAAGGGCAGGCCCTGAAATCTAGTGGCGAGCT-CGCTAGGAC-CCCGAGCGTAGTAG-TTATA-TCTCGTTCTGG-AAGGCCCTGGCGGCGCCCTGCCGTTAAA-CCCCCNNNNNNNNNNNNNN

>Diaporthe_ambigua_CBS_114015

ACAAGGTCTCCGTTGGTGAACCAGCGGAGGGATCATTGCTGGAACGCG-CCTC-GGCGCA-CCCAGAAACCCTTTGTGAACTTATACCT--ATCGTTGCCTCGGCG-AAGGCCGGCCTCC--CC----------ACCGAGGCC-----CCTTG---GGAACAAGGAGCA-GCCCGCCGGCGGCCAA-CCAAACTCTTGTTTCT-TAGTGAATCTCTGAGTAAAAAAAACATAAATGAATCAAAACTTTCAACAACGGATCTCTTGGTTCTGGCATCGATGAAGAACGCAGCGAAATGCGATAAGTAATGTGAATTGCAGAATTCAGTGAATCATCGAATCTTTGAACGCACATTGCGCCCTCTGGTATTCCGGAGGGCATGCCTGTTCGAGCGTCATTTCAACCCTCAAGCCTGGCTTGG-TGATGGGGCACTGCTTCCGA------GAGGGAGCAGGCCCTGAAATCTAGTGGCGAGCT-CGCCAGGAC-CCCGAGCGTAGTAG-TTATA-TCTCGCTCCGG-AAGGCCCTGGCGGTGCCCTGCCGTTAAA-CCCCCAACTTCTGAAAATT

>Diaporthe_compacta_LC3083

NNNNNNNNNNNNNNNNNNNNNNNNNNNNGGGATCATTGCTGGAACGCG-CCTC-GGCGCA-CCCAGAAACCCTTTGTGAACTTATACCC--ACTGTTGCCTCGGCG-CAGGCCGGTCTGC---------------CTCAGACC-----CCCTG---GAGACAGGGAGCA-GCCCGCCGGCGGCCAA-CCAAACTC-TGTTTCTATAGTGAATCTCTGAGT--AAAAAACATAAATGAATCAAAACTTTCAACAACGGATCTCTTGGTTCTGGCATCGATGAAGAACGCAGCGAAATGCGATAAGTAATGTGAATTGCAGAATTCAGTGAATCATCGAATCTTTGAACGCACATTGCGCCCTCTGGTATTCCGGAGGGCATGCCTGTTCGAGCGTCATTTCAACCCTCAAGCCTGGCTTGG-TGATGGGGCAGTGCCTTGGA------GACAAGGCACGCCCTGAAATTCAGTGGCGAGCT-CGCCAGGAC-CCCGAGCGTAGTAG-TTACA-TCTCGCTCTGG-AAGGCCCTGGCGGTGCCCTGCCGTTAAA-CCCCCAACTTCTGAAANNN

>Diaporthe_sambucusii_CFCC_51986

NNNNNNNNNNNNNNNNNNNNNNNNNNNNNNNNNNNNNNNNNNNNNNNNNNNNNNNNNNNNNNNNNNNNNCCCTTTGTGAACTTATACCC--ACTGTTGCCTCGGCG-CAGGCCGGTCTGT---------------CTCAGACC-----CCCTG---GAAACAGGGAGCA-GCCCGCCGGCGGCCAA-CCAAACTC-TGTTTCTATAGTGAATCTCTGAGT--AAAAAACATAAATGAATCAAAACTTTCAACAACGGATCTCTTGGTTCTGGCATCGATGAAGAACGCAGCGAAATGCGATAAGTAATGTGAATTGCAGAATTCAGTGAATCATCGAATCTTTGAACGCACATTGCGCCCTCTGGTATTCCGGAGGGCATGCCTGTTCGAGCGTCATTTCAACCCTCAAGCCTGGCTTGG-TGATGGGGCAGTGCCTTGGA------GACAAGGCACGCCCTGAAATTCAGTGGCGAGCT-CGCCAGGAC-CCCGAGCGTAGTAG-TTACA-TCTCGCTCTGG-AAGGCCCTGGCGGTGCCCTGCCGTTAAA-CCCCCAACTTCTGAAANNN

>Diaporthe_manihotia_CBS_505_76

NNNNNNNNNNNNNNNNNNNNNNNGCGGAGGGATCATTGCTGGAACGCG-CTTC-GGCGCA-CCCAGAAACCCTTTGTGAACTTATACCT--ACTGTTGCCTCGGCG-CAGGCCGGTCTGT---------------CTCAGACC-----CCCTG---GAAACAGGGAGCA-GCCCGCCGGCGGCCAA-CTAAACTC-TGTTTCTATAGTGAATCTCTGAGT--AAAAAACATAAATGAATCAAAACTTTCAACAACGGATCTCTTGGTTCTGGCATCGATGAAGAACGCAGCGAAATGCGATAAGTAATGTGAATTGCAGAATTCAGTGAATCATCGAATCTTTGAACGCACATTGCGCCCTCTGGTATTCCGGAGGGCATGCCTGTTCGAGCGTCATTTCAACCCTCAAGCCTGGCTTGG-TGATGGGGCAGTGCTCTGGA------GACAGAGCACGCCCTGAAATTCAGTGGCGAGCT-CGCCAGGAC-CCCGAGCGTAGTAG-TTATA-TCTCGCTCTGG-AAGGCCCTGGCGGTGCCCTGCCGTTAAA-CCCCCAACTTCTGAAAATT

>Diaporthe_ganjae_CBS_180_91

ACAAGGTCTCCGTTGGTGAACCAGCGGAGGGATCATTGCTGGAACGCG-CCCC-GGCGCA-CCCAGAAACCCTTTGTGAACCTATACCC--ACTGTTGCCTCGGCG-CAGGCCGGTCTGT---------------CTCAGACC-----CCCTG---GAGACAGGGAGCA-GCCCGCCGGCGGCCGA-CCAAACTC-CGTTTCTATAGTGAATCTCTGAGT--TAAAAACATAAATGAATCAAAACTTTCAACAACGGATCTCTTGGTTCTGGCATCGATGAAGAACGCAGCGAAATGCGATAAGTAATGTGAATTGCAGAATTCAGTGAATCATCGAATCTTTGAACGCACATTGCGCCCTCTGGTATTCCGGAGGGCATGCCTGTTCGAGCGTCATTTCAACCCTCAAGCCTGGCTTGG-TGATGGGGCAGTGCCTTGGA------GACAAGGCACGCCCTGAAATTCAGTGGCGAGCT-CGCCAGGAC-CCCGAGCGTAGTAG-TTACA-TCTCGCTCCGG-AAGGCCCTGGCGGTGCCCTGCCGTTAAA-CCCCCAACTTCTGAAAATT

>Diaporthe_alangii_CFCC_52556

NNNNNNNNNNNNNNNNNNNNNNNNNNNNNNNNNNNNNNNNNNNNNNNNNNNNNNNNNNNNNNNNNNNNNCCCTTTGTGAACTTATACCTATACTGTTGCCTCGGCG-CTGGCCGGCCTCC--TC----------ACCGAGGCC-----CCCTG---GAGACAGGGAGCA-GCCCGCCGGCGGCCAA-ACAAACTCTTGTTTCT-TAGTGAATCTCTGAGT--AAAAAACATAAATGAATCAAAACTTTCAACAACGGATCTCTTGGTTCTGGCATCGATGAAGAACGCAGCGAAATGCGATAAGTAATGTGAATTGCAGAATTCAGTGAATCATCGAATCTTTGAACGCACATTGCGCCCTCTGGTATTCCGGAGGGCATGCCTGTTCGAGCGTCATTTCAACCCTCAAGCCTGGCTTGG-TGTTGGGGCACCGCCTTTGC------AAAAGGGCGGGCCCTGAAATCTAGTGGCGAGCT-CGCCAGGAC-CCCGAGCGTAGTAG-TTATA-TCTCGTTCTGG-AAGGCCCTGGCGGTGCCCTGCCGTTAAA-CCCCCAACTTCTGAAANNN

>Phomopsis_glabrae_SCHM_3622

NNNNNNNNNNNNNNNNNNNNCCAGCGGAGGGATCATTGCTGGAACGCG-CTTC-GGCGCA-CCCAGAAACCCTTTGTGAACTTATACCTATACTGTTGCCTCGGCG-CTGGCCGGCCTCC--TC----------ACCGAGGCC-----CCCTG---GAGACAGGGAGCA-GCCCGCCGGCGGCCAA-ACAAACTCTTGTTTCT-TAGTGAATCTCTGAGT--AAAAAACATAAATGAATCAAAACTTTCAACAACGGATCTCTTGGTTCTGGCATCGATGAAGAACGCAGCGAAATGCGATAAGTAATGTGAATTGCAGAATTCAGTGAATCATCGAATCTTTGAACGCACATTGCGCCCTCTGGTATTCCGGAGGGCATGCCTGTTCGAGCGTCATTTCAACCCTCAAGCCTGGCTTGG-TGTTGGGGCACCGCCTTTGC------AAAAGGGCGGGCCCTGAAATCTAGTGGCGAGCT-CGCCAGGAC-CCCGAGCGTAGTAG-TTATA-TCTCGTTCTGG-AAGGCCCTGGCGGTGCCCTGCCGTTAAA-CCCCCAACTTCTGAAATTT

>Diaporthe_hubeiensis_JZB320123

NNNNNNNNNNNNNNNNNNNNNNNNNNNNNNNNNNNNNNNNNNNACGCG-CTTC-GGCGCA-CCCAGAAACCCTTTGTG-ACTTATACCTATACTGTTGCCTCGGCG-CTGGCCGGCCTCC--TC----------ACCGAGGCC-----CCCTG---GAGACAGGGAGCA-GCCCGCCGGCGGCCAA-ACAAACTCTTGTTTCT-TAGTGAATCTCTGAGT--AAAAAACAT-AATGAATCAAAACTTTCAACAACGGATCTCTTGGTTCTGGCATCGATGAAGAACGCAGCGAAATGCGATAAGTAATGTGAATTGCAGAATTCAGTGAATCATCGAATCTTTGAACGCACATTGCGCCCTCTGGTATTCCGGAGGGCATGCCTGTTCGAGCGTCATTTCAACCCTCAAGCCTGGCTTGG-TGTTGGGGCACCGCCTTTGC------AAAAGGGCGGGCCCTGAAATCTAGTGGCGAGCT-CGCCAGGAC-CCCGAGCGTAGTAG-TTATA-TCTCGTTCTGG-AAGGCCCTGGCGGTGCCCTGCCGTTAAA-CCCCCAACTTCTGAAATTT

>Diaporthe_tectonae_MFLUCC_12_0777

ACAAGGTCTCCGTTGGTGAACCAGCGGAGGGATCATTGCTGGAACGCG-CTTC-GGCGCA-CCCAGAAACCCTTTGTGAACTTATACCTATACTGTTGCCTCGGCG-CTGGCCGGCCTCC--TC----------ACCGAGGCC-----CCCTG---GAGACAGGGAGCA-GCCCGCCGGCGGCCAA-ACAAACTCTTGTTTCT-TAGTGAATCTCTGAGT--AAAAAACAT-AATGAATCAAAACTTTCAACAACGGATCTCTTGGTTCTGGCATCGATGAAGAACGCAGCGAAATGCGATAAGTAATGTGAATTGCAGAATTCAGTGAATCATCGAATCTTTGAACGCACATTGCGCCCTCTGGTATTCCGGAGGGCATGCCTGTTCGAGCGTCATTTCAACCCTCAAGCCTGGCTTGG-TGTTGGGGCACCGCCTTTGC------AAAAGGGCGGGCCCTGAAATCTAGTGGCGAGCT-CGCCAGGAC-CCCGAGCGTAGTAG-TTATA-TCTCGTTCTGG-AAGGCCCTGGCGGTGCCCTGCCGTTAAA-CCCCCAACTTCTGAAA-TT

>Diaporthe_tulliensis_BRIP_62248a

ACAAGGTCTCCGTTGGTGAACCAGCGGAGGGATCATTGCTGGAACGCG-CTTC-GGCGCA-CCCAGAAACCCTTTGTGAACTTATACCTATACTGTTGCCTCGGCG-CTGGCCGGCCTCC--TC----------ACCGAGGCC-----CCCTG---GAGACAGGGAGCA-GTCCGCCGGCGGCCAA-CCAAACTCTTGTTTCT-TAGTGAATCTCTGAGT--AAAAAACAT-AATGAATCAAAACTTTCAACAACGGATCTCTTGGTTCTGGCATCGATGAAGAACGCAGCGAAATGCGATAAGTAATGTGAATTGCAGAATTCAGTGAATCATCGAATCTTTGAACGCACATTGCGCCCTCTGGTATTCCGGAGGGCATGCCTGTTCGAGCGTCATTTCAACCCTCAAGCCTAGCTTGGTTGTTGGGGCACCGCCTTCGC------AAGAGGGCGGGCCCTGAAATCTAGTGGCGAGCT-CGCCAGGAC-CCCGAGCGTAGTAG-TTATA-TCTCGTTCTGG-AAGGCCCTGGCGGTGCCCTGCCGTTAAA-CCCCCAACTTCTGAAATTT

>Diaporthe_camporesii_JZB320143

NNNNNNNNNNNNNNCCCTCGCGACGCGGAGGGACATTGCTGG-ACGCG-CTTC-GGCGCA-CCCAGAAACCCTTTGTGAACTTATACCT--ATTGTTGCCTCGGCG-TAGGCCGGCCTCT--TC----------ACTGAGGCC-----CCCTG---GAAACAGGGAGCA-GCCCGCCGGCGGCCAA-CCAAACTCTTGTTTCTACAGTGAATCTCTGAGT--AAAAAACATAAATGAATCAAAACTTTCAACAACGGATCTCTTGGTTCTGGCATCGATGAAGAACGCAGCGAAATGCGATAAGTAATGTGAATTGCAGAATTCAGTGAATCATCGAATCTTTGAACGCACATTGCGCCCTCTGGTATTCCGGAGGGCATGCCTGTTCGAGCGTCATTTCAACCCTCAAGCCTGGCTTGG-TGATGGGGCACTGCTTTCGTC-----CAGAAAGCAGGCCCTGAAATCTAGTGGCGAGCT-CGCCAGGAC-CCCGAGCGTAGTAG-TTATA-TCTCGCTCCGG-AAGGCCCTGGCGGTGCCCTGCCGTTAAA-CCCCCAACTTCTGAAAATT

>Diaporthe_cerradensis_CMRP4331

ACAAGGTCTCCGTTGGTGAACCAGCGGAGGGATCATTGCTGGAACGCG-CTTC-GGCGCA-CCCAGAAACCCTTTGTGAACTTATACCT-TACTGTTGCCTCGGCG-CAGGCCGGCCCCT--CC----------CACGGGGCC-----CCTCC---GGAAGGAGGAGCA-GCCCGCCGGCGGCCAA-CTAAACTCTTGTTTCT-TAGTGAATCTCTGAGT--AAAAAACATAAATGAATCAAAACTTTCAACAACGGATCTCTTGGTTCTGGCATCGATGAAGAACGCAGCGAAATGCGATAAGTAATGTGAATTGCAGAATTCAGTGAATCATCGAATCTTTGAACGCACATTGCGCCCTCTGGTATTCCGGAGGGCATGCCTGTTCGAGCGTCATTTCAACCCTCAAGCACTGCTTGG-TGTTGGGGCACCGCCTGTG--------AAAGGGCGGGCCCTGAAAACTAGTGGCGAGCT-CGCCAGGAC-CCCGAGCGTAGTAGTTTATA-TCTCGTTCTGG-AAGGCCCTGGCGGTGCACTGCCGTTAAA-CCCCCAACTTCTGAAATTT

>Diaporthe_sclerotioides_CBS_296_67

ACAAGGTCTCCGTTGGTGAACCAGCGGAGGGATCATTGCTGGAACGCG-CTTC-GGCGCA-CCCAGAAACCCTTTGTGAACTTATACCT-TACTGTTGCCTCGGCG-CAGGCCGGCCTC---------------ACCGAGGCC-----CCTCG---GAAACGAGGAGCA-GCCCGCCGGCGGCCGA-CCAAACTCTTGTTTCT-CAGTGGATCTCTGAGT--AAAAAA-AAAAATGAATCAAAACTTTCAACAACGGATCTCTTGGTTCTGGCATCGATGAAGAACGCAGCGAAATGCGATAAGTAATGTGAATTGCAGAATTCAGTGAATCATCGAATCTTTGAACGCACATTGCGCCCTCTGGTATTCCGGAGGGCATGCCTGTTCGAGCGTCATTTCAACCCTCAAGCACTGCTTGG-TGTTGGGGCACCGCCTGTA--------AAAGGGCGGGCCCTGAAATCTAGTGGCGAGCT-CGCCGGGAC-CCCGAGCGTAGTAAATTATA-TTTCGTTCTGG-AAGGCCCCGGCGGTGCCCTGCCGTTAAA-CCCCCAACTCCTGAAAATT

>Diaporthe_neoraonikayaporum_MFLUCC_14_1136

ACAAGGTCTCCGTTGGTGAACCAGCGGAGGGATCATTGCTGGAACGCG-CTTC-GGCGCA-CCCAGAAACCCTTTGTGAACTTATACCT-TACTGTTGCCTCGGCG-CAGGCCGGCCTCT--C------------CTGAGGCC-----CCTCC---GGAAGGAGGAGCA-GCCCGCCGGCGGCCAG-CCAAACTCTTGTTTCT-TAGTGAATCTCTGAGT--AAACAACACAAATGAATCAAAACTTTCAACAACGGATCTCTTGGTTCTGGCATCGATGAAGAACGCAGCGAAATGCGATAAGTAATGTGAATTGCAGAATTCAGTGAATCATCGAATCTTTGAACGCACATTGCGCCCTTTGGTATTCCGAAGGGCATGCCTGTTCGAGCGTCATTTCAACCCTCAAGCCCGGCTTGG-TGTTGGGGCACTACTCCGA-------AGAGGAGTAGGCCCTGAAATCTAGTGGCGAGCT-CGCCAGGAC-CCCGAGCGTAGTAGTTTATA-TCTCGCTCTGG-AAGGCCCTGGCGGTGCCCTGCCGTTAAA-CCCCCAACTTCTGAAAATT

>Diaporthe_raonikayaporum_CBS_133182

ACAAGGTCTCCGTTGGTGAACCAGCGGAGGGATCATTGCTGGAACGCG-CTTC-GGCGCA-CCCAGAAACCCTTTGTGAACTTATACCT-TACTGTTGCCTCGGCG-CAGGCCGGCCTCT--CT----------TCTGAGGCC-----CCTCC---GGAAGGAGGAGCA-GCCCGCCGGCGGCCAG-CTAAACTCTTGTTTCT-TAGTGAATCTCTGAGT--AAAAAACACAAATGAATCAAAACTTTCAACAACGGATCTCTTGGTTCTGGCATCGATGAAGAACGCAGCGAAATGCGATAAGTAATGTGAATTGCAGAATTCAGTGAATCATCGAATCTTTGAACGCACATTGCGCCCTTTGGTATTCCGAAGGGCATGCCTGTTCGAGCGTCATTTCAACCCTCAAGCCTGGCTTGG-TGTTGGGGCACTGCTCCGA-------AGAGGAGTAGGCCCTGAAATCTAGTGGCGAGCT-CGCCAGGAC-CCCGAGCGTAGTAG-TTATA-TCTCGTTCTGG-AAGGCCCTGGCGGTGCCCTGCCGTTAAA-ACCCCAAATTCTGAAAATT

>Diaporthe_longispora_CBS_194_36

ACAAGGTCTCCGTTGGTGAACCAGCGGAGGGATCATTGCTGGAACGCG-CTTC-GGCGCA-CCCAGAAACCCTTTGTGAACTCATACCT-TACTGTTGCCTCGGCG-CAGGCCGGCCCCC---------------CTGGGGCC-----CCTCG---TTCCCGAGGAGCA-GCCCGCCGGCGGCCAA-CCAAACTCTTGTTTCT-TAGTGAGTCTCTGAGT--AAAAAACAAAAATAAATCAAAACTTTCAACAACGGATCTCTTGGTTCTGGCATCGATGAAGAACGCAGCGAAATGCGATAAGTAATGTGAATTGCAGAATTCAGTGAATCATCGAATCTTTGAACGCACATTGCGCCCTCTGGTATTCCGGAGGGCATGCCTGTTCGAGCGTCATTTCAACCCTCAAGCTCTGCTTGG-TGATGGGGCACCGCCCGTA--------AGAGGGCGGGCCCTGAAATCTAGTGGCGAGCT-CGCCAGGAC-CCCGAGCGTAGTAG-TTATA-TCTCGCCCTGG-AAGGCCCTGGCGGTGCCCTGCCGTTAAA-CCCCCAACTTCTGAAATTT

>Diaporthe_goulteri_BRIP_55657a

ACAAGGTCTCCGTTGGTGAACCAGCGGAGGGATCATTGCTGGAACGCG-CCCCTGGCGCA-CCCAGAAACCCTTTGTGAACTTATACCT--ACCGTTGCCTCGGCG-CAGGCCGGCCCCC--CT--------CACCGGGGGCC-----CCCCG---GAGACGGGGAGCA-GCCCGCCGGCGGCCAA-CCAAACTCTTGTTTCT-TAGTGAATCTCTGAGT--AAAAATCATAAATGAATCAAAACTTTCAACAACGGATCTCTTGGTTCTGGCATCGATGAAGAACGCAGCGAAATGCGATAAGTAATGTGAATTGCAGAATTCAGTGAATCATCGAATCTTTGAACGCACATTGCGCCCTCTGGTATTCCGGAGGGCATGCCTGTTCGAGCGTCATTTCAACCCTCAAGCCTGGCTTGG-TGATGGGGCACTGCCTTCGTA----ACAGAGGGCAGGCCCTGAAATCTAGTGGCGAGCT-CGCTAGGAC-CCCGAGCGTAGTAGTTTATA-TCTCGTTCTGG-AAGGCCCTGGCGGTGCCCTGCCGTTAAA-CCCCCAACTTCTGAAAATT

>Diaporthe_batatas_CBS_122_21

ACAAGGTCTCCGTTGGTGAACCAGCGGAGGGATCATTGCTGGAACGCG-CCCCTGGCGCA-CCCAGAAACCCTTTGTGAACTTATACC---ACTGTTGCCTCGGCG-CAGGCCGGCCTCT--TA----------GCTGAGGCC-----CCCCG---GAGACGGGGAGCA-GCCCGCCGGCGGCCAA-CCAAACTCTTGTTTCTATAGTGAATCTCTGAGT--AAAAAACATAAATGAATCAAAACTTTCAACAACGGATCTCTTGGTTCTGGCATCGATGAAGAACGCAGCGAAATGCGATAAGTAATGTGAATTGCAGAATTCAGTGAATCATCGAATCTTTGAACGCACATTGCGCCCTCTGGTATTCCGGAGGGCATGCCTGTTCGAGCGTCATTTCAACCCTCAAGCCTGGCTTGG-TGATGGGGCACTGCCTCCCTC-----AGGGGGGCAGGCCCTGAAATCTAGTGGCGAGCT-CGCCAGGAC-CCCGAGCGTAGTAG-TTACA-TCTCGCTCTGG-AAGGCCCTGGCGGTGCCCTGCCGTTAAA-CCCCCAACTTCTGAAAATT

>Diaporthe_chromolaenae_MFLUCC_17-1422

ACAAGGTCTCCGTTGGTGAACCAGCGGAGGGATCATTGCTGGAACGCG-CTTC-GGCGCA-CCCAGAAACCCTTTGTGAACTTATACCTAT--TGTTGCCTCGGCG-CAGGCCGGCCTCT--TC----------ACTGAGGCC-----CCCTG---GAAACAGGGAGCA-GCCCGCCGGCGGCCAA-CCAAACTCTTGTTTCTATAGTGAATCTCTGAGT-AAAAAAACATAAATGAATCAAAACTTTCAACAACGGATCTCTTGGTTCTGGCATCGATGAAGAACGCAGCGAAATGCGATAAGTAATGTGAATTGCAGAATTCAGTGAATCATCGAATCTTTGAACGCACATTGCGCCCTCTGGTATTCCGGAGGGCATGCCTGTTCGAGCGTCATTTCAACCCTCAAGCCTGGCTTGG-TGATGGGGCACTGCCTGTA--------ATAGGGCAGGCCCTGAAATCTAGTGGCGAGCT-CGCCAGGAC-CCCGAGCGTAGTAG-TTATA-TCTCGCTCTGG-AAGGCCCTGGCGGTGCCCTGCCGTTAAA-CCCCCAACTTCTGAAAATT

>Diaporthe_subellipicola_KUMCC_17_0153

ACAAGGTCTCCGTTGGTGAACCAGCGGAGGGATCATTGCTGGAACGCG-CTTC-GGCGCA-CCCAGAAACCCTTTGTGAACTTATACCTAT--TGTTGCCTCGGCG-CAGGCCGGCCTCT--TC----------ACTGAGGCC-----CCCTG---GAAACAGGGAGCA-GCCCGCCGGCGGCCAA-CCAAACTCTTGTTTCTATAGTGAATCTCTGAGT-AAAAAAACATAAATGAATCAAAACTTTCAACAACGGATCTCTTGGTTCTGGCATCGATGAAGAACGCAGCGAAATGCGATAAGTAATGTGAATTGCAGAATTCAGTGAATCATCGAATCTTTGAACGCACATTGCGCCCTCTGGTATTCCGGAGGGCATGCCTGTTCGAGCGTCATTTCAACCCTCAAGCCTGGCTTGG-TGATGGGGCACTGCCTGTA--------ATAGGGCAGGCCCTGAAATCTAGTGGCGAGCT-CGCCAGGAC-CCCGAGCGTAGTAG-TTATA-TCTCGCTCTGG-AAGGCCCTGGCGGTGCCCTGCCGTTAAA-CCCCCAACTTCTGAAAATT

>Diaporthe_heterostemmatis_SAUCC194_85

??????TCTCCGTTGGTGAACCAGCGGAGGGATCATTGCTGGAACGCG-CTTC-GGCGCA-CCCAGAAACCCTTTGTGAACTTATACCTAT--TGTTGCCTCGGCG-CAGGCCGGCCTCT--TC----------ACTGAGGCC-----CCCTG---GAAACAGGGAGCA-GCCCGCCGGCGGCCAA-CCAAACTCTTGTTTCTATAGTGAATCTCTGAGT-AAAAAAACATAAATGAATCAAAACTTTCAACAACGGATCTCTTGGTTCTGGCATCGATGAAGAACGCAGCGAAATGCGATAAGTAATGTGAATTGCAGAATTCAGTGAATCATCGAATCTTTGAACGCACATTGCGCCCTCTGGTATTCCGGAGGGCATGCCTGTTCGAGCGTCATTTCAACCCTCAAGCCTGGCTTGG-TGATGGGGCACTGCCTGTA--------ATAGGGCAGGCCCTGAAATCTAGTGGCGAGCT-CGCCAGGAC-CCCGAGCGTAGTAG-TTATA-TCTCGCTCTGG-AAGGCCCTGGCGGTGCCCTGCCGTTAAA-CCCCCAACTTCTGAAAATT

>Diaporthe_endophytica_CBS_133811

ACAAGGTCTCCGTTGGTGAACCAGCGGAGGGATCATTGCTGGAACGCG-CTTC-GGCGCA-CCCAGAAACCCTTTGTGAACTTATACCTAT--TGTTGCCTCGGCG-TAGGCCGGCCTCT--TC----------ACTGAGGCC-----CCCTG---GAAACAGGGAGCA-GCCCGCCGGCGGCCAA-CCAAACTCTTGTTTCTACAGTGAATCTCTGAGT--AAAAAACATAAATGAATCAAAACTTTCAACAACGGATCTCTTGGTTCTGGCATCGATGAAGAACGCAGCGAAATGCGATAAGTAATGTGAATTGCAGAATTCAGTGAATCATCGAATCTTTGAACGCACATTGCGCCCTCTGGTATTCCGGAGGGCATGCCTGTTCGAGCGTCATTTCAACCCTCAAGCCTGGCTTGG-TGATGGGGCACTGCCTGTA--------AAAGGGCAGGCCCTGAAATCTAGTGGCGAGCT-CGCCAGGAC-CCCGAGCGTAGTAG-TTATA-TCTCGCTCTGG-AAGGCCCTGGCGGTGCCCTGCCGTTAAA-CCCCCAACTTCTGAAAATT

>Diaporthe_fructicola_MAFF_246408

ACAAGGTCTCCGTTGGTGAACCAGCGGAGGGATCATTGCTGGAACGCG-CTTC-GGCGCA-CCCAGAAACCCTTTGTGAACTTATACCTAT--TGTTGCCTCGGCG-TAGGCCGGCCTCT--TC----------ACTGAGGCC-----CCCTG---GAAACAGGGAGCA-GCCCGCCGGCGGCCAA-CCAAACTCTTGTTTCTACAGTGAATCTCTGAGT--AAAAAACATAAATGAATCAAAACTTTCAACAACGGATCTCTTGGTTCTGGCATCGATGAAGAACGCAGCGAAATGCGATAAGTAATGTGAATTGCAGAATTCAGTGAATCATCGAATCTTTGAACGCACATTGCGCCCTCTGGTATTCCGGAGGGCATGCCTGTTCGAGCGTCATTTCAACCCTCAAGCCTGGCTTGG-TGATGGGGCACTGCCTGTA--------AAAGGGCAGGCCCTGAAATCTAGTGGCGAGCT-CGCCAGGAC-CCCGAGCGTAGTAG-TTATA-TCTCGCTCTGG-AAGGCCCTGGCGGTGCCCTGCCGTTAAA-CCCCCAACTTCTGAAAATT

>Diaporthe_masirevicii_BRIP_57892a

ACAAGGTCTCCGTTGGTGAACCAGCGGAGGGATCATTGCTGGAACGCG-CTTC-GGCGCA-CCCAGAAACCCTTTGTGAACTTATACCTAT--TGTTGCCTCGGCG-TAGGCCGGCCTCT--TC----------ACTGAGGCC-----CCCTG---GAAACAGGGAGCA-GCCCGCCGGCGGCCAA-CCAAACTCTTGTTTCTATAGTGAATCTCTGAGT--AAAAAACATAAATGAATCAAAACTTTCAACAACGGATCTCTTGGTTCTGGCATCGATGAAGAACGCAGCGAAATGCGATAAGTAATGTGAATTGCAGAATTCAGTGAATCATCGAATCTTTGAACGCACATTGCGCCCTCTGGTATTCCGGAGGGCATGCCTGTTCGAGCGTCATTTCAACCCTCAAGCCTGGCTTGG-TGATGGGGCACTGCCTGTA--------AGAGGGCAGGCCCTGAAATCTAGTGGCGAGCT-CGCCAGGAC-CCCGAGCGTAGTAA-TTATA-TCTCGCTCTGG-AAGGCCCTGGCGGTGCCCTGCCGTTAAA-CCCCCAACTTCTGAAAATT

>Diaporthe_schini_CBS_133181

ACAAGGTCTCCGTTGGTGAACCAGCGGAGGGATCATTGCTGGAACGCG-CTTC-GGCGCA-CCCAGAAACCCTTTGTGAACTTATACCTAT-CTGTTGCCTCGGCG-CAGGCCGGCTTCT--TC----------ACTGAAGCC-----CCCTG---GAGACAGGGAGCA-GCCCGCCGGCGGCCAA-CTAAACTCTTGTTTCTATAGTGAATCTCTGAGT--AAAAAACATAAATGAATCAAAACTTTCAACAACGGATCTCTTGGTTCTGGCATCGATGAAGAACGCAGCGAAATGCGATAAGTAATGTGAATTGCAGAATTCAGTGAATCATCGAATCTTTGAACGCACATTGCGCCCTCTGGTATTCCGGAGGGCATGCCTGTTCGAGCGTCATTTCAACCCTCAAGCCTGGCTTGG-TGATGGGGCACTGCCTGTA--------AAAGGGCAGGCCCTGAAATTCAGTGGCGAGCT-CGCCAGGAC-CCCGAGCGTAGTAG-TTATA-TCTCGCTTTGG-AAGGCCCTGGCGGTGCCCTGCCGTTAAA-CCCCCAACTTCTGAAAATT

>Diaporthe_terebinthifolii_CBS_133180

ACAAGGTCTCCGTTGGTGAACCAGCGGAGGGATCATTGCTGGAACGCG-CTTC-GGCGCA-CCCAGAAACCCTTTGTGAACTTATACCTAT-CTGTTGCCTCGGCGTCAGGCCGGCCTCT--TC----------ACTGAGGCC-----CCCTG---GAAACAGGGAGCA-GCCCGCCGGCGGCCAA-CTAAACTCTTGTTTCTATAGTGAATCTCTGAGT--AAAAAACATAAATGAATCAAAACTTTCAACAACGGATCTCTTGGTTCTGGCATCGATGAAGAACGCAGCGAAATGCGATAAGTAATGTGAATTGCAGAATTCAGTGAATCATCGAATCTTTGAACGCACATTGCGCCCTCTGGTATTCCGGAGGGCATGCCTGTTCGAGCGTCATTTCAACCCTCAAGCCTGGCTTGG-TGATGGGGCACTGCCTGTA--------AAAGGGCAGGCCCTGAAATCTAGTGGCGAGCT-CGCCAGGAC-CCCGAGCGTAGTAG-TTATA-TCTCGCTTTGG-AAGGCCCTGGCGGTGCCCTGCCGTTAAA-CCCCCAACTTCTGAAAATT

>Diaporthe_hordei_CBS_481_92

ACAAGGTCTCCGTTGGTGAACCAGCGGAGGGATCATTGCTGGAACGCG-CCTC-GGCGCA-CCCAGAAACCCTTTGTGAACTTATACCTAT-CTGTTGCCTCGGCG-CAGGCCGGCCCCC--TC----------ACCGGGGCC-----CCCTG---GAGACAGGGAGCA-GCCCGCCGGCGGCCAA-CCAAACTCTTGTTTCTACAGTGAATCTCTGAGT-AAAAAAACATAAATGAATCAAAACTTTCAACAACGGATCTCTTGGTTCTGGCATCGATGAAGAACGCAGCGAAATGCGATAAGTAATGTGAATTGCAGAATTCAGTGAATCATCGAATCTTTGAACGCACATTGCGCCCTCTGGTATTCCGGAGGGCATGCCTGTTCGAGCGTCATTTCAACCCTCAAGCCTGGCTTGG-TGATGGGGCACTGCCTGTG--------AAAGGGCAGGCCCTGAAATCTAGTGGCGAGCT-CGCCAGGAC-CCCGAGCGTAGTAG-TTATA-TCTCGCTCTGG-AAGGCCCTGGCGGTGCCCTGCCGTTAAA-CCCCCAACTTCTGAAAATT

>Diaporthe_kochmanii_BRIP_54033

???????????????????????????AGGGATCATTGCTGGAACGCG-CTTC-GGCGCA-CCCAGAAACCCTTTGTGAACTTATACCTAT--TGTTGCCTCGGCG-TAGGCCGGCCTCT--TC----------ACTGAGGCC-----CCCTG---GAAACAGGGAGCA-GCCCGCCGGCGGCCAA-CCAAACTCTTGTTTCTACAGTGAATCTCTGAGT--AAAAAACATAAATGAATCAAAACTTTCAACAACGGATCTCTTGGTTCTGGCATCGATGAAGAACGCAGCGAAATGCGATAAGTAATGTGAATTGCAGAATTCAGTGAATCATCGAATCTTTGAACGCACATTGCGCCCTCTGGTATTCCGGAGGGCATGCCTGTTCGAGCGTCATTTCAACCCTCAAGCCTGGCTTGG-TGATGGGGCACTGCTTTCGTC-----CAGAAAGCAGGCCCTGAAATCTAGTGGCGAGCT-CGCCAGGAC-CCCGAGCGTAGTAG-TTATA-TCTCGCTCTGG-AAGGCCCTGGCGGTGCCCTGCCGTTAAA-CCCCCAACTTCTGAAAATT

>Diaporthe_phaseolorum_CBS_113425

ACAAGGTCTCCGTTGGTGAACCAGCGGAGGGATCATTGCTGGAACGCG-CTTC-GGCGCA-CCCAGAAACCCTTTGTGAACTTATACCTAT--TGTTGCCTCGGCG-TAGGCCGGCCTCT--TC----------ACTGAGGCC-----CCCTG---GAAACAGGGAGCA-GCCCGCCGGCGGCCAA-CCAAACTCTTGTTTCTACAGTGAATCTCTGAGT--AAAAAACATAAATGAATCAAAACTTTCAACAACGGATCTCTTGGTTCTGGCATCGATGAAGAACGCAGCGAAATGCGATAAGTAATGTGAATTGCAGAATTCAGTGAATCATCGAATCTTTGAACGCACATTGCGCCCTCTGGTATTCCGGAGGGCATGCCTGTTCGAGCGTCATTTCAACCCTCAAGCCTGGCTTGG-TGATGGGGCACTGCTTTCGTC-----CAGAAAGCAGGCCCTGAAATCTAGTGGCGAGCT-CGCCAGGAC-CCCGAGCGTAGTAG-TTATA-TCTCGCTCTGG-AAGGCCCTGGCGGTGCCCTGCCGTTAAA-CCCCCAACTTCTGAAAATT

>Diaporthe_sojae_CBS_139282

???????????????????????GCGGAGGGATCATTGCTGGAACGCG-CTTC-GGCGCACCCCAGAAACCCTTTGTGAACTTATACCTAT--TGTTGCCTCGGCG-TAGGCCGGCCTCT--TC----------ACTGAGGCC-----CCCTG---GAAACAGGGAGCA-GCCCGCCGGCGGCCAA-CCAAACTCTTGTTTCTACAGTGAATCTCTGAGT--AAAAAACATAAATGAATCAAAACTTTCAACAACGGATCTCTTGGTTCTGGCATCGATGAAGAACGCAGCGAAATGCGATAAGTAATGTGAATTGCAGAATTCAGTGAATCATCGAATCTTTGAACGCACATTGCGCCCTCTGGTATTCCGGAGGGCATGCCTGTTCGAGCGTCATTTCAACCCTCAAGCCTGGCTTGG-TGATGGGGCACTGCTTTCGTC-----CAGAAAGCAGGCCCTGAAATCTAGTGGCGAGCT-CGCCAGGAC-CCCGAGCGTAGTAG-TCATA-TCTCGCTCTGG-AAGGCCCTGGCGGTGCCCTGCCGTTAAA-CCCCCAACTTCTGAAAATT

>Diaporthe_kongii_BRIP_54031

???????????????????????????AGGGATCATTGCTGGAACGCG-CTTC-GGCGCA-CCCAGAAACCCTTTGTGAACTTATACCTAT--TGTTGCCTCGGCG-TAGGCCGGCTTTT--TC-----------TAAAAGCC-----CCCTG---GAAACAGGGAGAA-GCCCGCCGGCGGCCAA-CCAAACTCTTGTTTCTACAGTGAATCTCTGAGT--AAAAAACATAAATGAATCAAAACTTTCAACAACGGATCTCTTGGTTCTGGCATCGATGAAGAACGCAGCGAAATGCGATAAGTAATGTGAATTGCAGAATTCAGTGAATCATCGAATCTTTGAACGCACATTGCGCCCTCTGGTATTCCGGAGGGCATGCCTGTTCGAGCGTCATTTCAACCCTCAAGCCTGGCTTGG-TGATGGGGCACTGCCTGTA--------AAAGGGCAGGCCCTGAAATCTAGTGGCGAGCT-CGCCAGGAC-CCCGAGCGTAGTAG-TTATA-TCTCGCTCTGG-AAGGCCCTGGCGGTGCCCTGCCGTTAAA-CCCCCAACTTCTGAAAATT

>Diaporthe_racemosae_CBS_143770

ACAAGGTCTCCGTTGGTGAACCAGCGGAGGGATCATTGCTGGAACGCG-CTTC-GGCGCA-CCCAGAAACCCTTTGTGAACTTATACCTAT-CTGTTGCCTCGGCG-CAGGCCGGCTTTTTGTG----------ACAAAAGCC-----CCCTG---GAGACAGGGAGCA-GCCCGCCGGCGGCCAA-CTAAACTCTTGTTTCTATAGTGAATCTCTGAGT--AAAAAACATAAATGAATTAAAACTTTCAACAACGGATCTCTTGGTTCTGGCATCGATGAAGAACGCAGCGAAATGCGATAAGTAATGTGAATTGCAGAATTCAGTGAATCATCGAATCTTTGAACGCACATTGCGCCCTCTGGTATTCCGGAGGGCATGCCTGTTCGAGCGTCATTTCAACCCTCAAGCCTGGCTTGG-TGATGGGGCACTGCCTGTA--------AAAGGGCAGGCCCTGAAATCTAGTGGCGAGCT-CGCCAGGAC-CCCGAGCGTAGTAG-TTATA-TCTCGCTTTGG-AAGGCCCTGGCGGTGCCCTGCCGTTAAA-CCCCCAACTCTTGAAAATT

>Diaporthe_infertilis_CBS_230_52

ACAAGGTCTCCGTTGGTGAACCAGCGGAGGGATCATTGCTGGAACGCG-CTTC-GGCGCA-CCCAGAAACCCTTTGTGAACTTATACCTA--CTGTTGCCTCGGCG-CAGGCCGGCCTTTTGTG----------ACAAAGGCC-----CCCTG---GAGACAGGGAGCA-GCCCGCCGGCGGCCAA-CTAAACTCTTGTTTCTATAGTGAATCTCTGAGT---AAAAACATAAATGAATCAAAACTTTCAACAACGGATCTCTTGGTTCTGGCATCGATGAAGAACGCAGCGAAATGCGATAAGTAATGTGAATTGCAGAATTCAGTGAATCATCGAATCTTTGAACGCACATTGCGCCCTCTGGTATTCCGGAGGGCATGCCTGTTCGAGCGTCATTTCAACCCTCAAGCCTGGCTTGG-TGATGGGGCACTGCCTGTT--------ACAGGGCAGGCCCTGAAATCTAGTGGCGAGCT-CGCCAGGAC-CCCGAGCGTAGTAG-TTATA-TCTCGCTTTGG-AAGGCCCTGGCGGTGCCCTGCCGTTAAA-CCCCCAACTTCTGAAAATT

>Diaporthe_longicolla_FAU_599

???????????????????????GCGGAGGGATCATTGCTGGAACGCG-CTTC-GGCGCA-CCCAGAAACCCTTTGTGAACTTATACCTA--CTGTTGCCTCGGCG-CAGGCCGGCCTTTTGTG----------ACAAAGGCC-----CCCTG---GAGACAGGGAGCA-GCCCGCCGGCGGCCAA-CCAAACTCTTGTTTCTACAGTGAATCTCTGAGT--ACAAAACATAAATGAATCAAAACTTTCAACAACGGATCTCTTGGTTCTGGCATCGATGAAGAACGCAGCGAAATGCGATAAGTAATGTGAATTGCAGAATTCAGTGAATCATCGAATCTTTGAACGCACATTGCGCCCTCTGGTATTCCGGAGGGCATGCCTGTTCGAGCGTCATTTCAACCCTCAAGCCTGGCTTGG-TGATGGGGCACTGCTCTCT------GACGGGAGCAGGCCCTGAAATCTAGTGGCGAGCT-CGCTAGGAC-CCCGAGCGTAGTAG-TTATA-TCTCGTTCTGG-AAGGCCCTGGCGGTGCCCTGCCGTTAAA-CCCCCAACTTCTGAAAATT

>Diaporthe_passifloricola_CBS_141329

ACAAGGTCTCCGTTGGTGAACCAGCGGAGGGATCATTGCTGGAACGCG-CCTC-GGCGCA-CCCAGAAACCCTTTGTGAACTTATACCTAT-TTGTTGCCTCGGCG-CCGGCCGGCCTTTTGTG----------ACAAAGGCC-----CCCTG---GAGACAGGGAGCA-GCCCGCCGGCGGCCAA-CTAAACTCTTGTTTCTATAGTGAATCTCTGAGT---AAAAACATAAATGAATCAAAACTTTCAACAACGGATCTCTTGGTTCTGGCATCGATGAAGAACGCAGCGAAATGCGATAAGTAATGTGAATTGCAGAATTCAGTGAATCATCGAATCTTTGAACGCACATTGCGCCCTCTGGTATTCCGGAGGGCATGCCTGTTCGAGCGTCATTTCAACCCTCAAGCCTGGCTTGG-TGATGGGGCACTGCCTTCT------AGCGAGGGCAGGCCCTGAAATCTAGTGGCGAGCT-CGCTAGGAC-CCCGAGCGTAGTAG-TTATA-TCTCGTTCTGG-AAGGCCCTGGCGGTGCCCTGCCGTTAAA-CCCCCAACTTCTGAAAATT

>Diaporthe_vochysiae_LGMF1583

ACAAGGTCTCCGTTGGTGAACCAGCGGAGGGATCATTGCTGGAACGCG-CTTC-GGCGCA-CCCAGAAACCCTTTGTGAACTTATACCTAT-CTGTTGCCTCGGCG-CAGGCCGGCCTTTTGTG----------ACAAAGGCC-----CCCTG---GAGACAGGGAGCA-GCCCGCCGGCGGCCAA-CCAAACTCTTGTTTCTGTAGTGAATCTCTGAGT--AAAAAACATAAATGAATCAAAACTTTCAACAACGGATCTCTTGGTTCTGGCATCGATGAAGAACGCAGCGAAATGCGATAAGTAATGTGAATTGCAGAATTCAGTGAATCATCGAATCTTTGAACGCACATTGCGCCCTCTGGTATTCCGGAGGGCATGCCTGTTCGAGCGTCATTTCAACCCTCAAGCCTGGCTTGG-TGATGGGGCACTGCCTTCT------AACGAGGGCAGGCCCTGAAATCTAGTGGCGAGCT-CGCTAGGAC-CCCGAGCGTAGTAG-TTATA-TCTCGTTCTGG-AAGGCCCTGGCGGTGCCCTGCCGTTAAA-CCCCCAACTTCTGAAAATT

>Diaporthe_miriciae_BRIP_54736j

ACAAGGTCTCCGTTGGTGAACCAGCGGAGGGATCATTGCTGGAACGCG-CTTC-GGCGCA-CCCAGAAACCCTTTGTGAACTTATACCTAT-TTGTTGCCTCGGCG-TAGGCCGGCCTCT--TC----------ACTGAGGCC-----CCCTG---GAGACAGGGAGCA-GCCCGCCGGCGGCCAA-CTAAACTCTTGTTTCTATAGTGAATCTCTGAGT--AAAAAACATAAATGAATCAAAACTTTCAACAACGGATCTCTTGGTTCTGGCATCGATGAAGAACGCAGCGAAATGCGATAAGTAATGTGAATTGCAGAATTCAGTGAATCATCGAATCTTTGAACGCACATTGCGCCCTCTGGTATTCCGGAGGGCATGCCTGTTCGAGCGTCATTTCAACCCTCAAGCCTGGCTTGG-TGATGGGGCACTGCCTTCT------AGCGAGGGCAGGCCCTGAAATCTAGTGGCGAGCT-CGCTAGGAC-CCCGAGCGTAGTAG-TTATA-TCTCGTTCTGG-AAGGCCCTGGCGGTGCACTGCCGTTAAA-CCCCCAACTTCTGAAAATT

>Diaporthe_ueckerae_FAU_656

???????????????????????GCGGAGGGATCATTGCTGGAACGCG-CTTC-GGCGCA-CCCAGAAACCCTTTGTGAACTTATACCTAT-TTGTTGCCTCGGCC-TAGGCCGGCCTCT--TC----------ACTGAGGCC-----CCCTG---GAGACAGGGAGCA-GCCCGCCGGCGGCCAA-CTAAACTCTTGTTTCTATAGTGAATCTCTGAGT--AAAAAACATAAATGAATCAAAACTTTCAACAACGGATCTCTTGGTTCTGGCATCGATGAAGAACGCAGCGAAATGCGATAAGTAATGTGAATTGCAGAATTCAGTGAATCATCGAATCTTTGAACGCACATTGCGCCCTCTGGTATTCCGGAGGGCATGCCTGTTCGAGCGTCATTTCAACCCTCAAGCCTGGCTTGG-TGATGGGGCACTGCCTTCT------AGCGAGGGCAGGCCCTGAAATCTAGTGGCGAGCT-CGCTAGGAC-CCCGAGCGTAGTAG-TTATA-TCTCGTTCTGG-AAGGCCCTGGCGGTGCACTGCCGTTAAA-CCCCCAACTTCTGAAAATT

>Diaporthe_rosae_MFLUCC_17_2658

ACAAGGTCTCTGTTGGTGAACCAGCGGAGGGATCATTGCTGGAACGCG-CTTC-GGCGCA-CCCAGAAACCCTTTGTGAACTTATACCTAT-CTGTTGCCTCGGCG-CAGGCCGGCCTCT--TC----------ACTGAGGCC-----CCCTG---GAAACAGGGAGCA-GCCCGCCGGCGGCCAA-CTAAACTCTTGTTTCTATAGTGAATCTCTGAGT--AAAAAACATAAATGAATCAAAACTTTCAACAACGGATCTCTTGGTTCTGGCATCGATGAAGAACGCAGCGAAATGCGATAAGTAATGTGAATTGCAGAATTCAGTGAATCATCGAATCTTTGAACGCACATTGCGCCCTCTGGTATTCCGGAGGGCATGCCTGTTCGAGCGTCATTTCAACCCTCAAGCCTGGCTTGG-TGATGGGGCACTGCCTTCT------AACGAGGGCAGGCCCTGAAATCTAGTGGCGAGCT-CGCTAGGAC-CCCGAGCGTAGTAG-TTATA-TCTCGTTCTGG-AAGGCCCTGGCGGTGCCCTGCCGTTAAA-CCCCCAACTTCTGAAAATT

>Diaporthe_rosiphthora_COAD_2913

??????????????GGTGAACCAGCGGAGGGATCATTGCTGGAACGCG-CTTC-GGCGCA-CCCAGAAACCCTTTGTGAACTTATACCTAT-CTGTTGCCTCGGCG-CAGGCCGGCTTCT--TC----------ACTGAAGCC-----CCCTG---GAGACAGGGAGCA-GCCCGCCGGCGGCCAA-CTAAACTCTTGTTTCTACAGTGAATCTCTGAGT--AAAAAACATAAATGAATCAAAACTTTCAACAACGGATCTCTTGGTTCTGGCATCGATGAAGAACGCAGCGAAATGCGATAAGTAATGTGAATTGCAGAATTCAGTGAATCATCGAATCTTTGAACGCACATTGCGCCCTCTGGTATTCCGGAGGGCATGCCTGTTCGAGCGTCATTTCAACCCTCAAGCCTGGCTTGG-TGATGGGGCACTACTTCCT------TACGGGAGTAGGCCCTGAAATTCAGTGGCGAGCT-CGCCAGGAC-CCCGAGCGTAGTAG-TTATA-TCTCGCTTTGG-AAGGCCCTGGCGGTGCCCTGCCGTTAAA-CCCCCAACTTCTGAAAATT

>Diaporthe_tectonendophytica_MFLUCC_13_0471

ACAAGGTCTCCGTTGGTGAACCAGCGGAGGGATCATTGCTGGAACGCG-CTTC-GGCGCA-CCCAGAAACCCTTTGTGAACTTATACCTA--CTGTTGCCTCGGCG-CAGGCCGGCCTCT--TC----------GCTGAGGCC-----CCCTG---GAGACAGGGAGCA-GCCCGCCGGCGGCCAA-CTAAACTCTTGTTTCTTTAGTGAATCTCTGAGT--AAAAAACATAAATGAATCAAAACTTTCAACAACGGATCTCTTGGTTCTGGCATCGATGAAGAACGCAGCGAAATGCGATAAGTAATGTGAATTGCAGAATTCAGTGAATCATCGAATCTTTGAACGCACATTGCGCCCTCTGGTATTCCGGAGGGCATGCCTGTTCGAGCGTCATTTCAACCCTCAAGCCTGGCTTGG-TGATGGGGCACTGCTCTCT------AGCGGGAGCAGGCCCTGAAATCTAGTGGCGAGCT-CGCCAGGAC-CCCGAGCGTAGTAG-TTATA-TCTCGTTCTGG-AAGGCCCTGGCGGTGCCCTGCCGTTAAA-CCCCCAACTTCTGAAAATT

>Diaporthe_unshiuensis_CGMCC3_17569

??????????????????????????????????????CTGGAACGCG-CTTC-GGCGCA-CCCAGAAACCCTTTGTGAACTTATACCTAT--TGTTGCCTCGGCG-CAGGCCGGCCTCT--TC----------ACTGAGGCC-----CCCTG---GAGACAGGGAGCA-GCCCGCCGGCGGCCAA-CCAAACTCTTGTTTCTACAGTGAATCTCTGAGT--ACAAAACATAAATGAATCAAAACTTTCAACAACGGATCTCTTGGTTCTGGCATCGATGAAGAACGCAGCGAAATGCGATAAGTAATGTGAATTGCAGAATTCAGTGAATCATCGAATCTTTGAACGCACATTGCGCCCTCTGGTATTCCGGAGGGCATGCCTGTTCGAGCGTCATTTCAACCCTCAAGCCTGGCTTGG-TGATGGGGCACTGCTCTCT------GACGAGAGCAGGCCCTGAAATCTAGTGGCGAGCT-CGCTAGGAC-CCCGAGCGTAGTAG-TTATA-TCTCGTTCTGG-AAGGCCCTGGCGGTGCCCTGCCGTTAAA-CCCCCAACTTCTGAAAATT

>Diaporthe_megalospora_CBS_143_27

ACAAGGTCTCCGTTGGTGAACCAGCGGAGGGATCATTGCTGGAACGCG-CTTC-GGCGCA-CCCAGAAACCCTTTGTGAACTTATACCTA--CTGTTGCCTCGGCG-CAGGCCGGCCTCC--CT----------TCCGAGGCC-----CCCTG---GGAACAGGGAGCA-GCCCGCCGGCGGCCGA-CCAGACTCTTGTTTCTGTAGTGGATCTCTGAGT-ACAAAAACACAAATGAATCAAAACTTTCAACAACGGATCTCTTGGTTCTGGCATCGATGAAGAACGCAGCGAAATGCGATAAGTAATGTGAATTGCAGAATTCAGTGAATCATCGAATCTTTGAACGCACATTGCGCCCTCTGGTATTCCGGAGGGCATGCCTGTTCGAGCGTCATTTCAACCCTCAAGCCTGGCTTGG-TGATGGGGCACTGCCCGTA--------ACAGGGCAGGCCCTGAAATCCAGTGGCGAGCT-CGCCAGGAC-CCCGAGCGTAGTAG-TTACA-TCTCGCTCCGG-AAGGCCCTGGCGGTGCCCTGCCGTTAAA-CCCCCAACTCCTGAAAATT

>Diaporthe_guangdongensis_ZHKUCC20_0014

????????TCCGTAGGTGAACCTGCGGAGGGATCATTGCTGGAACGCG-CCTC-GGCGCA-CCCAGAAACCCTTTGTGAACTTATACCTA--CTGTTGCCTCGGCG-CAGGCCGGCCTTTGTCA----------AAGAAGGCC-----CCCTG---GGAACAGGGAGCA-GCCCGCCGGCGGCCAA-CTAAACTCTTGTTTCTATAGTGAATCTCTGAGT-AAAAAAACATAAATGAATCAAAACTTTCAACAACGGATCTCTTGGTTCTGGCATCGATGAAGAACGCAGCGAAATGCGATAAGTAATGTGAATTGCAGAATTCAGTGAATCATCGAATCTTTGAACGCACATTGCGCCCTCTGGTATTCCGGAGGGCATGCCTGTTCGAGCGTCATTTCAACCCTCAAGCCTGGCTTGG-TGATGGGGCACTGCTCTCTC------GCGGGAGCAGGCCCTGAAATCTAGTGGCGAGCT-CGCCAGGAC-CCCGAGCGTAGTAG-TTACA-TCTCGCTCTGG-AAGGCCCTGGCGGTGCCCTGCCGTTAAA-CCCCCAACTCCTGAAAATT

>Diaporthe_melonis_CBS_507_78

ACAAGGTCTCCGTTGGTGAACCAGCGGAGGGATCATTGCTGGAACGCG-CCTC-GGCGCA-CCCAGAAACCCTTTGTGAACTTATACCTA--CTGTTGCCTCGGCG-CAGGCCGGCCTTTGTCA----------AAGAAGGCC-----CCCTG---GAGACAGGGAGCA-GCCCGCCGGCGGCCAA-CTAAACTCTTGTTTCTATAGTGAATCTCTGAGT---AAAAACATAAATGAATCAAAACTTTCAACAACGGATCTCTTGGTTCTGGCATCGATGAAGAACGCAGCGAAATGCGATAAGTAATGTGAATTGCAGAATTCAGTGAATCATCGAATCTTTGAACGCACATTGCGCCCTCTGGTATTCCGGAGGGCATGCCTGTTCGAGCGTCATTTCAACCCTCAAGCCTGGCTTGG-TGATGGGGCACTGCTCTCTC------GCGGGAGCAGGCCCTGAAATCTAGTGGCGAGCT-CGCCAGGAC-CCCGAGCGTAGTAG-TTACA-TCTCGCTCTGG-AAGGCCCTGGCGGTGCCCTGCCGTTAAA-CCCCCAACTTCTGAAAATT

>Diaporthe_vexans_CBS_127_14

ACAAGGTCTCCGTTGGTGAACCAGCGGAGGGATCATTGCTGGAACGCG-CCTC-GGCGCA-CCCAGAAACCCTTTGTGAACTTATACCTAT--TGTTGCCTCGGCG-CAGGCCGGCCTCTCCTG----------GCAGAGGCC-----CCCTG---GAGACAGGGAGCA-GCCCGCCGGCGGCCAG-CTAAACTCTTGTTTCTACAGTGAATCTCTGAGT---AAAAACATAAATGAATCAAAACTTTCAACAACGGATCTCTTGGTTCTGGCATCGATGAAGAACGCAGCGAAATGCGATAAGTAATGTGAATTGCAGAATTCAGTGAATCATCGAATCTTTGAACGCACATTGCGCCCTCTGGTATTCCGGAGGGCATGCCTGTTCGAGCGTCATTTCAACCCTCAAGCCTGGCTTGG-TGATGGGGCACTGCCTGTG--------AAAGGGCAGGCCTTGAAATCTAGTGGCGAGCT-CGCCAGGAC-CCCGAGCGTAGTAG-TATTA-TCTCGCCCTGG-AAGGCCCTGGCGGTGCCCTGCCGTTAAACCCCCCAACTCCTGAAAATT

>Diaporthe_ovalispora_ICMP20659

??????????????????????????????????????CTGGAACGCG-CCTC-GGCGCA-CCCAGAAACCCTTTGTGAACTTATACCTA--CTGTTGCCTCGGCG-CAGGCCGGCTTTTT-TT----------CTAAAAGCC-----CCCTG---GAAACAGGGAGCA-GCCCGCCGGCGGCCGA-CCAAACTCTTGTTTCTACAGTGGATCTCTGAGA--AAAAAACATAAATGAATCAAAACTTTCAACAACGGATCTCTTGGTTCTGGCATCGATGAAGAACGCAGCGAAATGCGATAAGTAATGTGAATTGCAGAATTCAGTGAATCATCGAATCTTTGAACGCACATTGCGCCCTCTGGTATTCCGGAGGGCATGCCTGTTCGAGCGTCATTTCAACCCTCAAGCCTGGCTTGG-TGATGGGGCACTGCCTTCGCC-----CAGGAGGCAGGCCCTGAAATCTAGTGGCGAGCT-CGCCAGGAC-CCCGAGCGCAGTAG-TCATA-TCTCGCTCTGG-AAGGCCCTGGCGGTGCCCTGCCGTTAAA-CCCCCAACTTCTGAAAATT

>Diaporthe_convolvuli_CBS_124654

ACAAGGTCTCCGTTGGTGAACCAGCGGAGGGATCATTGCTGGAACGCG-CCTC-GGCGCA-CCCAGAAACCCTTTGTGAACTTATACCTATACTGTTGCCTCGGCG-CAGGCCGGCCTCC--CC----------ACCGAGGCC-----CCCTG---GAGACAGGGAGCA-GCCCGCCGGCGGCCAA-CCAAACTCTTGTTTCTACAGTGGATCTCTGAGT--AAAAAACATAAATGAATCAAAACTTTCAACAACGGATCTCTTGGTTCTGGCATCGATGAAGAACGCAGCGAAATGCGATAAGTAATGTGAATTGCAGAATTCAGTGAATCATCGAATCTTTGAACGCACATTGCGCCCTCTGGTATTCCGGAGGGCATGCCTGTTCGAGCGTCATTTCAACCCTCAAGCCTGGCTTGG-TGATGGGGCGCTGCCTGTA-------AGACGGGCAGGCCCTGAAATCTAGTGGCGAGCT-CGCCAGGAC-CCCGAGCGTAGTAG-TTATA-TCTCGCTCCGGAAAGGCCCTGGCGGTGCCCTGCCGTTAAA-CCCCCAACTTCTGAAAATT

>Diaporthe_tecomae_CBS_100547

ACAAGGTCTCCGTTGGTGAACCAGCGGAGGGATCATTGCTGGAACGCG-CCTC-GGCGCA-CCCAGAAACCCTTTGTGAACGTATACCTAC-CTGTTGCCTCGGCGTTAGGCCGGCCTTTTGTG----------ACAAAGGCC-----CCCTG---GAGACAGGGAGCA-GCCCGCCGGCGGCCAA-CTAAACTCTTGTTTCTATAGTGAATCTCTGAG---AAAAAACATAAATGAATCAAAACTTTCAACAACGGATCTCTTGGTTCTGGCATCGATGAAGAACGCAGCGAAATGCGATAAGTAATGTGAATTGCAGAATTCAGTGAATCATCGAATCTTTGAACGCACATTGCGCCCTCTGGTATTCCGGAGGGCATGCCTGTTCGAGCGTCATTTCAACCCTCAAGCCTGGCTTGG-TGATGGGGCACTGCTTTTGTT-----ATAAAAGCAGGCCCTGAAATTCAGTGGCGAGCT-CGCCAGGAC-CCCGAGCGTAGTAG-TTATA-TCTCGCTTTTG-GAGGCCCTGGCGGTGCCCTGCCGTTAAA-CCCCCAACTTCTGAAAATT

>Diaporthe_helianthi_CBS_592_81

ACAAGGTCTCCGTTGGTGAACCAGCGGAGGGATCATTGCTGGAACGCGCCCCC-GGCGCA-CCCAGAAACCCTTTGTGAACTTATACCTAT-CTGTTGCCTCGGCG-CAGGCCGGCCCCC---C----------CTGGGGGCC-----CCCTG---GGAACAGGGAGCA-GCCCGCCGGCGGCCGA-CCAAACTCTTGTTTCTACAGTGGATCTCTGAGT--TAAAAACACAAATGAATCAAAACTTTCAACAACGGATCTCTTGGTTCTGGCATCGATGAAGAACGCAGCGAAATGCGATAAGTAATGTGAATTGCAGAATTCAGTGAATCATCGAATCTTTGAACGCACATTGCGCCCTCTGGTATTCCGGAGGGCATGCCTGTTCGAGCGTCATTTCAACCCTCAAGCCTGGCTTGG-TGATGGGGCACTGCCTGTG--------ACAGGGCAGGCCCTGAAATCCAGCGGCGAGCC-CGCCGGGAC-CCCGAGCGTAGTAG-TAACT-TCTCGCTCCGG-AAGGCCCTGGCGGCGCCCTGCCGTTAAA-CCCCCAACTCCTGAAAATT

>Diaporthe_thunbergiicola_MFLUCC_12-0033

ACAAGGTCTCCGTTGGTGAACCAGCGGAGGAATCATTGCGAGCGCGCA-TTCC--GCGCA-CCGAGAAACCCTTTGTGAACTTATACCT-TACTGTTGCCTCGGCG-CAGGCCGGCCTCT--------------GCTGAGGCC-----CCCCG---GAGACGGGGAGCA-GCCCGCCGGCGGCCAA-CCAAACTCTTGTTTCTACAGTGAATCTCTGAGT--AAAAAACATAAATGAATCAAAACTTTCAACAACGGATCTCTTGGTTCTGGCATCGATGAAGAACGCAGCGAAATGCGATAAGTAATGTGAATTGCAGAATTCAGTGAATCATCGAATCTTTGAACGCACATTGCGCCCTCTGGTATTCCGGAGGGCATGCCTGTTCGAGCGTCATTTCAACCCTCAAGCCTGGCTTGG-TGTTGGGGCACTGCTCCGA--------GAGGAGCAGGCCCTGAAATCTAGTGGCGAGCT-CGCTAGGAC-CCCGAGCGTAGTAG-TTATA-TCTCGTTCTGG-AAGGCCCTGGCGGTGCCCTGCCGTTAAA-CCCCCAACTTCTGAAATTT

>Diaporthe_mayteni_CBS_133185

ACAAGGTCTCCGTTGGTGAACCAGCGGAGGGATCATTGCTGGAACGCG-CCCC-GGCGCA-CCCAGAAACCCTTTGTGAACCTATACCT-TACTGTTGCCTCGGCG-CAGGCCGGCCTCC--------------CGTGAGGCC-----CCTCG---GAGACGAGGAGCA-GCCCGCCGGCGGCCAA-GCAAACTCTTGTTTCT-TAGTGGATCTCTGAGT--AAAAAACACAAATGAATCAAAACTTTCAACAACGGATCTCTTGGTTCTGGCATCGATGAAGAACGCAGCGAAATGCGATAAGTAATGTGAATTGCAGAATTCAGTGAATCATCGAATCTTTGAACGCACATTGCGCCCCCTGGTATTCCGGGGGGCATGCCTGTTCGAGCGTCATTTCACCCCTCAAGCCTGGCTTGG-TGCTGGGGCACTACTCCCTC------GCCGGAGTAGGCCCTGAAATCCAGCGGCGAGCC-CTATGGGTA-ACCGAGTGCAGTAG-T--AT-TCTCGCTCTGG-GATGCCCTGGCGGCGC---GCCGTTAAA-CCCCCAACTCCTGAGATTT

>Diaporthe_myracrodruonis_URM7972

NNNNNNNNNNNNNNNNNNNACCTGCGGAGGGATCATTGCTGGAACGCG-CCCC-GGCGCA-CCCAGAAACCCTTTGTGAACTTATACGTA--CTGTTGCCTCGGCG-CAGGCCGGCTCTG--AG----------ATACGAGCC-----CCCCG---GAGACGGGGAGAA-GCCCGCCGGCGGCCAA-GCAAACTCTTGTTTCTACAGTGGATCTCTGAGT--AAAAAACATAAATGAATCAAAACTTTCAACAACGGATCTCTTGGTTCTGGCATCGATGAAGAACGCAGCGAAATGCGATAAGTAATGTGAATTGCAGAATTCAGTGAATCATCGAATCTTTGAACGCACATTGCGCCCCCTGGTATTCCGGGGGGCATGCCTGTTCGAGCGTCATTTCACCCCTCAAGCCTGGCTTGG-TGATGGGGCACTGCCCGTA--------ACAAGGCAGGCCCTGAAATCCAGCGGCGCACCACTGTGGAAA-ACCGAGCGTAGTAG-TTATG-TCTCGCCCGGT-ACGCCCGCAGCGGT--CCTGCCGTTAAA-CCCCCAACTCCTGAAAATT

>Diaporthe_sinensis_ZJUP0033_4

NNNNNNNNNNNNNNNNNNNNNNNNNNNNNNNNNNNNNNNNNNNNNNNNNNNNNNNNNNNNNNNNNNNNNNNNNNNNNNNNNNNNNNNNNNNNNNNNNNNNNNNNNNNNNNNNNNNNNNNNNNNNNNNNNNNNNNNNNCCCCCC-----CCCCC---CGGGGGGGGAGCA-GCCCGCCGGCGGCCAACCCAAACTCCTGATTCTGCAGTGGATCTCTGAGC-AAAAAAACACAAATGAATCAAAACTTTCAACAACGGATCTCTTGGTTCTGGCATCGATGAAGAACGCAGCGAAATGCGATAAGTAATGTGAATTGCAGAATTCAGTGAATCATCGAATCTTTGAACGCACATTGCGCCCTCCGGCATTCCGGAGGGCATGCCTGTTCGAGCGTCATTTCAACCCTCAAGCCTGGCTTGG-TGATGGGGCGCTGCCCGTA--------GAAGGGCAGGCCCTGAAATCTAGTGGCGGGCC-CGCCGGGAC-CCCGAGCGTAGTAGCCTACA-CCTCGCTCCGG-GAGGCCCCGGCGGTGCCCTGCCGTTAAA-CCCCCAACACCCGAAATCT

>Diaporthe_amygdali_CBS_126679

ACAAGGTCTCCGTTGGTGAACCAGCGGAGGGATCATTGCTGGAACGCG-CCTC-GGCGCA-CCCAGAAACCCTTTGTGAACTTATACCT-TACTGTTGCCTCGGCG-CAGGCCGGCTCCC--AT----------CTGGGGGCC-----CCTCGTTTCTGACGAGGAGCAGGCTCGCCGGCGGCCAA-GTTAACTCTTGTTTTTAATTTGAAACTCTGAGA--ATAAAACATAAATGAATCAAAACTTTCAACAACGGATCTCTTGGTTCTGGCATCGATGAAGAACGCAGCGAAATGCGATAAGTAATGTGAATTGCAGAATTCAGTGAATCATCGAATCTTTGAACGCACATTGCGCCCTCTGGTATTCCGGAGGGCATGCCTGTTCGAGCGTCATTTCAACCCTCAAGCCTGGCTTGG-TGATGGGGCACTGCCTTTGTGTAAAAGCGAAGGCAGGCCCTGAAATTCAGTGGCGAGCT-CGCCAGGAC-TCCGAGCGCAGTAG-TTAAACCCTCGCTTTGG--AAGGACTGGCGGTGCCCTGCCGTTAAA-CCCCCAACTCTTGAAAATT

>Diaporthe_eres_CBS_138594

???????????????????????GCGGAGGGATCATTGCTGGAACGCG-CCCCAGGCGCA-CCCAGAAACCCTTTGTGAACTTATACCT-TACTGTTGCCTCGGCG-CTAGCTGGTCCCT----------------CGGGGCCCCTCACCCTC---GGGTGTTGAGACA-GCCCGTCGGCGGCCAA-CCTAACTCTTGTTTTTACACTGAAACTCTGAGC--ACAAAACATAAATGAATCAAAACTTTCAACAACGGATCTCTTGGTTCTGGCATCGATGAAGAACGCAGCGAAATGCGATAAGTAATGTGAATTGCAGAATTCAGTGAATCATCGAATCTTTGAACGCACATTGCGCCCTCTGGTATTCCGGAGGGCATGCCTGTTCGAGCGTCATTTCAACCCTCAAGCCTGGCTTGG-TGATGGGGCACTGCTTCTTAC----CCAAGAAGCAGGCCCTGAAATTCAGTGGCGAGCT-CGCCAGGAC-CCCGAGCGCAGTAG-TTAAACCCTCGCTCTGG-AAGGCCCTGGCGGTGCCCTGCCGTTAAA-CCCCCAACTTCTGAAAATT

>Diaporthe_celtidis_NCYU_19_0357

NNNNNNNNTCCGTTGGTGAACCAGCGGAGGGA-CATTGCTGGAACGCG-CCCC-GGCGCA-CCCAGAAACCCTTTGTGAACTTATACCTATACCGTTGCCTCGGCG-CTGGCCGGCCCCC--TC----------ACCGGGGCC-----CCCTG---GAGACAGGGAGCA-GCCCGCCGGCGGCCAG-ACAAACTCTTGTTTCT-TAGTGGATCTCTGAGT--AAAAAACAT-AATGAATCAAAACTTTCAACAACGGATCTCTTGGTTCTGGCATCGATGAAGAACGCAGCGAAATGCGATAAGTAATGTGAATTGCAGAATTCAGTGAATCATCGAATCTTTGAACGCACATTGCGCCCCCTGGTATTCCGGGGGGCATGCCTGTTCGAGCGTCATTTCACCCCTCAAGCCTGGCTTGG-TGTTGGGGCACCGCCCGTA--------AAAGGGCGGGCCCTTAAATCCAGCGGCGAGCC-AGAGGAAACGGCCTTGCGTAGTAG-TTCTA-TCTCGCATGTC-CTGCCCTCA--AGCGCCCTGCCGTTAAA-CCCCANNNNNNNNNNNNNN

>Diaporthe_durionigena_VTCC_930005

?????????????????????????????????????????????????????????????????????CCCTTTGTGAACTTATACCTAT-CTGTTGCCTCGGCG-TAGGCCGGCCTCT--TC----------ACTGAGGCC-----CCCTG---GAAACAGGGAGCA-GCCCGCCGGCGGCCAA-CTAAACTCTTGTTTCTATAGTGAATCTCTGAGT---AAAAACATAAATGAATCAAAACTTTCAACAACGGATCTCTTGGTTCTGGCATCGATGAAGAACGCAGCGAAATGCGATAAGTAATGTGAATTGCAGAATTCAGTGAATCATCGAATCTTTGAACGCACATTGCGCCCTCTGGTATTCCGGAGGGCATGCCTGTTCGAGCGTCATTTCAACCCTCAAGCCTGGCTTGG-TGATGGGGCACTGCCTTCT------AGCGAGGGCAGGCCCTGAAATCTAGTGGCGAGCT-CGCTAGGAC-CCCGAGCGTAGTAG-TTATA-TCTCGTTCTGG-AAGGCCCTGGCGGTGCCCTGCCGTTAAA-CCCCCAACTTCTGAAAATT

>Diaporthe_breyniae

ACAAGGTCTCCGTTGGTGAACCAGCGGAGGGATCATTGCTGGAACGCG-CTTC-GGCGCA-CCCAGAAACCCTTTGTGAACTTATACCTAT--TGTTGCCTCGGCG-TAGGCCGGCCTCT--TC----------ACTGAGGCC-----CCCTG---GAGACAGGGAGCA-GCCCGCCGGCGGCCAA-CTAAACTCTTGTTTCTATAGTGAATCTCTGAGT---AAAAACATAAATGAATCAAAACTTTCAACAACGGATCTCTTGGTTCTGGCATCGATGAAGAACGCAGCGAAATGCGATAAGTAATGTGAATTGCAGAATTCAGTGAATCATCGAATCTTTGAACGCACATTGCGCCCTCTGGTATTCCGGAGGGCATGCCTGTTCGAGCGTCATTTCAACCCTCAAGCCTGGCTTGG-TGATGGGGCACTGCTCTCT------AGCGGGAGCAGGCCCTGAAATCTAGTGGCGAGCT-CGCCAGGAC-CCCGAGCGTAGTAG-TTATA-TCTCGTTCTGG-AAGGCCCTGGCGGTGCCCTGCCGTTAAA-CCCCCAACTTCTGAAAATT

# Alignment of the *cal* sequences used in the second phylogenetic study

>Diaporthe_acericola_MFLUCC_17_0956

NNNNNNNNNNNNNNNNNNNNNNNNNNNNNNNNNNNNNCCTCGAGC-----TCCCCCGCCAT--GTTCTGCCTTTGCGCATG--ATGCTAACGGACCGTTTTCGGCCTGCAGGATAAGGATGGCGATGGTTAGTGCGGTCACCGCT----TCCTTCCCTCT-TTCTCAGCTACCCACGCGTCATACTCGATCCGCCGCGACGGTCTGCGCG-TGCAGTATACTC-TGAGCGAGCG-AACCTCAT----------ATCGATCACGAGAAATATGCTAAGAC--GGC-GTGTAGGACAAATCACCACCAAGGAGCTCGGCACAGTCATGCGGTCCCTTGGTCAAAACCCTTCCGAGTCCGAGCTGCAGGACATGATCAACGAGGTCGACGCCGACAACAATGGCACCATTGACTTCCCTGGTAAGTCTACATGT-CCACCCA-CTGAATATT

>Diaporthe_novem_CBS_127271

CTTCTCCCTCTTTGTAAGTTATTCTC---GCCA---GCCTCGAGC----TCCCCCCGCCAT--GTTCTGCCTTTGCGCATG--ATGCTAACGGACCGTTTTCGGCCTGCAGGATAAGGATGGCGATGGTTAGTGCGGTCACCGCT----TCCTTCCCTCT-TTCTCAGCTACCCACGCGTCATACTCGATCCGCCGCGACGGTCTGCGCA-TGCAGTACACTC-TGAGCGAGCG-AACCTCAT----------ATCGATCACGAGAAATATGCTAAGAC--GGC-GTGTAGGACAAATCACCACCAAGGAGCTCGGCACAGTCATGCGGTCCCTTGGTCAAAACCCTTCCGAGTCCGAGCTGCAGGACATGATCAACGAGGTCGACGCCGACAACAATGGCACCATTGACTTCCCTGGTAAGTCTACATGT-CCACCCA-CTGAATATT

>Diaporthe_cuppatea_CBS_117499

NNNNNNNNNNNNNNNNNNNNNNNNNNNNNNNNNNNNNNNNNNNNNNNNNNNNNNNNGCCAT--GTTCTGCCTTTGCGCATG--ATGCTAACGGACCGTTTTCGGCCTGCAGGATAAGGATGGCGATGGTTAGTGCGGTCACCGCT----TCCTTCCCTCT-TTCTCAGCTACCCACGCGTCATATTCGATCCGCCGCGACGGTCTGCGCG-TGCAGTACACTC-TGAGCGAGCG-AACCTCAC----------ATCGATCACGAGAAATATGCTAAGAC--GGC-GTGTAGGACAAATCACCACCAAGGAGCTCGGCACAGTCATGCGGTCCCTGGGTCAAAACCCTTCCGAGTCCGAGCTGCAGGACATGATCAACGAGGTCGATGCCGACAACAATGGCACCATTGACTTCCCTGGTAAGTCTAGATGC-CCACCCG-CTGAATATT

>Diaporthe_lusitanicae_CBS_123212

CTTCTCCCTCTTTGTAAGTTATTCTC---ACCT---GCCCTGAGC----TCCCCCCGCCAT--GTTCTGCCTTTGCGCATG--ATGCTAACGGACCGTTTTCGGCCTGCAGGATAAGGATGGCGATGGTTAGTGCGGTCACCGCT----TCCTTCCCTCT-TTCTCAGCTACCCACGCGTCATACTCGATCCGCCTCGACGGTCTGCGCG-TGCAGTACACTC-TGAGCGAGCG-AACCTCAT----------ATCGACCACGAGAAACATGCTAAGAC--GGC-GTGTAGGACAAATCACCACCAAGGAGCTCGGCACAGTTATGCGGTCCCTTGGTCAAAACCCTTCCGAGTCCGAGCTGCAGGACATGATCAACGAGGTCGACGCCGACAACAATGGCACCATTGACTTCCCTGGTAAGTCTAGATGT-CCACCCG-CTGAATATT

>Diaporthe_neoarctii_CBS_109490

CTTCTCCCTCTTTGTAAGTTATTCTC---ACCA---GCCTTGAGC----TCCCCACGCCGT--GTTCTGCCTTTGCGCATG--ATGCTAACGGACCGTTTTCGGCCTGCAGGATAAGGATGGTGATGGTTAGTGCGGTCACCGCT----TCCCTCCCTCT-TTCTCAGCTACCCACGCGTCATACTCGATCCGCCGCGACGATCTGCGCG-TGCAGTACATCC-TGAGCGAGCG-AACCTCGT----------ATCGATCACGAGAAATATGCTAAGAC--GGC-GTGTAGGACAAATCACCACCAAGGAGCTCGGCACAGTCATGCGGTCCCTTGGTCAAAACCCTTCCGAGTCCGAGCTGCAGGACATGATCAACGAGGTCGACGCCGACAACAATGGCACCATTGACTTCCCTGGTAAGTCTAGATGT-CCACCCA-CTGGATGTT

>Diaporthe_angelicae_CBS_111592

CTTCTCCCTCTTTGTAAGTTATTCTC---ACCA---GCCTTGAGC----TCCCCCCGCCAT--GTTCTGCTGTTGCGCATG--ATGCTAACGGACCGTTTTCGGCCTGCAGGATAAGGATGGCGATGGTTAGTGCGGTCACCGCT----TCCTTCCCTCT-CTCTCAGCTACCCACGCGTCATACTCGATCCGCCGCGACGGTCTGCGCG-TGCAGTACACTCTTGAGCGAGCG-AGCCTCAT----------ATCGATCACGAGAAATATGCTAAGAC--GGC-GTGTAGGACAAATCACCACCAAGGAGCTCGGCACAGTCATGCGGTCCCTTGGTCAAAACCCTTCCGAGTCCGAGCTGCAGGACATGATCAACGAGGTCGACGCCGACAACAATGGCACCATTGACTTCCCTGGTAAGTCTAGATGT-CCACCCA-CTGGATATT

>Diaporthe_arctii_CBS_136_25

NNNNNNNNNNNNNNNNNNNNNNNNNC---ACCA---GCCTTGAGC----TCCCCCCGCCAT--GTTCTGCCTTTGCGCATG--ATGCTAACGGACCGTTTTCGGCCTGCAGGATAAGGATGGCGATGGTTAGTGCGGTCACCGCT----TCCTCCCCTCT-CTCTCAGCTACCCACGCGTCATACTCGATCCGCCGCGACGGTCTGCGCG-TGCAGTACACTCTTGAGCGAGCG-AGCCTCAT----------ATCGATCACGAGAAATATGCTAAGAC--GGC-GTGTAGGACAAATCACCACCAAGGAGCTCGGCACAGTCATGCGGTCCCTTGGTCAAAACCCTTCCGAGTCCGAGCTGCAGGACATGATCAACGAGGTCGACGCCGACAACAATGGCACCATTGACTTCCCTGGTAAGTCTAAATGT-CCACCCA-CTGGATATT

>Diaporthe_subordinaria_CBS_101711

CTTCTCCCTCTTTGTAAGTTATTCTC---ACCA---GCCTTGAGC----TCCCCCCGCCAT--GTTCTGCCTTTGCGCATG--ATGCTAACGGACCGTTTTCGGCCTGCAGGATAAGGATGGCGATGGTTAGTGCGGTCACCGCT----TCCTCCCCTCT-CTCTCAGCTACCCACGCGTCATACTCGATCCGCCGCGACGGTCTGCGCG-TGCAGTACACTCTTGAGCGAGCG-AACCTCAT----------ATCGATCACGAGAAATATGCTAAGAC--GGC-GTGTAGGACAAATCACCACCAAGGAGCTCGGCACAGTCATGCGGTCCCTTGGTCAAAACCCTTCCGAGTCCGAGCTGCAGGACATGATCAACGAGGTCGACGCCGACAACAATGGCACCATTGACTTCCCTGGTAAGTCTAGATAT-CCACCCA-CTGGATATT

>Diaporthe_cichorii_MFLUCC_17_1023

NNNNNNNNNNNNNNNNNNNNNNNNNNNNNNNNNNNNNCCTTGAGCCCCTCCCCCCCGCCAT--GTTCTGCCTTTGCGCTTG--ATGCTAACGGACCGTTTTCGGCCTGCAGGATAAGGATGGCGATGGTTAGTGCGGTCACCGCT----TCCTTCCCTCT-TTCTCAGCTACCCACGCGTCATACTCGATCCGCCGCGACGGTCTGCGCG-TGCAGTACACTCTTGAGCGAGCA-AACCTCAT----------ATCGATCACGAGAAATATGCTAAGAC--GGC-GTGTAGGACAAATCACCACCAAGGAGCTCGGCACAGTCATGCGGTCCCTTGGTCAAAACCCTTCCGAGTCCGAGCTGCAGGACATGATCAACGAGGTCGACGCCGACAACAATGGCACCATTGACTTCCCTGGTAAGTCTAGATGT-CCACCCA-CTGGATATT

>Diaporthe_schoeni_MFLU_15_1279

NNNNNNNNNNNNNNNNNNNNNNNNNNNNNNNNNNNNNNCTTAGCT-----CCCCCCGCCAT--GTTCTGCTGTTGCGCATG--ATGCTAACGGACCGTTTTCGGCCTGCAGGATAAGGATGGCGATGGTTAGTGCGGTCACCGCT----TCCTCCCCTCT-CTCTCAGCTACCCACGCGTCATACTCGATCCGCCGCGACGGTCTGCGCG-TGCAGTACACTCTTGAGCGAGCG-AACCTCAC----------ATCGATCACGAGAAATATGCTAAGAC--GGC-GTGTAGGACAAATCACCACCAAGGAGCTCGGCACAGTCATGCGGTCCCTTGGTCAAAACCCTTCCGAGTCCGAGCTGCAGGACATGATCAACGAGGTCGACGCCGACAACAACGGCACCATTGACTTCCCTGGTGAGTCTAGATAT-CCACCCA-CTG-ATATT

>Diaporthe_guttulata_CGMCC_3_20100

NNNNNNNNNNNNNNNNNNNNNNNNNNNNNNNNNNNNNNNNNNNNNNNNNNNNNCCCGCCGT--GTTCTGCCTTTGCGCATG--ATGCTAACGGACCGTTTTCGGCCTGCAGGATAAGGATGGCGATGGTTAGTGCGGTCACCGCT----TCCTCCCCTCT-CTCTCAGCTACCCACGCGTCATACTCGATCCGCCGCGACGGTCTGCGCG-TGCAGTACACTCTTGAGCGAGCG-AACCTCAT----------ATCGATCACGAGAAACATGCTAAGAC--GGC-GTGTAGGACAAATCACCACCAAGGAGCTCGGCACGGTCATGCGATCCCTGGGTCAGAACCCGTCCGAGTCTGAGCTGCAAGATATGATTAACGAGGTCGACGCCGACAACAATGGCACCATTGACTTCCCTGGTAAGTCTAGATGT-CCACCCA-CTGNNNNNN

>Diaporthe_caryae_CFCC_52563

NNNNNNNNNNNNNNNNNNNNNNNNNNNNNNNNNNNNNNNNNNNNNNNNNNNNNNCCGCCAC--ACTGTGCTTTTGCGCATG--ATGCTAACGGACCGTTTTCGGCCTGCAGGATAAGGATGGCGATGGTTAGTGTGGTCACCGCC----TTCTTCCCTCT-TTCTCAGCTACGCACGCGTCATATTCGATCCGCCGCGACGGTCTGCGCG-CGCAGCATGCTC-CGAGCGACCG-ATCATCAC----------GTCTATCACGAGTAACATGCTAAGTC--GGC-GTGTAGGACAAATTACCACCAAGGAGCTCGGCACAGTCATGCGGTCCCTTGGTCAAAACCCTTCCGAGTCCGAGCTGCAGGACATGATCAACGAGGTCGACGCCGACAACAATGGCACCATTGACTTCCCTGGTAAGTCTGGATGC-TTATCCC-CTAGATGTT

>Diaporthe_infecunda_CBS_133812

CTTCTCCCTCTTTGTAAGTTATCTTC---GGAG---GCCTGGAGC-----TTCCCCGCCGT--CCTGTGCTTTTGCGCATG--ATGCTAACGGACCGTTTTCGGCCTGCAGGATAAGGATGGCGATGGTTAGTGTGGTCACCGCT----TTCTTCCCTCT-TGCTCAGCTACGCACGCGTCATACTCGATCCGCCGCGACGGTCTGCGCG-CGCAGCATGCTC-CGAGCGACCG-ATCATCAC----------GTCTATCACGAGTAACATGCTAAGTC--GGC-GTGTAGGACAAATCACCACCAAGGAGCTCGGCACAGTCATGCGGTCCCTTGGTCAAAACCCTTCCGAGTCCGAGCTGCAGGACATGATCAACGAGGTCGACGCCGACAACAATGGCACCATTGACTTCCCTGGTAAGTCTGGATGC-TCATCCC-CTAGACGTT

>Diaporthe_leucospermi_CBS_111980

NNNNNNNNTCTTTGTAAGTTATCTTC---GGAG---GCCTTGAGC-----TTCCCCGCCAT--CCTGTGCTTTTGCGCATG--ATGCTAACGGACCGTTTTCGGCCTGCAGGATAAGGATGGCGATGGTTAGTGTGGTCACCGCT----TTCTCCCCTCT-TTCTCAGCTACGCACGCGTCATACTCGATCCGCCGCGACGGTCTGCGCG-TGCAGCATGCTC-CGAGCGACCG-ATCATCAC----------GTCTATCACGAGTAACATGCTAAGTC--GGC-GTGTAGGACAAATCACCACCAAGGAGCTCGGCACAGTCATGCGGTCCCTTGGTCAAAACCCTTCCGAGTCCGAGCTGCAGGACATGATCAACGAGGTCGACGCCGACAACAATGGCACCATTGACTTCCCTGGTAAGTCTGGATGC-TCATCCC-CTAGACATT

>Diaporthe_pyracanthae_CBS142384

NNNNNNNNTCTTTGTAAGTTATCTTC---GGAG---GCCTTGAGC-----TTCCCCGCCAT--CCTGTGCTTTTGCGCATG--ATGCTAACGGACCGTTTTCGGCCTGCAGGATAAGGATGGCGATGGTTAGTGTGGTCACCGCT----TTCTCCCCTCT-TTCTCAGCTACGCACGCGTCATACTCGATCCGCCGCGACGGTCTGCGCG-TGCAGCATGCTC-CGAGCGACCG-ATCATCAC----------GTCTATCACGAGTAACATGCTAAGTC--GGC-GTGTAGGACAAATCACCACCAAGGAGCTCGGCACAGTCATGCGGTCCCTTGGTCAAAACCCTTCCGAGTCCGAGCTGCAGGACATGATCAACGAGGTCGACGCCGACAACAATGGCACCATTGACTTCCCTGGTAAGTCTGGATGC-TCATCCC-CTAGACATT

>Diaporthe_rossmaniae_CAA762

NNNNNNNNTCTTTGTAAGTTATCTTC---GGAG---GCCTTGAGC-----TTCCCCGCCAT--CCTGTGCTTTTGCGCATG--ATGCTAACGGACCGTTTTCGGCCTGCAGGATAAGGATGGCGATGGTTAGTGTGGTCACCGCT----TTCTCCCCTCT-TTCTCAGCTACGCACGCGTCATACTCGATCCGCCGCGACGGTCTGCGCG-TGCAGCATGCTC-CGAGCGACCG-ATCATCAC----------GTCTATCACGAGTAACATGCTAAGTC--GGC-GTGTAGGACAAATCACCACCAAGGAGCTCGGCACAGTCATGCGGTCCCTTGGTCAAAACCCTTCCGAGTCCGAGCTGCAGGACATGATCAACGAGGTCGACGCCGACAACAATGGCACCATTGACTTCCCTGGTAAGTCTGGATGC-TCATCCC-CTAGACATT

>Diaporthe_myracrodruonis_URM7972

NNNNNNNNNNNNNNNNNNNNNNNNNNNNNNNNNNNNNNNNNNNNC-----TTCCCCGCCAT--CCTGTGCTTTTGCGCATG--ATGCTAACGGACCGTTTTCGGCCTGCAGGATAAGGATGGCGATGGTTAGTGTGGTCACCGCT----TTCTTCCCTCT-TTCTCAGCTACGCACGCGTCATACTCGATCCGCCGCGACGGTCTGCGCG-TGCAGCATGCTC-CGAGCGACCG-ACCATCAC----------GTCTATCACGAGTAACATGCTAAGTC--GGC-GTGTAGGACAAATCACCACCAAGGAGCTCGGCACAGTCATGCGGTCCCTTGGTCAAAACCCTTCCGAGTCCGAGCTGCAGGACATGATCAACGAGGTCGACGCCGACAACAATGGCACCATTGACTTCCCTGGTAAGTCTTGATGC-TCATACC-CTAAATGTT

>Diaporthe_machili_SAUCC194_111

NNNNNNNNNNNNNNNNNNNNTGAATC---TCGA---GGCTTGAGC------TTCCCGCCAC--ACTGTGCTTTTGCGCATG--ATGCTAACGGACCGTTTTCGGCCTGCAGGATAAGGATGGCGATGGTTAGTGTGGTCACCGCT----TTCTTCCCTCT-TTCTCAGCTACGCACGCGTCATACTCGATCCGCCGCGACGGTCTGCGCG-CGCAGCATGCTC-CGAGCGACCG-ATCATCAC----------GTCTATCACGAGTAACATGCTAAGTC--GGC-GTGTAGGACAAATTACCACCAAGGAGCTCGGCACAGTCATGCGGTCCCTTGGTCAAAACCCTTCCGAGTCCGAGCTGCAGGACATGATCAACGAGGTCGACGCCGACAACAATGGCACCATTGACTTCCCTGGTAAGCCTGGATGC-TTATCCC-CTAGACGTC

>Diaporthe_pachirae_CDA_728

NNNNNNNNNNNNNNNNNNNNNNNNNNNNNNNNNNNNNNNNNNNNNNNNNNNNNNNNNNNNNNNCCTGTGCTTTTGCGCATG--ATGCTAACGGACCGTTTTCGGCCTGCAGGATAAGGATGGCGATGGTTAGTGTGGTCACCGCT----TTCTCCCCTCT-TTCTCAGCTACGCACGCGTCATACTCGATCCGCCGCGACGGTCTGCGCG-TGCAGCATGCTC-CGAGCGACCG-ATCATCAC----------GTCTATCACGAGTAACATGCTAAGTC--GGG-GTGTAGGACAAATCACCACCAAGGAGCTCGGCACAGTCATGCGGTCCCTTGGTCAAAACCCTTCCGAGTCCGAGCTGCAGGACATGATCAACGAGGTCGACGCCGACAACAATGGCACCATCGACTTCCCTGGTAAGTCTGGATGC-TCATCCC-CTAGACGTT

>Diaporthe_ganjae_CBS_180_91

CTTCTCCCTCTTTGTAAGTTATATCC---AGCA---GCCTTGGGC-----TCCCCCGCCGT--CCCCTGCTGTTGCGCATG--ATGCTAACGGACCGTTTTCGGCCTGCAGGATAAGGATGGCGATGGTTAGTGTGGTCACCGCT----TTCTTCCCTCC-TCCTCAGCCACGCACGCGTCATGCTCGATCCGCCGCGACGGTCTGCGCG-TGCATTATGCCC-AGAGCGATCGAATCATCAC----------ACCCATGACGAGCACCATGCTAAGAC--GGC-GTGCAGGACAAATCACCACCAAGGAGCTCGGCACGGTCATGCGGTCCCTGGGCCAAAACCCCTCCGAGTCCGAGCTGCAGGACATGATCAACGAGGTCGACGCCGACAACAACGGCACCATTGACTTCCCTGGTAGGTCCACATGTCCCTCCCA-CTGGATGTT

>Diaporthe_manihotia_CBS_505_76

CTTCTCCCTCTTTGTAAGTCATATCC---AGCA---GCCTCGGGC-----TCCCCTGTCGT--CCTCTGCTGTTGCGCATG--ATGCTAACGGACCGTTCTCGGCCTGCAGGATAAGGATGGCGATGGTTAGTGTGGTCACCGCT----TTCTTCCCTCC-TCCTAAGCCACGCACGCGTCATGCTCGATCCGCCGCGACGGTCTGCGCG-TGCATTATGCCC-CGAGCGACCGAATCATCAC----------ATCTATCACGAGTACCATGCTAAGAC--GGC-GTGCAGGACAAATCACCACCAAGGAGCTCGGCACGGTCATGCGGTCCCTGGGCCAAAACCCCTCCGAGTCCGAGCTGCAGGACATGATCAACGAGGTCGACGCCGACAACAACGGCACCATTGACTTCCCTGGTAGGTTCACATGT-CCACCCA-CTGGATGTT

>Diaporthe_sambucusii_CFCC_51986

NNNNNNNNTCTTTGTAAGTTATATCC---AGCA---ACCTTGGGC-----CCCCCCGCCGT--CCTCTGCTGTTGCGCATG--ATGCTAACGGACCGTTTTCGGCCTGCAGGATAAGGATGGCGATGGTTAGTGTGGTCACCGCT----TTCTTCCCTCC-TCCTCAGCCACGCACGCGTCATGCTCGATCCGCCGCGACGGTCTGCGCG-TGCATTATGCCC-CGAGCGATCGAATCATCAC----------ACCCATCACGAGTACCATGCTAAGAC--GGC-GTGCAGGACAAATCACCACCAAGGAGCTCGGCACGGTCATGCGGTCCCTGGGCCAAAACCCCTCCGAGTCCGAGCTGCAGGACATGATCAACGAGGTCGACGCCGACAACAACGGCACCATTGACTTCCCTGGTAGGTCCACATGT-CCACCCA-CTGGATGTT

>Diaporthe_ambigua_CBS_114015

CTTCTCCCTCTTTGTAAGTTATCTTC---TGTA---GCCTTGAGC----CTCTCCCGCCGT--GCTTTGCCGTTGCGCATG--ATGCTAACGGCCCGTTTTCGGCCTGCAGGATAAGGATGGCGATGGTTAGTGCAGTCACCACG----TTCTTCCCTCT-TTCCCAGCTACGCACGCGTCACACTCGATCCGCCGCGACGGTCTGCGCG-TGCA-TATACTC-CAGACGACCG-ACCATCAC----------ATCCATCTCGAGTGCCATGCTAAGAC--GGC-GTGTAGGACAAATCACCACCAAGGAGCTCGGCACGGTCATGCGTTCCCTGGGCCAGAACCCCTCCGAGTCCGAGCTGCAGGATATGATTAACGAGGTCGACGCCGACAACAATGGCACCATTGACTTCCCTGGTAAGTCCAGATGC-TCGCGCA-CTGAAAATT

>Diaporthe_longispora_CBS_194_36

NNNNNNNNNNNNNNNNNNNNNNNNNNNNNGGCA---GCCTTGAGC-----CTCCCCGCCGT--CCGCTGCTGTCGCGCATG--ATGCTAACGGACCGTTTTCGGCCTGCAGGATAAGGATGGCGATGGTTAGTGTGGTCACAACT----TTCTTCCCTCT-TTACTAGCTACGCATGCGTCACACTCGATCCCCCGCGACGGTCTGCGCGTTGCA-TATACTC-CGAGCGACCG-ATCGTCAC----------ATCTGTCA-GGATACCATGCTAAGAC--GGC-GTGTAGGACAAATCACCACCAAGGAGCTCGGCACGGTCATGCGTTCCCTGGGTCAAAACCCCTCCGAGTCCGAGCTGCAGGATATGATCAACGAGGTCGACGCCGACAACAATGGCACCATTGACTTCCCTGGTAAGTCTAGATGC-TCGCCCA-CCGGATGTT

>Diaporthe_sclerotioides_CBS_296_67

CTTCTCCCTCTTTGTAAGTCATCTTC---AGCA---GCCTTGGGC-----CTCCCCGCCGC--CCGCTGCCGTCGCGCATG--ATGCTAACCGACCGTTTTCGGCCTGCAGGATAAGGATGGCGATGGTTAGTGTGGCCACCACT----TTCTTCCCTCT-TTGCTAGCTACGCACGCGTCACACTCGATCCGCCGCGACGGTCTGCGCG-TGCA-TATACTC-CGAGCGACCG-ATCGTCAC----------ATCCATCA-GGATACCATGCTAAGAC--GGC-GTGTAGGACAAATCACCACCAAGGAGCTCGGCACGGTCATGCGTTCCCTCGGTCAAAACCCCTCCGAGTCTGAGCTGCAGGATATGATCAACGAGGTCGACGCCGACAACAATGGCACCATTGACTTCCCTGGTAAGTCTAGATGC-TTGCCCA-CCGGGTGTT

>Diaporthe_chinensis_MFLUCC_19_0101

NNNNNNNNNNNNNNNNNNNNNNNNNNNNNNNNNNNNNNNNNNNNNNNNNNNNNCCCGCCCT--GCTCTGCTGTCGCGCATG--ATGCTAACGGACCGTTTTCGGCTCGCAGGATAAGGATGGCGATGGTTAGTGCAGCCACCACT----TCTCTCTCTCTTTTCCCAACTACGCACGCGTCACTCTTGATCCGCTACGACGGTCTACGCG-TGCA-TATACTC-CAACCGACCG-ATCATCAC----------ATCCATCACGAGTACCATGCTAAGAC--GGC-GTGTAGGACAAATCACCACCAAGGAGCTCGGCACGGTCATGCGGTCCCTGGGTCAAAACCCCTCCGAGTCCGAGCTGCAGGATATGATCAATGAGGTCGACGCCGACAACAATGGCACCATTGACTTCCCTGGTAAGTCTAGATGC-TCGTATA-CTGAATGTT

>Diaporthe_yunnanensis_CGMCC_3_18289

CTTCTCCCTCTTTGTAAGTCATCTTA---ACTA---GCCTCAAGC-----CTCCCCGCCCT--GCTCTGCTGTCGCGCATG--ATGCTAACGGACCGTTTTCGGCTCGCAGGATAAGGATGGCGATGGTTAGTGCAGCCACCACTTCTCTCTCTCTTTCTCTTTCCAACTACGCACGCGTCACTCTTGATCCGCTACGACGGTCTACGCG-TGCA-TATACTC-CAACCGACCG-ATCATCAC----------ATCCATCACGAGTACCATGCTAAGAC--GGC-GTGTAGGACAAATCACCACCAAGGAGCTCGGCACGGTCATGCGGTCCCTGGGTCAAAACCCCTCCGAGTCCGAGCTGCAGGATATGATCAATGAGGTCGACGCCGACAACAATGGCACCATTGACTTCCCTGGTAAGTCTAGATGC-TCGCATA-CTGAATGTT

>Diaporthe_citriasiana_CBS_134240

CTTCTCCCCCTTCGTAAGTCACCATC---AGTA---GCCTCAAGC-----CTCCCCGCCCT--GCTCTGCTGTCGCGCATG--ATGCTAACGGACCGTTTTCGGATCGCAGGATAAGGATGGCGATGGTTAGTGCAACCACCTCT----TTCTTCTCTCTTTTTCCAACTACGCACGCGTCACGCTTGATCCGCTGCGACGGTCTGCGCG-TGTA-GATACTC-CAAGCGACCG-AACATCGC----------ATCCATCACGAGTATCATACTAAGAC--GGC-GCGTAGGACAAATCACCACCAAGGAACTCGGCACGGTCATGCGGTCCCTGGGTCAAAACCCCTCCGAGTCCGAGCTGCAGGATATGATCAATGAGGTCGACGCCGACAACAATGGCACCATTGACTTCCCTGGTAAGTCCAGATGC-TCGCTTA-CTGAACGGT

>Diaporthe_alangii_CFCC_52556

NNNNNNNNNNNNNNNNNNNNNNNNNNNNNNNNNNNNNNNNNNNNNNNNNNNNNNCCGCCAC--GCTCTGCTGTT---CATG--ATGCTAACGGACCGTTTTCGGCCCGCAGGATAAGGATGGCGATGGTTAGTGTGGTCACCACC----TTCTTCCCTCT-TCCTCAGCTACGCACGCGTCATGCTCGATCCGCCGCGACGGCCTGCGCG-TGCA-TATAATC-CAAGCGACCG-ATCATCG-----------ATCCATCACCAGTACCATGCTAAGAC--GGC-GTGCAGGACAAATCACCACCAAGGAGCTAGGCACGGTCATGCGGTCCCTGGGTCAAAACCCCTCCGAGTCTGAGCTGCAGGACATGATTAACGAGGTCGATGCCGACAACAATGGCACCATTGACTTCCCTGGTAAGCCAAGATGC-TCGCCCG-CCGAGTGTT

>Diaporthe_hubeiensis_JZB320123

CTTTTCCCTCTTTGTAAGTCATTTCC---AGCCGGCAGACATGAG-----CTCCCCGCCCT--CCTCTGCTGTTGTCCATA--ATGCTAACGGACCGTTTTCGGCCCGCAGGATAAGGATGGCGATGGTTAGTGTGGTCACCACC----TTCTTCCCTCT-TCCTCAGCTACGCACGCGTCATGCTCGAACCGCCGCGACGGCCTGCGCT-TGCA-TATAATC-CAAGCGACCG-ATCATCG-----------ATCCATCACCAGTACCATGCTAAGACG-GGC-GTGCAGGACAAATCACCACCAAGGAGCTCGGCACGGTCATGCGGTCCCTGGGTCAAAACCCCTCCGAGTCTGAGCTGCAGGACATGATTAACGAGGTCGATGCCGACAACAATGGCACCATTGACTTCCCTGGTAAGCCAAGATGC-TCGCCCG-CCGAGTGTT

>Diaporthe_tectonae_MFLUCC_12_0777

NNNNNNNNNNNNNNNNNNNNNNNNNNNNNNNNNNNTGACCCTGAG-----CCTCCCGCCAC--GCTCTGCTGTTGTCCATG--ATGCTAACGGACCGTTTTCGGCCCGCAGGATAAGGATGGCGATGGTTAGTGTGGTCACCACC----TTCTTCCCTCT-TCCTCAGCTACGCACGCGTCATGCTCGATCCGCCGCGACGGCCTGCGCG-TGCA-TATAATC-CAAGCGACCG-ATCATCG-----------ATCCATCACCAGTACCATGCTAAGACG-GGC-GTGCAGGACAAATCACCACCAAGGAGCTCGGCACGGTCATGCGGTCCCTGGGTCAAAACCCCTCCGAGTCTGAGCTGCAGGACATGATTAACGAGGTCGATGCCGACAACAATGGCACCATTGACTTCCCTGGTAAGCCAAGATGC-TCGCCCG-CCGAGTGTT

>Diaporthe_cerradensis_CMRP4331

NNNNNNNNNNNNNNNNNNNNNNNNNNNNNNNNNNNNNNNNTTGAC-----TTCTCCGCCGT--CCGCTGCTGTTGCGCATG--ATGCTAACGGCCCGTTTTCGGCCTGCAGGATAAGGATGGCGATGGTTAGTGTTGTCACCACT----TTCTCTCCTCT-TTCTCAGCTACGCACGCGTCGGGCTCGACCCGCCGCGACGGCCTGCGCG-TGCA-TATACCC-TAAGCGACCG-AACGTCAC----------A-ATATGACGAGTATCAAGCTAAGGC--GGC-GTGTAGGACAAATCACCACCAAGGAGCTCGGCACTGTCATGCGGTCCCTGGGTCAAAACCCCTCCGAGTCCGAGCTGCAGGACATGATCAACGAGGTCGACGCCGACAACAACGGCACCATTGACTTCCCTGGTAAGTTCAAATGC-TCGCCTG-CTGAATATA

>Diaporthe_mayteni_CBS_133185

CTTCTCCCTCTTTGTAAGCTGTCTTC---GCTA---GCCTTGAGC-----CACCCCGCCGT--CCGCTGTTGTTGCGCCTC--ATGCTAACGGACCGTTTTCGGCCTGCAGGATAAGGATGGCGATGGTTAGTGTGGTCACCACT----TTGTT-CCTCT-TTCCCAGCCACGCACGCGTCACACACGATCCGCCGCGA-GGTCTGCGCG-TTCA-TAAACCC-CAGGCGACCG-ATCACAAT----------ATCTATCACAAGTACCATGCTAAGAC--GGC-GTGTAGGACAAATCACCACCAAGGAGCTCGGCACTGTCATGCGCTCCCTGGGTCAAAACCCCTCCGAGTCCGAGCTGCAGGATATGATCAACGAGGTCGACGCCGACAACAACGGCACTATTGACTTCCCTGGTAAGTCCAGATGC-TCGCCTA-CTGAATATT

>Diaporthe_neoraonikayaporum_MFLUCC_14_1136

NNNNNNNNNNNNNNNNNNNNNNNNNNNNNNNCA---GCTTTGGGC-----CTGCCCGCCGT--CCACTGCTGTCGCGCATG--ATGCTAACGGACCGTTTTCGGCCTGCAGGATAAGGATGGCGATGGTTAGTGTGATCACCACT----TTCTTCCCTA--GTCCTAGCTACGCACGCCTCACACTTGGCCCGCCGCGACGGTCTGCGCG-TGCA-TGT-----CAAGCGACCG-AATACT------------ACCCATCACGAGTATCATGCTAAGAC--GAC-GTGTAGGACAAATCACCACCAAGGAGCTCGGCACTGTCATGCGGTCCCTAGGTCAAAACCCGTCCGAGTCCGAGCTGCAGGATATGATCAACGAGGTTGACGCCGACAACAACGGCACCATTGACTTCCCTGGTAAGCCCAGATGC-TCGCCTA-CCGGATATT

>Diaporthe_raonikayaporum_CBS_133182

CTTCTCCCTCTTTGTAAGTTGTCTTC---ACCA---GCTTTGGGC-----CTGCCCGCCGT--CCACTGCTGTCGCGCATG--ATGCTAACGGACCGTTTTCGGCCTGCAGGATAAGGATGGCGATGGTTAGTGTGATCACCACT----TTCTTCCCTA--GTCCTAGCTTCGCACGCCTCACGCTTGGCCCGCCGCGACGGTCTGCGCG-TGCA-TGT-----CAAGCGACCG-AATACT------------ACCCATCACGAGTATCATGCTAAGAC--GAC-GTGTAGGACAAATCACCACCAAGGAGCTCGGCACTGTCATGCGGTCCCTGGGTCAAAACCCGTCCGAGTCCGAGCTGCAGGATATGATCAACGAGGTTGACGCCGACAACAACGGCACCATTGACTTCCCTGGTAAGCCCAGATGC-TCGCCTA-CCGGATATT

>Diaporthe_amygdali_CBS_126679

CTTCTCCCTCTTTGTAAGTTATTTTC---AGGA------ATGATC------CCGCAGCCCTCCTCGCCACTGTCGCGCATG--ATGCTAACGGACCGTTCTCGGCCTCCAGGATAAGGATGGCGATGGTTAGTGCAGCTGCCTCT----TCTTCAC-----CTCCCAGCTTCGTACGCGTCACGATCGACCCGCCGCGACGGCTTGCGCG-TGCA-CATTTTC-CAACCAAGC--ACCATAAC----------ATCTACTATGAGCTCGATGCTAAGAT--GAC-GTGTAGGACAAATCACCACAAAGGAGCTCGGCACGGTCATGCGATCTCTGGGTCAGAACCCGTCCGAGTCTGAGCTGCAGGATATGATCAACGAGGTCGACGCCGACAACAATGGAACCATCGACTTCCCTGGTACGTCCAGATGC-TCGCTTGTTTGGAAGGN

>Diaporthe_eres_CBS_138594

CTTTTCCCTCTTTGTAAGTCATTTCCAGCCGGCA--GACATGAGC------TCCCCGCCCT--CCTCTGCTGGTGCGCATG--ATGCTAACGGACCGTTTTCGGCTTGTAGGATAAGGATGGCGATGGTTAGTGCGGCCGCCTCT----TTGCCCC-----CTCCCACCTACGCACGCGTCATGTTCGATCCGCCGCGACAGCCTGCGCG-TGCA-TAATTTC-CAACCAAGCG-ATTATCAC----------ATCTATCACGAGTATCATGCTGAGATATGGC-GTGTAGGGCAAATCACCACCAAGGAGCTCGGCACGGTCATGCGATCCCTGGGTCAGAACCCGTCCGAGTCTGAGCTGCAAGATATGATTAACGAGGTCGACGCCGACAACAATGGCACCATTGACTTCCCTGGTACGTCCAGATGC-TCGCGCT-CTG?????N

>Diaporthe_batatas_CBS_122_21

CTTCTCCCTCTTTGTAAGTTATCTTC------A---GCCTTGAGC-----TTCCCCGCCGT--TCTCTTCCGTTGCGCACG--ATGCTAACCGACCGTTTTCGGCATGTAGGATAAGGATGGCGATGGTTAGTGCGGTCACCGCT----TCCTCCCCTCT-TTCTCAGCTACGCACGCGTCATACTCGATCCACCGCGACGGTCTGCGCG-TGCAGTATACTG-CGAGCGACCG-ATCACCGA----------ATCTATCACGAGAAATCTACTAAGACGGCCCGGTGTAGGACAAATCACCACCAAGGAGCTCGGCACAGTCATGCGGTCCCTTGGTCAGAACCCTTCCGAGTCCGAGCTGCAGGACATGATCAACGAGGTCGACGCCGACAACAATGGCACCATTGACTTCCCTGGTAAGTCTGGATTC-CCACACA-CTGGGTATN

>Diaporthe_convolvuli_CBS_124654

CTTCTCCCTCTTTGTAAGTTATCTCCAGGAGGAGGAGCCTTGAGC-----TTCCCCGCCAT--TCTCTACTGTTGCGCATG--ATGCTAACGGACCGTTTTCGACCTGCAGGATAAGGATGGCGATGGTTAGTGCGGTCACCGCT----TCCTCACCCCT-TTCTCAGCTACGCACGCGTCGTACTCGATCCGCCGCGACGGTCTGCGCG-TGCAGTCTACTC-CGAGCGACCG-ATCATCAA----------ATCTATCAC----AGTATGCTAAGGCT-GGC-GTGTAGGACAAATCACCACCAAGGAGCTCGGCACAGTCATGCGGTCGCTTGGTCAAAACCCTTCCGAGTCCGAGCTGCAGGACATGATCAACGAGGTCGACGCTGACAACAACGGCACCATTGACTTCCCTGGTAAGTCTCTCAAC-TGTCACA-CTGGAGATN

>Diaporthe_endophytica_CBS_133811

CTTCTCCCTCTTTGTGAGTTATCTCC---CGGA---GCCTTGAGC-----TTCCCCGCCGT--TCTCTGCCGTTGCGCATG--ATGCTAACGGACCGTTTTCGGCCTGCAGGATAAGGATGGCGATGGTTAGTGCGGTCACCGCT----TCCTCCCCTCT-TTCCCGGCTACGCACGCGTCATGCTCGATCCGCCGCGACGGTCTGCGCG-TGCAGTCTACTC-CGAGCGACCG-ATCATCAA----------ATCTATCACGGGTGGTATGCTAAGGCT-GGC-ATGTAGGACAAATCACCACCAAGGAGCTCGGCACAGTCATGCGGTCGCTTGGTCAAAACCCTTCCGAGTCCGAGCTGCAGGACATGATCAACGAGGTCGACGCCGACAACAACGGCACCATTGACTTCCCTGGTAAGTCTCAACTG-T--TACA-CTGGGGATN

>Diaporthe_fructicola_MAFF_246408

CTTCTCCCTCTTTGTGAGTTATCTCC---AGGA---GCCTTGAGC-----TTCCCCGCCGT--TCTCTGCCGTTGCGCATG--ATGCTAACGGACCGTTTTCGGCCTGCAGGATAAGGATGGCGATGGTTAGTGCGGTCACCGCT----TCCTCCCCTCT-TTGCCGGCTACGCACGCGTCATGCTCGATCCGCCGCGACGGTCTGCGCG-TGCAGTCTACTC-CGAGCGACCG-ATCATCAA----------ATCCATCACGAGTGGTATGCTAAGGCT-GGC-ATGTAGGACAAATCACCACCAAGGAGCTCGGCACAGTCATGCGGTCGCTTGGTCAAAACCCTTCCGAGTCCGAGCTGCAGGACATGATCAACGAGGTCGACGCCGACAACAACGGCACCATTGACTTCCCTGGTAAGTCTGAACTG-T--TACA-CTGGGGATN

>Diaporthe_phaseolorum_CBS_113425

CTTCTCCCTCTTTGTGAGTTATCTCC---AGGA---GCCTTGAGC-----TCCCCCGCCGT--TCTCTGCCGTTGCGCATG--ATGCTAACGGACCGTTTTCGGCCTGCAGGATAAGGATGGCGATGGTTAGTGCGGTCACCGCT----TCCTCCCCTCT-TTCCCGGCTACGCACGCGTCATGCTCGATCCGCCGCGACGGTCTGCGCC-TGCAGTCTACTC-CGAGCGACCG-ATCATCAA----------ATCTATCACGAGTGGTATGCTAAGGCT-GGC-ATGTAGGACAAATCACCACCAAGGAGCTCGGCACAGTCATGCGGTCGCTTGGTCAAAACCCTTCCGAGTCCGAGCTGCAGGACATGATCAACGAGGTCGACGCCGACAACAATGGCACCATTGACTTCCCTGGTAAGTCTCAACTG-T--CACA-CCGGGGATN

>Diaporthe_sojae_CBS_139282

CTTCTCCCTCTTTGTGAGTTATCTCC---AGGA---GCCTTGAGC-----TCCCCCGCCGT--TCTCTGCCGTTGCGCATG--ATGCTAACGGACCGTTTTCGGCCTGCAGGATAAGGATGGCGATGGTTAGTGCGGTCACCGCT----TCCTCCCCTCT-TTCCCGGCTACGCACGCGTCATGCTCGATCCGCCGCGACGGTCTGCGCC-TGCAGTCTACTC-CGAGCGACCG-ATCATCAA----------ATCTATCACGAGTGGTATGCTAAGGCT-GGC-ATGTAGGACAAATCACCACCAAGGAGCTCGGCACAGTCATGCGGTCGCTTGGTCAAAACCCTTCCGAGTCCGAGCTGCAGGACATGATCAACGAGGTCGACGCCGACAACAATGGCACCATTGACTTCCCTGGTAAGTCTCAACTG-T--CACA-CCGGGGATN

>Diaporthe_heterostemmatis_SAUCC194_85

?????????????????GGCAATCTC---AGGA---GCCTTGAGC-----TT-CCCGCCGT--TCTCTGCCGTTGCGCATG--ATGCTAACGGACCGTTTTCGGCCTGCAGGATAAGGATGGCGATGGTTAGTGCGGTCACCGCT----TCCTCCCCTCT-TTCCCGGCTACGCACGCGTCATGCTCGATCCGCCGCGACGGTCTGCGCG-TGCAGTCTACTC-CGAGCGACCG-ATCATCAA----------ATCTATCACGAGTGGTATGCTAAGGCT-GGC-ATGTAGGACAAATCACCACCAAGGAGCTCGGCACAGTCATGCGGTCGCTTGGTCAAAACCCTTCCGAGTCCGAGCTGCAGGACATGATCAACGAGGTCGACGCCGACAACAACGGCACCATTGACTTCCCTGGTAAGTCTCAACTG-T--CACA-CTGGAGATN

>Diaporthe_guangdongensis_ZHKUCC20_0014

CTTCTCCCTCTTTGTAAGTCATCTTC---AGGA---GCCTTGAGC-----TTCCCCGCCGT--TCTCTGCCGTTGCGCATG--ATGCTAACGGACCGTTTTCGGCCTGCAGGATAAGGATGGCGATGGTTAGTGCGGTCACCGCT----TCCTCCCCTCT-TCCTCAGCTACGCACGCGTCACACTCGATCCGCCGCGACGGTCTGCGCG-TGCAGTATATTC-CGAGCGACCG-ATCATCAC----------ATCCATCACGAGTAGTATGCTAAGGCG-GGC-GTGTAGGACAAATCACCACCAAGGAGCTCGGCACAGTCATGCGGTCCCTTGGTCAAAACCCTTCCGAGTCCGAGCTGCAGGACATGATCAACGAGGTCGACGCCGACAACAATGGCACCATTGACTTCCCTGGTGAGTCTA-ATTC-TGGCACA-CTGGATATN

>Diaporthe_melonis_CBS_507_78

CTTCTCCCTCTTTGTAAGTTGTCTTC---AGGA---GCCTTGAGC-----TTCCCCGCCGT--TCTCTGCCGTTGCGCATG--ATGCTAACGGACCGTTTTCGGCCTGCAGGATAAGGATGGCGATGGTTAGTGCGGTCACCGCT----TCCTCCCCTCT-TTCTCAGCTACGCACGCGTCA-ACTCGATCCGCCGCGACGGTCTGCGCG-TGCAGTATATTC-CGAGCGACTG-ATGATCAC----------ATCCATCACGAGTGGTATGCTAAGGCG-GGC-GTGTAGGACAAATCACCACCAAGGAGCTCGGCACAGTCATGCGGTCCCTTGGTCAAAACCCTTCCGAGTCCGAGCTGCAGGACATGATCAACGAGGTCGACGCCGACAACAATGGCACCATTGACTTCCCTGGTGAGTCTAGATTC-TCGCACA-GTGGATATN

>Diaporthe_infertilis_CBS_230_52

CTTCTCCCTCTTTGTAAGTTATCTTC---AGGA---GCCTTGAGC-----TTCCCCGCCGT--TCTTTGCCGTTGCGCATG--ATGCTAACTGACCGTTTTCGGCCTACAGGATAAGGATGGCGATGGTTAGTGCGGTCACCGCT----TCCTCCCCTCT-TTCTCAGCTACGCACGCGTCATACTCGATCCGCCGCGACGGTCTGCGCG-TGCAGTATATTC-CGAGCGACCG-TTCATCAA----------ATCTATCACGAGTAGTATGCTAAGGCG-GGC-GTGCAGGACAAATCACCACCAAGGAGCTCGGCACAGTCATGCGGTCCCTGGGCCAGAACCCTTCCGAGTCCGAGCTGCAGGACATGATCAACGAGGTCGACGCCGACAACAACGGCACCATTGACTTCCCTGGTGAGTCCAGATTC-TCGCACA-TTGGTTTTN

>Diaporthe_megalospora_CBS_143_27

CTTCTCCCTCTTTGTAAGTTATGTCC---AGGA---GCCTTGAGC-----TTCCCCGCCGT--CCCATGCCGTTGCGCATG--ATGTTAACGGACCGTTTTCGGCCTGCAGGATAAGGATGGCGATGGTTAGTGCGGTCACCGCT----TGATTCCCTCT-TTCTCAGCTACGCACGCGTCATATTCGATCCGCCGCGACGGTCTGCGCG-TGCAGTATACTC-CGAGCGACCG-ATCATCAA----------ATATATCACGAGTAGTATGCTAAGGCG-GGC-GTGTAGGACAAATCACCACCAAGGAGCTCGGCACAGTCATGCGGTCCCTTGGTCAAAACCCTTCCGAGTCCGAGCTGCAAGATATGATCAACGAGGTTGACGCCGACAACAATGGCACCATTGACTTCCCTGGTAAGTCGAGATTC-CTGCACA-CTGGATATN

>Diaporthe_schini_CBS_133181

CTTCTCCCTCTTTGTAAGTTATCTTT---AGGA---GCCTTGAGC-----TCCCCCGCCGT--TCTATGCTGTCGCGCATG--ATGCTAACGGACCGTTTTCGGCCTGCAGGATAAGGATGGCGATGGTTAGTGCGGTCACCGCT----TCCTTCCCTCT-TTCTCAGCTACGCACGCGTCATACTCGATCCGCCGCGACGGTCTGCGCG-TGCAGTATACTC-CGAGCGACCG-ATCATCAA----------ATCTATCACGAGTAGTATGCTAAGGCG-GGC-GTGTAGGACAAATCACCACCAAGGAGCTCGGCACAGTCATGCGGTCCCTTGGTCAAAACCCCTCCGAGTCCGAGCTGCAGGACATGATCAACGAGGTCGACGCCGACAACAATGGCACCATTGACTTCCCTGGTAAGTCGAGATTC-TCGCACA-CTGGATATN

>Diaporthe_tecomae_CBS_100547

CTTCTCCCTCTTTGTAAGTTATCTTT---AGGA---GCCTTGAGC-----TTCCCCGCCGT--TCTCTGCTGTTGCGCGTG--ATGCTAATGGACCGTTTTCGGCCTGCAGGATAAGGATGGCGATGGTTAGTGCGGTCACCGCT----TCCTTCCCTCT-TTCTCAGCTACGCACGCGTCATACTCGATCCACCGCGACGGTCTGCGCG-TGCAGTATACCC-CGAGCGACCG-ATCATCAA----------ATCTATCACGAGTAGTATGCTAAGGCG-GGC-GTGTAGGACAAATCACCACCAAGGAGCTCGGCACAGTCATGCGGTCCCTTGGTCAAAACCCTTCCGAGTCCGAGCTGCAGGACATGATCAACGAGGTCGACGCCGACAACAATGGCACCATTGACTTCCCTGGTAAGCCTAGATTC-TCGCATA-CTGGATATN

>Diaporthe_terebinthifolii_CBS_133180

CTTCTCCCTCTTTGTAAGTTATATTT---AGGA---GCCTTGAGC-----TTCCCCGCCGT--TCTCTGCTGTTGCGCGTA--ATGCTAACGGACCGTTTTCGGCCTGCAGGATAAGGATGGCGATGGTTAGTGCGGTCACCGCT----TCCTTCCCTCT-TTCTCAGTTATGCACGCGTCATACTCGATCCGCCGCGACGGTCTGCGCG-TGCAGTATACCC-CGAGCGACCG-ATCATCAA----------ATCTATCACGAGTAGTATGCTAAGGCA-GGC-GTGTAGGACAAATCACCACCAAGGAGCTCGGCACAGTCATGCGGTCCCTTGGTCAAAACCCTTCCGAGTCCGAGCTGCAGGACATGATCAACGAGGTCGACGCCGACAACAATGGCACCATTGACTTCCCTGGTAAGTCTAGATTC-TCGCACA-CTGGATATN

>Diaporthe_tectonendophytica_MFLUCC_13_0471

?????????????????????????T---AGGT---GCCTTGAGC-----TTCCCCGCCGT--TCTCTGCTGTTGCGCATG--ATGCTAACTGACCGTTTTCGGCCTACAGGATAAGGATGGCGATGGTTAGTGCGGTCACCGCT----TCCTCCCCTCT-TTCTCAGCTACGCACGCGTCATACTCGATCCGCCGCGACGGTCTGCGCG-TGCAGTATATTC-CGAGCGACCG-ATCATCAA----------ATCTATCACGAGTAGTATGCTAAGGTG-GGC-GTGCAGGACAAATCACCACCAAGGAGCTCGGCACAGTTATGCGGTCCCTTGGTCAAAACCCTTCCGAGTCCGAGCTGCAGGACATGATCAACGAGGTCGACGCCGACAACAACGGCACCATTGACTTCCCTGGTAAGTCTAGATTC-TCGTACA-ATGGGTATN

>Diaporthe_racemosae_CBS_143770

???????????????????????????????????????????????????????????GT--TCTCTGCTGTCGCGCATG--ATGCTAACGGACTGTTTTCGGCCTGCAGGATAAGGATGGCGATGGTTAGTGCGGTCACCGCT----TCCTCCCCTCT-TTCTCAGCTACGCACGCGTCATACTCGGTCCGCCGCGACGATCTGCGCG-TGCAGTACACTC-CGAGCGACCG-ATCGTCAA----------ATCTATCACGAGTAGTATGCTAAGGCG-GGC-GTGTAGGACAAATCACCACCAAGGAGCTCGGCACAGTCATGCGGTCCCTTGGTCAAAACCCTTCCGAGTCCGAGCTGCAGGACATGATCAACGAGGTCGACGCCGACAACAACGGCACTATTGACTTCCCTGGTAAGTCTAGATTC-TCGCACA-CTGGATATN

>Diaporthe_rosiphthora_COAD_2913

???CTCCCTCTTTGTAAGTTATCTTC---AGAA---GCTTTGAGC-----TTCCCCGCGGT--TCTCTGCTGTTGCGCACG--ATGCTAACGGACCGTTGTCGGCCTGCAGGATAAGGATGGCGATGGTTAGTGCGGTCACCGCT----TCCTTCCCTCT-TTCTCAGCTACGCACGCGTCACACTCGATCCGCCGCGACGGTCTGCGCG-TGCAGAATACTC-CGAGCGACCG-ATCATCAA----------ATCTATCACGAGGAAAATGCTAAGACC-GGC-GTGTAGGACAAATCACCACCAAGGAGCTCGGCACAGTCATGCGGTCCCTTGGTCAAAACCCTTCCGAGTCCGAGCTGCAGGACATGATCAACGAGGTCGACGCCGACAACAATGGCACCATTGACTTCCCTGGTAAGTCTAGATTC-TCGCACA-CTGGATATN

>Diaporthe_longicolla_FAU_599

CTTCTCCCTCTTTGTAAGTTATATCC---AGGA---GCCTCGAGC-----TTCCCCGCCGT--TCTCTGCTGTTGCGCCTG--ATGCTAACGGACCGTTTTCGGCCTGCAGGATAAGGATGGCGATGGTTAGTGCGGTCACCGCT----TCCTCCCCTCT-TTCTCAGCTACGCACGCGTCATACTCGATCCGCCGCGACGGTCTGCGCG-TGCAG-CTACTC-CGACCGACCG-ACCATCAA----------ATCTATCACGAGTAGTATGCTAAGGCT-GGC-GTGTAGGACAAATCACCACCAAGGAGCTCGGCACAGTCATGCGGTCCCTTGGTCAAAACCCTTCCGAGTCCGAGCTGCAGGACATGATCAACGAGGTCGACGCCGACAACAACGGCACCATTGACTTCCCTGGTGAGTCTAGATCC-TCGTACA-CTGGATATN

>Diaporthe_rosae_MFLUCC_17_2658

CTTCTCCCTCTTTGTAAGTTATATCC---AGGA---GCCTTGAGC-----TTCCCCGCCGT--TCTCTGCTGTTGCGCCTG--ATGCTAACGGACCGTTTTCGGCCTACAGGATAAGGATGGCGATGGTTAGTGCGGTCACCGCT----TCCTCCCCTCT-TTCTCGGCTACACACGCGTCATACTCGATCCGCCGCGACGGTCTGCGCG-TGCAGTCCACTC-CGCGCGACCG-ACCACCAA----------ATCTGTCACGAGTAGTATGCTAAGGCT-GGG-ATGTAGGACAAATCACCACCAAGGAGCTCGGCACAGTCATGCGGTCCCTTGGTCAGAACCCTTCCGAGTCCGAGCTGCAGGACATGATCAACGAGGTCGACGCCGACAACAACGGCACCATTGACTTCCCTGGTAAGTCTAGATTC-TCGTACA-CTGGATATN

>Diaporthe_ueckerae_FAU_656

CTTCTCCCTCTTTGTAAGTTATATCC---AGGA---GCCTTGAGC-----TTCCCCGCCGT--TCTCTGCTGTTGCGCCTG--ATGCTAACGGACCGTTTTCGGCCTACAGGATAAGGATGGCGATGGTTAGTGCGGTCACCGCT----TCCTCCCCTCT-TTCTCGGCTACACACGCGTCATACTCGATCCGCCGCGACGGTCTGCGCG-TGCAGTCTACTC-CGAGCGACCG-ACCATCAA----------ATCTATCACGAGTAGTATGCTAAGGCT-GGG-GTGTAGGACAAATCACCACCAAGGAGCTCGGCACAGTCATGCGGTCCCTTGGTCAGAACCCTTCCGAGTCCGAGCTGCAGGACATGATCAACGAGGTCGACGCCGACAACAACGGCACCATTGACTTCCCTGGTGAGTCTAGATTC-TCGTACA-CTGGATATN

>Diaporthe_vochysiae_LGMF1583

CTTCTCCCTCTTTGTAAGTTATATCC---AGGA---GCCTTGAGC------TCCCCGCCGT--TCTCTGCTGTTGCGCCTG--ATGCTAACGGACCGTTTTCGGCCTACAGGATAAGGATGGCGATGGTTAGTGCGGTCACCGCT----TCCTCCCCTCT-TTCTCAGCTACACACGCGTCATACTCGATCCGCCGCGACGGTCTGCGCG-TGCAGTCTACTC-CGAGCGACCG-ACCATCAA----------ATCTATCACGAGTAGTATGCTAAGGCT-GGG-GTGTAGGACAAATCACCACCAAGGAGCTCGGCACAGTCATGCGGTCCCTTGGTCAGAACCCTTCCGAGTCCGAGCTGCAGGACATGATCAACGAGGTCGACGCCGACAACAACGGCACCATTGACTTCCCTGGTAAGTCTAGATTC-TCGTACA-CTGGATATN

>Diaporthe_breyniae

CTTCTCCCTCTTTGTAAGTTATATCC---AGGA---GCCTTGAGC-----TTCCCCGCCGT--TCTCTGCTGTTGCGCCTG--ATGCTAACGGACCGTTTTCGGCCTACAGGATAAGGATGGCGATGGTTAGTGCGGTCACCGCT----TCCTCCCCTCT-TTCTCAGCTACGCACGCGTCATACTCGATCCGCCGCGACGGTCTGCGCG-TGCAGTCTACTC-CGAGCGACCG-ACCATGAA----------ATCTATCACGAGTAGTATGCTAAGGCT-GGC-GTGTAGGACAAATCACCACCAAGGAGCTCGGCACAGTCATGCGGTCCCTTGGCCAGAACCCTTCCGAGTCCGAGCTGCAGGATATGATCAACGAGGTCGACGCCGACAACAACGGCACCATTGACTTCCCTGGTAAGACTATCTTC-GCGTACA-CTGGATACN

>Diaporthe_hordei_CBS_481_92

CTTCTCCCTCTTTGTAAGTTATCTTC---AAGA---GCCTTAAGC-----TTCCCCGCCGTTCTCTCTGCCGTTGCGCATG--GTGCTAACGGACCGTTTTCGGCCTGCAGGATAAGGATGGTGATGGTTAGTGCGGTCACCGCTTCCCTCCCTCCCTCT-TTCTCAGCTTTGAACGCGTCATACTCGATCCGCCGCGACGGTCTGCGCT-TGCAATATACTC-CGAGCGACCA-ATCTTCAA----------ATCTATCACGAGTAGTATGCTAAGGCG-GGC-GTGTAGGACAAATCACCACCAAGGAGCTCGGCACAGTCATGCGGTCCCTTGGTCAAAACCCTTCCGAGTCCGAGCTGCAGGACATGATCAACGAGGTCGACGCCGACAACAATGGCACCATTGACTTCCCTGGTAAGTCTAGATTC-TCTCACA-CTGAATATN

>Diaporthe_vexans_CBS_127_14

CTTCTCCCTCTTTGTAAGTTTTGTTC---AGGAGGGGCCTTGAGC-----TTCCCCGCCGT--TCTCTGCCGTTGCGCGTC--ATGCTAACGAACCGTTTTCGGCCTGCAGGATAAGGATGGCGATGGTTAGTGCGGTCACCGCT----TCCCTCCCTCT-CTCTCAGCTACGCACGCATCACACTCGATCCGCCGCGACGGTCTGCGCT-CGCCATATGTTC-CGAGCGACCG-ATCATCAA----------ATATACAACGACTAGTGTGCTAAGGCG-GAC-GTGTAGGACAAATCACCACCAAGGAGCTCGGCACAGTCATGCGGTCCCTTGGTCAAAACCCTTCCGAGTCCGAGCTGCAGGACATGATCAACGAGGTCGACGCCGACAACAATGGCACCATTGACTTCCCTGGTAAGTCTAGATCC-TCGCACA-CTGGATATN

>Diaporthe_helianthi_CBS_592_81

????????????????????????????????????????????????????????GCCGT--TCTCTGCTGTTGCGCCTGGTGTGCTAATGGACCGTTTTCTGCCTGCAGGATAAGGACGGCGATGGTTAGTGCGGTCACCGCT----TCCCGCCCTCT-CTCTCAGCTGCGCACGCGTCACAATCGATCCGCCGCAACGGTCTGCGCT-TGCAATGTACCC-CGAGCGACCG-ATCATTAAATCTATCACGATCTATCACGAGTCGTATGCTAAGGCG-GGC-GTGTAGGACAAATCACCACCAAGGAGCTGGGCACAGTCATGCGGTCCCTTGGTCAAAACCCTTCCGAGTCCGAGCTGCAGGACATGATCAACGAGGTCGACGCCGACAACAATGGCACCATTGACTTCCCTGGTACGTTTAGATTC-TCGTACA-CTGCATATN

# Alignment of the *his3* sequences used in the second phylogenetic study

>Diaporthe_breyniae

TCCGCGCCCTCCACCGGAGGTGTCAAGAAGCCTCACCGCTACAAGCCTGGTACCGTCGCTCTGCGTGAGATCCGTCGCTACCAGAAGAGCACCGAGCTGCTGATCCGCAAGCTCCCCTTCCAGCGTCTGGTATGTTTTGCACCTCACCAA------------TCACCCTCATCCTCGTACACCCTGCTGACTGTCG--CGCCTCCCTCTCCAGGTCCGTGAGATCGCCCAGGACTTCAAGTCCGACCTGCGCTTCCAGTCTTCCGCCATCGGTGCTCTCCAGGAGTCCGTCGAGTCTTACCTCGTCTCCCTCTTCGAGGACACCAACCTGTGCGCCATTCACGCCAAGCGTGTCACCATCCAGTCGGTACGTC

>Diaporthe_acaciarum_CBS_138862

TCCGCGCCCTCCACCGGAGGTGTCAAGAAGCCTCACCGCTACAAGCCTGGTACCGTCGCTCTGCGTGAGATCCGTCGCTACCAGAAGAGCACCGAGCTGCTGATCCGCAAGCTGCCCTTCCAGCGTCTGGTATG----------------------------------------------------------------------------CAGGTCCGTGAGATCGCCCAGGACTTCAAGTCCGACCTGCGTTTCCAGTCTTCCGCCATCGGTGCCCTTCAGGAGTCCGTCGAGTCTTACCTCGTCTCCCTCTTTGAGGACACCAACCTGTGCGCCATCCACGCNNNNNNNNNNNNNNNNNNNNNNNNNNNNN

>Diaporthe_infecunda_CBS_133812

TCCGCGCCCTCCACCGGAGGTGTCAAGAAGCCTCACCGCTACAAGCCTGGTACCGTCGCTCTGCGTGAGATCCGTCGCTACCAGAAGAGCACCGAGCTGCTGATCCGCAAGCTGCCCTTCCAGCGTCTGGTATG----------------------------------------------------------------------------TAGGTCCGTGAGATCGCCCAGGACTTCAAGTCCGACCTGCGCTTCCAGTCATCCGCCATCGGTGCCCTTCAGGAGTCCGTCGAGTCTTACCTCGTCTCCCTCTTTGAGGACACCAACCTGTGCGCCATCCACGCCAAGCGTGTCACCATCCAGTCGGTACGTC

>Diaporthe_leucospermi_CBS_111980

TCCGCGCCCTCCACCGGAGGTGTCAAGAAGCCTCACCGCTACAAGCCTGGTACCGTCGCTCTGCGTGAGATCCGTCGCTACCAGAAGAGCACCGAGCTGCTGATCCGCAAGCTGCCCTTCCAGCGTCTGGTATG----------------------------------------------------------------------------TAGGTCCGTGAGATCGCCCAGGACTTCAAGTCCGACCTGCGCTTCCAGTCTTCCGCCATCGGTGCCCTTCAGGAGTCCGTCGAGTCTTACCTCGTCTCCCTCTTTGAGGACACCAACCTGTGCGCCATCCACGCCAAGCGTGTCACCATCCAGTCGGTACGTC

>Diaporthe_biguttulata_ICMP20657

TCCGCGCCCTCCACCGGAGGTGTCAAGAAGCCTCACCGCTACAAGCCTGGTACCGTCGCTCTGCGTGAGATCCGTCGCTACCAGAAGAGCACCGAGCTGCTGATCCGCAAGCTCCCCTTCCAGCGTCTGGTATG----------------------------------------------------------------------------CAGGTTCGTGAGATCGCCCAGGACTTCAAGTCCGACCTGCGCTTCCAGTCTTCCGCCATCGGTGCCCTGCAGGAGTCCGTCGAGTCCTACCTCGTCTCCCTCTTTGAGGACACCAACCTGTGCGCCATCCACGCCAAGCGTGTCACCATCCAGTCGGTACGTC

>Diaporthe_yunnanensis_CGMCC_3_18289

TCCGCGCCCTCCACCGGAGGTGTCAAGAAGCCTCACCGCTACAAGCCTGGTACCGTCGCTCTGCGTGAGATCCGTCGTTACCAGAAGAGCACCGAGCTGCTGATCCGCAAGCTCCCCTTCCAGCGTCTGGTATG----------------------------------------------------------------------------CAGGTTCGTGAGATCGCCCAGGACTTCAAGTCCGACCTGCGCTTCCAGTCTTCCGCCATCGGTGCCCTGCAGGAGTCCGTCGAGTCTTACCTCGTCTCCCTCTTTGAGGACACCAACCTGTGCGCCATCCACGCCAAGCGTGTCACCATCCAGTCGGTACGTC

>Diaporthe_discoidispora_ICMP20662

TCCGCGCCCTCCACCGGAGGTGTCAAGAAGCCTCACCGCTACAAGCCTGGCACCGTCGCTCTGCGTGAGATCCGTCGCTACCAGAAGAGCACCGAGCTGCTGATCCGCAAGCTCCCCTTCCAGCGTCTGGTATG----------------------------------------------------------------------------CAGGTTCGTGAGATCGCCCAGGACTTCAAGTCCGACCTGCGCTTCCAGTCTTCCGCCATCGGTGCCCTTCAGGAGTCCGTCGAGTCCTACCTCGTCTCCCTCTTTGAGGACACCAACCTGTGCGCCATCCACGCCAAGCGTGTCACCATCCAGTCGGTACGTT

>Diaporthe_compacta_LC3083

TCCGCGCCCTCCACCGGAGGTGTCAAGAAGCCTCACCGCTACAAGCCTGGTACCGTCGCTCTGCGTGAGATCCGTCGCTACCAGAAGAGCACCGAGCTGCTCATCCGCAAGCTCCCCTTCCAGCGTCTGGTATG----------------------------------------------------------------------------CAGGTCCGTGAGATCGCCCAGGACTTCAAGTCCGACCTGCGCTTCCAGTCTTCCGCCATCGGTGCCCTTCAGGAGTCCGTCGAGTCTTACCTCGTCTCCCTCTTTGAGGACACCAACCTGTGCGCCATCCACGCCAAGCGTGTCACCATCCAGTCGGTACGTC

>Diaporthe_ganjae_CBS_180_91

TCCGCGCCCTCCACCGGAGGTGTCAAGAAGCCTCACCGCTACAAGCCTGGTACCGTCGCTCTGCGTGAGATCCGTCGCTACCAGAAGAGCACCGAGCTGCTCATCCGCAAGCTCCCCTTCCAGCGTCTGGTATG----------------------------------------------------------------------------CAGGTTCGTGAGATCGCCCAGGACTTCAAGTCCGACCTGCGCTTCCAGTCTTCCGCCATCGGTGCCCTTCAGGAGTCCGTCGAGTCTTACCTCGTCTCCCTCTTTGAGGACACCAACCTGTGCGCCATCCACGCCAAGCGTGTCACCATCCAGTCGGTACGTC

>Diaporthe_manihotia_CBS_505_76

TCCGCGCCCTCCACCGGAGGTGTCAAGAAGCCTCACCGCTACAAGCCTGGTACCGTCGCTCTGCGTGAGATCCGTCGCTACCAGAAGAGCACCGAGCTGCTCATCCGCAAGCTCCCCTTCCAGCGTCTGGTATG----------------------------------------------------------------------------CAGGTTCGTGAGATCGCCCAGGACTTCAAGTCCGACCTGCGCTTCCAGTCTTCCGCCATCGGTGCTCTCCAGGAGTCCGTCGAGTCTTACCTCGTCTCCCTCTTTGAGGACACCAACCTGTGCGCCATCCACGCCAAGCGTGTCACCATCCAGTCGGTACGTC

>Diaporthe_angelicae_CBS_111592

TCCGCGCCCTCCACCGGAGGTGTCAAGAAGCCTCACCGCTACAAGCCTGGTACCGTCGCTCTGCGTGAGATCCGTCGCTACCAGAAGAGCACCGAGCTGCTCATCCGCAAGCTCCCCTTCCAGCGTCTGGTAAG----------------------------------------------------------------------------CAGGTTCGTGAGATCGCCCAGGACTTCAAGTCCGACCTGCGCTTCCAGTCTTCCGCCATCGGCGCCCTCCAGGAGTCTGTCGAGTCTTACCTCGTCTCCCTCTTCGAGGACACCAACCTGTGCGCCATCCACGCCAAGCGTGTCACCATCCAGTCGGTACGTC

>Diaporthe_novem_CBS_127271

TCCGCGCCCTCCACCGGAGGTGTCAAGAAGCCTCACCGCTACAAGCCTGGTACCGTCGCTCTGCGTGAGATCCGTCGCTACCAGAAGAGCACCGAGCTGCTCATCCGCAAGCTCCCCTTCCAGCGTCTGGTAAG----------------------------------------------------------------------------CAGGTTCGTGAGATCGCCCAGGACTTCAAGTCCGACCTGCGCTTCCAGTCTTCTGCCATCGGCGCCCTTCAGGAGTCCGTCGAGTCTTACCTCGTCTCCCTCTTCGAGGACACCAACCTGTGCGCCATCCACGCCAAGCGTGTCACCATCCAGTCGGTACGTC

>Diaporthe_arctii_CBS_136_25

TCCGCGCCCTCCACCGGAGGTGTCAAGAAGCCTCACCGCTACAAGCCTGGTACCGTCGCTCTGCGTGAGATCCGTCGCTACCAGAAGAGCACTGAGCTGCTCATCCGCAAGCTGCCCTTCCAGCGTCTGGTAAG----------------------------------------------------------------------------CAGGTTCGTGAGATCGCCCAGGACTTCAAGTCCGACCTGCGCTTCCAGTCTTCCGCCATCGGCGCCCTCCAGGAGTCCGTCGAGTCCTACCTCGTCTCCCTCTTCGAGGACACCAACCTGTGCGCCATCCACGCCAAGCGTGTCACCATCCAGTCGGTACGTC

>Diaporthe_cucurbitae_DAOM_42078

TCCGCGCCCTCCACCGGAGGTGTCAAGAAGCCTCACCGCTACAAGCCTGGTACCGTCGCTCTGCGTGAGATCCGTCGCTACCAGAAGAGCACCGAGCTGCTCATCCGCAAGCTGCCCTTCCAGCGTCTGGTAAG----------------------------------------------------------------------------CAGGTTCGTGAGATCGCCCAGGACTTCAAGTCCGACCTGCGCTTCCAGTCTTCCGCCATCGGCGCCCTCCAGGAGTCCGTCGAGTCCTACCTCGTCTCCCTCTTCGAGGACACCAACCTGTGCGCCATCCACGCCAAGCGTGTCACCATCCAGTCGGTACGTC

>Diaporthe_subordinaria_CBS_101711

TCCGCGCCCTCCACCGGAGGTGTCAAGAAGCCTCACCGCTACAAGCCTGGTACCGTCGCTCTGCGTGAGATCCGTCGCTACCAGAAGAGCACCGAGCTGCTCATCCGCAAGCTGCCCTTCCAGCGTCTGGTAAG----------------------------------------------------------------------------CAGGTTCGTGAGATCGCCCAGGACTTCAAGTCCGACCTGCGCTTCCAGTCTTCCGCCATCGGCGCCCTCCAGGAGTCCGTCGAGTCCTACCTCGTCTCCCTCTTCGAGGACACCAACCTGTGCGCCATCCACGCGAAGCGTGTCACCATCCAGTCGGTACGTC

>Diaporthe_cuppatea_CBS_117499

TCCGCGCCCTCCACCGGAGGTGTCAAGAAGCCTCACCGCTACAAGCCTGGTACCGTCGCTCTGCGTGAGATCCGTCGCTACCAGAAGAGCACCGAGCTGCTGATCCGCAAGCTCCCCTTCCAGCGTCTGGTAAG----------------------------------------------------------------------------CAGGTTCGTGAGATCGCCCAGGACTTCAAGTCCGACCTGCGCTTCCAGTCTTCTGCCATCGGTGCCCTTCAGGAGTCCGTCGAGTCTTACCTCGTCTCCCTCTTTGAGGACACCAACCTGTGCGCCATCCACGCCAAGCGTGTCACCATCCAGTCGGTACGTC

>Diaporthe_lusitanicae_CBS_123212

TCCGCGCCCTCCACCGGAGGTGTCAAGAAGCCTCACCGCTACAAGCCTGGTACCGTCGCTCTGCGTGAGATCCGTCGCTACCAGAAGAGCACCGAGCTGCTGATCCGCAAGCTCCCCTTCCAGCGTCTGGTAAG----------------------------------------------------------------------------CAGGTTCGTGAGATCGCCCAGGACTTCAAGTCCGACCTGCGCTTCCAGTCTTCTGCCATTGGTGCCCTTCAGGAGTCCGTCGAGTCTTACCTCGTCTCCCTTTTCGAGGACACCAACCTGTGCGCCATCCACGCCAAGCGTGTCACCATCCAGTCGGTACGTC

>Diaporthe_neoarctii_CBS_109490

TCCGCGCCCTCCACCGGAGGTGTCAAGAAGCCTCACCGCTACAAGCCTGGTACCGTCGCTCTGCGTGAGATCCGTCGCTACCAGAAGAGCACCGAGCTGCTGATCCGCAAGCTCCCCTTCCAGCGTCTGGTAAG----------------------------------------------------------------------------CAGGTTCGTGAGATCGCCCAGGACTTCAAGTCCGACCTGCGCTTCCAGTCTTCCGCCATCGGCGCTCTTCAGGAGTCTGTCGAGTCTTACCTCGTCTCCCTCTTTGAGGACACCAACCTGTGCGCCATCCACGCCAAGCGTGTCACCATCCAGTCGGTACGTC

>Diaporthe_ambigua_CBS_114015

TCCGCGCCGTCCACCGGAGGTGTCAAGAAGCCTCACCGCTACAAGCCTGGTACCGTCGCTCTGCGTGAGATCCGTCGCTACCAGAAGAGCACCGAGCTGCTGATCCGCAAGCTCCCCTTCCAGCGTCTGGTACG----------------------------------------------------------------------------AAGGTTCGTGAGATCGCCCAGGACTTCAAGTCCGACCTGCGCTTCCAGTCTTCCGCCATCGGTGCCCTGCAGGAGTCCGTCGAGTCTTACCTCGTCTCCCTCTTCGAGGACACCAACCTGTGCGCCATCCACGCCAAGCGTGTCACCATCCAGTCGGTACGTC

>Diaporthe_longispora_CBS_194_36

TCCGCGCCCTCCACCGGAGGTGTCAAGAAGCCTCACCGCTACAAGCCTGGTACCGTCGCTCTGCGTGAGATCCGTCGCTACCAGAAGAGCACTGAGCTGCTGATCCGCAAGCTCCCCTTCCAGCGTCTGGTATG----------------------------------------------------------------------------CAGGTCCGTGAGATCGCCCAGGACTTCAAGTCCGACCTGCGCTTCCAGTCTTCTGCCATCGGTGCCCTGCAGGAGTCTGTCGAGTCTTACCTCGTCTCTCTCTTCGAGGACACCAACCTGTGCGCCATCCATGCCAAGCGTGTCACCATCCAGTCGGTACGTC

>Diaporthe_sclerotioides_CBS_296_67

TCCGCGCCTTCCACCGGAGGTGTCAAGAAGCCTCACCGCTACAAGCCCGGTACCGTCGCTCTGCGTGAGATCCGTCGCTACCAGAAGAGTACCGAGCTGCTGATCCGCAAGCTCCCCTTCCAGCGTCTTGTATG----------------------------------------------------------------------------CAGGTTCGTGAGATCGCCCAGGACTTCAAGTCCGACCTGCGCTTCCAGTCTTCCGCCATCGGTGCCCTGCAGGAGTCTGTCGAGTCTTACCTCGTCTCTCTCTTCGAGGACACCAACCTGTGCGCCATCCACGCCAAGCGTGTCACCATCCAGTCGGTACGTT

>Diaporthe_mayteni_CBS_133185

TCCGCACCCTCCACCGGAGGTGTCAAGAAGCCCCACCGCTACAAGCCTGGTACCGTCGCTCTGCGTGAGATCCGTCGCTACCAGAAGAGCACTGAGCTGCTGATCCGCAAGCTCCCCTTCCAGCGTCTGGTAAG----------------------------------------------------------------------------CAGGTTCGTGAGATTGCCCAGGACTTCAAGTCCGACCTGCGCTTCCAGTCCTCCGCCATCGGTGCCCTGCAGGAGTCCGTCGAGTCTTACCTCGTCTCCCTCTTCGAGGACACCAACCTGTGCGCCATCCACGCCAAGCGTGTCACCATCCAGTCGGTACGTT

>Diaporthe_pyracanthae_CBS142384

TCCGCGCCCTCCACCGGAGGTGTCAAGAAGCCTCACCGCTACAAGCCTGGTACCGTCGCTCTGCGTGAGATCCGTCGTTACCAGAAGAGCACTGAGCTGCTGATCCGCAAGCTCCCCTTCCAGCGTCTGGTATG----------------------------------------------------------------------------CAGGTTCGTGAGATCGCCCAGGACTTCAAGTCCGACCTCCGCTTCCAGTCCTCCGCCATCGGTGCCCTGCAGGAGTCCGTCGAGTCTTACCTCGTCTCCCTGTTCGAGGACACCAACTTGTGCGCCATCCACGCCAAGCGTGTCACCATCCAGTCGGTACGTT

>Diaporthe_citriasiana_CBS_134240

TCCGCGCCCTCCACCGGAGGTGTCAAGGAGCCTCACCGGCACAAGCGTGGGACCGTGGCTAGGCGTGAGATCCGTCGCTACCAGAAGAGCACCGAGCTGCTCATCCGCAAGCTCCCCTTCCAGCGTCTGGTATG----------------------------------------------------------------------------CAGGTTCGTGAGATCGCCCAGGACTTCAAGTCCGACCTGCGCTTCCAGTCTTCCGCCATCGGTGCCCTGCAGGAGTCCGTCGAGTCCTACCTCGTCTCCCTCTTCGAGGACACCAACCTGTGCGCCATCCACGCCAAGCGTGTCACCATCCAGTCGGTACGTC

>Diaporthe_amygdali_CBS_126679

TCCGCGCCCTCCACCGGAGGTGTCAAGAAGCCTCACCGCTACAAGCCTGGTACCGTCGCTCTGCGTGAGATTCGTCGCTACCAGAAGTCCACTGAGCTTCTGATCCGCAAGCTGCCCTTCCAGCGTCTGGTACG----------------------------------------------------------------------------CAGGTTCGTGAGATTGCCCAGGACTTCAAGTCCGACCTCCGCTTCCAGTCCTCCGCCATCGGTGCCCTGCAGGAGTCCGTCGAGTCCTACCTCGTCTCCCTCTTCGAGGACACCAACCTGTGCGCCATCCACGCCAAGCGTGTCACCATCCAGTCGGTATGTA

>Diaporthe_batatas_CBS_122_21

TCCGCGCCCTCCACCGGAGGTGTCAAGAAGCCTCACCGCTACAAGCCTGGTACCGTCGCTCTGCGTGAGATCCGTCGCTACCAGAAGAGCACCGAGCTGCTGATCCGCAAGCTCCCCTTCCAGCGTCTGGTATG----------------------------------------------------------------------------CAGGTCCGTGAGATCGCCCAGGACTTCAAGTCCGACCTGCGCTTCCAGTCCTCCGCCATCGGTGCTCTCCAGGAGTCCGTCGAGTCTTACCTCGTCTCCCTCTTTGAGGACACCAACCTGTGCGCCATCCACGCCAAGCGTGTCACCATCCAGTCGGTACGTC

>Diaporthe_endophytica_CBS_133811

TCCGCGCCCTCCACCGGAGGTGTCAAGAAGCCTCACCGCTACAAGCCTGGTACCGTCGCTCTGCGTGAGATCCGTCGCTACCAGAAGAGCACCGAGCTGCTGATCCGCAAGCTCCCCTTCCAGCGTCTGGTATG----------------------------------------------------------------------------CAGGTCCGTGAGATCGCCCAGGACTTCAAGTCCGACCTGCGCTTCCAGTCTTCCGCCATCGGTGCTCTCCAGGAGTCCGTCGAGTCTTACCTCGTCTCCCTCTTTGAGGACACCAACCTGTGCGCCATCCACGCCAAGCGTGTCACCATCCAGTCGGTACGTC

>Diaporthe_ovalispora_ICMP20659

TCCGCGCCCTCCACCGGAGGTGTCAAGAAGCCTCACCGCTACAAGCCTGGTACCGTCGCTCTGCGTGAGATCCGTCGCTACCAGAAGAGCACCGAGCTGCTGATCCGCAAGCTCCCCTTCCAGCGTCTGGTATG----------------------------------------------------------------------------CAGGTCCGTGAGATCGCCCAGGACTTCAAGTCCGACCTGCGCTTCCAGTCTTCCGCCATCGGTGCTCTCCAGGAGTCCGTCGAGTCTTACCTCGTCTCCCTCTTCGAGGACACCAACCTGTGCGCCATCCACGCCAAGCGTGTCACCATCCAGTCGGTACGTC

>Diaporthe_passifloricola_CBS_141329

TCCGCGCCCTCCACCGGAGGTGTCAAGAAGCCTCACCGCTACAAGCCCGGTACCGTCGCTCTGCGTGAGATCCGTCGCTACCAGAAGAGCACCGAGCTGCTGATCCGCAAGCTCCCCTTCCAGCGTCTGGTATG----------------------------------------------------------------------------CAGGTCCGTGAGATCGCCCAGGACTTCAAGTCCGACCTGCGCTTCCAGTCTTCCGCCATCGGTGCTCTCCAGGAGTCCGTCGAGTCTTACCTCGTCTCCCTCTTCGAGGACACCAACCTGTGCGCCATCCACGCCAAG?????????????????????????

>Diaporthe_ueckerae_FAU_656

TCCGCGCCCTCCACCGGAGGTGTCAAGAAGCCTCACCGCTACAAGCCCGGTACCGTCGCTCTGCGTGAGATCCGTCGCTACCAGAAGAGCACCGAGCTGCTGATCCGCAAGCTCCCCTTCCAGCGTCTGGTATG----------------------------------------------------------------------------CAGGTCCGTGAGATCGCCCAGGACTTCAAGTCCGACCTGCGCTTCCAGTCTTCCGCCATCGGTGCTCTCCAGGAGTCCGTCGAGTCTTACCTCGTCTCCCTCTTTGAGGACACCAACCTGTGCGCCATCCACGCCAAGCGTGTCACCATCCAGTCGGTACGTC

>Diaporthe_schini_CBS_133181

TCCGCGCCCTCCACCGGAGGTGTCAAGAAGCCTCACCGCTACAAGCCTGGTACCGTCGCTCTGCGTGAGATCCGTCGCTACCAGAAGAGCACCGAGCTGCTGATCCGCAAGCTCCCCTTCCAGCGTCTGGTATG----------------------------------------------------------------------------CAGGTCCGTGAGATCGCCCAGGACTTCAAGTCCGACCTGCGCTTCCAGTCTTCCGCCATCGGTGCCCTTCAGGAGTCCGTCGAGTCTTACCTCGTCTCCCTCTTTGAGGACACCAACCTGTGCGCCATCCACGCCAAGCGTGTCACCATCCAGTCGGTACGTC

>Diaporthe_terebinthifolii_CBS_133180

TCCGCGCCCTCCACCGGAGGTGTCAAGAAGCCTCACCGCTACAAGCCTGGTACCGTCGCTCTGCGTGAGATCCGTCGCTACCAGAAGAGCACCGAGCTGCTGATCCGCAAGCTCCCCTTCCAGCGTCTGGTATG----------------------------------------------------------------------------CAGGTCCGTGAGATCGCCCAGGACTTCAAGTCCGACCTGCGCTTCCAGTCTTCCGCCATCGGTGCCCTTCAGGAGTCCGTCGAGTCTTACCTCGTCTCCCTCTTTGAGGACACCAACCTGTGCGCCATCCACGCCAAGCGTGTCACCATCCAGTCGGTACGTC

>Diaporthe_longicolla_FAU_599

TCCGCGCCCTCCACCGGAGGTGTCAAGAAGCCTCACCGCTACAAGCCTGGTACCGTCGCTCTGCGTGAGATCCGTCGCTACCAGAAGAGCACCGAGCTGCTGATCCGCAAGCTCCCCTTCCAGCGTCTGGTATG----------------------------------------------------------------------------CAGGTCCGTGAGATCGCCCAGGACTTCAAGTCCGACCTGCGCTTCCAGTCTTCCGCCATCGGTGCCCTGCAGGAGTCCGTCGAGTCTTACCTCGTCTCCCTCTTTGAGGACACCAACCTGTGCGCCATCCACGCCAAGCGTGTCACCATCCAGTCGGTACGTC

>Diaporthe_unshiuensis_CGMCC3_17569

TCCGCGCCCTCCACCGGAGGTGTCAAGAAGCCTCACCGCTACAAGCCTGGTACCGTCGCTCTGCGTGAGATCCGTCGCTACCAGAAGAGCACCGAGCTGCTGATCCGCAAGCTCCCCTTCCAGCGTCTGGTATG----------------------------------------------------------------------------CAGGTCCGTGAGATCGCCCAGGACTTCAAGTCCGACCTGCGCTTCCAGTCTTCCGCCATCGGTGCCCTGCAGGAGTCCGTCGAGTCTTACCTCGTCTCCCTCTTTGAGGACACCAACCTGTGCGCCATCCACGCCAAGCGTGTCACCATCCAGTCGGTACGTC

>Diaporthe_infertilis_CBS_230_52

TCCGCGCCCTCCACCGGAGGTGTCAAGAAGCCTCACCGCTACAAGCCTGGTACCGTCGCTCTGCGTGAGATCCGTCGCTACCAGAAGAGCACCGAGCTGCTGATCCGCAAGCTCCCCTTCCAGCGTCTGGTATG----------------------------------------------------------------------------CAGGTCCGTGAGATCGCCCAGGACTTCAAGTCCGACCTGCGCTTCCAGTCTTCCGCCATCGGTGCGCTCCAGGAGTCCGTCGAGTCTTACCTCGTCTCCCTCTTTGAGGACACCAACCTGTGCGCCATCCACGCCAAGCGTGTCACCATCCAGTCGGTACGTC

>Diaporthe_phaseolorum_CBS_113425

TCCGCGCCCTCCACCGGAGGTGTCAAGAAGCCTCACCGCTACAAGCCTGGTACCGTCGCTCTGCGTGAGATCCGTCGCTACCAGAAGAGCACCGAGCTGCTGATCCGCAAGCTCCCCTTCCAGCGTCTGGTATG----------------------------------------------------------------------------CAGGTCCGTGAGATCGCCCAGGACTTCAAGTCCGACCTGCGCTTCCAGTCTTCCGCCATCGGTGCTCTCCAGGAGTCCGTCGAGTCTTACCTCGTGTCCCTCTTCGAGGACACCAACCTGTGCGCCATCCACGCCAAGCGTGTCACCATCCAGTCGGTACGTC

>Diaporthe_sojae_CBS_139282

TCCGCGCCCTCCACCGGAGGTGTCAAGAAGCCTCACCGCTACAAGCCTGGTACCGTCGCTCTGCGTGAGATCCGTCGCTACCAGAAGAGCACCGAGCTGCTGATCCGCAAGCTCCCCTTCCAGCGTCTGGTATG----------------------------------------------------------------------------CAGGTCCGTGAGATCGCCCAGGACTTCAAGTCCGACCTGCGCTTCCAGTCTTCCGCCATCGGTGCTCTCCAGGAGTCCGTCGAGTCTTACCTCGTGTCCCTCTTCGAGGACACCAACCTGTGCGCCATCCACGCCAAGCGTGTCACCATCCAGTCGGTACGTC

>Diaporthe_melonis_CBS_507_78

TCCGCGCCCTCCACCGGAGGTGTCAAGAAGCCTCACCGCTACAAGCCTGGTACCGTCGCTCTGCGTGAGATCCGTCGCTACCAGAAGAGCACCGAGCTGCTGATCCGCAAGCTCCCCTTCCAGCGTCTGGTACG----------------------------------------------------------------------------CAGGTCCGTGAGATCGCCCAGGACTTCAAGTCCGACCTGCGCTTCCAGTCTTCCGCCATCGGTGCTCTTCAGGAGTCCGTCGAGTCTTACCTCGTCTCCCTCTTTGAGGACACCAACCTGTGTGCCATCCACGCCAAGCGTGTCACCATCCAGTCGGTACGTC

>Diaporthe_convolvuli_CBS_124654

TCCGCGCCCTCCACCGGAGGTGTCAAGAAGCCTCACCGCTACAAGCCTGGTACCGTCGCTCTGCGTGAGATCCGTCGCTACCAGAAGAGCACCGAGCTGCTGATCCGCAAGCTCCCCTTCCAGCGTCTGGTATG----------------------------------------------------------------------------CAGGTCCGTGAGATCGCCCAGGACTTCAAGTCCGACCTGCGCTTCCAGTCTTCCGCCATCGGTGCTCTTCAGGAGTCCGTCGAGTCTTACCTCGTCTCTCTCTTCGAGGACACCAACCTGTGCGCCATCCATGCCAAGCGTGTCACCATCCAGTCGGTACGTT

>Diaporthe_hordei_CBS_481_92

TCCGCGCCATCCACCGGAGGTGTCAAGAAGCCTCACCGCTACAAGCCTGGTACCGTCGCTCTGCGTGAGATCCGTCGCTACCAGAAGAGCACTGAGCTGCTGATCCGCAAGCTCCCCTTCCAGCGCCTGGTATG----------------------------------------------------------------------------CAGGTCCGTGAGATCGCCCAGGACTTCAAGTCCGACCTGCGCTTCCAGTCTTCCGCCATCGGTGCCCTTCAGGAGTCCGTCGAGTCTTACCTCGTCTCCCTCTTTGAGGACACCAACCTGTGCGCCATCCACGCCAAGCGTGTCACCATCCAGTCGGTACGTC

>Diaporthe_tecomae_CBS_100547

TCCGCGCCTTCCACCGGAGGTGTCAAGAAGCCTCACCGCTACAAGCCTGGTACTGTTGCTCTGCGTGAGATCCGTCGCTACCAGAAGAGCACCGAGCTGCTGATCCGCAAGCTCCCTTTCCAGCGTCTGGTATG----------------------------------------------------------------------------CAGGTCCGTGAGATCGCCCAGGACTTCAAGTCCGACCTGCGCTTCCAGTCCTCCGCCATCGGTGCCCTTCAGGAGTCCGTCGAGTCTTACCTCGTCTCCCTCTTTGAGGACACCAACCTGTGCGCCATCCACGCCAAGCGTGTCACCATCCAGTCGGTACGTC

>Diaporthe_racemosae_CBS_143770

TCCGCGCCCTCCACCGGAGGTGTCAAGAAGCCTCACCGCTACAAGCCTGGTACCGTCGCTCTGCGTGAGATCCGTCGCTACCAGAAGAGCACCGAGCTGCTGATCCGCAAGCTCCCCTTCCAGCGTCTGGTATG----------------------------------------------------------------------------TAGGTCCGTGAGATCGCCCAGGACTTCAAGTCCGACCTGCGCTTCCAGTCTTCTGCCATCGGTGCCCTCCAGGAGTCTGTCGAGTCTTACCTCGTCTCCCTCTTTGAGGACACCAACCTGTGCGCCATCCACGCCAAGCGTGTCACCATCCAGTCGGTACGTA

>Diaporthe_megalospora_CBS_143_27

TCCGCGCCCTCCACCGGAGGTGTCAAGAAGCCTCACCGCTACAAGCCTGGTACCGTCGCTCTGCGTGAGATCCGTCGCTACCAGAAGAGCACCGAGCTGTTGATCCGCAAGCTCCCCTTCCAGCGTCTGGTACG----------------------------------------------------------------------------TAGGTCCGTGAGATCGCCCAGGACTTCAAGTCCGACCTGCGCTTCCAGTCTTCCGCCATCGGTGCCCTTCAGGAGTCCGTCGAGTCTTACCTCGTCTCCCTCTTCGAGGACACCAACCTGTGCGCCATCCACGCCAAGCGTGTCACCATCCAGTCGGTACGTC

>Diaporthe_vexans_CBS_127_14

TCCGCGCCCTCCACCGGAGGTGTCAAGAAGCCTCACCGCTACAAGCCTGGTACCGTCGCTCTGCGTGAGATCCGTCGCTACCAGAAGAGCACTGAGCTGCTGATCCGCAAGCTCCCCTTCCAGCGTCTGGTATG----------------------------------------------------------------------------TAGGTCCGTGAGATCGCCCAGGACTTCAAGTCCGACCTGCGCTTCCAGTCCTCCGCCATCGGTGCCCTTCAGGAGTCGGTCGAGTCCTACCTCGTCTCCCTCTTTGAGGACACCAACCTGTGCGCCATCCACGCCAAGCGTGTCACCATCCAGTCGGTACGTC

>Diaporthe_eres_CBS_138594

TCCGCGCCCTCCACCGGAGGTGTCAAGAAGCCTCACCGCTACAAGCCTGGTACCGTCGCTCTGCGTGAGATCCGTCGCTACCAGAAGAGCACCGAGCTGCTGATCCGCAAGCTCCCCTTCCAGCGTCTGGTATG----------------------------------------------------------------------------TAGGTCCGTGAGATCGCCCAGGACTTCAAGTCCGACCTCCGCTTCCAGTCTTCCGCCATCGGTGCCCTGCAGGAGTCGGTTGAGTCTTACCTCGTCTCCCTCTTCGAGGACACCAACCTGTGCGCCATCCACGCCAAGCGTGTCACCATCCAGTCGGTACGTT

>Diaporthe_helianthi_CBS_592_81

TCCGCGCCCTCCACCGGAGGTGTCAAGAAGCCCCACCGCTACAAGCCTGGTACCGTCGCTCTGCGTGAGATCCGTCGTTATCAGAAGAGCACCGAGCTGCTGATTCGCAAGCTCCCCTTCCAGCGTCTGGTATG----------------------------------------------------------------------------TAGGTTCGTGAGATCGCCCAGGACTTCAAGTCCGATCTCCGCTTCCAGTCTTCCGCCATCGGTGCCCTGCAGGAGTCTGTCGAGTCTTACCTCGTCTCCCTCTTTGAGGACACCAACCTGTGCGCCATCCACGCCAAGCGTGTCACCATCCAGTCGGTATGTC

>Diaporthe_fructicola_MAFF_246408

TCCGCGCCCTCCACCGGAGGTGTCAAGAAGCCTCACCGCTACAAGCCTGGTACCGTCGCTCTGCGTGAGATCCGTCGCTACCAGAAGAGCACCGAGCTGCTGATCCGCAAGCTCCCCTTCCAGCGTCTGGTATGTTTTCACACCCACCCAAAATCAA-----TCAACTTCACCCTCGTTTACCCTGCTGACCGCCG--CCTCTTCCTCCCCAGGTCCGTGAGATCGCCCAGGACTTCAAGTCCGACCTGCGCTTCCAGTCTTCCGCCATCGGTGCTCTCCAGGAGTCCGTCGAGTCTTACCTCGTCTCCCTCTTCGAGGACACCAACCTGTGCGCCATCCACGCCAAGCGTGTCACCATCCAGTCGGTACGTC

>Diaporthe_heterostemmatis_SAUCC194_85

TCCGCGCCCTCCACCGGAGGTGTCAAGAAGCCTCACCGCTACAAGCCTGGTACCGTCGCTCTGCGTGAGATCCGTCGCTACCAGAAGAGCACCGAGCTGCTGATCCGCAAGCTCCCCTTCCAGCGTCTGGTATGTTTTCACACCCACCCAAAATCAA-----TCAACTTCACCCTCGTTTACCCTGCTGACCGCCG--CCTCTTCCTCCCCAGGTCCGTGAGATCGCCCAGGACTTCAAGTCCGACCTGCGCTTCCAGTCTTCCGCCATCGGTGCTCTCCAGGAGTCCGTCGAGTCTTACCTCGTCTCCCTCTTCGAGGACACCAACCTGTGCGCCATCCACGCCAAGCGTGTCACCATCCAGTCGGTACGTC

>Diaporthe_vochysiae_LGMF1583

TCCGCGCCCTCCACCGGAGGTGTCAAGAAGCCTCACCGCTACAAGCCCGGTACCGTCGCTCTGCGTGAGATCCGTCGCTACCAGAAGAGCACCGAGCTGCTGATCCGCAAGCTCCCCTTCCAGCGTCTGGTATGTTCTGCACCCCACCAACCATCCTCGCTGTCGCCCTCACCCTCGTACACCCTGCTGACTGTCG--CGCCTCCCTCTCCAGGTCCGTGAGATCGCCCAGGACTTCAAGTCCGACCTGCGCTTCCAGTCTTCCGCCATCGGTGCTCTCCAGGAGTCCGTCGAGTCTTACCTCGTCTCCCTCTTCGAGGACACCAACCTGTGCGCCATCCACGCCAAGCGTGTCACCATCCAGTCGGTACGTC

>Diaporthe_cinnamomi_CFCC_52569

TCCGCGCCCTCCACCGGAGGTGTCAAGAAGCCTCACCGCTACAAGCCTGGTACCGTCGCTCTGCGTGAGATCCGTCGCTACCAGAAGAGCACCGAGCTGCTGATCCGCAAGCTCCCCTTCCAGCGTCTGGTATGTCT-GCAACCG-CCAAAGA-----------ACCCGTGCTCCCCACTCTCCTGCTGACCGTCG---CCTTCTCTCCCCAGGTTCGTGAGATCGCCCAGGACTTCAAGTCCGACCTGCGCTTCCAGTCTTCCGCCATCGGTGCCCTGCAGGAGTCCGTCGAGTCCTACCTCGTCTCCCTCTTTGAGGACACCAACCTGTGCGCCATCCACGCCAAGCGTGTCACCATCCAGTCGGTACGTT

>Diaporthe_caryae_CFCC_52563

TCCGCGCCCTCCACCGGAGGTGTCAAGAAGCCTCACCGCTACAAGCCTGGTACCGTCGCTCTGCGTGAGATCCGTCGCTACCAGAAGAGCACCGAGCTGCTGATCCGCAAGCTCCCCTTCCAGCGTCTGGTATGTCT-GCACCCG-CCAAAC-------------CCCTTCGTGCTACTTTCCCTGCTGACCGCCG-CCCTCTCTGTTTCCAGGTCCGTGAGATCGCCCAGGACTTCAAGTCCGACCTGCGCTTCCAGTCTTCCGCCATCGGTGCCCTTCAGGAGTCCGTCGAGTCTTACCTCGTCTCCCTCTTTGAGGACACCAACCTGTGCGCCATCCACGCCAAGCCAGTCACCATCCAGTCGGTACGTC

>Diaporthe_machili_SAUCC194_111

TCCGCGCCCTCCACCGGAGGTGTCAAGAAGCCTCACCGCTACAAGCCTGGTACCGTCGCTCTGCGTGAGATCCGTCGCTACCAGAAGAGCACCGAGCTGCTGATCCGCAAGCTCCCCTTCCAGCGTCTGGTATGTC--GCACCCG-CCAAAC-------------CCCCTCGTGCTACTTCCCCTGCTGACCGTCG-CCCTCTTTGCTTCCAGGTCCGTGAGATCGCCCAGGACTTCAAGTCCGACCTGCGCTTCCAGTCTTCCGCCATCGGTGCCCTTCAGGAGTCCGTCGAGTCTTACCTCGTCTCCCTCTTTGAGGACACCAACCTGTGCGCCATCCACGCCAAGCGTGTCACCATCCAGTCGGTACGTC

>Diaporthe_rossmaniae_CAA762

TCCGCGCCCTCCACCGGAGGTGTCAAGAAGCCTCACCGCTACAAGCCTGGTACCGTCGCTCTGCGTGAGATCCGTCGCTACCAGAAGAGCACCGAGCTGCTGATCCGCAAGCTGCCCTTCCAGCGTCTGGTATGTCT-GCACCTGCCCAAACT-------------CACTCGCCCTGCTCCCCCTGCTGACCGTCG-CCCGCTTTCCCTCTAGGTCCGTGAGATCGCCCAGGACTTCAAGTCCGACCTGCGCTTCCAGTCTTCCGCCATCGGTGCCCTTCAGGAGTCCGTCGAGTCTTACCTCGTCTCCCTCTTTGAGGACACCAACCTGTGCGCCATCCACGCCAAGCGTGTCACCATCCAGTCGGTACGTC

>Diaporthe_alangii_CFCC_52556

TCCGCGCCCTCCACCGGAGGTGTCAAGAAGCCTCACCGCTACAAGCCTGGTACCGTCGCTCTGCGTGAGATCCGTCGCTACCAGAAGAGCACCGAGCTGCTGATCCGCAAGCTCCCCTTCCAGCGTCTGGTATGGCT--GCACCGTCCCAATAGCGC-------CCGCGCCCTCCTTATTCTCCTGCTGACCGCCT-------CCTCTTCCAGGTCCGTGAGATCGCCCAGGACTTCAAGTCCGACCTGCGCTTCCAGTCTTCTGCCATTGGTGCCCTGCAGGAGTCCGTCGAGTCTTACCTCGTCTCTCTCTTCGAGGACACCAACCTGTGCGCCATCCACGCCAAGCGTGTCACCATCCAGTCGGTACGTT

>Diaporthe_guttulata_CGMCC_3_20100

TCCGCGCCCTCCACCGGAGGTGTCAAGAAGCCTCACCGCTACAAGCCTGGTACCGTCGCTCTGCGTGAGATCCGTCGCTACCAGAAGAGCACCGAGCTGCTGATCCGCAAGCTCCCCTTCCAGCGTCTGGTAAGTCC--TGCATCACAAACTGTCAC------GCCTCTTGCCCTTGCCCTTGCTGACCATCGCCC---TTCTTCTCTTGCAGGTTCGTGAGATCGCCCAGGACTTCAAGTCCGACCTGCGCTTCCAGTCTTCCGCCATCGGTGCCCTGCAGGAGTCCGTCGAGTCTTACCTCGTCTCCCTGTTCGAGGACACCAACCTGTGCGCCATCCACGCCAAGCGTGTCACCATCCAGTCGGTACGTC

>Diaporthe_sambucusii_CFCC_51986

TCCGCGCCCTCCACCGGAGGTGTCAAGAAGCCTCACCGCTACAAGCCTGGTACCGTCGCCCTGCGTGAGATCCGTCGCTACCAGAAGAGCACCGAGCTGCTCATCCGCAAGCTCCCCTTCCAGCGTCTGGTATGTTT--GCGCCTGCCAATCGGCCT-----CCACCCCGCTCCTGCTCCCGTCCCCTTGCTGACAGCCGTCCCCTCTTGCAGGTCCGTGAGATCGCCCAGGACTTCAAGTCCGACCTGCGCTTCCAGTCTTCCGCCATCGGTGCCCTTCAGGAGTCCGTCGAGTCCTACCTCGTCTCCCTCTTCGAGGACACCAACCTGTGCGCCATCCACGCCAAGCGTGTCACCATCCAGTCGGTACGTC

>Diaporthe_cerradensis_CMRP4331

TCCGCGCCCTCTACCGGAGGTGTCAAGAAGCCTCACCGCTACAAGCCTGGTACCGTCGCTCTGCGTGAGATCCGTCGCTACCAGAAGAGCACCGAGCTGCTGATCCGCAAGCTCCCCTTCCAGCGTCTGGTATGTCT--GCACTTGCCAATCA--------------CCGCCCCGACCCGCCAATTCCTGCTGACCATCGCCTCCTTTCCCAGGTTCGTGAGATCGCCCAGGACTTCAAGTCCGACCTGCGCTTCCAGTCTTCCGCCATCGGTGCCCTGCAGGAGTCCGTCGAGTCGTACCTCGTCTCCCTCTTCGAGGACACCAACCTGTGTGCCATCCACGCCAAGCGTGTCACCATCCAGTCGGTATGTC

# Alignment of the *tef1* sequences used in the second phylogenetic study

>Diaporthe_pseudolongicolla_CBS_117165

NNNNNNNNNNGTCAGTAAATAT--CGCACA---------TCCCACAGCATG--AACT--CCA-C-CAAG---CCCACCGTGCGCAC-------ATCGTCATCATTG--GTGCG----CGAC-TGCCTGCGCGGCTTCAT--CACCC----CTCAAACCA-TTTTCACCCCTCCCTCTGGGTTTTTTTTCCATT---TTCAGTGC-------------------GGGTGCGGGGTGCGCTTATCA-G----CCGCTTATCTCCT--A--TGCAAAA---------CCCTGCT---GGCATTA--ACCACTCCTT--------GCCACCG-CCAACACCAACTCTACCACTTTCAACCCTATCAATCTCCCAGGCGAAACG---AACTGTCAA--------GAATATGAAGCTGACTTAC----TCTCCACACAGCCGCCGAGCTGGGTAAGGG

>Diaporthe_novem_CBS_127271

GAGAAGGAAGGTCAGTAAATAT--CGCACA---------TCCCACAGCATG--AACT--CCA-C-CAAG---CCCACCGTGCGCAC-------ATCGTCATCATTG--GTGCG----CGAC-TGCCTGCGCGGCTTCAT--CACCC----CTCAAACCA-TTTTCACCCCTCCCTCTGGGTTTTTTTTCCATT---TTCAGTGC-------------------GGGTGCGGGGTGCGCTTATCA-G----CCGCTTATCTCCT--A--TGCAAAA---------CCCTGCT---GGCATTA--ACCACTCCTT--------GCCACCG-CCAACACC--------------------------------------------------------------GAATATGAAGCTGACTTAC----TCTCCACACAGCCGCCGAGCTGGGTAAGGG

>Diaporthe_angelicae_CBS_111592

GAGAAGGAAGGTCAGTAAACAT--CACACATCCAACCCTTCCCACAGCATC--AACT--CCA-C--AAG---TCCTCCATGCGCAC-------ATTGTCATCATTG--GTGCG----CGAC-TGCCTGCGCGGCTTGGTCACACCC----GTCAAGCCA-TTTTCACCCCT-CCTCTGGG--TTTTTTCCATT---TTCAGTGC-------------------GGGTGCGGGGTGCGGTTATCAGG----CCGCTTATCTCCCGCA--CGCAAAA---------CCCTGCT---GGCATTA--ACCACTCCCT--------ACCACCG-TCAACACC--------------------------------------------------------------GAACATGACGCTGACTTAA----TTTCCACACAGCCGCCGAGCTGGGCAAGGG

>Diaporthe_arctii_CBS_136_25

GAGAAGGAAGGTCAGTAAACAT--CACACATCCAACCCTTCCCACAGCATC--AACT--CCA-C-AAAG---CCCTCCATGCGCAC-------ATTGTCATCATTG--GTGCG----CGAC-TGCCTGCGCGGCTTCGTCACACCC----GTCAAGCCA-TTTTCACCCCTCCCTCTGGG--TTTTTTCCATT---TTCAGTGC-------------------GGGTGCGGGGTGCGCTTATCAGG----CCGCTTATCTCCCACA--CGCAAAA---------CCCTGCT---GGCATTA--ACCACTCCCT--------ACCACCG-TCAACACC--------------------------------------------------------------GAACATGACGCTGACTTGA----TTTCCATACAGCCGCCGAGCTGGGCAAGGG

>Diaporthe_cucurbitae_DAOM_42078

GAGAAGGAAGGTCAGTAAACAT--CACACATCCAACCCTTCCCACAGCATC--AACT--CCA-C-AAAG---CCCTCCATGCGCAC-------ATTGTCATCATTG--GTGCG----CGAC-TGCCTGCGCGGCTTCGTCACACCC----GTCAAGCCA-TTTTCACCCCTCCCTCTGGG--TTTTTTCCATT---TTCAGTGC-------------------GGGTGCGGGGTGCGCTTATCAGG----CCGCTTATCTCCCACA--CGCAAAA---------CCCTGCT---GGCATTA--ACCACTCCCT--------ACCACCG-TCAACACC--------------------------------------------------------------GAACATGACGCTGACTTAA----TTTCCATACAGCCGCCGAGCTGGGTAAGGG

>Diaporthe_stewartii_CBS_193_36

NNNNNNNAAGGTCAGTAAACAT--CACACATCCAACCCTTCCCACAGCATC--AACT--CCA-C-AAAG---CCCTCCATGCGCAC-------ATTGTCATCATTG--GTGCG----CGAC-TGCCTGCGCGGCTTCGTCACACCC----GTCAAGCCA-TTTTCACCCCTCCCTCTGGG--TTTTTTCCATT---TTCAGTGC-------------------GGGTGCGGGGTGCGCTTATCAGG----CCGCTTATCTCCCACA--CGCAAAA---------CCCTGCT---GGCATTA--ACCACTCCCT--------ACCACCG-TCAACACC--------------------------------------------------------------GAACATGACGCTGACTTAA----TTTCCATACAGCCGCCGAGCTGNNNNNNNN

>Diaporthe_gulyae_BRIP_54025

NAGAAGGAAGGTCAGTAAACAT--CACACATCCAACACTTCCCACAGTATC--AACT--CCA-C-AAAG---TACTCCATGCGCAC-------ATTGTCATCATTG--GTGCG----CGAC-TGCCTGCGCGGCTTCGTCACACCC----GTCAAGCCA-TTTTCACCCCTCCCTCTGGG--TTTTTTCCATT---TTCAGTGC-------------------GGGTGCGGGGTGCGCTTATCAGG----CCGCTTATCTCCCACA--CGCAAAA---------CCCTGCT---GGCATTA--ACCACTCCCT--------ACCACCG-TCAACACC--------------------------------------------------------------GAACATGACGCTGACTTAA----TTTTCATACAGCCGCCGAGCTGGGCAAGGG

>Diaporthe_subordinaria_CBS_101711

GAGAAGGAAGGTCAGTAAACAT--CACACATCCAACCCTTCCCACAGCATC--AACT--CCA-C-AAAG---CCCTCCATGCGCAC-------ATTGTCATCATTG--GTGCG----CGAC-TGCCTGCGCGGCTTCGTCACACCC----GTCAAGCCA-TTTTCACCCCTCCCTCTGGG--TTTTTTCCATT---TTCAGTGC-------------------GGGTGCGGGGTGCGCTTATCAGG----CCGCTTATCTCCCACC--CGCAAAA---------CCCTGCT---GGCATTA--ACCACTCCCT--------ACCACCG-TCAACACC--------------------------------------------------------------GAACATGACGCTGACTTAAAT--TTCCCATACAGCCGCCGAGCTGGGTAAGGG

>Diaporthe_cuppatea_CBS_117499

NNNNNNNNNNGTCAGTAAACAT--CACACA---------TCCCACAGCATG--AACT--CCA-C-AAAG---TCCTCCATGCGCAC-------ATTGCCATCATAG--GTGCG----CGAC-TGTCTGCGCGGCTTCGTCACACCC----GTCAAGCCA-TTTTCACCCCTCCCTCTGGG--TTTTTTCCATT---TTCAGTGC-------------------GGGTGCGGGGTGCGCTTATCAGG----CCGCTTATCTCCTACA--CGCAAAA---------CCCTGCT---GGCAATA--ACCACTCCTT--------CCCGTCG-CCAACACC--------------------------------------------------------------GAATATGACGCTGACTCAC----TTTCCACACAGCCGCCGAGCTGGGTAAGGG

>Diaporthe_lusitanicae_CBS_123212

GAGAAGGAAGGTCAGTAAACAT--CACGCA---------TCCCACAGCATG--AACT--CCA-C-AAAG---TCCTCCATGCGCAC-------ATTGTCATCATTG--GTGCG----CGAC-TGCCTGCGCGGCTTCGT--CACCC----GTCAAGGCA-TTTTCACCCCTCCCTCTGGG-TTTTTTTTCATT---TTCAGTGC-------------------GGGTGCGGGGTGCGCTTATCAGG----CCGCTTATCTCCTACA--CGCAAAA---------CCCTGCT---GGCATTA--ACCACTCCTT--------ACCACCG-CCAACACC--------------------------------------------------------------GAAAATGACGCTGACTTAC----TTTTCACCCAGCCGCCGAGCTGGGTAA-GG

>Diaporthe_beilharziae_BRIP_54792

NNGAAGGAAGGTCAGTTAATAT--CACACA---------TGCCGCATCATG--AACC--CCA-T-GAAGC--CCCCCCACACGCAC-------ATCCTCACCATGG--GCGCG----CGAC-TGTTTGCGCGGCTGCGTCACACCC----GTCAAGCCA-TTTTCACCCCTCCCTCTGGA---TTTTTCCATT---TTCAGTGC-------------------GGGTGCGGGGTGCGCTTATCAGG----CCGCTTATCTTCCACA--CGCAAAA---------CCCTGCT---GGCGTCA--ATCACTTCCT--------TGCACTG-CCAGCACC--------------------------------------------------------------GACAATCATACTGACTTAC----TTCCCACACAGCCGCCGAGCTGGGCAAGGG

>Diaporthe_infecunda_CBS_133812

GAGAAGGAAGGTCAGTAAATAT--CACACA---------TGCCGCATCATG--AACC--CCA-T-CAAG---TCCCCCACACGCAC-------ATCGTCACCATGG--GCGCG----CGAC-TGTTTGCGCGGCTGCGTCGCACCC----TTCAAGCCA-TTTTCACCCCTCCCTCTGGA---TTTTTCCATT---TTCAGTGC-------------------GGGTGCGGGGTGCGCTTATCAGG----CCGCTTATCTTCCACA--TGCAAAA---------CCCTGCT---GGTGTCA--ATCACTTCCT--------TGCACTG-CCAGCACC--------------------------------------------------------------GACAATCATGCTGACTTAC----TTCCCACACAGCCGCCGAGCTGGGTAAGGG

>Diaporthe_leucospermi_CBS_111980

NNNNNNNAAGGTCAGTAAATAT--CACACA---------TGCCGCATCATG--AACC--CCA-T-CAAG---TCCCCCACACGCAC-------ATCGTCACCATGG--GCGCG----CGAC-TGTTTGCGCGGCTGCGTCGCACCC----TTCAAGCCA-TTTTCACCCCTCCCTCTGGA---TTTTTCCATT---TTCAGTGC-------------------GGGTGCGGGGTGCGCTTATCAGG----CCGCTTATCTTCCACA--TGCAAAA---------CCCTGCT---GGCGTCA--TTCACCTCCT--------TGCACTG-CCAGCACC--------------------------------------------------------------GACAATCATGCTGACTCAC----TTCCCACACAGCCGCCGAGCTGNNNNNNNN

>Diaporthe_pyracanthae_CBS142384

NNNNNNNAAGGTCAGTAAATAT--TACACA---------TGCCGCATCATG--AACC--CCA-T-CAAG---TCCCCCACACGCAC-------ATCGTCACCATGG--GCGCG----CGAC-TGTTTGCGCGGCTGCGTCGCACCC----TTCAAGCCA-TTTTCACCCCTCCCTCTGGA---TTTTTCCATT---TTCAGTGC-------------------GGGTGCGGGGTGCGCTTATCAGG----CCGCTTATCTTCCACA--TGCAAAA---------CCCTGCT---GGCGTCA--TTCACCTCCT--------TGCACTG-CCAGCACC--------------------------------------------------------------GACAATCATGCTGACTCAC----TTCCCACACAGCCGCCGAGCTGNNNNNNNN

>Diaporthe_serafiniae_BRIP_55665a

NNNAAGGAAGGTCAGTAAACAT--CACACA---------TGCCGCATCATG--AACC--ACA-T-GAAG---TCCCCCACACGCAC-------ATCGTCACCATGG--GCGCG----CGAC-TGCTTGCGCGGCTGCGTCACACCC----GTCAAGCCA-TTTTCACCCCTCCCTCTGGA---TTTTTCCATT---TTCAGTGC-------------------GGGTGCGGGGTGCGCTTATCAGG----CCGCTTATCTCCCACACGCGCAAAA---------CCCTGCC---GGTATCA--ATCACTTCCT--------TGCACTG-CCAGCACC--------------------------------------------------------------GACAATCATGCTGACTTAC----TTCC--CACAGCCGCCGAGCTGGGCAAGGG

>Diaporthe_middletonii_BRIP_54884e

NAGAAGGAAGGTCAGTAAACAT--CACACA---------TCCCGCATCATG--AACC--CCA-T-GAAC---CCCGCCTCACGCAC-------ATCGTCACCATGG--GCGCG----CGAC-TTTTTGCGCGGCTGCGT--CACCC----GTCAAGCCA-TTTTCGCCCCTCCCTCTGGA---TTTTTCCATT---TTCAGTGC-------------------GGGTGCGGGGTGTGCTTATCAGG----CCGCTTATCTCCCACA--CGCAAAA---------CCCTGCT---GGCATCA--ATCACTTCCT--------TGCACTG-CCAGCTCC--------------------------------------------------------------GACAATCATGCTGACTTAC----TTCCCACACAGCCGCCGAGCTGGGCAAGGG

>Diaporthe_sackstonii_BRIP_54669b

NAGAAGGAAGGTCAGTAAACAT--TACACA---------TCCCGCATCATG--AACC--CCA-T-GAAC---TCCGCCTCACGCAC-------ATCGTCACCATGG--GCGCG----CGAC--TCTTGCACGGCTGCGTCACACCC----GTCAAGCCA-TTTTCGCCCCTCCCTCTGAA---TTTTTCCATT---TTCAGTGC-------------------GAGTGCGGGGTGTGCTTATCAGG----CCGCTTATCTCCCACA--CGCAAAA---------CCCTGCT---GGCATTA--ATCACTTCCT--------TGCACTG-CCTGCTCC--------------------------------------------------------------GACAATCATGCTGACTTAC----ATCTCACACAGCCGCCGAGCTGGGCAAGGG

>Diaporthe_caryae_CFCC_52563

GAGAAGGAAGGTCAGTAAACAT--TACACA---------TCCCGCATCATG--AACC--CCA-T-GAAC---TCCGCCTGACGCAC-------ATCGTCACCATGG--GCGCG----CGAC-TTTTTGCACGGCTGCGTCACACCC----GTCAAGCCA-TTTTCGCCCCTCCCTCTGGA---TTTTTCCATT---TTCAGTGC-------------------GGGTGCGGGGTGTGCTTATCAGG----CCGCTTATCTCCCACA--CGCAAAA---------CCCTGCT---GGCATCA--ATCACTTCCT--------TGCACTG-CCTGCTCCAACATCACAGGCATCAAACACCATCAACCTCTACTTGCGCAACA-AACTGTTCG--------GACAATCATGCTGACTTAC----ATCCCACACAGCCGCCGAGCTGGGTAAGGG

>Diaporthe_machili_SAUCC194_111

NNNNNNNNNNNNNNNGATTCTT--CTTAAA---------TCCCGCATCATG--AACC--CCA-T-GAAC---TCCGCCTCACGCAC-------ATCGTCACCATGG--GCGCG----CGACTTTTTTGCACGGCTGCGTCACACCC----GTCAAGCCA-TTTTCGCCCCTCCCTCTGGA---TTTTTCCATT---TTCAGTGC-------------------GGGTGCGGGGTGTGCTTATCTGG----CGGCTTATCTCCCACA--CGCAAAA---------CCCTGCT---GGCATCA--ATCACTTCCT--------TGCACTG-CCTGCTCCAACATCACAAACATCAAACACCATCAACCTCTACTTGCGCAACA-ATCTGTTCG--------GACAATCATGCTGACTTAC----ATCCCACACAGCCGCCGAGCTGNNNNNNNN

>Diaporthe_pachirae_CDA_728

NNNNNNNAAGGTCAGTAAATAT--CACACA---------TGCCGCATCATG--AACC--CCA-T-CAAG---TCCCCCACACGCAC-------ATCGTCACCATGG--GCGCG----CGAC-TGTTTGCGCGGCTGCGTCGCACCC----TTCCAGCCA-TTTTCACCCCTCCCTCTGGA---TTTTTCCATT---TTCAGTGC-------------------GGGTGCGGGGTGCGCTTATCAGG----CCGCTTATCTTCCACA--TGCGAAA---------CCCTGCT---GGCGTCA--ATCACTTCCT--------TGCACTG-CCAGCACCAACGTCACAAACATCAAACCCCATCAACCTCTACTTGCGCGACAGAACTGTTCG--------GACAATCATGCTGACTTAC----TTCCCACACAGCCGCCGAGCTGGGCAAGGG

>Diaporthe_rossmaniae_CAA762

NNNNNNNAAGGTCAGTAAATAT--TACACA---------TGCCGCATCATG--AACC--CCA-T-CAAG---T-CCCCACACGCAC-------ATCGTCACCATGG--GCGCG----CGAC-TGTTTGCGCGGCTGCGTCGCACCC----TTCAAGCCA-TTTTCACCCCTCCCTCTGGA---TTTTTCCATT---TTCAGTGC-------------------GGGTGCGGGGTGCGCTTATCAGG----CCGCTTATCTTCCACA--TGCAAAA---------CCCTGCT---GGTGTCA--ATCACCTCCT--------TGCACTG-CCAGCACCAACGTCACAAACCTCAAACCCCATCAACCTCTACTTGCGCGACAGAACTGTTCG--------GACAATCATGCTGACTCAC----TTCCCACACAGCCGCCGAGCTGGGCAAGGG

>Diaporthe_neoarctii_CBS_109490

GAGAAGGAAGGTCAGTAAACAT--CCCACA---------TCCCACAGCATC--ATTT--CCT-C-AAGGCCATCCTCCATGTGCAC-------ATTGTCATCATTG--GCGCGCGACCGAC-CGCCTGCGCGGCTTCGTCACACCC----GTCAAGCCA-TTTTCACCCCTCCCTCTGGG--TTTTTTCCATT---TTCAGTGCGGGC---------------GGGTGCGGGGTGTGCTTATCAGG----CCGCTTATCT-CCACA--CGCAAAA---------CCCTGCT---GGCATTA--ACCACGCCTT--------ACCACCG-CCAACACC--------------------------------------------------------------GAATATGACGCTGACGTAA----TTTCCACACAGCCGCCGAGCTGGGTAAGGG

>Diaporthe_myracrodruonis_URM7972

NNNNNNNNNNNNNNNNNNNNNNNNNNNNNNNNNNNNNNNNNNNNNNNNNNNNNNNNNNNNNNNNNNNNNNNNNNNNNNNNNNNNNNNNNNNNNNNNNNNNNNNNNNNNNNNNNNNNNNNNNNNNNNNNNNNNNNNGTGTCACACCC----GTCATGCCA-TTTTCACCCCTCCCTCTGGA---TTTTTCCATT---TTCAGTGC-------------------GGGTGCGGGGTGCGCTTATCAGG----CCGCTTATCTCCCACA--CGCAAAA---------CCCTGCT---GGCATCA--ATCACTTCCT--------TGCACTG-CCAGCACCAACGTCACAAACATCAAACCCCATCAACCTCTACTTGCGCAACA-AACTGCTCG--------GACAATCATGCTGACTTAC----TTCTCACACAGCTGCCGAGCTGGGTAAGGG

>Diaporthe_acericola_MFLUCC_17_0956

NNNNNNNNNNNNNNNNNNNNNNNNNNNNNNNNNNNNNNNNNNNNNNNGGAT--GACT--CCA-C-CAAG---CCCACCGTGCGCAC-------ATCGTCATCATTG--GTGCG----CGAC-TGCCTGCGCGGCTTCAT--CACCC----CTCAAACCA-TTTTCACCCCTCCCTCTGGGTTTTTTTTTCATT---TTCAGTGC-------------------GGGTGCGGGGCGCGCTTATCA-G----CCGCTTATCTCCT--A--TGCAAAA---------CCCTGCT---GGCATTA--ACCACTCCTT--------GCCACCG-CCAACACCAACTCTACCACTTTCAACCCTATCAATCTCCCAGGCGAAACG---AACTGTCAA--------GAATATGAAGCTGACTTAC----TTTCCACACAGCCGCCGAGCTGGGTAAGGG

>Diaporthe_cichorii_MFLUCC_17_1023

NNNNNNNNNNNNNNNNNNNNNNNNNNNNNNNNNNNNNNNNNNNNNNGTATC--AACC--CCA-C-AAAG---TCCTCCATGCGCAC-------ATTGTCCTGATTG--GTGCG----CGAC-TGCCTGCGCGGCTTCGTCGCACCC----GTCAAGCCA-TTTTCACCCCTCCCTCTGGG--TTTTTTCCATT---TTCAGTGC-------------------GGGTGCGGGGTGCGCTTATCAGG----CCGCTTATCTCCAACA--CGCAAAA---------CCCTGCT---GGCATGA--ACCACTCCCT--------ACCACCG-TCAACACCAACTCTACCACTTTGAACCCTATCAAACACCCTCCTGCCGAAAAGATCTGTCAA--------GAACATGACGCTGACTTAC----TTTCCACACAGCTGCCGAGCTGGGTAAGGG

>Diaporthe_guttulata_CGMCC_3_20100

NNNNNNNNNNNNNNNNNNNNNNNNNNNNNNNNNNNNNNNNNTCACAGCATC--AACT--CCA-C-AAAG---CCCTCCATGCGCAC-------ATTGTCATCATTG--GTGCG----CGAC-TGCCTGCGCGGCTTCGTCACACCC----GTCAAGCCA-TTTTCACCCCTCCCTCTGGG--TTTTTTCCATT---TTCAGTGC-------------------GGGTGCGGGGTGCGCTTATCAGG----CCGCTTATCTCCCACA--CGCAAAA---------CCCTGCT---GGCATTA--ACCACTCCCT--------ACCACCG-TCAACACCGACTCTACCACTTTGAACCCTATCAAACACCCTCCTGCCGAAAAGAACTGTCAA--------GAACATGACGCTGACTTGA----TTTCCATACAGCCGCCGAGCTTGGTNNNNN

>Diaporthe_schoeni_MFLU_15_1279

NNNNNNNNNNNNNNNNNNNNNNNNNNNNNNNNNNNNNNNNNNNNNNNNNNNNNNNNNNNNNNNNNNNNNNNNNNNNNNNNNNNNACATGCAGAATTGTCATCATTG--GTGCG----CGAC-TGCCTGCGCGGCTTCGTCACACCC----GTCAAGCCA-TTTTCACCCCTCCCTCTGGG--TTTTTTCCATT---TTCAGTGC-------------------GGGTGCGGGGTGCGCTTATCAGG----CCGCTTATCTCCCACA--CGCAAAA---------CCCTGCT---GGCATCA--ACTACTCCCT--------ACCACCG-TCAACACCAACTCTACCACTTTGAACCCTATCAAACACCCTCCTGCCGAAAAGAACTGTCAA--------GAACATGACGCTGACTTAA----TTTCCATACAGCCGCCGAGCTGGGTAAGGG

>Diaporthe_chinensis_MFLUCC_19_0101

NNNNNNNNNNNNNNNNNNNNNNNNNNNNNNNNNNNNNNNNNNNNNNNNNNNNNNNNNNNNNC-T-TG-----CCCTCCATGTGCAT-------CTCAGAATCATTG--GCGCG----TGGC-CATCCGCGAGGCTTCGTCACATCC----GTCAAGGCA-TTTTCACCCCTCGCTCTGGA----TTTTCCATT---TTCAGTGC-------------------GGGTGCGGGGTGTGCTTATCAGG----CGGCTTATCTCTTACA--TCTACAA---------CCCTGTT----GCATCA--ACCACTCCAT--------CCCGCTG-CCTCCTCCAACACCATCACTATCATCTTTCAGCTAAAAAAAA--AACCTGATGAAATGCGCC--------GGAATTCATGCTGACCATT-GTTTTTAAAAACAGCCGCCGAGCTTGGTAANNN

>Diaporthe_sclerotioides_CBS_296_67

NNNNNNNNNNNNNNNNNNNNNNNNNNNNNNNNNNNNNNNNTCCATGCCACA--CACT--CCA-T-TG-----CCCTCCATGTGCAG-------CTCAGAGTCATCG--TCGCG----CGGC-CGTTTGCCCGGCTGTGTCCCACCC----GTCAAGGCA-TTTTCACCCCTCCCTCTGGA----TTTTCCATT---TCCAGTGC-------------------GGGTGCGGGGT--GCTTATCAGG----CGGCTTATCTCTCGCA--TCCAAAA---------CCCTGTT----GCACCA--ACCACTCCCTCCCTTGCTGCTGCTG-CCACCACC--------------------------------------------------------------GGAGATCAAGCTGACATTT----CTTGTACACAGCCGCCGAGCTGGGCAAGGG

>Diaporthe_mayteni_CBS_133185

NNNNNNNNNNNNNNNNNNNNNNNNNNNNNNNNNNNNNNNNNNNNNNNNNNNNNNNNNNNNNNNNNNNNNNNNNNNNNNNNNNNNNNNNNNNNNNNNNNNNNNNNNNNNNNNNNNNNNCGGC-CATCTGTGCGGCTTCGTCTCTCCC----G-CAAGGCA-TTTTCACCCCTCCCTCTGGA----TTTTCCATT---TTCAGTGC-------------------GGGTGCGGGGTGCGCTTATCAGG----CCGCTTATCTCTCACA--CCCAAAA---------CCCTGGT----GCATCA--ACCACTCCTT--------ACCGCAG-CTACCATC--------------------------------------------------------------GGAAATCATGCTGANNNNNNNNNNNNNNNNNNNNNNNNNNNNNNNNNNNNNNN

>Diaporthe_sinensis_ZJUP0033_4

GAGAAGGAAGGTTAGTAAACAA--CACA-----------GCCCCAAGCATG--AACC--CTA-C-AAAG---TCCTCCAGTCGCAC-------ATCGTCATTATTG--GTGCG----CGAC-TGCCCGCGCGGCTTTGTTAGACCC----GTCAGGCCA-TTTTCACCCCTCCCTCTGGT----TTTTCCCCCTCGTTCAGTGC-------------------GGGTGCGGGGTGCGCTTATCAAG----CCACTTATCTCCCACA--CGCAATA---------CCGTGCT---GGCAAGG--GCGGCTCCTC--------ACCACTG-TCAACACTCTCCCCGCCACCACCATCAACCCAATCCATCTCCCTGGCGAAACGAGCTTTCAG--------GATAATCAAGCTGACTTAC----TTTTCACATAGCCGCCGAGCTGGGTAAGGG

>Diaporthe_compacta_LC3083

GAGAAGGAAGGTCAGTAAACAT--AACAAA---------CTCCACAACATC--AACC--CCAA--AGTC---ATCCCAGTGCACAT-------CGCGGGATCATCG--GCGCG----CGAC-CGTCTGCGCCGCTTCGT--CACTC----GTCAGAGCA-TTTTCACCCCTCGTTCTGGA----TTTTCCACT---TTCAGTGC-------------------GGGTGCGGGGTGTGCTTATCAGG----CCGCTTATCGCTTGCG-TTCCAAAA---------CCCTGCTGGGGGCATCA--ACCACTCTTT--------GCCGCTG-CCCACATT--------------------------------------------------------------GAAAACAATGCTGACTCGC----TTTCCATACAGCCGCCGNNNNNNNNNNNNN

>Diaporthe_ganjae_CBS_180_91

NNNNNNNNNNNNNNNNNNNNNNNNNNNNAA---------CTCCACAACATC--AACC--CCAA--AGTC---ATCCCAGTGCACAT-------CGCGCGATCATCG--GCGCG----CGAC-CGTCTGCGCCGCTTCGT--CACTC----GTCACAGCA-TTTTCACCCCTCGTCCTGGA----TTTTCCACT---TTCAGTGC-------------------GGGTGCGGGGTGTGCTTATCAGG----CCGCTTATCGCTTGCG-TTCCAAAA---------CCCTGCCTGGGGCACCA--ACCACTCTTC--------GCCGCTG-CCCACATT--------------------------------------------------------------GAAAACCATGCTGACTCGC----TTTCCATACAGCCGCCGAGCTGGGTAAGGG

>Diaporthe_manihotia_CBS_505_76

GAGAAGGAAGGTCAGTAAACAC--TACAAA---------CTCCACAACACC--AACC--CCAA--AGTC---ACCCCAGTGCACCT-------CGCGCGACCATTGGCGCGCG----CGAC-AGCCTGCGCGGCTTCGT--CACTC----GTCACAGCA-TTTTCACCCCTCGTTCTGGA----TTTTCCACT---TTCAGTGC-------------------GGGTGCGGGGTGTGCTTATCAGG----CCGCTTATCGCGTGCC-TTCCAAAA---------CCCTGCTGCGGGCATCA--ACCACTCTCT--------GCCGCTG-CCCACCAC--------------------------------------------------------------GAAAACCATGCTGACTCGC----TTTCCATACAGCCGCCGAGCTGGGTAAGGG

>Diaporthe_sambucusii_CFCC_51986

NNNNNGGAAGGTCAGTAAACAT--TACAAA---------CTCCACAACATC--AACC--CCAA--AGTC---ATTCCAGTGCACAT-------CGCGCGATCATCG--GCGCG----CGAC-CGTCTGCGCCGCTTCGT--CACTC----GTCACAGCA-TTTTCACCCCTCGTTCTGGA----TTTTCCACT---TTCAGTGC-------------------GGGTGCGGGGTGTGCTTATCAGG----CCGCTTATCGCTTGCG-TTCCAAAA---------CCCTGCTGGGGGCATCA--ACCACTCTTT--------GCCGCTG-CCCACATTAACGCCAACGCCATCAATCTCATCACCCCACCCCCTCTGACAAGACAAACCCTGAG------GAAAACCATGCTGACTCGC----TTTCCATACAGCCGCCGAGCTGGGTAANNN

>Diaporthe_alangii_CFCC_52556

GAGAAGGAAGGTGAGTAAACAT--CAAAA----------GTCTACGGCATA--CACT--CCCAT-TG-----TACCCCAGTTGCAC-------ATCAGGATTACTG--GCGCG----CTGC-CGTTTGCGCGGCTTCGTCACACCC----GCCAGGGCA-TTTTCACCCCTCCCTCTGGA----TTTTCCATT---TTCAGTGC-------------------GGGTGCGGGGTGCGCTTATCAGG----CGGCTTATCTCTGACG-CTCACAAA---------CCCTGCT----GCATCA--ACCATTCCTT--------GTCGCTG-CCACCACCACCATCACCGTCAAATTCATCGCACGGTCAAATTGATCGGATGACTTGTGTCTTGGAGA---AATCATGAAGCTGACTTTC----CATCTCTATAGCCGCCGAGCTCGGTAAGGG

>Diaporthe_hubeiensis_JZB320123

GAGAAGGAAGGTTA-------------------------GTCTACGGCATA--CACT--CCCAT-TG-----TACCCCAGTTGCAC-------ATCAGGATTACTG--GCGCG----CTGC-CGTTTGCGCGGCTTCGTCACACCC----GCCAGGGCA-TTTTCACCCCTCCCTCTGGA----TTTTCCATT---TTCAGTGC-------------------GGGTGCGGGGTGCGCTTATCAGG----CGGCTTATCTCTGACG-CTCACAAA---------CCCTGCT----GCATCA--ACCATTCCTT--------GTCGCTG-CCACCACCACCATCACCGTCAAATTCATCGCACGGTCAAATTGATCGGATGACTTGTGTCTTGGAGA---AATCATGAAGCTGACTTTC----CATCTCTATAGCCGCCGAGCTCGGTAAGGG

>Diaporthe_celtidis_NCYU_19_0357

GAGAAGGAAGGTTAGTAAACAT--CAAAA----------GTCTACGGCACA--CACT--CCCAT-TG-----TACCCCATTTGCAC-------ATCAGGATTACTG--GCGCG----CTGC-CGTTTGCGCGGCTTCGTCACACCC----GCCAGGGCA-TTTTCACCCCTCCCTTTGGA----TTTTCCATT---TTCAGTGC-------------------GGGTGCGGGGTGCGCTTATCAGG----CGGCTTA--TCTGACG-CTCACAAA---------CCCTGCT----GCATCA--ACCATTCCTT--------GTCGCTG-CCACCACCACCATCACCGTCAAATTCATCGCACTGTCAAATTGATCGGATGACTTGTGTCTTGGAGG---AATCATGAAGCTGACTTTT----CATCTCTATAGCCGCCGAGCTCGGTAAGGG

>Diaporthe_tectonae_MFLUCC_12_0777

GAGAAGGAAGGTTAGTAAACAT--CAAAA----------GTCTACGGCATA--CACT--CCCAT-TG-----TACCCCAGTTGCAC-------ATCAGGATTACTG--GCGCG----CTGC-CGTTTGCGCGGCTTCGTCACAGCC----GCCAGGGCA-TTTTCACCCCTCCCTCTGGA----TTTTCCATT---TTCAGTGC-------------------GGGTGCGGGGTGCGCTTATCAGG----CGGCTTATCTCTGACG-CTCACAAA---------CCCTGCT----GCATCA--ACCATTCTTT--------GTCGCTG-CCACCACC--------------------------------------------------------------AATCATGAAGCTGACNNNNNNNNNNNNNNNNNNNNNNNNNNNNNNNNNNNNNN

>Diaporthe_tulliensis_BRIP_62248a

G-GAAGGAAGGTTAGTAAACAT--CAAAA----------GTCTACGGCACA--CACT--CCCAT-TG-----TACCCCATTTGCAC-------ATCAGGATTACTG--GCGCG----CTGC-CGTTTGGGCGGCTTCGTCACACCC----GCCAGGGCA-TTTTCACCCCTCCCTTTGGA----TTTTCCATT---TTCAGTGC-------------------GGGTGCGGGGTGCGCTTATCAGG----CGGCTTA--TCTGACG-CTCACAAA---------CCCTGCT----GCATCA--ACCATTCCTT--------GTCGCTG-CCACCACC--------------------------------------------------------------AATCATGAAGCTGACTTTC----CATCTCTACAGCCGCCGAGCTCGGCAAGGG

>Diaporthe_ambigua_CBS_114015

GAGAAGGAAGGTCAGTAAACAT--CGAAT----------GCCTACGGCATA--CACC--CCCAT-TG-----CCTTTCATATGCAC-------TTCAGAATCACTT--GTGCG----CGGC-CGTTTGCGCGGCTTCGCCACACCC----TTCAAGGCATTTTTCACCCCTCCCTCTGGA----TTTTCCATT---TTCAGTGC-------------------GGGTGCGGGGTGTGCTTATCAGG----CGGCTTATCTCTTACA-CTCACAAACC-------CCTTGTT----GCATCA-ACCCACTCGGT--------GCTGCTG-TTATCACC--------------------------------------------------------------GAACATCATGCTGACCTCTT---TTCCCACACAGCCGCCGAGCTGGGTAAGGG

>Diaporthe_biguttulata_ICMP20657

GAGAAGGAAGGTTAGTAAACAT--CATGA----------GTACACAGCATA--AACT--CCC-T-TG------CGTCCGTATGCAC-------CTCCGAATTATCG--GCGCG----CGGC-CATCTGCGCGGCTTCGTCACATCT----GTGAAGGCA-TTTTCACCCCTCGCTCTGGA----TTTTCCATT---TTCAGTGC-------------------GGGTGCGGGGTGCGCTTATCAGG----CGGCTTATCTCTTACA--CCCAAAA---------CCCTGTT----GCATCC--ACCACTCCAT--------GCCGC-G-CTACCACC--------------------------------------------------------------GGAACATATGCTGACCATC----TTTCTACACAGCCGCCGAGCTTGGTAAGGG

>Diaporthe_cinnamomi_CFCC_52569

GAGAAGGAAGGTTAGTAAACAT--CATGA----------GCTCGCAGCATA--CACT--CTC-T-TG-----CCCCCCATATGCAT-------CTCGAAGTCATTG--GCGCG----CGAC-CATCTGCGCGGCTTCGTCACATCC----GTAAAGGCA-TTTTCACCCCTCGCTCTGGA---TTTTTCCATT---TTCAGTGC-------------------GGGTGCGGGGTGCGCTTATCAGG----CGGCTTATCTCTTACA-TCACAAAA---------CCCTGTT----GCATCA--CCCACTCCAT--------CCCGCTG-CTAGTACCAACACCATCACTATCATCTTTGAGCTGGAAACAG--AACCTGATGAATTGCGCT--------GGAATCTATGCTGACCATT----TTCCTACACAGCCGCCGAGCTTGGTAAGGG

>Diaporthe_citriasiana_CBS_134240

GAGAAGGAAGGTTAGTAAACAT--CATGA----------GTTCGCAGCATA--CACT--TCC-T-TG-----CCCTCCATATGCAT-------CTCAGAATCATTG--GCGCG----CGGC-CATCTGCGCGGCTTCGTCACATCC----GTCAAGGCA-TTTTCACCCCTCGCTCTGGA----TTTTCCATT---TTCAGTGC-------------------GGGTGCGGGGTGCGCTTATCAGG----CGGCTTATCTCTTACA--TCCACAA---------CCCTGTT----GCATCA--ACCACTCCAT--------CCCGCTG-CCACCTCC--------------------------------------------------------------GGAATTTATGCTGACCATT----TTTCTACACAGCCGCCGAGCTTGGTAAGGG

>Diaporthe_siamensis_MFLUCC_10_0573a

GAGAAGGAAGGTTAGTAAATAT--TACGA----------GTCCGTTGCATA--CACT--CTC-T-CG-----CCCTCCATATGCAT-------CTCAGAATCATTG--GCGCG----TGGC-CATCCGCGCGGCCTCGTCACATCT----GTCAAGGCA-TTTTCACCCCTCGCTCTGGA----TTTTCCATT---TTCAGTGC-------------------GGGTGCGGGGTGTGCTTATCAGG----CGGCTTATCTCTCACA--TCTATAA---------CCCTGCT----GCATCA--ACCACTCCAT--------CCCGCTG-CCACCACC--------------------------------------------------------------GGAATATATGCTGACCATTAGTGTTTGAAAACAGCCGCCAAGCTTGGTAAGGG

>Diaporthe_yunnanensis_CGMCC_3_18289

GAGAAGGAAGGTTAGTAAACAT--CATGA----------GTTCGCTGCATA--CACT--CCC-T-TG-----CCCTCCATGTGCAT-------CTCAGAATCATTG--GCGCG----TGGC-CATCCGCGAGGCTTCGTCACATCC----GTCAAGGCA-TTTTCACCCCTCGCTCTGGA----TTTTCCATT---TTCAGTGC-------------------GGGTGCGGGGTGTGCTTATCAGG----CGGCTTATCTCTTACA--TCTACAA---------CCCTGTT----GCATTA--ACCACTCCAT--------CCCGCTG-CCTCCACCAACACC--------------------------------------------------------GGAATTCATGCTGACCATT-GTTTTTGAAAACAGCCGCCGAGCTTGGTAAGGG

>Diaporthe_fici_septicae_MFLU_18_2588

GAGAAGGAAGGTTAGTAAACAT--CATGA----------GTTCGCAGCATA--CACT--TCC-T-TG-----CCCTCCACATGCAT-------CGCAGAATCATTG--GCGCG----CGGC-CATCTGCGCGGCTTCGTCACATCC----GTCAAGGCA-TTTTCACCCCTCGCTCTGGA----TTTTCCATT---TTCAGTGC-------------------GGGTGCGGGGTACGCTTATCAGG----CCGCTTATCTCTTGCA--TCCACAA---------CCCTGTT----GCATCA--CCCACTCCAT--------CCCGCCG-CCACCACCAACACCATCACTACCATCTTTGAACTGAAAAAGAACAACCTGATGAATTGCGCT--------GGAATCTATGCTGACCATT----TTTCTACACAGCCGCCGAGCTTGGTAAGGG

>Diaporthe_discoidispora_ICMP20662

GAGAAGGAAGGTTAGTAAACAT--CATGA----------GCTCGCAGCATA--CAAT--CTC-T-TG-----CCCTCCATATGCA--------CTCAGAATCATCG--GCGCG----CTAC-ATTCTGCGCGGCTTCGTAACATCC----GTAAAGGCA-TTTTCACCCCTCGCTCTGGA----TTTTCCATT---TTCAGTGC-------------------GGGTGCGGGGTGCGCTTATCAGG----CGGCTTATCTCTTACA-TCACAAAA---------CCCTCTT----GCATCA--ACCACTCCAT--------CCCGTTG-CTACTACC--------------------------------------------------------------GGAACCTAAGCTGACCTTTTTTTCTTCTACACAGCCGCCGAGCTTGGTAAGGG

>Diaporthe_longispora_CBS_194_36

GAGAAGGAAGGTCAGTAAACAC--CAAAAA---------GTCCATGCCACA--CACC--TCA-T-TG-----CCCTCCATGTGCAC-------CTCGGAATCATTG--GCGCG----CGGC-CGTTTGCGCGGCTTCGTCCCACCC----CTCAAGGCA-TTTTCACCCCTCCCTCTGGA----TTTTCCATT---TCTAGTGC-------------------GGGTGCGGGGT--GCTTATCAGG----CGGCTTATCTCTCGCA--CCCAAAA---------CCCTGTT----GCATCA--ACCACTCCTT--------ACTGCTGCCCACCACC--------------------------------------------------------------GGAAATGAAGCTGACATTT----TTTCTACACAGCCGCCGAGCTGGGTAAGGG

>Diaporthe_neoraonikayaporum_MFLUCC_14_1136

GAGAAGGAAGGTTAGTAAATAT--CAAAA----------ACTCCCAGCATA--CACT--GCA-T-TA-----TCCTCCATGTGCTA-------CTTAGAATCATCG--CTGCG----CGGC-CACCTGCGCGGC-TCATCACACCC----GTCAACGCA-TTTTCACCCCTCCCTCTGGA----TTTTCCATT---TTCAGTGC-------------------GGGTGCGGGGTGTGCTTATCTGG----CGGCTTATCTCCCACACCTCAAAAA---------CCCTGTC----GCATCA--ACCACCACCT--------GCCACCA-CCATCAAC--------------------------------------------------------------AGAAATCGTGCTGACNNNNNNNNNNNNNNNNNNNNNNNNNNNNNNNNNNNNNN

>Diaporthe_raonikayaporum_CBS_133182

GAGAAGGAAGGTTAGTAAATAT--CAAA-----------ACTCCCAGCATA--CACT--GCA-T-TA-----TCCTCCATGTGCTA-------CTCAGAATCATCG--CTGCG----CGGC-CACCTGCGCGGC-TCATCACACCC----GTCAATGCATTTTTCACCCCTCCCTCTGGA----TTTTCCATT---TTCAGTGC-------------------GGGTGCGGGGTGTGCTTATCTGG----CGGCTTATCTCCCACA-CTCAAAAA---------CCCTGTT----GCATCA--ACCACTCCCTAC----CTGCCACCA-CCATCAAC--------------------------------------------------------------AGAAATCGTGCTGACCTCTT---TGTCTGCGTAGCCGCCGAGCTCGGTAAGGG

>Diaporthe_cerradensis_CMRP4331

NNNNNNNNNNNNNNNNNNNNNNNNNNNNNNNNNNNNNNNNNNNACAGCTTA--CACT--C------------TGCCCTCTATCCCT-------CAGAGAATCGTTG--GCGCA----CGGC-CGTCTGCGCGGCTTCGT--CACCC----GTGAAGGCA-TTTTCACCCCTCCCTCTGGA----TTTTCCATT---TTCAGTGC-------------------GGGTGCGGGGTGCGCTTATCAGG----CCGCTTATCTCTGACC--TTTACAA---------CCCTGGC----GCATCA--ACCACTCATT--------ACCGCTG-CTACCACCAACAACATCACTGCCGATTCCACTGACGTCTCCCGCCAAAACAACCTGATGAGCCCTGCTGGAAAATTCACGCTGACCTTT----TTTCTACACAGCCGCCGAGCTGGGTAAGGG

>Diaporthe_batatas_CBS_122_21

?????????????????????????CATCA---------GAACCCAGCTTG--ACCA--CTA-C-GT-----CCCTCCACATGCAC-------TCAGAAATCCTTG--GCCCG----CG-C-TGTCTGTGACGCTTCGTCACACCT----GTGAAAGCA-TTTTCACCCCTCCCTCTGGA----TTTTCCATT---TTCAGTGC-------------------GGGTGCGGGGTGCGCTTATCAGG----CCGCTTATCTCCCA---CACCAAAA---------CCCTGTC----GCACCC--ACCACTCTTTG-------ACCAGTG-ACATTCC---------------------------------------------------------------GGAAACCATGCTGACTC------TCTCTACACAGCCGCCGAGCTGGGTAAGGG

>Diaporthe_convolvuli_CBS_124654

GAGAAGGAAGGTTAGTAAACAT---ACTAA---------ATGGGTGGCCTG--ACCT--CCA-T-AT-----CCCTCCACATGAAC---------GGAAATCGTTG--GCGCG----CG-C-TGTCTGCGAGGCTTCGTCACACCT----GTCAAGCCA-TTTTCACCCCTCCCTCTGGA----TTTTCCATT---TTCAGTGC-------------------GGGTGCGGGGTGCGCTTATCAGG----CCGCTTATCTCTCACA-CACCAAAG---------CCCTGTC----GCACCTCATCACTCTTTT--------ACCAGCT-ACACACC---------------------------------------------------------------GAAAATCATGCTGACTCTG----TTTCTACACAGCCGCCGAGCTGGGTAAGGG

>Diaporthe_endophytica_CBS_133811

GAGAAGGAAGGTTAGTAAACAT--CGCCCA---------ACGGGCACCTTG--ACCT--CCA-C-AT-----CACCCCATACGCCC---------AGAACTCGTTG--GCGCG----CG-C-TGTCTACGAGGCTTCGTCACACCT----CTCAAGGCA-TTTTCACCCCTCCCTCTGGA----TTTTCCATT---TTCAGTGC-------------------GGGTGCGGGGTGCGCTTATCAGG----CCGCTTATCTTTCA---CACCAAAA---------CCCTGTC----GCACCTTACCTCTCTTTC--------ACCAGTG-ACGCACC---------------------------------------------------------------GAAAATGATGCTGACTC------TTTCTGAACAGCCGCCGAGCTGGGTAAGGG

>Diaporthe_kongii_BRIP_54031

???AAGGAAGGTTAGTAAACAT--CGCCCA---------ACGGGCACCTTG--ACCT--CCA-C-AT-----CACCCCATACGCCC---------AGAACTCGTTG--GCGCG----CG-C-TGTCTGCGAGGCTTCGTCACACCT----GTCAAGGCA-TTTTCACCCCTCCCTCTGGA----TTTTCCATT---TTCAGTGC-------------------GGGTGCGGGGTGCGCTTATCAGG----CCGCTTATCTTTCA---CACCAAAA---------CCCTGTC----GCACCTTACCTCTCTTTC--------ACCAGTG-ACGCACC---------------------------------------------------------------GAAAATGATGCTGACTC------TTTCTGAACAGCCGCCGAGCTGGGCAAGGG

>Diaporthe_masirevicii_BRIP_57892a

????AGGAAGGTTAGTAAACAT--CGCCCA---------ACGGGCACCTTG--ACCT--CCA-CAAT-----CACCCCATACGCCC---------AGAACTCGTTG--GCGCG----CG-C-TGTCTGCGAGGCTTCGTCACACCT----GTCAAGGCA-TTTTCACCCCTCCCTCTGGA----TTTTCCATT---TTCAGTGC-------------------GGGTGCGGGGTGCGCTTATCAGG----CCGCTTATCTTTCA---CACCAAAA---------CCCTGTC----GCACCTTACCTCTCTTTC--------ACCAGTG-ACGCACC---------------------------------------------------------------GAAAATGATGCTGACTC------TTTCTGAACAGCCGCCGAGCTGGGCAAGGG

>Diaporthe_kochmanii_BRIP_54033

??GAAGGAAGGTTAGTAAACAT--CGCCCA---------ACGGGCACCTTG--ACCT--CCA-C-AT-----CACCCCATACGCAC---------AGAACTCGTTG--GCGCG----CG-C-TGCCTGCGAGGCTTCGTCACACCT----GTCAAGGCA-TTTTCACCCCTCCCTCTGGA----TTTTCCATT---TTCAGTGC-------------------GGGTGCGGGGTGCGCTTATCAGG----CCGCTTATCTCTCA---CACCAAAA---------CCCTGTC----GCACCT-ACCTCTCTTCC--------ACCAGTG-ACGCACC---------------------------------------------------------------GAAAATGATGCTGACTC------TTTCTGAACAGCCGCCGAGCTGGGCAAGGG

>Diaporthe_phaseolorum_CBS_113425

GAGAAGGAAGGTTAGTAAACAT--CGCCCA---------ACGGGCACCTTG--ACCT--CCA-C-AT-----CACCCCATACGCAC---------AGAACTCGTTG--GCGCG----CG-C-TGCCTGCGAGGCTTCGTCACACCT----GTCAAGGCA-TTTTCACCCCTCCCTCTGGA----TTTTCCATT---TTCAGTGC-------------------GGGTGCGGGGTGCGCTTATCAGG----CCGCTTATCTCTCA---CACCAAAA---------CCCTGTC----GCACCT-ACCTCTCTTCC--------ACCAGTG-ACGCACC---------------------------------------------------------------GAAAATGATGCTGACTC------TTTCTGAACAGCCGCCGAGCTGGGTAAGGG

>Diaporthe_sojae_CBS_139282

NAGAAGGAAGGTTAGTAAACAT--CGCCCA---------ACGGGCACCTTG--ACCT--CCA-C-AT-----CACCCCATACGCAC---------AGAACTCGTTG--GCGCG----CG-C-TGCCTGCGAGGCTTCGTCACACCT----GTCAAGGCA-TTTTCACCCCTACCTCTGGA----TTTTCCATT---TTCAGTGC-------------------GGGTGCGGGGTGCGCTTATCAAG----CCGCTTATCTCTCA---CACCAAAA---------CCCTGTC----GCACCTTAC--CTCTTCC--------ACCAGTG-ACGCACC---------------------------------------------------------------GAAAATGATGCTGACTC------TTTCTGAACAGCCGCCGAGCTGGGTAAGGG

>Diaporthe_fructicola_MAFF_246408

GAGAAGGAAGGTTAGTAAACAT--CGCCCA---------ACGGGCACCTTG--ACCT--CCA-C-AT-----CACCCCATACGCCC---------AGAACTCGTTG--GCGCG----CG-C-TGTATGCGAGGCTTCGTCACACCT----GTCAAGGCA-TTTTCACCCCTCCCTCTGGA----TTTTCCATT---TTCAGTGC-------------------GGGTGCGGGGTGCGCTTATCAGG----CCGCTTATCTTTCA---CACCAAAA---------CCCTGTC----GCACCTTACCTCTCTTTC--------ACCAGTG-ACGCACCAACAGCATCACCTTCACTCCCA--TCTTTTGCTTGAGGGAGCTTTTCAACTCGCTC-------GAAAATGATGCTGACTC------TTTCTGAACAGCCGCCGAGCTGGGTAAGGG

>Diaporthe_subellipicola_KUMCC_17_0153

??GAAGGAAGGTTAGTAAACAT--CGCCCA---------ACGGGCACCTTG--ACCT--CCA-C-AT-----CACCCCATACGCCC---------AGAACTCGTTG--GCGCG----CG-C-TGTCTACGAGGCTTCGTCACACCT----GTCAAGGCA-TTTTCACCCCTCCCTCTGGA----TTTTCCATT---TTCAGTGC-------------------GGGTGCGGGGTGCGCTTATCAGG----CCGCTTATCTTTCA---CACCAAAA---------CCCTGTC----GCACCTTACCTCTCTTTC--------ACCAGTG-ACGCACCAACAGCATCACCTTCATTCCCA--TCTTTTGCTTGAGGGAGCTTTTCAACTCGCTC-------GAAAATGATGCTGACTC------TTTCTGAACAGCCGCCGAGCTGGGCAAGGG

>Diaporthe_heterostemmatis_SAUCC194_85

??????????????????GCGT--ACTCGC---------ACGGGCACCTTG--ACCT--CCA-C-AT-----CACCCCATACGCCC---------AGAACTCGTTG--GCGCG----CG-C-TGTCTACGAGGCTTCGTCACACCT----GTCAAGGCA-TTTTCACCCCTCCCTCTGGA----TTTTCCATT---TTCAGTGC-------------------GGGTGCGGGGTGCGCTTATCAGG----CCGCTTATCTTTCA---CACCAAAA---------CCCTGTC----GCACCTTACCTCTCTTTC--------ACCAGTG-ACGCACCAACAGCATCACCTTCATTCCCA--TCTTTTGCTTGAGGGAGCTTTTCAACTCGCTC-------GAAAATGATGCTGACTC------TTTCTGAACAGCCGCCGAGCTG????????

>Diaporthe_ovalispora_ICMP20659

GAGAAGGAAGGTTAGTAAACAT--CACCGA---------ACGGGCACCTTG--ACCT--CCA-C-AT-----CTCTCCATATCCAC---------AGAACTCGCTG--GCGCG----TG-A-TGTCTGCGAGGCTTCGTCACGCCT----GTCAGGGCA-TTTTCACCCCTCCCTCTGGA----TTTTCCATT---TTCAGTGC-------------------GGGTGCGGGGTGCGCTTATCAGG----CCGCTTATCTAT-----CACCAAAA---------CCCTGTC----GCACCTCACCACTCTTTC--------ACCAGTG-GCGTGCC---------------------------------------------------------------GAGAATCATGCTGACTC---------CTACACAGCCGCCGAGCTGGGTAAGGG

>Diaporthe_guangdongensis_ZHKUCC20_0014

GAGAAGGAAGGTTAGTAAACAT--CACCAA---------ACCCGCAGCTTG--ACCT--CCA-T-GC-----TCCTCCGTATGCAC-------ATGGAAATCGTTG--GCGCG----CG-C-TGTCTGCGAGGCTTCGTCACACCT----GTCAAGGCA-TTTTCACCCCTCCCTCCGGA----TTTTCCATT---TTCAGTGC-------------------GGGTGCGGGGTGCGCTTATCAGA----CCGCTTATCTCCTCG--CACCAAAA---------CCCTGCC----GCACCT--AGCACTCTTC--------ACCTGTG-ACGCACCAATATCATCGTTTTTATTCCCAATTTTGTTGCGCGAAGGAGCTACTCGAATCGCTT-------GGAAGTCATGCTGACTC------TTTCTGTACAGCCGCCGAGCTGGGTAAGGG

>Diaporthe_melonis_CBS_507_78

GAGAAGGAAGGTTAGTAAACAT--CACCAA---------ACCCGCAGTTTG--ACCT--CCA-T-GT-----CCCTCCATACGCAC-------ATAGAAATCGTTG--GCGCG----CG-C-TGCCTGCGGGGCTTCGTCACACCT----GTCCAGGCA-TTTTCACCCCTCCCTCCGGA----TTTTCCATT---TTCAGTGC-------------------GGGTGCGGGGTGTGCTTATCAGA----CCGCTTATCTCCTCG--CACCAAAA---------CCCTGCC----GCACCT--AGCACACTTC--------ACCTGTG-ACGCACC---------------------------------------------------------------GAAAGTCATGCTGACTC------TTTCTGTACAGCCGCCGAGCTGGGTAAGGG

>Diaporthe_vochysiae_LGMF1583

???????????????????????????????????????????????????--??CT--CCA-T-AT-----CCTTCCACATGCAC-------ATGGGAATCGTTG--CCGTG----CG-C-TGTCTGCGACGCTTCGTCACACCC----GTCAAGACA-TTTTCACCCCTCCCTCTGGA----TTTTCCATT---TTCAGTGC-------------------GGGTGCGGGGTGCGCTTATCAGG----CCGCTTATCTCACA---CATCAAA----------CCCTGTC----GCACCTCTGCCACTCTTC--------ACCAGTG-ACACACCAATAGCGTCAC-TTAATTCCCA--TTTGTTGCTCCAGGAAGCTTTTTGAATCGTTC-------GAAAATCATGCTGACTT------TTTCTACACAGCCGCCGAGCTGGGTAAGGG

>Diaporthe_thunbergiicola_MFLUCC_12-0033

GAGAAGGAAGGTTAGTAAATAT--CACTAC---------ATGTGCTGCTTG--CACT--CCA-T-AA-----CCCTCC-CATGCAC-------ATGGAAATCGTTG--CCGTG----CG-C-TGTCTGCGACGCTTCGTCACACCC----GTCAAGGTA-TTTTCCCCCCTCCCTCTGGA----TTTTCCTTT---TTCAGTGC-------------------GGGTGCGGGGTGTGCTTATCAGG----CCGCTTATCTCCCA---CATCAAA----------CCCCGTC----GCACCTCTGCCACTCCTG--------ACCAGGG-ACACACCAACAGCGTCCTTTTCATTCCCA--TTTGTTGCTCCATGAAGTTTTTTGAATCGTTC-------GAAAAACAAGCTGACTC------TTTCTACACAGCCGCCAAGCTGGGTTAGGG

>Diaporthe_longicolla_FAU_599

?AGAAGGAAGGTCAGTAAATAC--CACCAC---------ACGTGCAGCTTG--CGCT--CCA-T-AT-----CCCTCCATGTGCAC-------ATAGAAACCGTTG--GCGTG----CG-C-TGTCTGCGACGCTCCGTCACACCTACCTGTAAAGGCA-TTTTCACCCCTCCCTCTGGA----TTTTCCATT---TTCAGTGC-------------------GGGTGCGGGGTGCGCTTATCAGG----CCGCTTATCTCACA---CATCAA-----------CCCTGTC----GCACCTTTACCACTGTTC--------GCCAGTG-ACACACC---------------------------------------------------------------CAAAATCATGCTGACTC------TTTCTACACAGCCGCCGAGCTGGGTAAGGG

>Diaporthe_unshiuensis_CGMCC3_17569

GAGAAGGAAGGTTAGTAAATAC--CACCAC---------ACCTGCAGCTTG--CGCT--CCA-T-AT-----CCCTCTATATGCAC-------ATAGAAACCGTTG--GCGTG----CG-C-TGTCTGCGACGCTCCGTCACACCT----GTAAAGGCA-TTTTCACCCCTCCCTCTGGA----TTTTCCATT---TTCAGTGC-------------------GGGTGCGGGGTGCGCTTATCAGG----CCGCTTATCTCACA---CATCAA-----------CCCTGTC----GCACCTTTACCACTGTTC--------GCCAGTG-ACACACC---------------------------------------------------------------CAAAATCATGCTGACTC------TTTCTACACAGCCGCCGAGCTGGGTAAGGG

>Diaporthe_miriciae_BRIP_54736j

GAGAAGGAAGGTTAGTAAATAT--CACTAC---------ATGTGCTGCTTG--CGCT--CCA-T-AT-----CCTTCCACATGCAC-------ATGGGAATCGTTG--CCGTG----CG-C-TGTCTGCGACGCTTCGTCACACCC----GTCAAGGCA-TTTTCACCCCTCCCTCTGGA----TTTTCCATT---TTCAGTGC-------------------GGGTGCGGGGTGCGCTTATCAGG----CCGCTTATCTCACA---CATCAAA----------CCCTGTC----GCACCTCTGCCACCCTTC--------ACCAGTG-ACACACC---------------------------------------------------------------GAAAATCATGCTGACTT------TTTCTACACAGCCGCCGAGCTGGGCAAGGG

>Diaporthe_ueckerae_FAU_656

?AGAAGGAAGGTTAGTAAATAT--CACTAC---------ATGTGCTGCTTG--CGCT--CCA-T-AT-----CCTTCCACATGCAC-------ATGGGAATCGTTG--CCGTG----CG-C-TGTCTGCGACGCTTCGTCACACCC----GTCAAGGCA-TTTTCACCCCTCCCTCTGGA----TTTTCCATT---TTCAGTGC-------------------GGGTGCGGGGTGCGCTTATCAGG----CCGCTTATCTCACA---CATCAAA----------CCCTGTC----GCACCTCTGCCACCCTTC--------ACCAGTG-ACACACC---------------------------------------------------------------GAAAATCATGCTGACTT------TTTCTACACAGCCGCCGAGCTGGGTAAGGG

>Diaporthe_tectonendophytica_MFLUCC_13_0471

GAGAAGGAAGGTTAGTAAATAT--CACTAT---------ATGCGCAGCTTG--CGCT--CCA-C-AT-----CCCTCCATATGCAC-------ATAGAGATCGTTG--GTGTG----CG-C-TGTCTGCGACGCTTCGTCACACCT----GTCAAGGCA-TTTTCACCCCTCCCTCTGGA----TTTTCCATT---TTCAGTGC-------------------GGGTGCGGGGTGCGC-TATCAGG----CCGCTTATCTCACA---CATCAA-----------CCCTGTC----GCACCTTTATCACTGTTC--------ACCAGTG-ACATACC---------------------------------------------------------------GAAAATCATGCTGAC??????????????????????????????????????

>Diaporthe_infertilis_CBS_230_52

GAGAAGGAAGGTTAGTAAATAT--CACAAC---------ATGCGCAGTTTG--TCCA--CCA-T-AT-----TCCTCCATGTGCTC-------ACGAAACTCGTCG--GCGCA----CC-C-CGTCTGCGAGGCTTCGTCACACCT----GTCAAGGCA-TTTTCCCCCCTCCCTCTGGA----TTTTCCATT---TTCAGTGC-------------------GGGTGCGGGGTGCGCTTATCAGG----CCGCTTATCTCTCA---CACCAAAA---------CCCTGTC----GCACCT-TACCACTCCTT--------GCCAGTC-ATGCACA---------------------------------------------------------------GAAACTCATGCTGACTT------TTCCTACACAGCCGCTGAGCTGGGTAAGGG

>Diaporthe_hordei_CBS_481_92

GAGAAGGAAGGTTAGTAAACAT--CACCCA---------ATCTGCAGCTTG--AACT--CCA-T-AG-----CCCGCCACATGCCC-------ATCAAATTCG-----ACGCG----CGAC-TCTCTGCGAGGCTTCGTCATACCT----GTCAAGGCA-TTTTCACCCCTCCCTCTTGA----TTTTCCATT---TTCAGTGC-------------------GGGTGCGGGGTCCGCTTATCAGG----CCGCTTATCTCTTA---CACCAAAA---------CCCTGTC----GCACCT--ACCATTATTC--------GCCAGCG-CCACCCC---------------------------------------------------------------GAAAATCATGCTGACTC---------CTATACAGCCGCTGAGCTGGGTAAGGG

>Diaporthe_racemosae_CBS_143770

GAGAAGGAAGGTTAGTTAACAT--CACCCA---------ACCCGCAGCCTGAAAACT--TCA-T-AG-----CCTTC-ACATGCAC-------ATCAAAATCGTTG--GCGCG----CGAC-TGTCTGCGAGGCTTCGTCACAACT----GTCAAGGCA-TTTTCACCCCTCGCTCTGGA----TTTTCCATT---TTCAGTGC-------------------GGGTGCGGGGTGCGCTTATCAGG----CCGCTTATCTGCTC---CACCAAAA---------CCCTGTT----GCACCA--ACCGCTCTTT--------ACCACTG-CCACGCA---------------------------------------------------------------GGAAATCATGCTGACTT------GTTCTGTACAGCCGCCGAGCTGGGCAAGGG

>Diaporthe_schini_CBS_133181

GAGAAGGAAGGTCAGTAAACAT--CACCCA---------ACCCGCAGCCTG--AACA--TCA-T-AG-----CCTTC-ACATGCAC-------ATCAAAATCGTTG--GCGCG----CGAC-TGTCTGCGAGATTTCGTCACAACT----GTCAAGCCA-TTTTCACCCCTCGCTCTGGA----TTTTCCATT---TTCAGTGC-------------------GGGTGCGGGGTGCGCTTATCAGG----CCGCTTATCTCCTC---CACCAAAA---------CCCTGTT----GCACCA--ATCGCTCTTT--------ACCACTG-CCACGCA---------------------------------------------------------------GGAAATCATGCTGACTC------CTTCTGTACAGCCGCCGAGCTGGGTAAGGG

>Diaporthe_tecomae_CBS_100547

GAGAAGGAAGGTTAGT-AACAT--CACCCA---------ACCCGCAGCCCG--GGCT--CCA-T-AG-----CCCTCCGTATGCAC-------ATCAAATTCGTTG--GTGCG----CGAC-TGTCCGCAAGACTTGGTCACAACT----GTCAAGGCA-TTTTCACCCCTCGCTCTGGA----TTTTCCATT---TTCAGTGC-------------------GAGTGCGGGGTGCGCTTATCAGG----CCGCTTATCTCTTA---CACCAAAA---------CCCTTTT----GCACCA--ATCGCGCTTT--------ATCACTG-CCACGCA---------------------------------------------------------------GGAAATCATGCTGACTC------CTTCTGTACAGCCGCCGAGCTGGGTA????

>Diaporthe_terebinthifolii_CBS_133180

GAGAAGGAAGGTTAGTAAACAT--TACCCA---------ACCCGCAGCCTG--AGCC--CCA-T-AG-----CCCTCTGTATGCAC-------ATCAAAATCGTTG--GCGCG----CGAC-TGTCCGCAAGACCTGGTCACAACT----GTCAAGGCA-TTTTCACCCCTCGCTCTGGA----TTTTCCATT---TTCAGTGC-------------------GAGTGCGGGGTGCGCTTATCAGG----CCGCTTATCTCTTA---CACCAAAA---------CCCTGTT----GCACCA--ATCGCTCTTT--------ACCACTG-CCACGCA---------------------------------------------------------------GGAAATCATGCTGACTC------CTACTGTACAGCTGCCGAGCTGGGTAAGG?

>Diaporthe_rosiphthora_COAD_2913

GAGAAGGAAGGTCAGTAAACAT--CACCCA---------ACCCGCAGCCTG--AACA--TCA-T-AG-----CCTTC-ACATGCAC-------ATCAAAATCGTTG--GCGCG----CGAC-TGTCTG-GAGATTTCGTCACAACT----GTCAAGCCA-TTTTCACCCCTCGCTCTGGA----TTTTCCATT---TTCAGTGC-------------------GGGTGCGGGGTGCGCTTATCAGG----CCGCTTATCTCCTC---CACCAAAA---------CCCTGTT----GCACCA--ATCGCTCTTT--------ACCACTG-CCACGCAAACGTCGTTGCCATCATCCTCAAATCTCGCCAACAAACCTTCAATGTGGCTG-----------GGAAATCATGCTGACCC------CTTCTGTACAGCCGCCGAGCTGGGTAAGGG

>Diaporthe_megalospora_CBS_143_27

GAGAAGGAAGGTTAGTAAACATACCCCCCT---------CTCCGCAGCTTG--AACT--CCA-C-AG-----CCCTCTATATGCAC-------AAGGAAATCTTTG--GGCCG----CGAC-TGTCTGCGAGGCTTCGTAAGACCT----GCCATGGCA-TTTTCGCCCCGCCCTCTGGA----TTTTTCATT---TTCAGTGC-------------------GGGTGCGGGGTGCGCTTATCAGG----CCGCTTATCTCTTGAA-CACCAAAA---------CCCTGTT----GCACCT--ACCACTCTTC--------ACCACTG-CCACCCC---------------------------------------------------------------GAAAATCATGCTGACTC------TTTCTTCACAGCCGCCGAGCTGGGTAAGGG

>Diaporthe_vexans_CBS_127_14

GAGAAGGAAGGTTAGTAAACAT--CACCCA---------GCCTGCAGCTTG--AACCCACAG-T-CA-----CTCCATATATGCAC-------ATGAAAATCGTTT--G-GCG----CGAC-ACACTGCGAGGCTTCGTCATACCT----GTCAAGGCA-TTTTCACCCCTCTCTCTGGA----TTTTCCATT---TTCAGTGC-------------------GGGTGCGGGGTGTGCTTATCAGG----CCGCTTATCTCTCACC-AAACAAAA---------CCCCGTC----AAACCT--GCCACTCTTC--------ACCAGTG-CCACCCC---------------------------------------------------------------GAAAATCATGCTGACTT------TTTCTACACAGCCGCCGAGCTGGGTAAGGG

>Diaporthe_helianthi_CBS_592_81

GAGAAGGAAGGTTAGTAAACAT--TACCA----------GGCCGCACCTTG--AACCCATAA-CCCT-----CACTCCACATGCACATC----ATGATAATCGTTG--GCGCG----CCAC-TGCATGCGAGGCTTCGTCACACCT----GTCAAGGCA-TTTTCACCCCTCCCTCTGGA----TTTTCCATT---TTCAGTGCGGGTGCGGGTACGGGTGCGGGGTGCGGGGTGCGCTTATCAGG----CCGCTTATCTCTTACA---CCAAAA---------CCCTCCT-TATGCACCC--ACCGCACTCC--------TGCACCA-GTACAACCTT------------------------------------------------------------CAGAATCGGACTGACTC------TTTCTATATAGCCGCCGAGTTGGGTAAGGG

>Diaporthe_goulteri_BRIP_55657a

GAGAAGGAAGGTTAGTAAAAAC--AACACAA--------ATCCTCTGCATA--TACT--CCA----------CTCTTTCACAGTAC-------TATGGGAGTACTG--GTGCG----CGGC---CTGTGGCGGCTTCGTCACACCC----GTCAAGCCA-TTTTCACCCCTCCCTCTGGA----TTTTCCATT---TTCAGTGC-------------------GAGTGCGGGGTGTCCTTATCAGG----CGGCCTATCTCTTACACTCACAAAACTCGCAAACCCCTGCT----CCTTCA--ACCGCTGCCC--------AGAGCAA-ACACCACC--------------------------------------------------------------GAATATCATGCTGACA-------TGAGTATACAGCCGCCGAGCTGGGCAAGGG

>Diaporthe_amygdali_CBS_126679

GAGAAGGAAGGTTAGTAAACATCAACCTCTAC-------AATCGCAACACA--TTCT--GCA-T-GC-----CCCTTCACATGCTCGACT---TCATAAATCCTGG--GCGTG----CGCC-CGTTCCCACAGCGTCATCACACCT----GGGGGCGCA-TTTTCACCCCTCGCTCTGGA----TTTTCCATT---TTCAGTGC-------------------GGGTGCGGGGTGCGCTTATCAGC----GAGCTTATCTCCCACC---CGAAAA---------CCCTGCA--------------CACACTAC--------ATCACTACATACCACC--------------------------------------------------------------TTGGATCATGCTGACTTCC----CATCTTCACAGCCGCTGAGCTCGGTAAGGG

>Diaporthe_eres_CBS_138594

GAGAAGGAAGGTTAGTAAATAT--CAC------------AGTCACGGAACA--TGCT--ACC-T-GG-----CCCTCCATAC-TGC-------ACCTCAATCATCA--GCCCG----CAGC-TGCTCGCGCGGCCTCGCCATGTCG----GGGGGCGCA-TTTTCACCCCTCGCTTTGGA----TTTTCAATT---TTCAGTGC-------------------GAGTGCGGGGTGCGCTTATCAGGGGGCGGGCTTATCTCCTACA--ACCAAAA---------CCCTGTT----ACATCA--CTCACTCAATC-------CTTGTCA-CCACCACC--------------------------------------------------------------GAATATTATGCTGACCCTC----TATCTACACAGCCGCCGAGCTTGGTAAGGG

>Diaporthe_durionigena_VTCC_930005

????????????????????????????????????????TGTGCTGCTTG--CGCT--CCA-T-AT-----CCCTCCACATGCAC-------ATGGGAATCGTTG--CCGTG----CG-C-TGTCTGCGACGCTTCGTCACACCC----GTCAAGGCA-TTTTCACCCCTCCCTCTGGA----TTTTCCATT---TTCAGTGC-------------------GGGTGCGGGGTGCGCTTATCAGG----CCGCTTATCTCACA---CATCAAA----------CCCTGTC----GCACCTCTGCCACTCTTG--------ACCAGTG-ACACACCAACAGCGTCACTTTCATTCCCA--TTTGTTGCTCCAGGAAGCTTTTTGAATCGTTC-------GAAAATCATGCTGACTC------TTTCTACACAGCCGCCGAGCTGGGTAAGGG

>Diaporthe_breyniae

GAGAAGGAAGGTTAGTAAACAT--CACTGC---------ATGTGCAGCTTG--CGCT--CCG-T-AT-----CCCTCCATATGCAC-------ATGGGAGTCGTTG--CCGTG----CG-C-TGTCTGCGACGCTTCGTCACACCT----GTCAAGGCA-TTTTCACCCCTCCCTCTGGA----TTTTCCATT---TTCAGTGC-------------------GGGTGCGGGGTGCGCTTATCAGG----CCGCTTATCTCACA---CATCAA-----------CCCTGTC----GCACCTCTACCACTGTTC--------ACCAGTG-ACACACCAATAGCATCACTTTCATTCCCA--TTTGTTGCTCCAGGAAGCTTTTTGGATCGTTC-------GAAAATCATGCTGACTC------TTTCTACACAGCCGCCGAGCTGGGTAAGG?

# Alignment of the *tub2* sequences used in the second phylogenetic study

>Diaporthe_cinnamomi_CFCC_52569

NNNNGC-CCATGC--TGCTTTCGCA----TCCTCTGC----CCCTGAGCCTAAGGC----TACCCCACCATCGCGACCACACCCACGG--TCAAGCCTCAAAAACACCATCGACACCCTGGGAAGAGCACCCAGATG-CCCTCGGAAGACGCGTCAGATTGCTAACATGAACTTT-TTCTCGACCACAGGTTCACCTTCAGACCGGCCAATGCGTAAGTTGCTTCCTGTCAAC-ACCA-CCGGACCTTATCGCCA-CCTGTAGCTGACACGTTTCCCAGGGTAACCAAATCGGTGCTGCTTTCTGGTGCGTC------------------------CCAG--CTCCAG---CTCCAAGC-------------------------------CTACCACCGCG-ACTCTCGAC-------GCGCGATA-AGACTAGCTCGCAACATCG-TT----ACTGACCTCGGATCTTTAGGCAAACCATCTCTGGCGAGCACGGCCTCGACAGCAATGGCGTGTATGTACCTCCTATTCCC--TGCCCA-CTG-----ATCTCGTCCTCCCCCCCCCCC-GGCTTGGCACTGACAACTGCACAGTTACAACGGCACTTCTGAGCTCCAGCTCGAGCGCATGAACGTCTACTTCAACGAGGTAAGT----CAACA-GCCACGTCGTC------------------AA-TACAAT-TTTGT-C----------CATCTACT-GC-ATGG-TCTCCTGCCGCCGC----CAAG----GTCTTGCTAACGCA-TTATCGCCC-AGGCCTCCGGCAACAAGTATGTGCCTCGCGCCGTCCTCGTCGATCTCGAGCCCGGTACCATGGACGCCGNN

>Diaporthe_acaciarum_CBS_138862

GTCGGC-CCTTGCTGTGCTCTCGCA-CCCTCCTCTGC----CCCTGAGCCTCAGGC----TACCCCACCATCGCGACCACACCCACGG--TCGGGCCC--AAAACACCACCAGCACCCTGCGATGAGCACCCAAATG-CGTTTGGAAGACGCGTCAGATTGCTAACGTGAACTTT-TTCTCGCCCACAGGTTCACCTTCAGACCGGCCAATGCGTAAGTTGCCTCCTGTCAAC-ACC-GCCCGACCTTATCGCCA-CCCATAGCTGACACGTTTCCCAGGGTAACCAAATCGGTGCTGCTTTCTGGTGCGTC------------------------CCAG--CTCCAG---CCCCGAGC-------------------------------CTCCCACCACG-ATGCTCGAC-------GCGCGACA-AGACTAGCTCGCAACATCG-AT----ACTGATCTCGTCTCGTTAGGCAAACCATCTCTGGCGAGCACGGTCTCGACAGCAATGGCGTGTATGTACCTCCTGTTCCC--TGGCAG-CCG-----ATCTCGTC--CTCTCCTCC---GGCTTGGCACTGATGATCGCACAGTTACAACGGCACTTCCGAGCTCCAGCTCGAGCGCATGAACGTCTACTTCAACGAGGTAAGC----CTACG-GCCACGTCTTC------------------AA-TCCAAA-TTTGA-C----------CGTCT-CG-GC-ATGG-TTTACTGCCGCCGC----CAGG----GCCTTGCTAACGCG-CTCTCGCCC-AGGCCTCCGGCAACAAGTATGTGCCTCGCGCCGTCCTCGTCGATCTCGAGCCTGGTACCATGGACGCCGTC

>Diaporthe_beilharziae_BRIP_54792

GTCGGC-CCTTGCTGTGCTCTCGCA-TTCTCCTCTGC----CCCTGAGCCTCAGGC----TACCCCACCATCGCGACCACACCCACGG--TCGGGCTC--AAAACACCACCAGCACCCTGCGAAGAGCACCCAGATG-CGTTTTGAAGACGCGTCAGATTGCTAACATGAACTTT-TTCTTGCCCACAGGTTCACCTTCAGACCGGCCAATGCGTAAGTTGCCTCCTGTCAAC-ACC-GCCCGACCTTATCGCCACCCCGTAGCTGACACGTTTCCCAGGGTAACCAAATCGGTGCTGCTTTCTGGTGCGTA------------------------CCAG--CTCCAG---CTCCGAGC-------------------------------CTGCCACTGCG-ATGCTCGAC-------GCGCGACA-AGACCACCTCCTAGCATCG-TT----ATTGACCTCGTCTCTTTAGGCAAACCATCTCTGGCGAGCACGGTCTCGACAGCAATGGCGTGTATGTACCTCCTATTCCC--GGCCCA-CCG-----ATCTCGTC--CTCTCCTCC---GGCTTGGCACTGATGATCGCACAGTTACAATGGCTCTTCCGAGCTCCAGCTCGAGCGCATGAACGTCTACTTCAACGAGGTAAGC----CTACG-GTCACGTCTTC------------------GA-TCCAAA-TTTGA-C----------CGTCT-CG-GC-ATGG-TTTACTGCCGCCGC----CAGG----GCCTTGCTAACGCG-CTCTCGCCC-AGGCCTCCGGCAACAAGTATGTGCCTCGCGCCGTCCTCGTCGATCTCGAGCCTGGTACCATGGACGCCGTC

>Diaporthe_infecunda_CBS_133812

GTCGGC-CCTTGCTGTGCTCTCGCA-TCCTCCTCTGC----CCCTGAGCCTCAGGC----TACCCCACCATCGCGACCACACCCACGG--TCGGGCTC--AAAACACCACCAGCACCCTGCGATGAGCACCCAGATA-TGTTTGGAAGACGCGTCAGATTGCTAACATGAACTTT-TTCTTGCCCACAGGTTCACCTTCAGACCGGCCAATGCGTAAGTTGCCTCCTGTCAAC-ACCG-CCCGACCTTATCGCCA-CCCATAGCTGACACGTTTCCCAGGGTAACCAAATCGGTGCTGCTTTCTGGTGCGTA------------------------CCAG--CTCCAG---CTCCGAGC-------------------------------CTGCCACCGCG-ATGCTCGAC-------GCGCGACA-AGACCACCTCCAAGCATCG-TT----ACTGACCTTGTTTCTTTAGGCAAACCATCTCTGGCGAGCACGGTCTCGACAGCAATGGCGTGTATGTACCTCCTATTCCC--TGCCCA-CCG-----ATCTCGTC--CTCTCCTCC---GGCTTGGCACTGATGATCGCACAGTTACAACGGCACTTCCGAGCTCCAGCTCGAGCGCATGAACGTCTACTTCAACGAGGTAAGC----CTATG-GCCACGTCTTC------------------AA-TCCAAG-TTTGA-C----------CGTCT-CG-GC-ATGG-TTTACTGCCGCCGC----CAGG----GCCTTGCTAATGCG-CTCTTGCCC-AGGCCTCCGGCAACAAGTATGTGCCTCGCGCCGTCCTCGTCGATCTCGAGCCCGGTACCATGGACGCCGTC

>Diaporthe_serafiniae_BRIP_55665a

GTCGGC-CCTTGCTGTGCTCTCGCA-TCCTCCTCTGC----CCCTGAGCCTCAGGC----TACCCCACCATCGCGACCACACCCACGG--TCGGGCTC--AAAACACCACCAGCACCCTGCGAAGAGCACCTAGATG-CGTTTGGAAGACGCGTCAGATTGCTAACATGAACTTT-TTCTTGCCCACAGGTTCACCTTCAGACCGGCCAATGCGTAAGTTGCCTCCTGTCAAC-ACCG-CCCGACCTTATCGCCA-CCCATAGCTGACACGTTTCCCAGGGTAACCAAATCGGTGCTGCTTTCTGGTGCGTA------------------------CCAG--CTCCAG---CTCCGAGC-------------------------------CTGCCACCGCG-ATGCTCGAC-------GCGCGACA-AGACCACCTCCAAGCATCG-TT----ACTGACCTTGTTTCTTTAGGCAAACCATCTCTGGCGAGCACGGTCTCGACAGCAATGGCGTGTATGTACCTCCTATTCCC--TGCCCA-CCG-----ATCTCGTC--CTCTCCTCC---GGCTTGGCACTGATGATCGCACAGTTACAACGGCACTTCCGAGCTCCAGCTCGAGCGCATGAACGTCTACTTCAACGAGGTAAGC----CTATG-GCCACGTCTTC------------------AA-TCCAAG-TTTGA-C----------CGTCT-CG-GC-ATGG-TTTACTGCCGCCGC----CAGG----GCCTTGCTAATGCG-CTCTCGCCC-AGGCCTCCGGCAACAAGTATGTGCCTCGCGCCGTCCTCGTCGATCTCGAGCCCGGTACCATGGACGCCGTC

>Diaporthe_rossmaniae_CAA762

GTCGGC-CCTTGCTGTGCTCTCGCA-CCCTCCTCTGC----CCCTGAGCCTCAGGC----TACCCCACCATCGCGACCACACCCACGG--TCGGGCTC--AAAACACCACCAGCACCCTGCGATGAGCACCCAGATA-CGTTCGGAAGACGCGTCAGATTGCTAACATGAACTTT-TTCTTGCCCACAGGTTCACCTTCAGACCGGCCAATGCGTAAGTTGCCTCCTGTCAAC-ACCG-CCCGACCTTATCGCCA-CCCATAGCTGACACGTTTCCCAGGGTAACCAAATCGGTGCTGCTTTCTGGTGCGTA------------------------CCAG--CTCCAG---CTCCGAGC-------------------------------CTGCCACCGCG-ATGCTCAAC-------GCGCGACA-AGACCACCTCCAAGCATCG-AT----ACTGACCTTGTTTCTTTAGGCAAACCATCTCTGGCGAGCACGGTCTCGACAGCAATGGCGTGTATGTACCTCCTATTCCC--TGCCTA-CCG-----ATCTCGTC--CTCTCCTCC---GGCTTGGCACTGATGATCGCACAGTTACAACGGCACTTCCGAGCTCCAGCTCGAGCGCATGAACGTCTACTTCAACGAGGTAAGC----CTACG-GCCACGTCTTC------------------AA-TCCAAA-TTTGA-C----------AGTCT-CG-GC-ATGG-TTTACTGCCGCCGC----CAGG----GCCTTGCTAATGCG-CTCTTGCCC-AGGCCTCCGGCAACAAGTATGTGCCTCGCGCCGTCCTCGTCGATCTCGAGCCCGGTACCATGGACGCCGTC

>Diaporthe_sackstonii_BRIP_54669b

GTCGGC-CCTTGCTGCGCTCTCGCA----TCCTCTGC----CCCTGAGCCTCAGGC----TACCCCACCATCGCGAC--CACCTACGG--TCGGGCTC-AAAAACACCACCAGCACCCTGCGATGAGCACACAGATG-CGTTTGGAAGACGCGTCAGATTGCTAACATGAACTTT-TTCTTGCCCACAGGTTCACCTTCAGACCGGCCAATGCGTAAGTTGCCTCCTGTCAAC-ACCG-CCCGACCTTATCGCCA-CCCATAACTGACACGTTTCCCAGGGTAACCAAATCGGTGCTGCTTTCTGGTGCGTA------------------------CCAG--CTCCAG---CTCCGAGC-------------------------------CTGCCACCGCG-ATGCTCGAC-------GCGCGACA-AGATCACCTCCAAGCATCG-TT----ACTGACCGTGTTTCTTTAGGCAAACCATCTCTGGCGAGCACGGTCTCGACAGCAATGGCGTGTATGTACCTCCTATTCCC--TGCCCA-CCG-----ATCTCATC--CTCTCCTCC---GGCTTGGCACTGATGATCGCACAGTTACAACGGCACTTCCGAGCTCCAGCTCGAGCGCATGAACGTCTACTTCAACGAGGTAAGC----CTACG-GACACGTCTTC------------------AA-TCCAAA-TTTGA-C----------CGTCT-CG-GC-ATGG-TTTACTGCCGCCGC----CAGG----GCCTTGCTAATGCG-CTCTCGCCC-AGGCCTCCGGCAACAAGTATGTGCCTCGCGCCGTCCTCGTCGATCTCGAGCCCGGTACCATGGACGCCGTC

>Diaporthe_caryae_CFCC_52563

NNNNGC-CCTTGCTGCGCTCTCGCA----TCCTCTGC----CCCTGAGCCTCAGGC----TACCCCACCATCGCGACCACACCCACGG--TCGGGCTC--AAAACACCACCAGCACCCTGCGATGAGCACCCAGATA-TGTTTGGAAGACGCGTCAGATTGCTAACATGAACTTT-TTCTTGCCCACAGGTTCACCTTCAGACCGGCCAATGCGTAAGTTGCCTCCTGTCAAC-ACCG-CCCGACCTTATCGCCACCCCGTAGCTGACACGTTTCCCAGGGTAACCAAATCGGTGCTGCTTTCTGGTGCGTA------------------------CCAG--CTCCAG---CTCCGAGC-------------------------------CTGCCACCGCG-ACGCTCGAC-------GCGCGACA-AGACTAGCTCGCAACATCT-TT----ACTGACCTCGTCTCTTTAGGCAAACCATCTCTGGCGAGCACGGTCTCGACAGCAATGGCGTGTATGTACCTCCTATTCCC--TGCCCA-CCG-----ATCTCGTC--CTCTCCTCC---GGCTTGGCACTGATGATCGCACAGTTACAACGGCACTTCCGAGCTCCAGCTCGAGCGCATGAATGTCTACTTCAACGAGGTAAGC----CTATG-GCCACGTCTTC------------------AA-TCCAAA-TTTGA-C----------CGTCT-CG-GC-ATGG-TTTACTGCCGCCGC----CAGG----GCCTTGCTAATGCG-CTCTCCCCC-AGGCCTCCGGCAACAAGTATGTGCCTCGCGCCGTCCTCGTCGATCTCGAGCCCGGTACCATGGACGCCGNN

>Diaporthe_middletonii_BRIP_54884e

GTCGGC-CCTTGCTGTGCCCTCGCA-TCCTCCTCTGC----CCCTGAACCTCAGGC----TACCCCACCATCGCGACCACACCCACGG--TCGGGCTC--AAAACACCACCAGCACCCTGCGATGAGCACCAAGATT-CGTTTGGAAGACGCGTCACATTGCTAACATGAACTTT-CTCCT--CCACAGGTTCACCTTCAGACCGGCCAATGCGTAAGTTGCCTCCTGTCAAC-ATCG-CCCGACCTTATCGCCACCCCGTAGCTGACACGTTTCCCAGGGTAACCAAATCGGTGCTGCTTTCTGGTGCGTA------------------------CCAG--CTCCAG---CTCCGAGC-------------------------------CTGCCACCGCG-ACACTCGAC-------GCGCGACA-ATACTAGCTCGCAATATCG-TT----GCTGACCTCGTCTCTTCAGGCAAACCATCTCTGGCGAGCACGGTCTCGACAGCAATGGCGTGTATGTACCTCCTATTCCC--TGCCGA-CCG-----ATCTCATC--CTCTCCTCC---GGCTTGGCACTGATGATCGCACAGTTACAACGGCACTTCCGAGCTCCAGCTCGAGCGCATGAACGTCTACTTCAACGAGGTAAGC----CTATG-GCCACGTCTTC------------------GA-TCCAAG-TTTGA-C----------CGTCT-CG-GC-ATGG-TTTACTGCCGCCGC----CAGG----ACCTTGCTAATGCG-CTCTCGCCC-AGGCCTCCGGCAACAAGTATGTGCCTCGCGCCGTCCTCGTCGATCTCGAGCCCGGTACCATGGACGCCGTC

>Diaporthe_alangii_CFCC_52556

NNNNGC-CCATGC--TGCTTTCGCA----TCCTCTGC----CCCTGAACCTCAGGC----TACCCCACCATCGCGACCACACCCACAG--TCAGGCCTCAAAAACACCATCAACACCCTGGGAAAGGCCACCCAGATGCTCTCAAAAGACGCGTCGGATTGCTAACATGGACTTT-TTCTTGCCCACAGGTTCACCTCCAGACTGGCCAATGCGTAAGTTGCTTCCTGTCAAC-ACCG-CCCGACCTTATCGCCA-CCTCTAGCTGACACGTTTCCCAGGGTAACCAAATCGGTGCTGCTTTCTGGTGCGTC------------------------CCAG--CTCCAG---CTCAGAGT-------------------------------CGACCACCGCG-ACAATCGAC-------GAGCGACA-ACAGTAGCTCGTAGCATTG-AT----ACTGACATCGGCTC-CTAGGCAAACCATCTCTGGCGAGCACGGCCTCGACAGCAATGGCGTGTATGCACCTCCTATTCCC--TGCCTA-CTG-----GTCTCGTCCTCTCTCCTAC---GCCTTGGCACTGACAATGGCACAGCTACAACGGCACTTCTGAGCTCCAGCTCGAGCGCATGAACGTCTACTTCAACGAGGTAAGC----CAAAG-CCCACGTTGTC------------------AA-TCCGGA-TTTGA-C----------CACCTGCA-GC-ACAA-TCCCTTGCCACCGC----CAAG----GCCTAGCTAACGCG-TTATCGTCC-AGGCCTCCGGCAACAAGTATGTGCCTCGCGCCGTCCTCGTCGATCTCGAGCCCGGTACCATGGATGCCGNN

>Diaporthe_tulliensis_BRIP_62248a

GTCGGC-CCATGC--TGCTTTCGCA----TCCTCTGC----CCCTGAACCTCAGGC----TACCCCACCATCGCGACCACACCCACAG--TCAGGCCTCAAAAACACCATCAACACCCTGGGAAAGACCACCCAGATGCTCTCAAAAGACGCGTCGGATTGCTAACATGGACTTT-TTCTCGCCCACAGGTTCACCTCCAGACTGGCCAATGCGTAAGTTGCTTCCTGTCAAC-ACCG-CCCGACCTTATCGCCA-CCTGTAGCTGACACGTTTCCCAGGGTAACCAAATCGGTGCTGCTTTCTGGTGCGTC------------------------CCAG--CTCCAG---CTCAGAGT-------------------------------CGACCACCGCG-ACAATCGAC-------GCGCGACA-ACAGTAGCTCGTAGCATTG-TT----ACTGACATCGGCTC-CTAGGCAAACCATCTCTGGCGAGCACGGCCTCGACAGCAATGGCGTGTATGCACCTCCTATTCCC--TGCCTA-CTG-----GTCTCGTC--CTCTCCTAC---GCCTTGGCACTGACAATGGCACAGCTACAACGGCACTTCTGAGCTCCAGCTCGAGCGCATGAACGTCTACTTCAACGAGGTAAGC----CAAAG-CCCACGTTGTC------------------AA-TCCGGA-TTTGA-C----------CATCTGCG-GC-ACAA-TCCCCTGCCACCGC----CAAG----GCCTAGCTAACGCG-TTATCGTCC-AGGCCTCCGGCAACAAGTATGTGCCTCGCGCCGTCCTCGTCGATCTCGAGCCCGGTACCATGGATGCCGTC

>Diaporthe_ambigua_CBS_114015

GTCGGC-CCATGC--TGCTTTCGCA-TCCTCCTCTGC----CCCTGAACCTGAGGC----TACCCCACCATCGCGACCACACCCACGG-TTCAAACCTC-AAAACACCATCAACACCCTGGGAAGAGCACCCAGATG-CACTCGGAAGACGCGTCAGATTGCTAACATGAACTTT-TTCTCGCCCACAGGTTCACCTTCAGACCGGCCAATGCGTAAGTTGCTCCCTGTCAAC-ACCACCCGGACCTTATCGCCA-CCTGTAGCTGACACGTTTCCCAGGGTAACCAAATCGGTGCTGCTTTCTGGTGCGTC------------------------CCAG--CTCCAG---CTCCAAGT-------------------------------CTACAACCGCG-ACACTCGAC-------GCGCGACA-ACACTAGCTCGGGGCATCA-TT----ACTGACCTCAGCTCT-TAGGCAAACCATCTCTGGCGAGCACGGTCTCGACAGCAATGGCGTGTACGTACCTCGTATCCCC--TGCCCA-CTG-----GTCTCGTC--CTCTCCCTC---GGCTTGGCACTGACAACTGCACAGTTACAACGGCACTTCCGAGCTCCAGCTCGAGCGCATGAACGTCTACTTCAACGAGGTAAGT----CAACA-GCCACGTCGTC------------------AA-TTCAAA-TTTGA-C----------AACCTACG-GC-ATGG-TTTCCCGCCGTCGC----CAAG----GCCTTGCTAACGCATTTATCGCCC-AGGCCTCCGGCAACAAGTATGTGCCTCGCGCCGTCCTCGTCGATCTCGAGCCCGGTACCATGGACGCCGTC

>Diaporthe_longispora_CBS_194_36

GTCGGC-CCATGC--TGCTTTCGCA-TCCTCCTCTGC----CCCTGAACCTCAGGC----TACCCCACCATCGCGACCACGCCCACGG--TCAGGCCTC-AAAACACCACCAACACCCTGCTGGGAGCACCCAGATG-CTCTCCGAAGACACGTCAGATTGCTAACATGGTCTTT-TTCTCGCCCACAGGTTCACCTTCAGACCGGCCAATGCGTAAGTTGCTTCCTGTCAAC-ACCACCCGGACCTCATCGCCA-CCTGTAGCTGACACGTTTCCCAGGGTAACCAAATCGGTGCTGCTTTCTGGTGCGTC------------------CCAGCTCCAG--CTCCAG---CTCCAAGT-------------------------------CTGCCGCCGCG-ACCCTCGAC-------GCGCGACA-ACACTAGCTCGCGACATCA-TC----ACTGACCTCGCCTCT-TAGGCAAACCATCTCTGGCGAGCACGGTCTCGACAGCAATGGCGTGTATGTACCTCTTATTCCC--TGCCTATCTG-----ATCTCGTC--CCCTCCTCC---GGCTTGACACTGACAATTGCACAGTTACAACGGCACTTCCGAGCTCCAGCTCGAGCGCATGAACGTCTACTTCAACGAGGTAAGT----CAACA-GCCACGTCGTC------------------AA-TCCAAA-TTTGA-C----------CAC--ACG-GC-ATGG-TTTCCTGCCGGCGC----CAAG----GCCTTGCTAACGCG-TTATCGCCC-AGGCTTCCGGCAACAAGTATGTGCCTCGCGCCGTCCTCGTCGATCTCGAGCCCGGTACCATGGATGCCGTC

>Diaporthe_sclerotioides_CBS_296_67

GTCGGCACCATGT--TGCTTTCGCA-TCCTCCTCTGC----CCCTGAACCTCAGGC----TACCCCACTATCGCGACCACACCCACAG--TCGGGCTTCAAAAACATCACCAACACCCTGGGAAAAGCACCCAGATGCCACTCGGAAGACGCGTCAGATTGCTAACATGGACTTT-TTCTCGCCCATAGGTTCACCTTCAGACCGGCCAATGCGTAAGTTGCTTCCTGTCAAC-ACCACCCGCACCTTATCGCCA-CCTGTAGCTGACACGTTTCCCAGGGTAACCAAATCGGTGCTGCTTTCTGGTGCGTC------------------------CCAG--CTCCAG---CTCCAAGC-------------------------------CTGCCGCCGCG-ACGCTCGAC-------TCGCGACA-GCACTAGCTCGCGGCATCA-TT----ACTAACCGCAGCTCT-TAGGCAAACCATCTCTGGCGAGCACGGTCTCGACAGCAATGGCGTGTATGTACCTCCTATTCCC--TGCCCAACTG-----ATCCCGTC--CTCTCCTCC---GGCTTGGCACTGACAATTGCACAGTTACAACGGCACTTCCGAGCTCCAGCTCGAGCGTATGAATGTCTACTTCAACGAGGTAAGT----CAACA-GCCACGTCGTC------------------AA-TCCGAA-TTTAAAC----------AACTTACG-GC-ATGG-TTTCCTGCCGCCGC----CAAGC---GCCTTGCTAACGCG-TCATCGTCC-AGGCCTCCGGCAACAAGTATGTGCCTCGCGCCGTCCTCGTCGATCTCGAGCCCGGTACCATGGACGCCGTC

>Diaporthe_mayteni_CBS_133185

GTCGGC-CCTTGC--TGCTTTCGCA-TCTTCCTCTGC----CCCTGAGCCTCAGGC----TACCCCACCATCGCGACCACACCCACGG--TCAGGCCTC-AAAACATCACCAACACCCTGAGGAGAGCACCATGATG-CCCTCGGAACACGCGTCAGATTGCTAACATGGACTTT-TTCTCGCCCACAGGTTCACCTTCAGACCGGCCAATGCGTAAGTTGCTTTCTGTCAAC-ACCT-CCAGACCTTATCGCCA-CCTGTAGCTGACACGTTTCGCAGGGTAACCAAATCGGTGCTGCTTTCTGGTGCGTC------------------------CCAG--CTCCAG---CTCCAAGT-------------------------------CTACCACCGCG-ACGCTCGAC-------ACGCGACA-AGACTAGCTCACAGCATCT-TT----ACTGACCTCTGCCCT-TAGGCAAACCATCTCTGGCGAGCACGGCCTCGACAGCAATGGCGTGTATGCACCTCCTATTCCC--TGAACA-ATG-----TTCTCGTC--CTCTCCTCC---GGCTTGGCACTGATGATCGCACAGTTACAACGGCACTTCCGAGCTCCAGCTCGAGCGCATGAACGTCTACTTCAACGAGGTAAGT----CAAAA-GCCACGTCGTG------------------AA-TTCAAT-TTTGACC----------CTCTTACG-GC-ATGA-TTTCCTGCCGCCGC----CAAG----GCCTTGCTAACGCG-TTATCGCCC-AGGCTTCCGGCAACAAGTATGTGCCTCGCGCCGTCCTCGTCGATCTCGAGCCCGGTACCATGGACGCCGTC

>Diaporthe_goulteri_BRIP_55657a

NNNNNNNNNNNNNNNNNNNNNNNNNNNNNNNNNNNNNNNNNNNNNNNNNNNNNNNNNNNNNNNNNNNNNATCGCGACCACACCCACAT--TCAGGC-TC-AAAACACCATCAAAACCCTTGGAAGAGCACCCAGGTG-TCCTCTGAAGACGCGTCAGATTGCTAACGTGAACTTT-CTCTCGCCCACAGGTTCACCTCCAGACCGGCCAGTGCGTAAGTTGCTCCCTGTCAAC-ACCACCCGGACCTTATCGCCA-CCTGTAGCTGACACGTTTCCCAGGGTAACCAAATCGGTGCTGCTTTCTGGTGCGTC------------------------CCTG--CTCCAG---TCCCAAGC-------------------------------CTACGACCGCG-ATACTCGAC-------GCGCGAGA-ATACCAGCTCGCATCATCA-TC----ACTGACCTCAGCTC-TTAGGCAAACCATCTCTGGCGAGCACGGTCTCGACAGCAATGGCGTGTATGCACCTCCTATCCCC--CATCCA-CCG-----GTCTCGTC--CACTTCTCC---GGCTTGCCACTGACAATCGCACAGCTACAATGGCACCTCCGAGCTCCAGCTCGAGCGCATGAACGTCTACTTCAACGAGGTAAGT----CTACA-GTCACTTCGTC------------------AA-TCTAAA-TTTGA-T----------CACCTGCG-GCATTGG-TTTGCTGCCGCCGC----CAAG----GCCCTGCTAACGCG-TTCTCGCCC-AGGCCTCCGGCAACAAGTATGTGCCTCGCGCCGTCCTCGTCGATCTCGAGCCCGGTACCATGGACGCCGTC

>Diaporthe_arezzoensis_MFLU_19_2880

NNNNNNNNNNNNNNNNNNNNNNNNNNNNNNNNNNNNNNNNNNNNNNNNNNNNNNNNNNNNNNNNNNNNNNNNNNNNNNNNNNNNNNNNNNNNNNNNNNNNNNNNNNNNNNNNNNNNNNNNNNNNNNNNNNNNNNNNNNNNNNNNNNNNNNNNNNNNNNNNNNNNNNNNNNNNNNNNNNNNNNNNNNNNNNNNNNNNNNNNNNNNNNNNNNNNNNNNNNNNNNNNNNNNNNNNNNNNNNNNNNNNNNNNNNNNNNNNNNNNNNNNNNNNNNNNNNNNNNNNNNNNNNNNNTCGGTGCTGCTTTCTGGTGCGTC------------------------CCAG--CTCCAG---CTCCGAGC-------------------------------CTGCCACCGCG-ACGCTCGAC-------GC-CGGCA-ACACTAGCTCGCAACATCG-TTCCTGACTGACCTCGGCTCTTTAGGCAAACCATCTCTGGCGAGCACGGTCTCGACAGCAATGGCGTGTATGCACCTCCTATTCCC--TGCCCA-CTG-----ATCTCGTC--CTCCCCTCC---GGCTTGGCACTGATGATTATACAGTTACAACGGCACTTCCGAGCTCCAGCTCGAGCGCATGAGCGTCTACTTCAACGAGGTATGT----CAACA-GCCACGTCGTC------------------AA-TTCAAA-CTTGA-C----------CGTCT-CG-GCAATGG-TTCGCTGCCGCCGC----AAAT----GCCTTGCTAACGCG-TCTTCGCCC-AGGCCTCCGGCAACAAGTATGTGCCTCGCGCCGTCCTCGTCGATCTCGAGCCCGGTACCATGGACGCCGTC

>Diaporthe_tectonae_MFLUCC_12_0777

NNNNNNNNNNNNNNNNNNNNNNNNNNNNNNNNNNNNNNNNNNNNNNNNNNNNNNNNNNNNNNNNNNNNNNNNNNNNNNNNNNNNNNNNNNNNNNNNNNNNNNNNNNNNNNNNNNNNNNNNNNNNNNNNNNNNNNNNNNNNNNNNNNNNNNNNNNNNNNNNNNNNNNNNNNNNNNNNNNNNNNNNNNNNNNNNNNNNNNNNNNNNNNNNNNNNNNNNNNNNNNNNNNNNNNNNNNNNNNNNNNNNNNNNNNNNNNNNNNNNNNNNNNNNNNNNNNNNNNNNNNNNNNNNNNNNNNNNNNNNNNNNNNNNNNNC------------------------TCAG--CT----------AGAGT-------------------------------CGACCACCGCG-ACAATCGAC-------GCGCGACA-ACAGTAGCTCGTAGCATTG-AT----ACTGACATCAGCTC-CTAGGCAAACCATCTCTGGCGAGCACGGCCTCGACAGCAATGGCGTGTATGCACCTCCTATCCCC--TGCCTA-CGG-----GTCTCGTC--CTCTCCTAC---GCCTCGGCACTGACAATGGCACAGCTACAACGGCACTTCTGAGCTCCAGCTCGAGCGCATGAACGTCTACTTCAACGAGGTAAGC----CAAAG-CCCACGTTGTC------------------AA-TCCGGA-TTTGA-C----------CATCTGCG-GC-ACAA-TCCCCTGCCACCGC----CAAG----GCCTAGCTAACGCG-TTATCGTCC-AGGCCTCCGGCAACAAGTATGTGCCTCGCGCCGTCCTCGTCGATCTCGAGCCCGGTACCATGGATGCCGTC

>Diaporthe_myracrodruonis_URM7972

NNNNNNNNNNNNNNNNNNNNNNNNNNNNNNNNNNNNNNNNNNNNNNNNNNNNNNNNNNNNNNNNNNNNNNNNNNNNNNNNNNNNNNNNNNNNNNNNNNNNNNNNNNNNNNNNNNNNNNNNNNNNNNNNNNNNNNNNNNNNNNNNNNNNNNNNNNNNNNNNNNNNNNNNNNNNNNNNNNNNNNNNNNNNNNNNNNNNNNNNNNNNNNNNNNNNNNNNNNNNNNNNNNNNNNNNNNNNNNNNNNNNNNNNNNNNNNNNNNNNNNNNNNNNNNNNNNNNNNNNNNNNNNNNNNNNNTGCTGCTTTCTGGTGCGTA------------------------ACTG--CTCCAG---CTCCGAGC-------------------------------CTGCCACCGCG-GCGCTCGAC-------GCGCGACA-ATACTAGCTCGCAACATCG-TT----ACTGACCTCGTCTCGTTAGGCAAACCATCTCTGGCGAGCACGGTCTCGACAGCAATGGCGTGTATGTACCTCCTGTTCCC--TGCCCA-CCG-----ATCTCGTC--CTCTCCTCC---GGCTTGGCACTGATGATCGCACAGTTACAACGGCACTTCCGAGCTCCAGCTCGAGCGCATGAACGTCTACTTCAACGAGGTAAGC----CTACG-GCCACGTCTTC------------------AA-TTCAAA-TTTGA-C----------CGTCT-CG-GC-ATGG-TTTACTGCCGCCGC----CAGG----GCCTTGCTAACGCG-CTTTCGCCC-AGGCCTCCGGCAACAAGTATGTGCCTCGCGCCGTCCTCGTCGATCTCGAGCCCGGTACCATGGACGCCGTC

>Diaporthe_hubeiensis_JZB320123

NNNNNNNNNNNNNNNNNNNNNNNNNNNNNNNNNNNNNNNNNNNNNNNNNNNNNNNNNNNNNNNNNNNNNNNNNNNNNNNNNNNNNNNNNNNNNNNNNNNNNNNNNNNNNNNNNNNNNNNNNNNNNNNNNNNNNNNNNNNNNNNNNNNNNNNNNNNNNNNNNNNNNNNNNNNNNNNNNNNNNNNNNNNNNNNNNNNNNNNNNNNNNNNNNNNNNNNNNNNNNNNNNNNNNNNNNNNNNNNNNNNNNNNNNNNNNNNNNNNNNNNNNNNNNNNNNNNNNNNNNNNNNNNNNNNGGTGCTGCTTTCTGGTGCGTT------------------------CCCG--CT-CAG---CTCAGAGT-------------------------------CGACCACCGCG-ACAATCGAC-------GCGCGACA-ACAGTAGCTCGTGGCATTG-AT----ACTGACATCGGCTC-CTAGGCAAACCATCTCTGGCGAGCACGGCCTCGACAGCAATGGCGTGTATGCACCTCCTATTCCC--TGCCTA-CTG-----GTCTCGTCCTCTCTCCTAC---GCCTTGGCACTGATAATGGCACAGCTACAACGGCACTTCTGAGCTCCAGCTCGAGCGCATGAACGTCTACTTCAACGAGGTGAGC----CAAAG-CCCACGTTGTC------------------AA-TCCGGA-TTTGA-C----------CACCTGCA-GC-ACAA-TCCCCTGCCACCGC----CAAG----GCCTAGCTAACGCG-TTATCGTCC-AGGCCTCCGGCAACAAGTATGTGCCTCGCGCCGTCCTCGTCGATCTCGAGCCCGGTACCATGGATGCCGTC

>Diaporthe_celtidis_NCYU_19_0357

NNNNNNNNNNNNNNNNNNNNNNNNNNNNNNNNNNNNNNNNNNNNNNNNNNNNNNNNNNNNNNNNNNNNNNNNNNNNNNNNNNNNNNNNNNNNNNNNNNNNNNNNNNNNNNNNNNNNNNNNNNNNNNNNNNNNNNNNNNNNNNNNNNNNNNNNNNNNNNNNNNNNNNNNNNNNNNNNNNNNNNNNNNNNNNNNNNNNNNNNNNNNNNNNNNNNNNNNNNNNNNNNNNNNNNNNNNNNNNNNNNNNNNNNNNNNNNNNNNNNNNNNNNNNNNNTTTCCCAGGGTAACCAAATCGGTGCTGCTTTCTGGTGCGTC------------------------CCAG--CTCCAG---CTCAGAGT-------------------------------CGACCACCGCG-ACAATCGAC-------GCGCGACA-ACAGTAGCTCGTAGCATTG-TT----ACTGACATCGGCTC-CTAGGCAAACCATCTCTGGCGAGCACGGCCTCGACAGCAATGGCGTGTATGCACCTCCTATCCCC--TGCCTA-CTG-----GTCTCGTC--CTCTCCTCC---GGCTTGGCACTGACAATGGCACAGCTACAACGGCACTTCTGAGCTCCAGCTCGAGCGCATGAACGTCTACTTCAACGAGGTAAGC----CAAAG-CCCACGTTGGC------------------AA-TCCGGA-TTTGA-C----------CATCTGCG-GC-ACAA-TCCCCTGCCACCGC----CAAG----GCCTAGCTAACGCG-TTATCGTCC-AGGCCTCCGGCAACAAGTATGTGCCTCGCGCCGTCCTCGTCGATCTCGAGCCCGGTACCATGGATGCCGTC

>Diaporthe_leucospermi_CBS_111980

NNNNNNNNNNNNNNNNNNNNNNNNNNNNNNNNNNNNNNNNNNNNNNNNNNNNNNNNNNNNNNNNNNNNNNNNNNNNNNNNNNNNNNNNNNNNNNNNNNNNNNNNNNNNNNNNNNNNNNNNNNNNNNNNNNNNNNNNNNNNNNNNNNNNNNNNNNNNNNNNNNNNNNNNNNNNNNNNNNNNNNNNNNNNNNNNNNNNNNNNNNNNNNNNNNNNNNNNNNNNNNNNNNNNNNNNNNNNNNNNNNNNNNNNNNNNNNNNNNNNNNNNNNNNNNNNNNNNNNNNNNNNNNNNNNNNNNNNNNNNNNNTGGTGCGTA------------------------CCAG--CTCCAG---CTCCGAGT-------------------------------CTGCCACCGCG-ATGCTCAAC-------GCGCGACA-AGACCACCTCCAAGCATCG-AT----ACTGACCTTGTTTCTTTAGGCAAACCATCTCTGGCGAGCACGGTCTCGACAGCAATGGCGTGTATGTACCTCCTATTCCC--TGCCTA-CCG-----ATCTCGTC--CTCTCCTCC---GGCTTGGCACTGATGATCGCACAGTTACAACGGCACTTCCGAGCTCCAGCTCGAGCGCATGAACGTCTACTTCAACGAGGTAAGC----CTACG-GCCACGTCTTC------------------AA-TCCAAA-TTTGA-C----------AGTCT-CG-GC-ATGG-TTTACTGCCGCCGC----CAGG----GCCTTGCTAATGCG-CTCTTGCCC-AGGCCTCCGGCAACAAGTATGTGCCTCGCGCCGTCCTCGTCGATCTCGAGCCCGGTACCATGGACGCCGTC

>Diaporthe_pyracanthae_CBS142384

NNNNNNNNNNNNNNNNNNNNNNNNNNNNNNNNNNNNNNNNNNNNNNNNNNNNNNNNNNNNNNNNNNNNNNNNNNNNNNNNNNNNNNNNNNNNNNNNNNNNNNNNNNNNNNNNNNNNNNNNNNNNNNNNNNNNNNNNNNNNNNNNNNNNNNNNNNNNNNNNNNNNNNNNNNNNNNNNNNNNNNNNNNNNNNNNNNNNNNNNNNNNNNNNNNNNNNNNNNNNNNNNNNNNNNNNNNNNNNNNNNNNNNNNNNNNNNNNNNNNNNNNNNNNNNNNNNNNNNNNNNNNNNNNNNNNNNNNNNNNNNNTGGTGCGTA------------------------CCAG--CTCCAG---CTCCGAGC-------------------------------CTGCCACCGCG-ATGCTCGAC-------GCGCGACA-AGACCACCTCCAAGCATCG-AT----ACTGACCTTGTTCCTTTAGGCAAACCATCTCTGGCGAGCACGGTCTCGACAGCAATGGCGTGTATGTACCTCCTATTCCC--TGCCCA-CCG-----ATCTCGTC--CTCTCCTCC---GGCTTGGCACTGATGATCGCACAGTTACAACGGCACTTCCGAGCTCCAGCTCGAGCGCATGAACGTCTACTTCAACGAGGTAAGC----CTACG-GCCACGTCTTC------------------AA-TCCAAA-TTTGA-C----------CGTCT-CG-GC-ATGG-TTTACTGCCGCCGC----CAGG----GCCTTGCTAACGCG-CTCTCGCCC-AGGCCTCCGGCAACAAGTATGTGCCTCGCGCCGTCCTCGTCGATCTCGAGCCCGGTACCATGGACGCCGTC

>Diaporthe_yunnanensis_CGMCC_3_18289

NNNNNNNNNNNNNNNNNNNNNNNNNNNNNNNNNNNNNNNNNNNNNNNNNNNNNNNNNNNNNNNNNNNNNNNNNNNNNNNNNNNNNNNNNNNNNNNNNNNNNNNNNNNNNNNNNNNNNNNNNNNNNNNNNNNNNNNNNNNNNNNNNNNNNNNNNNNNNNNNNNNNNNNNNNNNNNNNNNNNNNNNNNNNNNNNNNNNNNNNNNNNNNNNNNNNNNNNNNNNNNNNNNNNNNNNNNNNNNNNNNNNNNNNNNNNNNNNNNNNNNNNNNNNNNNNNNNNNNNNNNNNNNNNNNNGGTGCTGCTTTCTGGTGCGTC------------------------CCAG--CTCCAG---CTCCAAGC-------------------------------CTACCATCGCG-ACTCTCGTC-------GCGCGACA-ACACTAGCTTGCAGCATCG-TT----ACTGACATCTGCTCTTTAGGCAAACCATCTCTGGCGAGCACGGCCTCGACAGCAATGGCGTGTATGTGCCTCCTATTCTC--TGCCCA-ATG-----ATCTCGTC--CTCTCCTCC---CGCTTGGCACTGACAATTGCATAGTTACAACGGCACTTCCGAGCTCCAGCTCGAGCGCATGAACGTCTACTTCAACGAGGTCAGT----AAACA-GCCACGTCGCC------------------AA-TTCACA-TCTGA-C----------CATCTACG-AC-ATGG-TTCCATGTCGCCGC----CAAG----GTCTTGCTAACGCG-TTATCGCCC-AGGCCTCCGGCAACAAGTATGTGCCTCGCGCCGTCCTCGTCGATCTCGAGCCCGGTACCATGGATGCCGTC

>Diaporthe_citriasiana_CBS_134240

NNNNNNNNNNNNNNNNNNNNNNNNNNNNNNNNNNNNNNNNNNNNNNNNNNNNNNNNNNNNNNNNNNNNNNNNNNNNNNNNNNNNNNNNNNNNNNNNNNNNNNNNNNNNNNNNNNNNNNNNNNNNNNNNNNNNNNNNNNNNNNNNNNNNNNNNNNNNNNNNNNNNNNNNNNNNNNNNNNNNNNNNNNNNNNNNNNNNNNNNNNNNNNNNNNNNNNNNNNNNNNNNNNNNNNNNNNNNNNNNNNNNNNNNNNNNNNNNNNNNNNNNNNNNNNNNNNNNNNNGGTAACCAAATCGGTGCTGCTTTCTGGTGCGTC------------------------CCAG--CTCCAG---CTCCAAGC-------------------------------CTACCACCGCG-ACTCTTGAC-------GCGCGACA-ACACTAGCTCGCAACATCG-TT----ACTGACCTCGGTTCTTTAGGCAAACCATCTCTGGCGAGCACGGTCTCGACAGCAATGGCGTGTATGCACCTCCTATTCCC--TGCCCA-CTG-----ATCTCGTC--GTCTCCTCC---GGCCTCGCACTGACAATTGCACAGTTACAACGGCACTTCCGAGCTCCAGCTCGAGCGCATGAACGTCTACTTCAACGAGGTAAGT----CAACA-GCCACGTCGTC------------------AA-TACGAT-TTTGT-C----------CATCTACG-GC-ACGG-CCTCCTGCCGCCGC----TAAG----GTCTTGCTAACGCG-TCATCGCCC-AGGCCTCCGGCAACAAGTATGTGCCTCGCGCCGTCCTCGTCGATCTCGAGCCCGGTACCATGGACGCCGTC

>Diaporthe_fici_septicae_MFLU_18_2588

NNNNNNNNNNNNNNNNNNNNNNNNNNNNNNNNNNNNNNNNNNNNNNNNNNNNNNNNNNNNNNNNNNNNNNNNNNNNNNNNNNNNNNNNNNNNNNNNNNNNNNNNNNNNNNNNNNNNNNNNNNNNNNNNNNNNNNNNNNNNNNNNNNNNNNNNNNNNNNNNNNNNNNNNNNNNNNNNNNNNNNNNNNNNNNNNNNNNNNNNNNNNNNNNNNNNNNNNNNNNNNNNNNNNNNNNNNNNNNNNNNNNNNNNNNNNNNNNNNNNNNNNNNNNNNNTTTCCCAGGGTAACCAAATCGGTGCTGCTTTCTGGTGCGTC------------------------CCAG--CTCCAG---CTCAGAGT-------------------------------CGACCACCTCG-ACTCTCGAC-------GCGCGACA-ACACTAGCTCGCATCAACG-TT----ACTGACCTCGGCTCTTTAGGCAAACCATCTCTGGCGAGCACGGTCTCGACAGCAATGGCGTGTATGCACCTCCTATTCCC--TGCCCA-CTG-----ATCTCGTC--CTCTCCCCC---GGCCTGGCACTGACGATTGTACAGTTACAACGGCACTTCCGAGCTCCAGCTCGAGCGCATGAACGTCTACTTCAACGAGGTAAGT----CAACA-GCCACGTCATC------------------AA-TACAAT-TTTGT-C----------CATCTACG-GC-ATGG-TCTCCTGCAGCCGC----CAAG----GTCTTGCTAACGCG-TTATCGCTC-AGGCCTCCGGCAACAAGTATGTGCCTCGCGCCGTCCTCGTCGATCTCGAGCCCGGTACCATGGACGCCGTC

>Diaporthe_machili_SAUCC194_111

NNNNNNNNNNNNNNNNNNNNNNNNNNNNNNNNNNNNNNNNNNNNNNNNNNNNNNNNNNNNNNNNNNNNNNNNNNNNNNNNNNNNNNNNNNNNNNNNNNNNNNNNNNNNNNNNNNNNNNNNNNNNNNNNNNNNNNNNNNNNNNNNNNNNNNNNNNNNNNNNNNNNNNNNNNNNNNNNNNNNNNNNNNNNNNNNNNNNNNNNNNNNNNNNNNNNNNNNNNNNNNNNNNNNNNNNNNNNNNNNNNNNNNNNNNNNNNNNNNNNNNNNNNNNNNNNNNNNNNNNNNNNNNNNNNNNNNNNNNNNNNNNNNNNNNNNNNNNNNNNNNNNNNNNNNNNNNNNNNNNNNNNCTCA---GCTCGAGC-------------------------------CTGCCACCGCG-ACGCTCGAC-------GCGCGACA-AGACTAGCTCGCAACATCT-TT----ACTGACCTCGTCTCTTTAGGCAAACCATCTCTGGCGAGCACGGTCTCGACAGCAATGGCGTGTATGTACCTCCTATTCCC--TGCCCA-CCG-----ATCTCGTC--CTCTCCTCC---GGCTTGGCACTGATGATCGCACAGTTACAACGGCACTTCCGAGCTCCAGCTCGAGCGCATGAACGTCTACTTCAACGAGGTAAGC----CTATG-GCCACGGCTCC------------------AA-TCCAAA-TTTGA-C----------CGTCT-CG-GC-ATGG-TTTACTGCCGCCGC----CAGG----GCCTTGCTAATGCG-CTCTCGCCC-AGGCCTCCGGCAACAAGTATGTGCCTCGCGCCGTCCTCGTCGATCTCGAGCCCGGTACCATGGACGCCGTC

>Diaporthe_neoraonikayaporum_MFLUCC_14_1136

NNNNNNNNNNNNNNNNNNNNNNNNNNNNNNNNNNNNNNNNNNNNNNNNNNNNNNNNNNNNNNNNNNNNNNNNNNNNNNNNNNNNNNNNNNNNNNNNNNNNNNNNNNNNNNNNNNNNNNNNNNNNNNNNNNNNNNNNNNNNNNNNNNNNNNNNNNNNNNNNNNNNNNNNNNNNNNNNNNNNNNNNNNNNNNNNNNNNNNNNNNNNNNNNNNNNNNNNNNNNNNNNNNNNNNNNNNNNNNNNNNNNNNNNNNNNNNNNNNNNNNNNNNNNNNNNNNNNNNNNNNNNNNNNNNNNNNNNNNNNNNNNNNNNNNNNNNNNNNNNNNNNNNNNNNNNNNNNNNNNNNNNNNNNNNNNNNNNNNT-------------------------------CTACCACCGCG-ACGCTTGAC-------TCGCGACA-AGACTAGTTAGCAGCTTCG-TT----ACTGACCTCGGCTC-TTAGGCAAACCATCTCTGGCGAGCACGGCCTCGACAGCAATGGCGTGTATGCACCTCCTATTCCC--TGCCAA-ATG-----ATATCGTC--CTCTCTTCC---GGCTTGGCACTGACAATTGCATAGTTACAACGGCACTTCCGAGCTGCAGCTCGAGCGCATGAATGTCTACTTCAACGAGGTCAGT----AAACA-GCTACATCGTG------------------AA-TCCAGA-TTTGG-C----------CATCTACC-AC-ATTG-TATCCTGCCGCCGA----CAAG----GCTTCGCTAACGTG-TTATCGCCC-AGGCCTCCGGCAACAAGTATGTGCCTCGCGCCGTCCTCGTCGATCTCGAGCCCGGTACCATGGACGCCGTC

>Diaporthe_chinensis_MFLUCC_19_0101

NNNNNNNNNNNNNNNNNNNNNNNNNNNNNNNNNNNNNNNNNNNNNNNNNNNNNNNNNNNNNNNNNNNNNNNNNNNNNNNNNNNNNNNNNNNNNNNNNNNNNNNNNNNNNNNNNNNNNNNNNNNNNNNNNNNNNNNNNNNNNNNNNNNNNNNNNNNNNNNNNNNNNNNNNNNNNNNNNNNNNNNNNNNNNNNNNNNNNNNNNNNNNNNNNNNNNNNNNNNNNNNNNNNNNNNNNNNNNNNNNNNNNNNNNNNNNNNNNNNNNNNNNNNNNNNNNNNNNNNNNNNNNNNNNNNNNNNNNNNNNNNNNNNNNNNNNNNNNNNNNNNNNNNNNNNNNNNNNNNNNNNNNNNNNNNNNNNNNNC-------------------------------CTACCATCGCG-ACTCTCGAC-------GCACGACA-ACACTAGCTCGCATCATTG-TT----ACTGACCTCTGCTCTTTAGGCAAACCATCTCTGGCGAGCACGGCCTCGACAGCAATGGCGTGTATGTACCTCCTATTCCC--TGCCCA-CTG-----ATCTCGTC--CTGTTCCCC---CGCTTAGCACTGACAATTGCATAGTTACAACGGCACTTCCGAGCTCCAGCTCGAGCGCATGAACGTCTACTTCAACGAGGTCAGT----AAACA-GCCACGTCGCC------------------AA-TTCAAA-TCTGA-C----------CATCTACG-AC-ATGG-TTCCATGTCGCCGC----CAAG----GTCTTGCTAACGCG-TTATCGCCC-AGGCCTCCGGCAACAAGTATGTGCCTCGCGCCGTCCTCGTCGATCTCGAGCCCGGTACCATGGATGCCGTC

>Diaporthe_guttulata_CGMCC_3_20100

NNNNNNNNNNNNNNNNNNNNNNNNNNNNNNNNNNNNNNNNNNNNNNNNNNNNNNNNNNNNNNNNNNNNNNNNNNNNNNNNNNNNNNNNNNNNNNNNNNNNNNNNNNNNNNNNNNNNNNNNNNNNNNNNNNNNNNNNNNNNNNNNNNNNNNNNNNNNNNNNNNNNNNNNNNNNNNNNNNNNNNNNNNNNNNNNNNNNNNNNNNNNNNNNNNNNNNNNNNNNNNNNNNNNNNNNNNNNNNNNNNNNNNNNNNNNNNNNNNNNNNNNNNNNNNNNNNNNNNNNNNNNNNNNNNNNNNNNNNNNNNNNNNNNNNNNNNNNNNNNNNNNNNNNNNNNNNNNNNNNNNNNNNNNNNNNNNNNNNNNNNNNNNNNNNNNNNNNNNNNNNNCAGCCTGCCACGACCGCG-ACATTCGAC-------ACGCGACA-AGATTAGCTCGCAACATCT-TTAT--ACTGACCTCGGCTGTTTAGGCAAACCATCTCTGGCGAGCACGGTCTCGACAGCAATGGCGTGTATGCACCTCCTATTCCCC-TGCCCA-CTG-----ATCTCGTC--CTCCCCTCC---GGCTTGGCACTGATGTTTGCACAGTTACAACGGCACTTCTGAGCTCCAGCTCGAGCGCATGAACGTCTACTTCAACGAGGTGAGT----CAAAATGCCACGTCTTC------------------AA-TTCAAG-TTTGA-G----------CCTTCTCG-GC-ATGA-TTTCCTGCCGCCGC----AAAAG---ACCTTACTGACGCG-CTTTCGCCC-AGGCCTCCGGCAACAAGTATGTGCCTCGCGCCGTCCTCGTCGATCTCGAGCCCGGTACCATGGACGCCGTC

>Diaporthe_pachirae_CDA_728

NNNNNNNNNNNNNNNNNNNNNNNNNNNNNNNNNNNNNNNNNNNNNNNNNNNNNNNNNNNNNNNNNNNNNNNNNNNNNNNNNNNNNNNNNNNNNNNNNNNNNNNNNNNNNNNNNNNNNNNNNNNNNNNNNNNNNNNNNNNNNNNNNNNNNNNNNNNNNNNNNNNNNNNNNNNNNNNNNNNNNNNNNNNNNNNNNNNNNNNNNNNNNNNNNNNNNNNNNNNNNNNNNNNNNNNNNNNNNNNNNNNNNNNNNNNNNNNNNNNNNNNNNNNNNNNNNNNNNNNNNNNNNNNNNNNNNNNNNNNNNNNNNNNNNNNNNNNNNNNNNNNNNNNNNNNNNNNNNNNNNNNNNNNNNNNNNNNNNNNNNNNNNNNNNNNNNNNNNNNNNNNNNNNNNNNNNNNNCCGCG-ATGCTCGAC-------GCGCGACA-AGACCACCTCCAAGCATCG-TT----ACTGACCTTGTTTCTTTAGGCAAACCATCTCTGGCGAGCACGGTCTCGACAGCAATGGCGTGTATGTACCTCCTATTCCC--TGCCCA-CCG-----ATCTCGTC--CTCTCCTCC---GGCTTGGCACTGATGATCGCACAGTTACAACGGCACTTCCGAGCTCCAGCTCGAGCGCATGAACGTCTACTTCAACGAGGTAAGC----CTATG-GCCACGTCTTC------------------AA-TCCAAG-TTTGA-C----------CGTCT-CG-GC-ATGG-TTTACTGCCGCCGC----CAGG----GCCTTGCTAATGCG-CTCTCGCCC-AGGCCTCCGGCAACAAGTATGTGCCTCGCGCCGTCCTCGTCGATCTCGAGCCCGGTACCATGGACGCCGTC

>Diaporthe_biguttulata_ICMP20657

NNNNNNNNNNNNNNNNNNNNNNNNNNNNNNNNNNNNNNNNNNNNNNNNNNNNNNNNNNNNNNNNNNNNNNNNNNNNNNNNNNNNNNNNNNNNNNNNNNNNNNNNNNNNNNNNNNNNNNNNNNNNNNNNNNNNNNNNNNNNNNNNNNNNNNNNNNNNNNNNNNNNNNNNNNNNNNNNNNNNNNNNNNNNNNNNNNNNNNNNNNNNNNNNNNNNNNNNNNNNNNNNNNNNNNNNNNNNNNNNNNNNNNNNNNNNNNNNNNNNNNNNNNNNNNNNNNNNNNNNNNNNNNNNNNNNNNNNNNNNNNNNNNNNNNNNNNNNNNNNNNNNNNNNNNNNNNNNNNNNNNNNNNNNNNNNNNNNNNNNNNNNNNNNNNNNNNNNNNNNNNNNNNNNNNNNNNNNNNNNNNNNNNNNNNNNNNNNNNNNNNNNNNNNNNNNAGCTCGCAGCATCG-TT----ACTGACATCTGCTTTC-AGGCAAACCATCTCTGGCGAGCACGGCCTCGACAGCAATGGCGTGTATGCACCTCCTATTCCC--TGCCCA-TTG-----ATCTCGTC--CTCTCCTCC---GGCTTGGCACTGACAATTGCACAGTTACAACGGCACTTCCGAGCTCCAGCTCGAGCGCATGAACGTCTACTTCAACGAGGTAAGT----CAACA-GCCACGTCGTC------------------AA-TTCGAG-TCTAT-C----------CATCTACG-GC-ATGG-TTTCCTGCCGCCGC----CAAG----GTCTTGCTAACGCG-TTATCTCCC-AGGCCTCCGGCAACAAGTATGTGCCTCGCGCTGTCCTCGTCGATCTCGAGCCCGGTACCATGGACGCCGTC

>Diaporthe_siamensis_MFLUCC_10_0573a

NNNNNNNNNNNNNNNNNNNNNNNNNNNNNNNNNNNNNNNNNNNNNNNNNNNNNNNNNNNNNNNNNNNNNNNNNNNNNNNNNNNNNNNNNNNNNNNNNNNNNNNNNNNNNNNNNNNNNNNNNNNNNNNNNNNNNNNNNNNNNNNNNNNNNNNNNNNNNNNNNNNNNNNNNNNNNNNNNNNNNNNNNNNNNNNNNNNNNNNNNNNNNNNNNNNNNNNNNNNNNNNNNNNNNNNNNNNNNNNNNNNNNNNNNNNNNNNNNNNNNNNNNNNNNNNNNNNNNNNNNNNNNNNNNNNNNNNNNNNNNNNNNNNNNNNNNNNNNNNNNNNNNNNNNNNNNNNNNNNNNNNNNNNNNNNNNNNNNNNNNNNNNNNNNNNNNNNNNNNNNNNNNNNNNNNNNNNNNNNNNNNNNNNNNNNNNNNNNNNNNNNNNNNNNNNNAGCTGGCATCATCG-TT----ACTGACCTCTGCTCTTTAGGCAAACCATCTCTGGCGAGCACGGCCTCGACAGCAATGGCGTGTATGCACCTCCTATTCCC--TGCCCA-TTG-----ATCTCGCC--CTCTCCTCC---GGCTCGGCACTGACAATTGCACAGTTACAACGGCACTTCTGAGCTCCAGCTCGAGCGCATGAATGTCTACTTCAACGAGGTAAGT----CAACA-GCCACGTCGCC------------------AA-TTCAAA-TCTGA-C----------CATCTACG-GC-ATGG-TTCCATGCCGCCGC----CAAG----GTCTTGCTAACACA-TTATCGCCC-AGGCTTCCGGCAACAAGTATGTGCCTCGCGCCGTCCTCGTCGATCTCGAGCCCGGTACCATGGACGCCGTC

>Diaporthe_discoidispora_ICMP20662

NNNNNNNNNNNNNNNNNNNNNNNNNNNNNNNNNNNNNNNNNNNNNNNNNNNNNNNNNNNNNNNNNNNNNNNNNNNNNNNNNNNNNNNNNNNNNNNNNNNNNNNNNNNNNNNNNNNNNNNNNNNNNNNNNNNNNNNNNNNNNNNNNNNNNNNNNNNNNNNNNNNNNNNNNNNNNNNNNNNNNNNNNNNNNNNNNNNNNNNNNNNNNNNNNNNNNNNNNNNNNNNNNNNNNNNNNNNNNNNNNNNNNNNNNNNNNNNNNNNNNNNNNNNNNNNNNNNNNNNNNNNNNNNNNNNNNNNNNNNNNNNNNNNNNNNNNNNNNNNNNNNNNNNNNNNNNNNNNNNNNNNNNNNNNNNNNNNNNNNNNNNNNNNNNNNNNNNNNNNNNNNNNNNNNNNNNNNNNNNNNNNNNNNNNNNNNNNNNNNNNNNNNNNNNNNNAGCTCGCAACATCG-TT----ACTGACCTAGACTCTTTAGGCAAACCATCTCTGGCGAGCACGGTCTCGACAGCAATGGCGTGTATGCAACTCCTATTCCC--TGCCCA-CTG-----ATATCGTC--CTCTCCTCC---GGCCTGGCACTGACAATTGCACAGTTACAACGGCACTTCCGAGCTCCAGCTCGAGCGCATGAACGTCTACTTCAACGAGGTAAGT----CAATA-GCCACGTCGTC------------------AA-TACAAC-TTTGT-C----------CATCTACATGC-ATGG-TCTCCTGCCGCCGC----CGAG----GTCTTGCTAACGCG-TTACCGCCC-AGGCCTCCGGCAACAAGTATGTGCCTCGCGCCGTCCTCGTCGATCTCGAGCCCGGTACCATGGACGCCGTC

>Diaporthe_cerradensis_CMRP4331

NNNNNNNNNNNNNNNNNNNNNNNNNNNNNNNNNNNNNNNNNNNNNNNNNNNNNNNNNNNNNNNNNNNNNNNNNNNNNNNNNNNNNNNNNNNNNNNNNNNNNNNNNNNNNNNNNNNNNNNNNNNNNNNNNNNNNNNNNNNNNNNNNNNNNNNNNNNNNNNNNNNNNNNNNNNNNNNNNNNNNNNNNNNNNNNNNNNNNNNNNNNNNNNNNNNNNNNNNNNNNNNNNNNNNNNNNNNNNNNNNNNNNNNNNNNNNNNNNNNNNNNNNNNNNNNNNNNNNNNNNNNNNNNNNNNNNNNNNNNNNNNNNNNNNNNNNNNNNNNNNNNNNNNNNNNNNNNNNNNNNNNNNNNNNNNNNNNNNNNNNNNNNNNNNNNNNNNNNNNNNNNNNNNNNNCGACGACCGCG-ACGCTCGAC-------GCGCGACA-AGACTAGCTGGTAGCATTG-TT----ACTGACTTCGGCTC-TTAGGCAAACCATCTCTGGCGAGCACGGCCTCGACAGCAATGGCGTGTATGCACCTCCTATCCCC--TGTCCG-ATG-----TCCTCGTC--CTCTCCTCC---GGCTTGGCACTGATGATCGCACAGTTACAACGGCACTTCCGAGCTCCAGCTCGAGCGCATGAACGTCTACTTCAACGAGGTCAGT----CAACA-GCCACGTCGTA------------------AA-TTCGGA-TTTGA-T----------CGTCTACG-GC-ATGG-TTTCCTGCCGCCGC----CAGG----GCCTTGCTAACGCG-TTATCGCCC-AGGCTTCCGGCAACAAGTATGTGCCTCGCGCCGTCCTCGTCGATCTCGAGCCCGGTACCATGGACGCCGTC

>Diaporthe_cichorii_MFLUCC_17_1023

NNNNNNNNNNNNNNNNNNNNNNNNNNNNNNNNNNNNNNNNNNNNNNNNNNNNNNNNNNNNNNNNNNNNNNNNNNNNNNNNNNNNNNNNNNNNNNNNNNNNNNNNNNNNNNNNNNNNNNNNNNNNNNNNNNNNNNNNNNNNNNNNNNNNNNNNNNNNNNNNNNNNNNNNNNNNNNNNNNNNNNNNNNNNNNNNNNNNNNNNNNNNNNNNNNNNNNNNNNNNNNNNNNNNNNNNNNNNNNNNNNNNNNNNNNNNNNNNNNNNNNNNNNNNNNNNNNNNNNNNNNNNNNNNNNNNNNNNNNNNNNNNNNNNNNNNNNNNNNNNNNNNNNNNNNNNNNNNNNNNNNNNNNNNNNNNNNNNNNNNNNNNNNNNNNNNNNNNNNNNNNNNNNNNNNNNACGACCGCG-ACATTCGAC-------ACGCGACA-AGATTAGCTCGCAACGTCG-TTAT--ACTGACCTCGGCTGTTTAGGCAAACCATCTCTGGCGAGCACGGTCTCGACAGCAATGGCGTGTATGCACCTCCTGTTTCCC-TGCCCA-CTG-----ATCTCGTC--CTTCCCTCC---GGCTTGGCACTGATGTTTGCACAGTTACAACGGCACTTCTGAGCTCCAGCTCGAGCGCATGAACGTCTACTTCAACGAGGTGAGT----CAAAA-GCCACGTCTTC------------------AA-TTCGAG-TTTGA-G----------CCTCCTCG-GC-ATGA-TTTCCTGCCGCCGC----AAAAAG--ACCTTACTGACGCG-CTTTCGCCC-AGGCCTCCGGCAACAAGTATGTGCCTCGCGCCGTCCTCGTCGATCTCGAGCCCGGTACCATGGACGCCGTC

>Diaporthe_cuppatea_CBS_117499

NNNNNNNNNNNNNNNNNNNNNNNNNNNNNNNNNNNNNNNNNNNNNNNNNNNNNNNNNNNNNNNNNNNNNNNNNNNNNNNNNNNNNNNNNNNNNNNNNNNNNNNNNNNNNNNNNNNNNNNNNNNNNNNNNNNNNNNNNNNNNNNNNNNNNNNNNNNNNNNNNNNNNNNNNNNNNNNNNNNNNNNNNNNNNNNNNNNNNNNNNNNNNNNNNNNNNNNNNNNNNNNNNNNNNNNNNNNNNNNNNNNNNNNNNNNNNNNNNNNNNNNNNNNNNNNNNNNNNNNNNNNNNNNNNNNNNNNNNNNNNNNNNNNNNNNNNNNNNNNNNNNNNNNNNNNNNNNNNNNNNNNNNNNNNNNNNNNNNNNNNNNNNNNNNNNNNNNNNNNNNNNNNNNNNNNNNNNNNNNNNNNNNNNNNNNNNNNNNNNNNNNNNNNNNNNNAGCTCGCAATATCG-TTAT--GCTGACCTCGGCTGTTTAGGCAAACCATCTCTGGCGAGCACGGTCTCGACAGCAATGGCGTGTATGCACCTCCTATTCCC--TGTCCT-CTG-----ATCTCGTC--CTCCCCTCC---GGCTTGGCACTGATGTTTGCACAGTTACAACGGCACTTCCGAGCTCCAGCTCGAGCGCATGAACGTCTACTTCAACGAGGTGAGT----CAAAA-GCCACGTCTTC------------------GA-TTCAAA-TTGGA-G----------CGTTCTCG-GC-ATGA-TTTACTGCCGCCGC----AAAG----ACTCTGCTAACGCG-CTTTCGCCC-AGGCCTCCGGCAACAAGTATGTGCCTCGCGCCGTCCTCGTCGATCTCGAGCCCGGTACCATGGACGCCGTC

>Diaporthe_compacta_LC3083

NNNNNNNNNNNNNNNNNNNNNNNNNNNNNNNNNNNNNNNNNNNNNNNNNNNNNNNNNNNNNNNNNNNNNNNNNNNNNNNNNNNNNNNNNNNNNNNNNNNNNNNNNNNNNNNNNNNNNNNNNNNNNNNNNNNNNNNNNNNNNNNNNNNNNNNNNNNNNNNNNNNNNNNNNNNNNNNNNNNNNNNNNNNNNNNNNNNNNNNNNNNNNNNNNNNNNNNNNNNNNNNNNNNNNNNNNNNNNNNNNNNNNNNNNNNNNNNNNNNNNNNNNNNNNNNNNNNNNNNNNNNNNNNNNNNNNNNNNNNNNNNNNNNNNNNNNNNNNNNNNNNNNNNNNNNNNNNNNNNNNNNNNNNNNNNNNNNNNNNNNNNNNNNNNNNNNNNNNNNNNNNNNNNNNNNNNNNNNNNNNNNNNNNNNNNNNNNNNNNNNNNNNNNNNNNNAGCTCGCAACATTG-TT----ACTGACCTCGGCTCTGTAGGCAAACCATCTCTGGCGAGCACGGCCTCGACAGCAATGGCGTGTATGCACCTCCTATTCCC--TGCCCA-CTG-----ATCCCGTC--CTGTCCTCC---GGCTTGGCACTGATGATTGCACAGTTACAACGGCACTTCCGAGCTCCAGCTCGAGCGCATGAACGTCTACTTCAACGAGGTATGT----CAATG-GCCACGTCGTC------------------AA-TCCAGA-TTTGC-C----------CATCTACT-GC-GCCG-CGTGCTGCCGTAGC----CAAA----GCCTTGCTAACGCG-TTGTCGCCC-AGGCTTCCGGCAACAAGTATGTGCCCCGCGCTGTCCTCGTCGATCTCGAGCCCGGTACCATGGACGCCGTC

>Diaporthe_schoeni_MFLU_15_1279

NNNNNNNNNNNNNNNNNNNNNNNNNNNNNNNNNNNNNNNNNNNNNNNNNNNNNNNNNNNNNNNNNNNNNNNNNNNNNNNNNNNNNNNNNNNNNNNNNNNNNNNNNNNNNNNNNNNNNNNNNNNNNNNNNNNNNNNNNNNNNNNNNNNNNNNNNNNNNNNNNNNNNNNNNNNNNNNNNNNNNNNNNNNNNNNNNNNNNNNNNNNNNNNNNNNNNNNNNNNNNNNNNNNNNNNNNNNNNNNNNNNNNNNNNNNNNNNNNNNNNNNNNNNNNNNNNNNNNNNNNNNNNNNNNNNNNNNNNNNNNNNNNNNNNNNNNNNNNNNNNNNNNNNNNNNNNNNNNNNNNNNNNNNNNNNNNNNNNNNNNNNNNNNNNNNNNNNNNNNNNNNNNNNNNNNNNNNATCGCG---GCTCGAC-------GCGCGATG-ACAGGACCTCGCAACATCG-TT----ATTGACTTCGACT-TTTAGGCAAACCATCTCTGGCGAGCACGGCCTCGACAGCAATGGCGTGTATGTACCTCCCATTCCC--TACTCG-TCG-----GTCTCGTC-------CGCC---GGCTTGGCACTGACAGCTTCACAGTTACAACGGCTCTTCTGAGCTCCAGCTCGAGCGCATGAACGTCTACTTCAACGAGGTCAGT----CCTCG-ATATTTTTATT------------------GC--------ACCCA-C----------GATCTCCA-AA-ATTG-CCTTGTGTTGTCGT----TTGG----ACTTTGCTGACACC-TTATCGTCC-AGGCTTCCGGCAACAAGTATGTGCCTCGCGCTGTCCTCGTCGATCTCGAGCCCGGTACCATGGACGCCGTC

>Diaporthe_acericola_MFLUCC_17_0956

NNNNNNNNNNNNNNNNNNNNNNNNNNNNNNNNNNNNNNNNNNNNNNNNNNNNNNNNNNNNNNNNNNNNNNNNNNNNNNNNNNNNNNNNNNNNNNNNNNNNNNNNNNNNNNNNNNNNNNNNNNNNNNNNNNNNNNNNNNNNNNNNNNNNNNNNNNNNNNNNNNNNNNNNNNNNNNNNNNNNNNNNNNNNNNNNNNNNNNNNNNNNNNNNNNNNNNNNNNNNNNNNNNNNNNNNNNNNNNNNNNNNNNNNNNNNNNNNNNNNNNNNNNNNNNNNNNNNNNNNNNNNNNNNNNNNNNNNNNNNNNNNNNNNNNNNNNNNNNNNNNNNNNNNNNNNNNNNNNNNNNNNNNNNNNNNNNNNNNNNNNNNNNNNNNNNNNNNNNNNNNNNNNNNNNNNNNNATCGCG--GTCTCGAC-------GCGCGATG-ACAGGACCTCGCAACATCG-TT----ATTGACTTCGACT-TTTAGGCAAACCATCTCTGGCGAGCACGGCCTCGACAGCAATGGCGTGTATGTACCTCCCATTCCC--TACTCG-TCG-----GTCTCGTC-------CGCC---GGCTTGGCACTGACAGCTTCACAGTTACAACGGCTCTTCTGAGCTCCAGCTCGAGCGCATGAACGTCTACTTCAACGAGGTCAGT----CCTCG-ATATTTTTATT------------------GC--------ACCCA-C----------GATCTCCA-AA-ATTG-CCTTGTGTTGTCGT----TTGG----ACTTTGCTGACACC-TTATCGTCC-AGGCTTCCGGCAACAAGTATGTGCCTCGCGCTGTCCTCGTCGATCTCGAGCCCGGTACCATGGACGCCGTC

>Diaporthe_camporesii_JZB320143

NNNNNNNNNNNNNNNNNNNNNNNNNNNNNNNNNNNNNNNNNNNNNNNNNNNNNNNNNNNNNNNNNNNNNNNNNNNNNNNNNNNNNNNNNNNNNNNNNNNNNNNNNNNNNNNNNNNNNNNNNNNNNNNNNNNNNNNNNNNNNNNNNNNNNNNNNNNNNNNNNNNNNNNNNNNNNNNNNNNNNNNNNNNNNNNNNNNNNNNNNNNNNNNNNNNNNNNNNNNNNNNNNNNNNNNNNNNNNNNNNNNNNNNNNNNNNNNNNNNNNNNNNNNNNNNNNNNNNNNNNNNNNNNNNNNNNNNNNTATGAACATTAGCTC--------------------AGCTACAG--CTCCAG---CTCGAAGC-------------------------------CTACCGCCGCG-ATGCTCGAC-------GCGCGACA-AGACCAGCTCGCAACATTG-TT----ACTGACCTCGGCTCCGTAGGCAAACCATCTCTGGCGAGCACGGCCTCGACAGCAATGGCGTGTATGCACCTCCTATCCCC--TGCCCA-CTG-----ATCTCGTC--CTGTCCTCC---GGCCTGGCACTGATGATTGCACAGCTACAACGGCACTTCCGAGCTCCAGCTCGAGCGCATGAACGTCTACTTCAACGAGGTATGT----CAATG-GCCACGTCGTC------------------AA-TCCAGA-TTTGC-C----------CATCTGCT-GC-ATGG-TGTGCTGCTGTAGC----CAAA----GCCCTCCTAACGCG-TTATCGCCC-AGGCTTCCGGCAACAAGTATGTGCCCCGCGCTGTCCTCGTCGACCTCGAGCCCGGTACCATGGACGCCGTC

>Diaporthe_cucurbitae_DAOM_42078

NNNNNNNNNNNNNNNNNNNNNNNNNNNNNNNNNNNNNNNNNNNNNNNNNNNNNNNNNNNNNNNNNNNNNNNNNNNNNNNNNNNNNNNNNNNNNNNNNNNNNNNNNNNNNNNNNNNNNNNNNNNNNNNNNNNNNNNNNNNNNNNNNNNNNNNNNNNNNNNNNNNNNNNNNNNNNNNNNNNNNNNNNNNNNNNNNNNNNNNNNNNNNNNNNNNNNNNNNNNNNNNNNNNNNNNNNNNNNNNNNNNNNNNNNNNNNNNNNNNNNNNNNNNNNNNNNNNNNNNNNNNNNNNNNNNNNNNNNNNNNNNNNGTGCGTC------------------------CCAG--CTCCAG---CTCCAGCTCCAGTTCCAGCTCAAGCT----CCAAGCCTGCCACGGCCGCG-ACATTCGAC-------ACGCGACA-AGATTAGCTCGCAACACCT-TTAT--ACTGACCTCGGCTGTTTAGGCAAACCATCTCTGGCGAGCACGGTCTCGACAGCAATGGCGTGTATGCACCTCCTATTTCCC-TGCCCA-CTG-----ATCTCGTC--CTCCCCTCC---GGCTTGGCATTGATGTTTGCACAGTTACAACGGCACTTCTGAGCTTCAGCTCGAGCGCATGAACGTCTACTTCAACGAGGTGAGT----CAAAATGCCACGTCTTC------------------AA-TTCAAG-TTTGA-G----------CGTTCTTG-GC-ATGA-TTTCCTGCCGCCGC----AAAGA---CCTTTACTGACGCG-CTTTCGCCC-AGGCCTCCGGCAACAAGTATGTGCCTCGCGCCGTCCTCGTCGATCTCGAGCCCGGTACCATGGACGCCGTC

>Diaporthe_sambucusii_CFCC_51986

GTCTGC-CCATGC--TGCTCTCGCA-TCCTCCTCTGC----CCCTGAGCCTCAGCC----TACCCCACCATCGCGACCACACTCCCACAGCAGGGCCTC-AAAACATCACCAGCACCTTGCGATGAGCACCCGTGTG-CCCTTGGAACACGCGTCAGATTGCTAACATGGACTTT-TTCTCGCCTGCAGGTTCACCTCCAGACCGGCCAATGCGTAAGTTGCTCCCTGTCAAC-ACTG-CCCGACCTTATCGCCA-CCCGTAGCTGACACGTTTCCCAGGGTAACCAAATCGGTGCTGCTTTCTGGTGCGTC------------CCAGCTCCAGCTCCAG--CTCCAG---CTCCAAGC-------------------------------CTACCGGCGCG-ATGCTCGAC-------GCGCGACA-AGACCAGCTCGCAACATTG-TT----ACTGACCTCGGCTCTGTAGGCAAACCATCTCTGGCGAGCACGGCCTCGACAGCAATGGCGTGTATGCACCTCCTATTCCC--TGCCCA-CTG-----ATCTCGTC--CTGTCCTCC---GGCTTGACACTGATGATTGCACAGTTACAACGGCACTTCCGAGCTCCAGCTCGAGCGCATGAACGTCTACTTCAACGAGGTATGT----CAATG-GCCACGTCGTC------------------AA-TCCAAA-TTTGC-C----------CATCTACT-GC-GCGG-CGTGCTGCCGTAGC----CAAA----GCCTTGCTAACGCG-TTGTCGCCC-AGGCTTCCGGCAACAAGTATGTGCCCCGCGCTGTCCTCGTCGATCTCGAGCCCGGTACCATGGACGCCGTC

>Diaporthe_manihotia_CBS_505_76

GTCGGC-CCATGC--TGCTCTCGCA-CCCTCCTCTGC----CCCTGAACCTCAGCC----TACCCCACCATCGCGACCACGCTCCCACAGCAGGGCCTC-AAAACACCACCAGCACCTTGCGATGAGCACCCGCGTG-CCCTTGGAACACGCGTCAGATTGCTAACATGGACTTT-TTCTCGCCTGCAGGTTCACCTCCAGACCGGCCAATGCGTAAGTTGCTCCCTGTCAAC-ACCG-CCCGACCTTATCGCCA-CCCGTAGCTGACACGTTTCCCAGGGTAACCAAATCGGTGCTGCTTTCTGGTGCGTC------------CCAGCTCCAGCTCCAG--CTCCAG---CTCGAGGC-------------------------------CTACCGCCGCG-ATGCTCGAC-------GCGCGACA-AGACCAGCTCGCAACATTG-TT----ACTGACCTCGGCTCCGTAGGCAAACCATCTCTGGCGAGCACGGCCTCGACAGCAATGGCGTGTATGCACCTCCTATTCCC--TGCCCA-CCG-----ATCTCGTC--CTGTCCTCC---GGCTTGGCACTGATGATCGCACAGTTACAACGGCACTTCTGAGCTCCAGCTCGAGCGCATGAACGTCTACTTCAACGAGGTATGT----CAATG-GCCACGTCGTC------------------AA-TCCAAA-TTTGC-C----------CATCTGCT-GC-ATTG-TGTGCTGCCGTAGC----CAAA----GCCTTGCTAACGCG-TTATCGCCC-AGGCTTCCGGCAACAAGTATGTGCCCCGCGCTGTCCTCGTCGATCTCGAGCCCGGTACCATGGACGCCGTC

>Diaporthe_ganjae_CBS_180_91

GTCGGC-CCATC---TGCTCTCGCA-TCCTCCTCTGC----CCCTGAGCCTCAGCC----TACCCCACCATCGCGACCACACTCCCACAGCAGGGCCTC-AAAACATCACCAGCACCTTGTGATGAGCACCCGTGTG-CCCTTGGAACACGCGTCAGATTGCTAACATGGACTTT-TTCTGGCCTGCAGGTTCACCTCCAGACCGGCCAATGCGTAAGTTGCTCCCTGTCAAC-ACCG-CCCGACCTTATCGCCA-CCCGTAGCTGACACGTTTCCCAGGGTAACCAAATCGGTGCTGCTTTCTGGTGCGTCCCAGCTCCAGCTCCAGCTCCAGCTCCAG--CTCCAG---CTCGAGGC-------------------------------CTACCGCCGCG-ATGCTCGAC-------GCGCGACA-AGACCAGCTCGCAACATTG-TT----ACTGACCTAGGCTCTGTAGGCAAACCATCTCTGGCGAGCACGGTCTCGACAGCAATGGCGTGTATGCACCTCCTATTCCC--TGCCCA-CTG-----ATCTCGTC--CTGTCCTCC---GGCTTGGCACTGATGATTGCACAGTTACAACGGCACTTCCGAGCTCCAGCTCGAGCGCATGAACGTCTACTTCAACGAGGTATGT----CAATG-GCCACGTCGTC------------------AA-TCCAAA-TTTGC-C----------CATCTACT-GC-GCGG-CGTGCTGCCGTAGC----CAAA----GCCTTGCTAACGCG-TTGTCGCCC-AGGCTTCCGGCAACAAGTATGTGCCCCGCGCTGTCCTCGTCGATCTCGAGCCCGGTACCATGGACGCCGTC

>Diaporthe_raonikayaporum_CBS_133182

GTCGGC-CCATGC--TGCTTTCGCA-TCCTCCTCTGC----CCCTGAGCCTCAGAC----TACCCCACCATCGCGACCACACCCACAG--TCAGGCCTC-AAAACACCACCAAC---------AGAGCACCCAGATG-CCCTGAGAAGACGCGTCAGATCGCTAACATGGACTTT-TTCTCGCCCACAGGTTCACCTTCAGACCGGCCAATGCGTAAGTTGCTTCCCGTCAAC-ACCA-CTGGACCTTATCGCCA-CCTGTAGCTGACACGTTTCCCAGGGTAACCAAATCGGTGCTGCTTTCTGGTGTGTA------------------------CCAG--CTCCAG--CCCTCAAGC-------------------------------CTACCACCGCG-ACGCTTGAC-------TCGCGACA-AGACTAGTTAGCAGCTTCG-TT----ACTGACCTCGGCTC-TTAGGCAAACCATCTCTGGCGAGCACGGCCTCGACAGCAATGGCGTGTATGCACCTCCTATTCCC--TGCCAA-ATG-----ATATCGTC--CTCTCTTCC---GGCTTGGCACTGACAATTGCACAGTTACAACGGCACTTCCGAGCTGCAGCTCGAGCGCATGAATGTCTACTTCAACGAGGTCAGT----AAACA-GCTACATCGTG------------------AA-TCCAGA-TTTGG-C----------CATCTACC-AC-ATGG-TATCCTGCCGCCGA----CAAG----GCTTCGCTAACGTG-TTATCGCCC-AGGCCTCCGGCAACAAGTATGTGCCTCGCGCCGTCCTCGTCGATCTCGAGCCCGGTACCATGGACGCCGTC

>Diaporthe_angelicae_CBS_111592

GTCGGCCCTTTGCTGTGCTCTCGCA-TCCTCCTCTGC---CCCCTGAGCCTCAGGC----TACCCCACCATCGCGACCACACCCACGT--TCAGGCCTC-AAAACACCACCAGCACCCTGCGAAGTGCACCCAGATG-CCCTTGGAAGACGCGTCAGATTGCTAACATGGACTTTTCTCTCTCCTACAGGTTCACCTTCAGACCGGCCAATGCGTAAGTCGCCTCCTGTCAAC-ACCGCCGGGACCTTATCGCCA-CCCGTAGCTGACACGTTTCCCAGGGTAACCAAATCGGTGCTGCTTTCTGGTGCGTC------------------------CCAG--CTCCAG---CTCCAGCTCCAGCTCCAGCCCAAGCTCAACCAAAACCTGCCACGGCCGCG-ACATTCGAC-------ACGCGACA-AGATTAGCTCGCAACATCT-TTAT--ACTGACCTCGGCTGTTTAGGCAAACCATCTCTGGCGAGCACGGTCTCGACAGCAATGGCGTGTATGGACCTCCTATTCCCC-TGCCCA-CTG-----ATCTCGTC--CTCCCCTCC---GGCTTGGCACTGATGTTTGCACAGTTACAACGGCACTTCTGAGCTCCAGCTCGAGCGCATGAACGTCTACTTCAACGAGGTGAGT----CAAAATGCCACGTCTTC------------------AA-TTCAAG-TTTGA-G----------CCTTCTCG-GC-ATGA-TTTCCTGCCGCCGC----AAAAGA--CCTTTACTGACGCG-CTTTCGCCC-AGGCCTCCGGCAACAAGTATGTGCCCCGCGCCGTCCTCGTCGATCTCGAGCCCGGTACCATGGACGCCGTC

>Diaporthe_arctii_CBS_136_25

GTCGGCCCTTTGCTGTGCTCTCGCA-TCCTCCTCTGCTGCCCCCTGAGCCTCAGGC----TACCCCACCATCGCGACCACACCCACGT--TCAGGCCTC-AAAACACCACCAGCGCCCTGCGAAGTGCACCCAGATG-GCCTTGGAAGACGCGTCAGATTGCTAACATGGACTTTGTTCTCTCCTACAGGTTCACCTTCAGACCGGCCAATGCGTAAGTTGCCTCCTGTCAAC-ACCGCCGAGACCTTATCGCCA-CCCGTAACTGACACGTTTCCCAGGGTAACCAAATCGGTGCTGCTTTCTGGTGCGTC------------------------CCAG--CTCCAG---CTCCAGCTCCAGTTCCAGCTCAAGCT----CCAAGCCTGCCACGGCCGCG-ACATTCGAC-------ACGCGACA-AGATTAGCTCGCAACACCT-TTAT--ACTGACCTCGGCTGTTTAGGCAAACCATCTCTGGCGAGCACGGTCTCGACAGCAATGGCGTGTATGCACCTCCTATTCCCC-TGCCCA-CTG-----ATCTCGTC--CTCCCCTCC---GGCTTGGCACTGATGTTTGCACAGTTACAACGGCACTTCTGAGCTCCAGCTCGAGCGCATGAACGTCTACTTCAACGAGGTGAGT----CGAAATGCCACGTTTTC------------------AA-TTCAAG-TTTGA-G----------CCTTCTCG-GC-ATGA-TTTCCTGCCGCCGC----AAAGA---CCTTTACTGACGCG-CTTTCGCCC-AGGCCTCCGGCAACAAGTATGTGCCTCGCGCCGTCCTCGTCGATCTCGAGCCCGGTACCATGGACGCCGTC

>Diaporthe_gulyae_BRIP_54025

GTCGGCCCTTTGCTGTGCTCTCGCA-TCCTCCTCTGCTGCCCCCTGAGCCTCAGGC----TACCCCACCATCGCGACCACACCCACGT--TCAGGCCTC-AAAACACCACCAGCGCCCTGCGAAGTGCACCCAGATG-CCCTTGGAAGACGCGTCAGATTGCTAACATGGACTTCGTTCTCTCCTACAGGTTCACCTTCAGACCGGCCAATGCGTAAGTTGCCTCCTGTCAAC-ACCGCCGAGACCTTATCGCCA-CCCGTAACTGACACGTTTCCCAGGGTAACCAAATCGGTGCTGCTTTCTGGTGCGTC------------------------CCAG--CTCCAG---CTCCAGCTCCAGTTCCAGCTCAAGCT----CCAAGCCTGCCACGGCCGCG-ACATTCGAC-------ACGCGACA-AGATTAGCTCGCAACACCT-TTAT--ACTGACCTCGGCTGTTTAGGCAAACCATCTCTGGCGAGCACGGTCTCGACAGCAATGGCGTGTATGCACCTCCTATTCCCC-TGCCCA-CTG-----ATCTCGTC--CTCCCCTCC---GGCTTGGCACTGATGTTTGCACAGTTACAACGGCACTTCTGAGCTCCAGCTCGAGCGCATGAACGTCTACTTCAACGAGGTGAGT----CAAAATGCCACGTCTTC------------------AA-TTCGAG-TTTGA-G----------CGTTCTCG-GC-ATGA-TTTCCTGCCGCCCC----AAAGA---CCTTTACTGACGCG-CTTTCGCCC-AGGCCTCCGGCAACAAGTATGTGCCTCGCGCCGTCCTCGTCGATCTCGAGCCCGGTACCATGGACGCCGTC

>Diaporthe_subordinaria_CBS_101711

GTCGGCCCTTTGCTGTGCTCTCGCA-TCCTCCTCTGCTGCTCCCTGAGCCTCAGGCT---TACCCCACCATCGCGACCACACCCACGT--TCAGGCCTC-AAAACACCACCAGCGCCCTGCAAAGTGCACCCAGATG-CCCTTGGAAAACGCGTCAGATTGCTAACATGGACTTTGTTCTCTCCTACAGGTTCACCTTCAGACCGGCCAATGCGTAAGTTGCCTCCTGTCAAC-ACCGCCGGGACCTTATCGCCA-CCCGTAGCTGACACGTTTCCCAGGGTAACCAAATCGGTGCTGCTTTCTGGTGCGTC------------------------CCAG--CTCCAG---CTCCAGCTCCAGCCCAAGCT----------CCAAGCCTGCCACGGCCCGC-GACATCGAC-------ACGCGACA-AGATTAGCTCGCAACATCT-TTAT--ACTGACCTCGGCTGTTTAGGCAAACCATCTCTGGCGAGCACGGTCTCGACAGCAATGGCGTGTATGCACCTCCTATTCCCC-TGCCCA-CTA-----ATCTCGTC--CTCCCCTCC---GGCTTGGTACTGATGTTGGCACAGTTACAACGGCACTTCTGAGCTCCAGCTCGAGCGCATGAACGTCTACTTCAATGAGGTGAGT----CAAAATGCCACGTCTTC------------------AA-TTCAAA-TTTGA-G----------CCTTCTCG-GC-ATGA-TTTCCTGCCGCCGC----AAAGA---CCTTTACTGACGCG-CTTTCGCCC-AGGCCTCCGGCAACAAGTATGTGCCTCGCGCCGTCCTCGTCGATCTCGAGCCCGGTACCATGGACGCCGTC

>Diaporthe_novem_CBS_127271

GTCGGCCCTCTGCTGGGCTCTCGCATTCCTCCTCTGC----CCCTGAGCCTCAGGC----TACCCCACCATCGCGACCACACCCACGT--TCAGGCCTC-AAAACACCACCAGCACCCTGCGAAGTGCACCCAGATG-CCCTTGAAAGACGCGTCAGACTGCTAACATGGACTTT-TTCTCTCCTACAGGTTCACCTTCAGACCGGCCAATGCGTAAGTTGCCTCCTGTCAAC-ACCGCCGGGACCTTATCGCCA-CCCGTAGCTGACACGTTTCCCAGGGTAACCAAATCGGTGCTGCTTTCTGGTGCGTC------------------------CCAG--CTCCAG---CTCCAGCTCCAGCTCCAGCT----------TCAAGCCTGCCACGACCGCG-ACATTCGAC-------ACGCGACA-AGACGAGCTCGAAACATGA-TTAT--ACTGACCTCGGCTGTTTAGGCAAACCATCTCTGGCGAGCACGGTCTCGACAGCAATGGCGTGTATGCACCTCCTATTCCCC-TGCCCA-CTG-----ATCTCGTC--CTCCTCTCC---GGCTTGGCACTGATGTTTGCACAGTTACAACGGCACTTCTGAGCTCCAGCTCGAGCGCATGAACGTCTACTTCAACGAGGTAAGC----CAAACAGCCACGTCTTC------------------AA-TTCACA-TTTGA-G----------CGTTCTCG-GC-ATGG-ATTATTGCCGCCGC----AGAG----ACCTGACTGACGCG-CTTTCGCCT-AGGCCTCCGGCAACAAGTATGTGCCTCGCGCCGTCCTCGTCGATCTCGAGCCCGGTACCATGGACGCCGTC

>Diaporthe_lusitanicae_CBS_123212

GTCGGCCCTCTGCTGTGCTCTCGCA-TCCTCCTCTGC----CCCTGAGCCTCAGGC----GACCCCACCATCGCGACCACACCCACGG--TCAGGCCTC-AAAACACCACCAGCGCCCTGCGAAGTGCACCCAGATG-CCCTTGGAAGACGCGTCAGATTGCTAACATGGACTTTTTTCTCTCCTACAGGTTCACCTTCAGACCGGCCAATGCGTAAGTTGCCTCCTGTCAAC-ACC-GCCCGACCTTATCGCCA-CCCGTAGCTGACACGTTTCCCAGGGTAACCAAATCGGTGCTGCTTTCTGGTGCGTC------------------------CCAG--CTCGAG---CTCCAGCT----------------------CCAAGCCTGCCACGACCGCG-ACATTCGAC-------ACGCGACA-AGACTGGCTCGCAATATCC-TTAT--ACTGACCTCGGCTGTTTAGGCAAACCATCTCTGGCGAGCACGGTCTCGACAGCAATGGCGTGTATGCACCTCCTATTCCC--TGCCCT-TTG-----ATCTCATC--CTCCCCTCC---GGCTTGGCACTGATGTTTGCACAGTTACAACGGCACTTCCGAGCTCCAGCTCGAGCGCATGAACGTCTACTTCAACGAGGTGAGT----CAAAA-GCCACGTCTTC------------------AG-TTCAAA-TTTGA-G----------CGACCTCG-GC-ATGA-TTTACTGCCGCCGC----AAAG----ACTTTGCTAACGCG-CTTTCGCCC-AGGCCTCCGGCAACAAGTATGTGCCTCGCGCCGTCCTCGTCGATCTCGAGCCCGGTACCATGGACGCCGTC

>Diaporthe_neoarctii_CBS_109490

GTCGGCCCTTTGCTGTGCTCTCGCATTCCTCCTCTGC---CCCCTGAGCCTCAGGCTAC-TACCCCACCATCGCGACCACACCCACGG--TCAGGCCTC-AAAACACCACCAGCGCCCTGCAAAGTGCACCCAGATG-CCCTTGGAAGACGCGTCAGATTGCTAACATGGACCTTGTTCTCTCCTACAGGTTCACCTCCAGACCGGCCAATGCGTAAGTTGCCTCCTGTCAAC-ACCGCCGGGACCTTATCGCCA-CCCGTAGCTGACACGTTTCCCAGGGTAACCAAATCGGTGCTGCTTTCTGGTGCGTC------------------------CCAG--CTCCAG---CTCAAGCTTCAGCTCCAGCTCCAGCT----CTAAGCCTGCTACGACCGCG-ACATTCGAC-------ACGCGACA-GGAATAGCTCGCAATATCT-TTAT--ACTGACCTCGGCTGTTTAGGCAAACCATCTCTGGCGAGCACGGTCTCGACAGCAATGGCGTGTATGCACCTCCTATTCCCCTTGCCCA-CTG-----ATCTCGTCCTCTCCCCCCCTCGGGCCTAGCACTGATGTTTGCACAGTTACAACGGCACTTCTGAGCTCCAGCTCGAGCGCATGAACGTCTACTTCAACGAGGTGAGT----CAAAA-GCCACGTCTTC------------------AA-TTCAAG-TTTGA-G----------TGTTCTCG-GC-ATGA-TTTCCTGCTACCGCCGCAAAAAG---ACCTTACTGACACATCTTTCGCCC-AGGCCTCCGGCAACAAGTATGTGCCTCGCGCCGTCCTCGTCGATCTCGAGCCCGGTACCATGGACGCCGTC

>Diaporthe_sinensis_ZJUP0033_4

GTCGGCCCTCTGCTGTGCTCTCGCA----TCCTCTGC---CCCCTGAGCCTCGGGC----TACCCCACCATCGCGACCACACCCACAG--TCAGGCCTC-AAAACATCACGAGCGTCCTGCGAGCTGCAGCCCGATG-CCCTTGGAAGACGCGCCAGATTGCTAACATGGACTTTTCCCTCGCCCATAGGTTCACCTTCAGACCGGCCAATGCGTAAGTAACCTCCTGTCAAC-ACC-TTAGGACCTTGCCGCCA-CCCCTAGCTGACACGTTTCCCAGGGTAACCAAATCGGTGCTGCTTTCTGGTGCGTC------------------------CCAG--CTCCAG---CCCCAGCCTCAGCTCCAGCCCCCGCT----CCAAGCCTGCTACGACCGCG-ACATTCGAT-------ACGCGACA-AGACAAGCTCCCAGCATCG-TTTT--ACTGACCTCGGCTGTTTAGGCAAACCATCTCTGGCGAGCACGGTCTCGACAGTAATGGCGTGTATGCACCTCCTATTCCC-TGGCCCA-CTG-----ACCTCGTC--CTTCCCTCC---GGCATGGCACTGATGATGGCGCAGTTACAACGGCACTTCCGAGCTGCAGCTCGAGCGCATGAACGTCTACTTCAACGAGGTGAGT----CAAGAATCCACGTCTTC------------------AA-GTCAAA-ATTGA-G----------CGTTCTCG-GC-ATGG-TTTATTGCCGCACC----AAAG----ACCTCGCTAACGCG-CCTTCGCCC-AGGCCTCCGGCAACAAGTATGTGCCTCGCGCCGTCCTCGTCGACCTCGAGCCCGGTACCATGGACGCCGTC

>Diaporthe_batatas_CBS_122_21

GTCGGC-CCATGCTGTGCTCTCGCA-TCCTCCTCTGC----CCCTGAGCCTAAGGC----TACCCCACCATCGCGACCACACCCACCG--TCGGGCCTC-AAAACACCACCAGCTGCCTGCGAAAAGCACCCAGGTG-CCCTTGGAACACGCGTCAGATTGCTAACGTGACCTTT-TTCTCGCCCACAGGTTCACCTCCAGACCGGCCAATGCGTAAGTTGCCTCCTGTCAACAACC-GCCCGACCTTATCGCCA-CCCGTAGCTGACACGTTTCCCAGGGTAACCAAATCGGTGCTGCTTTCTGGTGCGTC------------------------GCAGCTCTCCAG---CTCCAAGTC------------------------------CTACCACCGCGACGACTCGAC-------ACGCGCGATAGGCGAGCTCGAAGCATCG-GT----ACTGACCTCGGCTGTTTAGGCAAACCATCTCTGGCGAGCACGGTCTCGACAGCAATGGCGTGTATGCACCTCCTAATCCC---------CTA-----CCCTCGTC--CTCTCCTCC---GCCTTGGCACTGACGATCGCACAGTTACAACGGCACTTCCGAGCTCCAGCTCGAGCGCATGAACGTCTACTTCAACGAGGTAAGT----CAACA-GCCACGTCGTC------------------AA-TCCAAA-CTTGA-C----------CGCCT-CG-GC-GTGG-TCAACTGCCGCCGC----CAAG----CCCTTGCTAACGCG-TTTTCACCC-AGGCCTCCGGCAACAAGTATGTTCCTCGCGCCGTCCTCGTCGATCTCGAGCCCGGTACCATGGACGCCGTC

>Diaporthe_convolvuli_CBS_124654

GTCGGC-CCATGCTGTGCTCTCACT----TCCTCTGC----CCCTGAGCATCAGGC----TACCCCACCATCGCGACCACACCCACGG--TCGGGCCTC-AAAACACCACCAGCTCCCTGCGACGAGCACCCAGATA-CCCTTGGAACACGCGTCAGATTGCTAACGTGACCTTT-TCCTCGCCCACAGGTTCACCTTCAGACCGGCCAATGCGTAAGTTGCTCCTCGTTAAC-ACC-GCCAGACCTTATCGCCA-CCCGTAGCTGACACGTTTCCCAGGGTAACCAAATCGGTGCTGCTTTCTGGTGCGTC------------------------GCAACTCTCCAG---CTCCAAAC-------------------------------CTACCACCGCG-ACGCTCGAC-------GCGCGATC-AGGCGAGCTCCAAGCATCG-GT----GCTGACCTCGGTTCTTTAGGCAAACCATCTCTGGCGAGCACGGTCTCGACAGCAATGGCGTGTATGCACCCCCTATTCCC--TGCCCA-CTG---------CCCA--GTCCTCTCC---GGCTTGGCACTGATGATCGCACAGTTACAACGGCACTTCCGAGCTCCAGCTCGAGCGCATGAACGTCTACTTCAACGAGGTATGT----CAATA-G-CACGTCGTC------------------AA-TTCAAATTTTGA-C----------CCTCT-CG-GC-ATGC-TTAACCGCCGCCGCC---CAAG----CCATTGCTAACGCG-TCTTCTCCC-AGGCTTCCGGCAACAAGTATGTGCCCCGCGCCGTCCTCGTCGATCTCGAGCCCGGTACCATGGACGCCGTC

>Diaporthe_miriciae_BRIP_54736j

GTCGGC-CCAAGCTGTGCTCTCGCA----TCCTCTGC----CCCTGAGCCTGAGGC----TACCCCACCATCGCGACCACACTCACGG--TCGGGCCTC-AAAACACCACCAGCTCCCTGCGAAGAGCACCCAGATG-CCCTTGGAACACGCGTCAGATTGCTAACATAGCCTTT-TTCTCGCCTACAGGTTCACCTTCAGACCGGCCAATGCGTAAGTTGCTCCCTGTCAAC-ACC-GCCGGACCTTATCGCCA-CCCGTAGCTGACACGTTTACCAGGGTAACCAAATCGGTGCTGCTTTCTGGTGCGTC------------------------GCAGCTCTCCAG---CTCCAAGC-------------------------------CTACCGCCGCGAACCCTCGAC-------GCGCGACA-AGGCGAGCTCGAAGCATCG-AT----ACTGACCTCGGTTCTTTAGGCAAACCATCTCCGGCGAGCACGGTCTCGACAGCAATGGCGTGTATGCACCTCCTATTCCC--TGCCCG-TGG-----CCCTCATC--CTCTTCTCC---GGCTTGGCACTGATGATCGCACAGTTACAACGGCACTTCCGAGCTCCAGCTCGAGCGCATGAACGTCTACTTCAACGAGGTCAGT----CAATA-GCCACGTCGTC------------------AA-TTCAAA-TTTGA-A----------CCTCT-CG-GC-ATGG-TCAACTGCCGCCGC----CAAG----CCCCTGCTAACGCG-TTTTCGCCC-AGGCCTCCGGCAACAAGTATGTGCCCCGCGCCGTCCTCGTCGATCTCGAGCCCGGTACCATGGACGCCGTC

>Diaporthe_passifloricola_CBS_141329

????????????????????????????????????????????????????????????????????????????????????????????????????????????????????????????????????????????????????????????????????????????????????????????????????????????????AATGCGTAAGTTGCTCCCTGTCAAC-ACC-GCCGGACCTTATCGCCA-CCCGTAGCTGACACGTTTACTAGGGTAACCAAATCGGTGCTGCTTTCTGGTGCGTC------------------------GCAGCTCTCCAG---CTCCAAGC-------------------------------CTACCACCGCGAACCCTCGAC-------GCGCGACC-AGGCGAGCTCGAAGCATCG-AT----ACTGACCTCGGTTCTTTAGGCAAACCATCTCTGGCGAGCACGGTCTCGACAGCAATGGCGTGTATGCACCTCCTATTCCC--TGCCCG-TGG-----CCCTCGTC--CTCTTCTCC---GGCTTGGCACTGATGATCGCACAGTTACAACGGCACTTCCGAGCTCCAGCTCGAGCGCATGAACGTCTACTTCAACGAGGTCAGT----CAATA-GCCACGTCGCC------------------AA-TTCAAA-TTTGA-A----------CCTCT-CG-GC-ATGG-TCAACTGCCGCCGC----CAAG----CCCCTGCTAACGCG-TTTTCGCCC-AGGCCTCCGGCAACAAGTATGTGCCCCGCGCCGTCCTCGTCGATCTCGAGCCCGGTACCATGGACGCCGTC

>Diaporthe_eres_CBS_138594

NNNNNNN????????????????????????????????????????????????????????????????????????????????????????????????????????????????????????????????????????????????????????????????????????????????????????????????????????????????????????????????????????????????????????????????????????????????????????????GGTGCTGCTTTCTGGTGCGTT------------------------CCAG--CTCGAG---CTCCAAGT-------------------------------CCACCGCCGCG-ACGCTTGAC-------ACGCGACA-ATACGACCTCGAAGCATCG-TT----GCTGACCTCGACT-TTTAGGCAAACCATCTCTGGCGAGCACGGCCTCGACAGCAATGGCGTGTATGCACCTCCTATGCCC--TGTCCA-CTG-----ATCTTGAC--CTCTCTTCC---GGCTTGGCACTGACAATCGCACAGTTACAACGGCACTTCTGAGCTCCAGCTCGAGCGCATGAACGTCTACTTCAACGAGGCAAGT----CAATA-ACAGCAC----------------------AA-CATTCA-TCCGA-C----------CATCTCCA-AC-ACGG-TTTACTGCCGTCGC----CCGA----AGTTCGCTAACGCG-TTATCGCCC-AGGCCTCCGGCAACAAGTATGTTCCTCGCGCCGTCCTCGTCGATCTCGAGCCCGGTACCATGGACGCCGTC

>Diaporthe_ueckerae_FAU_656

????????????????????????????????????????????????????????????????????????????????????????????????????????????????????????????????????????????????????????????????????????????????????????????????????????????????????????????????????????????????????????????????????????????????????????????????????GTGCTGCTTTCTGGTGCGTC------------------------GCAGCTCTCCAG---CTCCAAGC-------------------------------CTACCGCCGCGAACCCTCGAC-------GCGCGACA-AGGCGAGCTCGAAGCATCG-AT----ACTGACCTCGGTTCTTTAGGCAAACCATCTCCGGCGAGCACGGTCTCGACAGCAATGGCGTGTATGCACCTCCTATTCCC--TGCCCG-TGG-----CCCTCATC--CTCTTCTCC---GGCTTGGCACTGATGATCGCACAGTTACAACGGCACTTCCGAGCTCCAGCTCGAGCGCATGAACGTCTACTTCAACGAGGTCAGT----CAATA-GCCACGTCGTC------------------AA-TTCAAA-TTTGA-A----------CCTCT-CG-GC-ATGG-TCAACTGCCGCCGC----CAAG----CCCCTGCTAACGCG-TTTTCGCCC-AGGCCTCCGGCAACAAGTATGTGCCCCGCGCCGTCCTCGTCGATCTCGAGCCCGGTACCATGGACGCCGTC

>Diaporthe_longicolla_FAU_599

????????????????????????????????????????????????????????????????????????????????????????????????????????????????????????????????????????????????????????????????????????????????????????????????????????????????????????????????????????????????????????????????????????????????????????????????????GTGCTGCTTTCTGGTGCGTC------------------------GCAGCTCTCCAG---CTCCAAGC-------------------------------CTACCACCGCG-ACCCTCGAC-------GCGCGACA-AGGCGAGCTCGAAGCATCG-AT----ACTGACCTCGGTCCTTTAGGCAAACCATCTCTGGCGAGCACGGTCTCGACAGCAATGGCGTGTATGCACCTCCTATCCCC--TGCCCG-TGG----CCCCTCGTC--CTCTCCTCC---GGCTTGGCACTGATGATCGCACAGTTACAACGGCACTTCCGAGCTCCAGCTCGAGCGCATGAACGTCTACTTCAACGAGGTAAGT----CAATA-GCCACGTCGTC------------------AA-TTCAAA-TTTGA-CCCTCTCGGCACCTCT-CG-GC-ATGG-TCAACTGCTGCCGC----CAAG----CCTTTGCTAACGCG-TTTTCGCCC-AGGCCTCCGGCAACAAGTATGTGCCCCGCGCCGTCCTCGTCGATCTCGAGCCCGGTACCATGGACGCCGTC

>Diaporthe_guangdongensis_ZHKUCC20_0014

???????????????????????????????????????????????????????????????????????????????????????????????????????????????????????????????????????????????????????????????????????????????????????????????????????????????????????????????????????????????????????????????????????????????????????GGTAACCAAATCGGTGCTGCTTTCTGGTGCGTC------------------------GCAGCTCTCCAG---CTCCGAGC-------------------------------CTACCACCGCG-ACACCCGAC-------GCGCGACA-AGGCGAGCTCGAAACAGCG-AT----ACTGACCTCGATTCTTTAGGCAAACCATCTCTGGCGAGCACGGTCTCGACAGCAATGGCGTGTATGGACCTCCTATTCCC--TGACTA-CCG-----ACCTCGTC--CTCTCCTCC---GGCTTGGCACTGATGATCGCACAGTTACAACGGCACTTCCGAGCTCCAGCTCGAGCGCATGAACGTCTACTTCAACGAGGCAAGT----CAATA-GCCACGTCGCC------------------AA-TTCAGA-CTTGA-C----------CGTCT-TG-GC-ATGG-TAAACTGCCGCCGC----CAAG----CCCTTGCTAACGCG-TATTCGCCC-AGGCCTCCGGCAACAAGTATGTGCCCCGCGCCGTCCTCGTCGATCTCGAGCCCGGTACCATGGACGCCGTC

>Diaporthe_sojae_CBS_139282

????????????????????????????????????????????????????????????????????????????????????????????????????????????????????????????????????????????????????????????????????????????????????????????????????????????????????????????????????????????????????????????????????????????????????????????????????GTGCTGCTTTCTGGTGCGTC------------------------GCAGCTCTCCAG---CTCCAAGC-------------------------------CTACCACCGCG-ACCCTCGAC-------GCGCGACA-AGGCGAGCTCGAAGCATCG-AT----ACTGACCTCGTTTCTTTAGGCAAACCATCTCTGGCGAGCACGGTCTCGACAGCAATGGCGTGTATGCACCTCCTATTCCC--TGCCCA-CTG-ACTGCCCTGGTC--CTCCCCGCC---GGCTTGGCACTGATGATCGCACAGTTACAACGGCACTTCCGAGCTCCAGCTCGAGCGCATGAACGTCTACTTCAACGAGGTATGT----CAACG-GCCACGTCGTC------------------AA-TTCAAA-TTTGA-C----------CCTCT-CG-GC-ATGGATCAACCGCCGCCGC----CAAG----CCCTTGCTAACGCG-TTTTCCCCC-AGGCTTCCGGCAACAAGTATGTGCCCCGCGCCGTCCTCGTCGATCTCGAGCCCGGTACCATGGACGCCGTC

>Diaporthe_racemosae_CBS_143770

??????????????????????????????????????????????????????????????????????????????????????????????????????????????????????????????????????????????????????????????????????????????????????????????????????????????????????????????????????????????????????????????????????????????????????????????????????????????????????TA------------------------CCAG--CTCCAG---CTCCGAGC-------------------------------CTACCACCGCG-ATGATCGAC-------GCGCGACA-AGGCGAGCTCGAAGCATCG-AT----ACTGACCTCGGTCCTTTAGGCAAACCATCTCTGGCGAGCACGGTCTCGACAGCAATGGCGTGTATGTACCTCCTATTCCC--TGACTA-CTG-----ACCTCGTC--CTCTCCTCC---GGCTTGGCACTGACGATCGCACAGTTACAACGGCACTTCCGAGCTCCAGCTCGAGCGCATGAACGTCTACTTCAACGAGGTAAGT----CAACA-GCCACGTCGTC------------------AA-TTCGAA-TTTGA-C----------CGTCT-CG-GC-ATGG-TTAAATGCCGCCGC----CAAG----CCCTTGCTAACGCG-TTCTCGCCC-AGGCATCCGGCAACAAGTATGTGCCCCGCGCCGTCCTCGTCGATCTCGAGCCCGGTACCATGGACGCCGTC

>Diaporthe_rosae_MFLUCC_17_2658

???????????????????????????????????????????????????????????????????????????????????????????????????????????????????????????????????????????????????????????????????????????????????????????????????????????????????????????????????????????????????????????????????????????????????????????????????????????????????????????????????????????????????????????????????????????????????????????????????????????ACCGCGAACCCTCGAC-------GCGCGACC-AGGCGAGCTCGAAGCATCG-AT----ACTGACCTCGGTTCTTTAGGCAAACCATCTCTGGCGAGCACGGTCTCGACAGCAATGGCGTGTATGCACCTCCTATTCCC--TGCCCG-TGG-----CCCTCGTC--CTCTTCTTC---GGCTTGGCACTGATGATCGCACAGTTACAACGGCACTTCCGAGCTCCAGCTCGAGCGCATGAACGTCTACTTCAACGAGGTCAGT----CAATA-GCCACGTCGTC------------------AA-TTCAAA-TTTGA-A----------CCTCT-CG-GC-ATGG-TCAACTGCCGCCGC----CAAG----CCCCTGCTAACGCG-TTTTCGCCC-AGGCCTCCGGCAACAAGTATGTGCCCCGCGCCGTCCTCGTCGATCTCGAGCCCGGTACCATGGACGCCGTC

>Diaporthe_ovalispora_ICMP20659

????????????????????????????????????????????????????????????????????????????????????????????????????????????????????????????????????????????????????????????????????????????????????????????????????????????????????????????????????????????????????????????????????????????????????????????????????????????????????????????????????????????????????????????????????????????????????????????????????????????????????????????????????????????????AGCTCGAAGCATCG-AT----GCTGACTTCGGTTCTTTAGGCAAACCATCTCTGGCGAGCACGGTCTCGACAGCAATGGCGTGTATGCACCTCCTATTCCC--TGCCCA-CTG-ACTGCCCTGGTC--CTCTCCTCC---AGCTTGGCACTGATGATCGCACAGTTACAACGGCACTTCGGAGCTCCAGCTCGAGCGCATGAACGTCTACTTCAACGAGGTATGT----CAACG-GCCACGTCGTC------------------AA-TTCAAA-TTTGA-C----------TCTGT-CG-GC-ATGG-TCAGCTGCCGCCGC----CAAG----CCCTTGCTAACGCG-TTTTCCCCC-AGGCTTCCGGCAACAAGTATGTGCCCCGCGCCGTCCTCGTCGATCTCGAGCCCGGTACCATGGACGCCGTC

>Diaporthe_unshiuensis_CGMCC3_17569

????????????????????????????????????????????????????????????????????????????????????????????????????????????????????????????????????????????????????????????????????????????????????????????????????????????????????????????????????????????????????????????????????????????????????????????????????????????????????????????????????????????????????????????????????????????????????????????????????????????????????????????????????????????????AGCTCGAAGCATCG-AT----ACTGACCTCCGTCCTTTAGGCAAACCATCTCTGGCGAGCACGGTCTCGACAGCAATGGCGTGTATGCACCTCCTTTTCCC--TGCCCG-TGG----CCCCTCGTC--CTCTCCTCC---GGCTTGGCACTGATGATCGCACAGTTACAACGGCACTTCCGAGCTCCAGCTCGAGCGCATGAACGTCTACTTCAACGAGGTAAGT----CAATA-GCCAGGTCGTC------------------AA-TTCAAA-TTTGA-CCCTCTCGGCACCTTT-CG-GC-ATGG-TCAACTGCTGCCGC----CAAG----CCTTTGCTAACGCG-TTTTCGCCC-AGGCCTCCGGCAACAAGTATGTGCCCCGCGCCGTCCTCGTCGATCTCGAGCCCGGTACCATGGACGCCGTC

>Diaporthe_heterostemmatis_SAUCC194_85

??????????????????????????????????????????????????????????????????????????????????????????????????????????????????????????????????????????????????????????????????????????????????????????????????????????????????????????????????????????????????????????????????????????????????????????????????????????????????????????????????????????????????????CTCTCA---GCTC-AGC-------------------------------CTACCACCGCG-ACCCTCGAC-------GCGCGACA-AGGCGAGCCCGAAGCATCG-AT----ACTGACCTCGTTTCTTTAGGCAAACCATCTCTGGCGAGCACGGTCTCGACAGCAATGGCGTGTATGCACCTCCTATTCCC--TGCCCA-CTG-ACTGCCCTGGTC--CTCTCCTCC---GGCTTGGCACTGATGATCGCACAGTTACAACGGCACTTCCGAGCTCCAGCTCGAGCGCATGAACGTCTACTTCAACGAGGTATGT----CAACG-GCCACGTCGTC------------------AA-TTCAAA-TTTGA-C----------CCTCT-CG-GC-GTGGATCAACCGCCGCCGC----CAAG----CCCTTGCTAACGCG-TTTTCCCCC-AGGCTTCCGGCAACAAGTATGTGCCCCGCGCCGTCCTCGTCGATCTCGAGCCCGGTACCATGGACGCCGTC

>Diaporthe_tectonendophytica_MFLUCC_13_0471

????????????????????????????????????????????????????????????????????????????????????????????????????????????????????????????????????????????????????????????????????????????????????????????????????????????????????????????????????????????????????????????????????????????????????????????????????????????????????????????????????????????????????CTCT-CAG---CTCC-AGC-------------------------------CTACCACCGCG-ACCCTCGAC-------GCGCGACA-AGGCGAGCTCGAAGCATCG-AT----ACTGACCTCGGTTCTTTAGGCAAACCATCTCTGGCGAGCACGGTCTCGACAGCAATGGCGTGTATGTACCTCCTATTCCC--TGCCCA-CTG-----CCCTCGTC--CTCTCCTCC--GGGCTTGGCACTGATGATCGCGCAGTTACAACGGCACTTCCGAGCTCCAGCTCGAGCGCATGAACGTCTACTTCAACGAGGTAAGT----CAATA-GCCACGTCGCC------------------AA-TTCAAT-TTTGA-C----------CGTCT-CG-GT-ATGA-TGAACTGCCGCCGC----CAAG----CCATTGCTAACGCG-TTTTCCCCC-AGGCCTCCGGCAACAAGTATGTGCCCCGCGCCGTCCTCGTCGATCTCGAGCCCGGTACCATGGATGCCGTC

>Diaporthe_infertilis_CBS_230_52

GTCGGC-CCATGCTGTGCTCTCGCA----TCCTCTTC----CCCTGAGCATCAGGC----TACCCCACCATCGCGACCACACCCACGG--TCGGGCCTC-AAAACACAACCAGCTCCCTTCGAAGAGCACCCAGATG-CCCTTGGAACACGCGTCAGATTGCTAACATGACCTTT-TCCTCGCCCACAGGTTCACCTTCAGACCGGCCAATGCGTAAGTTACCTCCTGTCAAC-ACC-GCCAGACCTTATCGCCA-CCCGTAGCTGACACGTTTCCCAGGGTAACCAAATCGGTGCTGCTTTCTGGTGCGTC------------------------GCAGCTCTCCAG---CTCCAAGC-------------------------------CTACTGCCGCG-ACCCTCGAC-------GCGCGACA-AGGCGAGCTCGAAGCATCG-AT----ACTGACCTCGGTTCTCTAGGCAAACCATCTCTGGCGAGCACGGTCTCGACAGCAATGGCGTGTATGTACCTCCTATTCCC--TGCCGA-CCG-----CCCTCGTC--CTCTCCTCC---GGCTTGGCACTGATGATCGCACAGTTACAACGGCACTTCCGAGCTCCAGCTCGAGCGCATGAACGTCTACTTCAACGAGGTAAGT----CAATA-GCCACGTTGCC------------------AA-TTCAGA-TTTGA-G----------CCTCT-CG-GC-ATGG-TTGACTGCCGCCGC----CAAG----CCCTAGCTAACGCG-TATTCGCCC-AGGCCTCCGGCAACAAGTATGTCCCCCGCGCCGTCCTCGTCGATCTCGAGCCCGGTACCATGGACGCCGTC

>Diaporthe_melonis_CBS_507_78

GTCGGC-CCATGCT--GCTCTCGCA-TCCTCCTCTGC----CCCTGAACCTCAGCC----TACCCCACCATCGCGACCACACCCACGG--TCGGGCCTC-AAAACACCACCAGCGCCCTGCAAAGAGCTCCAAGATG-CCCTTGGAATACGCGTCAGATTGCTAACATGACCTTT-TTCTCGCCCACAGGTTCACCTTCAGACCGGCCAATGCGTAAGTTGCTCCTTGTCAAC-ACC-GCCAGACCTTATCGCCA-CCCGTAGCTGACACGTTTCCCAGGGTAACCAAATCGGTGCTGCTTTCTGGTGCGTC------------------------GCAGCTCTCCAG---CTCCGAGC-------------------------------CTACCACCGCG-ACACTCGAC-------GCGCGACA-AGGCGAGCTCGAAACAGCC-AT----ACTGACCTCGATTCTTTAGGCAAACCATCTCTGGCGAGCACGGTCTCGACAGCAATGGCGTGTATGGACCTCCTATTCCC--TGACTA-CCG------------C--CTCTCCTCT---GGCTTGGCACTGATGATCGCACAGTTACAACGGCACTTCCGAGCTCCAGCTCGAGCGCATGAACGTCTACTTCAACGAGGTAAGT----CAATA-GCCACGTCGCC------------------AA-TTCAGA-CTTGA-C----------CGTCT-CG-GC-ATGG-TGAACTGCCGCCGC----CAAG----CCCTTGCTAACGCG-TTTTCCTTT-AGGCCTCCGGCAACAAGTATGTGCCCCGCGCCGTCCTCGTCGATCTCGAGCCCGGTACCATGGACGCCGTC

>Diaporthe_endophytica_CBS_133811

GTCGGC-CCATGCTGTGCTCTCGCA----TCCTCTGC----CCCTGAGCCTGAGGC----TACCCCACCATCGCGACCACACCCATGG--TCGGGCCTC-AAAATACCACCAGCGCCCTGCGAAGAGCACCCAGATG-CTCTTGGAACACGCGTCAGATTGCTAACATGACCTTT-TTCTCGCCCACAGGTTCACCTTCAGACCGGCCAATGCGTAAGTTGCCTCCTGTCAAC-ACC-GCCAGGGCTTATCGCCA-CCCGTAGCTGACACGTTTCCCAGGGTAACCAAATCGGTGCTGCTTTCTGGTGCGTC------------------------GCAGCTCTCCAG---CTCCAAAC-------------------------------CTACCACCGCG-GCCCTCGAC-------GCGCGACA-AGGCGAGCTCGAAGCATCG-AT----ACTGACCTCGTTTCTCTAGGCAAACCATCTCTGGCGAGCACGGTCTCGACAGCAATGGCGTGTATGCACCTCCTATTCCC--TGCCCA-CTG-ACTGCCCTGGTC--CTCTCCTCC---GGCTTGGCACTGATGATCGCACAGTTACAACGGCACTTCCGAGCTCCAGCTCGAGCGCATGAACGTCTACTTCAACGAGGTATGT----CAACG-GCCACGTCGTC------------------AA-TTCAAA-TTTGA-A----------GCTCT-CG-GC-ATGG-TCAACTGCCGCCGC----CAAG----CCCTTGCTAACGCG-TTTTCCCCC-AGGCTTCCGGCAACAAGTATGTGCCCCGCGCCGTCCTCGTCGATCTCGAGCCCGGTACCATGGACGCCGTC

>Diaporthe_kongii_BRIP_54031

GTCGGC-CCATGCTGTGCTCTCGCA----TCCTCTGC----CCCTGAGCCTGAGGC----TACCCCACCATCGCGACCACACCCATGG--TCGGGCCTC-AAAACACCACCGTCGCCCTGCGAAGAGCACCCAGATG-CTACTGGAACACGCGTCAGATTGCTAACATGACCTTT-TTCTCGCCCACAGGTTCACCTTCAGACCGGCCAATGCGTAAGTTGCCTCCTGTCAAC-ACC-GCCAGACCTTATCGCCA-CCCGTAGCTGACACGTTTCCCAGGGTAACCAAATCGGTGCTGCTTTCTGGTGCGTC------------------------GCAGCTCTCCAG---CTTCAAGC-------------------------------CTACCACCGCG-ACCCTCGAC-------GCGCGACA-AGGCGAGCTCGAAGCATCG-AT----ACTGACCTCGTTTCTTTAGGCAAACCATCTCTGGCGAGCACGGTCTCGACAGCAATGGCGTGTATGCACCTCCTATTCCC--TGCCCA-CTG-ACTGCCCTGGTC--CTCTCCTCC---GGCTTGGCACTGATGATCGCACAGTTACAACGGCACTTCCGAGCTCCAGCTCGAGCGCATGAACGTCTACTTCAACGAGGTATGT----CAACG-GCCACGTCGTC------------------AA-TTCAAA-TTTGA-A----------CCTCT-CG-GC-ATGG-TCAACTGCCGCCGC----CAAG----CCCTTGCTAACGCG-TTTTCCCCC-AGGCTTCCGGCAACAAGTATGTGCCCCGCGCCGTCCTCGTCGATCTCGAGCCCGGTACCATGGACGCCGTC

>Diaporthe_masirevicii_BRIP_57892a

GTCGGC-CCATGCTGTGCTCTCGCA----TCCTCTGC----CCCTGAGCCTGAGGC----TACCCCACCATCGCGACCACACCCATGG--TCGGGCCTC-AAAACACCACCGTCGCCCTGCGAAGAGCACCCAGATG-CTACTGGAACACGCGTCAGATTGCTAACATGACCTTT-TTCTCGCCCACAGGTTCACCTTCAGACCGGCCAATGCGTAAGTTGCCTCCTGTCAAC-ACC-GCCAGACCTTATCGCCA-CCCGTAGCTGACACGTTTCCCAGGGTAACCAAATCGGTGCTGCTTTCTGGTGCGTC------------------------GCAGCTCTCCAG---CTTCAAGC-------------------------------CTACCACCGCG-ACCCTCGAC-------GCGCGACA-AGGCGAGCTCGAAGCATCG-AT----ACTGACCTCGTTTCTTTAGGCAAACCATCTCTGGCGAGCACGGTCTCGACAGCAATGGCGTGTATGCACCTCCTATTCCC--TGCCCA-CTG-ACTGCCCTGGTC--CTCTCCTCC---GGCTTGGCACTGATGATCGCACAGTTACAACGGCACTTCCGAGCTCCAGCTCGAGCGCATGAACGTCTACTTCAACGAGGTATGT----CAACG-GCCACGTCGTC------------------AA-TTCAAA-TTTGA-A----------CCTCT-CG-GC-ATGG-TCAACTGCCGCCGC----CAAG----CCCTTGCTAACGCG-TTTTCCCCC-AGGCTTCCGGCAACAAGTATGTGCCCCGCGCCGTCCTCGTCGATCTCGAGCCCGGTACCATGGACGCCGTC

>Diaporthe_subellipicola_KUMCC_17_0153

GTCGGC-CCATGCTGTGCTCTCGCA----TCCTCTGC----CCCTGAGCCTGAGGC----TACCCCACCATCGCGACCACACCCATGG--TCGGGCCTC-AAAACACCACCAGCGCCCTGCGAAGAGCACCCAGATG-CTACTGGAACACGCGTCAGATTGCTAACATGACCTTT-TTCTCGCCCACAGGTTCACCTCCAGACCGGCCAATGCGTAAGTTGCCTCCTGTCAAC-ACC-GCCAGACCTTATCGCCA-CCCGTAGCTGACACGTTTCCCAGGGTAACCAAATCGGTGCTGCTTTCTGGTGCGTC------------------------GCAGCTCTCCAG---CTCCAAGC-------------------------------CTACCACCGCG-ACCCTCGAC-------GCGCGACA-AGGCGAGCCCGAAGCATCG-AT----ACTGACCTCGTTTCTTTAGGCAAACCATCTCTGGCGAGCACGGTCTCGACAGCAATGGCGTGTATGCACCTCCTATTCCC--TGCCCA-CTG-ACTGCCCTGGTC--CTCTCCTCC---GGCTTGGCACTGATGATCGCACAGTTACAACGGCACTTCCGAGCTCCAGCTCGAGCGCATGAACGTCTACTTCAACGAGGTATGT----CAACG-GCCACGTCGTC------------------AA-TTCAAA-TTTGA-C----------CCTCT-CG-GC-GTGG-TCAACCGCCGCCGC----CAAG----CCCTTGCTAACGCG-TTTTCCCCC-AGGCTTCCGGCAACAAGTATGTGCCCCGCGCCGTCCTCGTCGATCTCGAGCCCGGTACCATGGACGCCGTC

>Diaporthe_fructicola_MAFF_246408

GTCGGC-CCATGCTGTGCTCTCGCA----TCCTCTGC----CCCTGAGCCTGAGGC----TACCCCACCATCGCGACCACACCCATGG--TCGGGCCTC-AAAACACCACCAGCGCCCTGCGAAGAGCACCCAGACG-CTCTTGTAACACGCGTCAGATTGCTAACATGACCTTT-TTCTCGCCCACAGGTTCACCTTCAGACCGGCCAATGCGTAAGTTGCCTCCTGTCAAC-ACC-GCCAGACCTTATCGCCA-CCCGTAGCTGACACGTTTCCCAGGGTAACCAAATCGGTGCTGCTTTCTGGTGCGTC------------------------GCAGCTCTCCAG---CTCCAAGC-------------------------------CTACCACCGCG-ACCCTCAAC-------GCGCGACA-AGGCGAGCTCGAAGCATCG-AT----ACTGACCTCGTTTCTTTAGGCAAACCATCTCTGGCGAGCACGGTCTCGACAGCAATGGCGTGTATGCACCTCCTATTCCC--TGCCCA-CTG-ACTGCCCTGGTC--CTCTCCTCC---GGCTTGGCACTGATGATCGCACAGTTACAACGGCACTTCCGAGCTCCAGCTCGAGCGCATGAACGTCTACTTCAACGAGGTATGT----CAACG-GCCACGTCGTC------------------AA-TTCAAA-TTTGA-C----------CCTCT-CG-GC-GTGGATCAACCGCCGCCGC----CAAG----CCCTTGCTAACGCG-TTTTCCCCC-AGGCTTCCGGCAACAAGTATGTGCCCCGCGCCGTCCTCGTCGATCTCGAGCCCGGTACCATGGACGCCGTC

>Diaporthe_phaseolorum_CBS_113425

GTCGGC-CCATGCTGTGCTCTCGCA----TCCTCTGC----CCCTGAGCCTGAGGC----TACCCCACCATCGCGACCACACCCATGG--TCGGGCCTC-AAAACACCACCAGCGCCCTGTGAAGAGCACCCAGATG-CTCTTGGAACACGCGTCAGATTGCTAACATGACCTTT-TTCTCGCCCACAGGTTCACCTTCAGACCGGCCAATGCGTAAGTTGCCTCCTGTCAAC-ACC-GCCAGACCTTATCGCCA-CCCGTAGCTGACACGTTTCCCAGGGTAACCAAATCGGTGCTGCTTTCTGGTGCGTC------------------------GCAGCTCTCCAG---CTCCAAGC-------------------------------CTACCACCGCG-ACCCTCGAC-------GCGCGACA-AGGCGAGCTCGAAGCATCG-AT----ACTGACCTCGTTTCTTTAGGCAAACCATCTCTGGCGAGCACGGTCTCGACAGCAATGGCGTGTATGCACCTCCTATTCCC--TGCCCA-CTG-ACTGCCCTGGTC--CTCCCCGCC---GGCTTGGCACTGATGATCGCACAGTTACAACGGCACTTCCGAGCTCCAGCTCGAGCGCATGAACGTCTACTTCAACGAGGTATGT----CAACG-GCCACGTCGTC------------------AA-TTCAAA-TTTGA-C----------CCTCT-CG-GC-ATGGATCAACCGCCGCCGC----CAAG----CCCTTGCTAACGCG-TTTTCCCCC-AGGCTTCCGGCAACAAGTATGTGCCCCGTGCCGTCCTCGTCGATCTCGAGCCCGGTACCATGGACGCCGTC

>Diaporthe_schini_CBS_133181

GTCGGC-CCATGCT--GCTCTCGCA-CCTTCCTCTGC----CCCTGAGCCTCAGGC----TACCCCACCATCGCGACCACACCCACGG--TCGGGCCTC-AAAACACCACCAGCACCTTGCGAAGACCACCCAGATG-CCCTTGGAACACGCGTCAGATTGCTAACATGAACTTT-TTTTCGCCTACAGGTTCACCTTCAGACCGGCCAATGCGTAAGTTGCCTCCTGTCAAC-ACC-GCCCGACCTTATCGCCA-CCCGTAGCTGACACGTTTCCCAGGGTAACCAAATCGGTGCTGCTTTCTGGTGCGTA------------------------CCAG--CTCCAG---CTCCGAGC-------------------------------CTACCACCGCG-ATGATCGAC-------GCGCGACA-AGGCGAGCTCGAAGCATCG-AT----ACTGACCTCGGTTCTTTAGGCAAACCATCTCTGGCGAGCACGGTCTCGACAGCAATGGCGTGTATGTACCTCCTATTCCC--TGACTA-CTG-----ACCTCGTC--CTCTCCTCC---GGCTTGGCACTGACGATCGCACAGTTACAACGGCACTTCCGAGCTCCAGCTCGAGCGCATGAACGTCTACTTCAACGAGGTAAGT----CAATA-GCCACGTCGTC------------------AA-TTCGAA-TTTGA-C----------CCTCT-CG-GC-ATGG-TTGACTGCCGCCGC----CAAA----CCCTTGCTAACGCG-TTCTCGCCC-AGGCCTCCGGCAACAAGTATGTGCCCCGCGCCGTCCTCGTCGATCTCGAGCCCGGTACCATGGACGCCGTC

>Diaporthe_terebinthifolii_CBS_133180

GTCGGC-CCATGCT--GCTCTCGCA-TCCTCCTCTGC----CCCTGAACCTCAGGC----TACCCCACCATCGCGACCACACCCACGG--TCGGGCCTC-AAAACACCACCAGCACCTTGCGAAGACCACCCAGATG-CCCTTGGAATACGCGTCAGATTGCTAACATGACCTTT-TTCTCGCCCACAGGTTCACCTTCAGACCGGCCAATGCGTAAGTTGCCTCCTGTCAAC-ACC-GCCCGACCTTATCGCCA-CCCGTAGCTGACACGTTTCCCAGGGTAACCAAATCGGTGCTGCTTTCTGGTGCGTC------------------------CCAG--CTCCAG---CTCCGAGC-------------------------------CTACCACCGCG-ACCCTCGAC-------GCGCGACA-AGGCGAGCTCGAAGCATCG-AT----ACTGACCTTGGCTGTTTAGGCAAACCATCTCTGGCGAGCACGGTCTCGACAGCAATGGCGTGTACGTACCTCCTATTCCC--TGACTA-CTG-----ACCTCGTC--CTCTCCTCC---GGCTTGGCACTGACGATCGCACAGTTACAACGGCACTTCCGAGCTCCAGCTCGAGCGCATGAACGTCTACTTCAACGAGGTAAGT----CAATA-GCCACGTCGTC------------------AA-TTCAAA-TTTGA-C----------CGTCT-CG-GC-ATGA-TTGATAACCGCCGC----CAAA----CCCTTGCTAACGCG-CTCTCGCCC-AGGCCTCCGGCAACAAGTATGTGCCCCGCGCCGTCCTCGTCGATCTCGAGCCCGGTACCATGGACGCCGTC

>Diaporthe_tecomae_CBS_100547

GTCGGC-CCATGCTG-GCTCTCGCA-TCCTCCTCTGC----CCCTGAGCCTCAGGC----TACCCCACCATCGCGACCACACCCACGG--TCGGGCCTC-AAAACACCACCAGCACCCTGCAAAGAGCACTCAGATG-CCCTTGGAACACGCGTCAGATTGCTAACATGAACTTT-TTCTCGCCCACAGGTTCACCTTCAGACCGGCCAATGCGTAAGTTGCCTCCTGTCAAC-ACC-GCCCGACCTTATCGCCA-CCCGTAGCTGACACGTTTCCCAGGGTAACCAAATCGGTGCTGCTTTCTGGTGCGTC------------------------CCAG--CTCCAGCTCCTCCGAGC-------------------------------CTACCACCGCG-ACCCTCGAC-------GCGCGACA-AGGCGAGCTCGAAGCATCG-AT----ACTGACCTTGGCTGTTTAGGCAAACCATCTCTGGCGAGCACGGTCTCGACAGCAATGGCGTGTACGTACCTCCTATTCCC--TGACTA-CTC-----ACCTCGTC--CTCTCCTCC---GGCTTGGCACTGACCATCGCACAGTTACAACGGCACTTCCGAGCTCCAGCTCGAGCGCATGAACGTCTACTTCAACGAGGTAAGT----CAACA-GCCACGTCGTC------------------AA-TTCAGA-CTTGA-C----------CCCCT-CG-GC-ATGG-TTAATTGCCGCCGC----CAAG----CCCTTGCTAAGGCG-TTTTCGCCC-AGGCCTCCGGCAACAAGTATGTGCCCCGCGCCGTCCTCGTCGATCTCGAGCCCGGTACCATGGACGCCGTC

>Diaporthe_megalospora_CBS_143_27

GTCGGC-CCATGCT--GCTCTCGCA-TCCTCCTCTGC----CCCTGAGCCTCAGGC----TACCCCACCATCGCGATCACACCCACGG--TCGGGCCTC-AAAACACCACCAGCACCCTGCAAAGAACACCCAGATG-CCGTTGTAAGACGCGTCAGATTGCTAACATGACCTTT-TTCTTGCCCACAGGTTCACCTTCAGACCGGCCAATGCGTAAGTTGCTCCCTGTCAAC-ACC-CCCAGACCTCATCGCCA-CCTGTAGCTGACACGTTTCCCAGGGTAACCAAATCGGTGCTGCTTTCTGGTGCGTC------------------------CCAG--CTCCAG---CTTCGAGC-------------------------------CTACCACCGCG-ACGCTCGACGCTCGTTGCGCGACA-GGGCTAGCTCGTAGTATCG-AC----ACTGACCTCTGTTCTTTAGGCAAACCATCTCTGGCGAGCACGGTCTCGACAGCAATGGCGTGTATGTACCTCCTATTCCC--CGACTA-CCG-----ACCTCGTCCTCTCTCCTCC---GGCTTGGCACTGATGATCGCACAGTTACAACGGCACTTCCGAGCTCCAGCTCGAGCGCATGAACGTCTACTTCAACGAGGTATGT----CAATA-GCCACGTCGCC------------------AA-TTCAAA-TTTGA-C----------CGTCT-CG-GC-ATGG-TTAACTGCCGCCGC----CAAG----CCCTTGCTAATTCG-TTTTCGCCC-AGGCCTCCGGCAACAAGTATGTGCCCCGCGCCGTCCTCGTCGATCTCGAGCCCGGTACCATGGACGCCGTC

>Diaporthe_vochysiae_LGMF1583

GTCGGC-CCATGCTGTGCTCTCGCA----TCCTCTGC----CCCTGAGCCTGAGGC----TACCCCACCATCGCGACCACACCCACGG--TCGGGCCTC-AAAACACCACCAGCTCCCTGCGAAGAGCACCCAGATG-CCCTTGGAACACGCGTCAGATTGCTAACATGACCTTT-TTCTCGCCTGCAGGTTCACCTTCAGACCGGCCAATGCGTAAGTTGCTCCCCGTCAAC-ACC-GCCGGACCTTATCGCCA-CCCGTAGCTGACACGTTTACCAGGGTAACCAAATCGGTGCTGCTTTCTGGTGCGTC------------------------GCAGCTCTCCAG---CTCCAAGC-------------------------------CTACCACCGCGAACCCTCGAC-------GCGCGACA-AGGCGAGCTCGAAGCATCG-AT----ACTGACCTCGTTCCTTTAGGCAAACCATCTCTGGCGAGCACGGTCTCGACAGCAATGGCGTGTATGCACCTCCTATTCCC--TGCCCG-TGG-----CCCTCGTA--CTCTTCTCC---GGCTTGGCACTGATGATCGCACAGTTACAACGGCACTTCCGAGCTCCAGCTCGAGCGCATGAACGTCTACTTCAACGAGGTCAGT----CAATA-GCCACGTTGTC????????????????????-??????-?????-?----------?????-??-??-????-??????????????----????----??????????????-?????????-???????????????????????????????????????????????????????????????????????

>Diaporthe_hordei_CBS_481_92

GTCGGC-CCATGCTGTGCTCTCGCA-TCCTCCTCTGC----CCCTGAGACTCAGGCTACCTACCCCACCATCGCGACCACACCCACGG--TCGGGCCTC-AAAACACCACCAGCACCTTGCGAAGAGCACCCAG------------AGACGCGTCAGATTGCTAACATAGCCTTT-TCCTTGCCCACAGGTTCACCTCCAGACCGGCCAATGCGTAAGTTGCCTCCTGTCAAC-ACC-GTCGGACCTTACCGCCA-CCCGTAGCTGACACATTTCCCAGGGTAACCAAATCGGTGCTGCTTTCTGGTGCGTC------------------------CCAG--CTTCAG---CTCCGAGC-------------------------------CTACCACCGCG-ACGCTCGAC-------GCGCGACA-AGGCTAGCTCGTAGCCTCG-AT----ACTGACCTCGGCTCTTTAGGCAAACCATCTCTGGCGAGCACGGTCTCGACAGCAATGGCGTGTATGCACCTCCTATTCCC--TGACTT-CTG-----ACCTCGTC--CTCTCCTGC---GGCTTGGCACTGATGATCGCACAGTTACAACGGCACTTCCGAGCTCCAGCTCGAGCGCATGAATGTCTACTTCAACGAGGTAAGT----CAACA-GCCACGTCGCC------------------AATTTCAGA-TTTGA-C----------CGTCT-CG-GC-ATGG-TTGACTGCCACCGC----CAAG----GACTTGCTAACGCG-TTTTCGCCC-AGGCCTCCGGCAACAAGTATGTGCCCCGGGCCGTCCTCGTCGACCTCGAGCCCGGTACCATGGACGCCGTC

>Diaporthe_vexans_CBS_127_14

GTCGGC-CCATGCTGTGCTCTCGCA-TCCTCCTCTGC----CCCTGAGCCTCAGGC----TACCCCACCATCGCGGCCACACCAACGA--TCGGGCCTC-AAAACACCACCAGCACCTTGCGAAGAGCACCCAG------------AGACGCGTCAGATTGCTAACATGGCGTTT-TTCTTGCCCACAGGTTCACCTTCAGACCGGCCAATGCGTAAGTTGCCTACTGTCAAC-ACC-GTCGGACCTTCTCGCCA-CCCGTAGCTGACACGTTTCCCAGGGTAACCAAATCGGTGCTGCTTTCTGGTGCGTC------------------CCAGCTCCAG--CTCCAG---CTCTCTGC-------------------------------CTACCACCGCG-ACCCTCGAC-------GCGCGACA-AGGCTAGCTCGTAGTCTCGAAT----ACTGACCTCGAATCCTTAGGCAAACCATCTCTGGCGAGCACGGTCTCGACAGCAATGGCGTGTATGCACCTCCTATTCCC--TGACTC-CTGACCTCGTCTCGTC--CTCTCCTCC---GGCCTGGCACTGATGATCGAACAGTTACAACGGCACTTCCGAGCTCCAGCTCGAGCGCATGAACGTCTACTTCAACGAGGTAAGCTAAACAACA-GCCACGTCACC------------------AA-TTCAGA-TTTGA-C----------CGTCC-CG-GC-ATGG-TTGACTGCCGCCGC----CAAAGACTGACTTGCTAACGCG-TTCTCGCCC-AGGCCTCCGGCAACAAGTATGTGCCCCGCGCCGTCCTCGTCGATCTCGAGCCCGGTACCATGGACGCCGTC

>Diaporthe_helianthi_CBS_592_81

GTCGGC-CCATGCTGTGCTCTCGCA-TCCTCCTCTGC----CCCTGAGCCTCAACGC---TACCCCACCATCGCGACCACACCCACGG--TCGCGCCTC-AAAACACCACCATCACCCTACGAAGAGCACCCAGATG-CGCTTGAAGGACGCGTCAGATTGCTAACATGAGCTTT-TCCTCGCCTACAGGTTCACCTTCAGACCGGCCAATGCGTAAGTTGCTCCCTGTCAAC-ACG-GCCGGGCCGTTTCGCCA-CCTGTAGCTGACACGTTTCCCAGGGTAACCAAATCGGTGCTGCTTTCTGGTGCGTC------------------CCTGCTCCAG--CTTCTG---CTCCAAGC-------------------------------CTACCACCGCG-ACGCTCGAC-------ACGCGAAA-AGGCTGGCTCGTAGTCTCG-AT----GCTGACCCCGGCTCTTCAGGCAAACCATCTCTGGCGAGCACGGTCTCGACAGCAATGGCGTGTATGTACCTCCTATTCCC--TGACTT-CTA-----AACTCGTC--CTCTCCTCA---AGCTTGCCACTAATGATCGCACAGCTACAACGGCACTTCCGAGCTCCAGCTCGAGCGCATGAACGTCTACTTCAACGAGGTACGC----GAACG-GCCACCTCGTC------------------AA-TTCAGA-TTTGA-C----------CCTCT-CG-GC-ATAATTAAACGGCTGTCGC----CAAG----CCCTTGCTAACGTG-TTTTCGCCC-AGGCTTCCGGCAACAAGTATGTGCCTCGCGCCGTCCTCGTCGATCTCGAGCCCGGTACCATGGACGCCGTC

>Diaporthe_amygdali_CBS_126679

GTCGGC-CCATGGC-TGCTTTCGCA----TCCTCTGC----CCCTGAGCC----------TACCCCACCATCGCGACCACACCCACGA--TCGGGCCTC-AAAACACCACAAATACCCTGAAACAAGCACTTCCATG--CCTTCGAAGACGCGTCAGATTGCTAACATGGCCTTT-TTCTCGCCCACAGGTTCATCTCCAGACCGGCCAATGCGTAAGTTGCTCCTGTCAACACACC-ACCGCACCTTATCGCCG-CCTGTAGCTGACACGTTTCCCAGGGTAACCAAATCGGTGCTGCTTTCTGGTGCGTC------------------------CCAG--CT----------------------------------------------CCATCACCGCG-ATACTCGAC-------GCGCGACA-ACACGACCTCGCAACATCC-TT----ACTGACCTCGACTC-GTAGGCAAACCATCTCTGGCGAGCACGGCCTCGACACCAATGGCGTGTATGCACCTCCTATTCCA--TGCCCA-TCA-----ATCTCGGC--CT---CGG----GGATTGGCACTGACAATTGCACAGCTACAACGGCACTTCCGAGCTCCAGCTCGAGCGCATGAACGTCTACTTCAACGAGGTAAGT----CAATC-ACCATGTCATG------------------GC-GTTAAC----GA-G----------CCCCCAAC-AC-GATG-CCTTCTTTTGTCGC----TTGG----GCTTTGCTGACCGC-TTATCGCCCTAGGCCTCCGGCAACAAGTATGTTCCCCGCGCCGTCCTCGTCGATCTCGAGCCCGGTACCATGGACGCCGTC

>Diaporthe_durionigena_VTCC_930005

??????????????????????????????????????????????????????????????????????????????????????????????????????????????????????????????????????????????????????????????????????????????????????????????????????????????????????????????????????????????????????????????????????????????????????????????????CGGTGCTGCTTTCTGGTGCGTC------------------------GCAGCTCTCCAG---CTCCAAGC-------------------------------CTGCCACCGCGGACCCTCGAC-------GCGCGACC-AGGCGAGCTCGAAGCATCG-AT----ACTGACCTCGGTTCTTTAGGCAAACCATCTCTGGCGAGCACGGTCTCGACAGCAATGGCGTGTATGCACCTCCTATTCCC--TGCCCG-TGG-----CCCTCGTC--CTCTTCTCC---GGCTTGGCACTGATGATCGCACAGTTACAACGGCACTTCCGAGCTCCAGCTCGAGCGCATGAACGTCTACTTCAACGAGGTCAGT----CAATA-GCCACGTCGTC------------------AA-TTCAAA-TTTGA-A----------CCTCT-CG-GC-ATGG-TCAACTGCCGCCGC----CAAG----CCCCTGCTAACGCG-TTTTCGCCC-AGGCCTCCGGCAACAAGTATGTGCCCCGCGCCGTCCTCGTCGATCTCGAGCCCGGTACCATGGACGCCGTC

>Diaporthe_breyniae

?????????????????????????????????????????????????????????????????????????????????????????????????????????????????????????????????????????????????????????????????????????????????????????????????????????????????????????????????????????????????????????????????????????????????????????????????????TGCTGCTTTCTGGTGCGTC------------------------GCAGCTCTCCAG---CTCCAAGC-------------------------------CTACCACCGCG-ACCCTCGAC-------GCGCGACA-AGGCGAGCTCGAAGCATCG-AT----ACTGACCTCGGTTCTTTAGGCAAACCATCTCTGGCGAGCACGGTCTCGACAGCAATGGCGTGTATGCACCTCCTATTCCC--TGCCCG-TGG-----CCCTCGTC--CTCTCCTCC---GGCTTGGCACTGATGATCGCACAGTTACAACGGCACTTCCGAGCTCCAGCTCGAGCGCATGAACGTCTACTTCAACGAGGTCAGT----CAATA-GCCACGTTGTC------------------AA-TTCAAA-TTTGA-G----------CCTCT-CG-GC-ATGG-TCAACTGCCGCCGC----CAAG----CCCTTGCTAACGCG-TTTTCGCCC-AGGCCTCCGGCAACAAGTATGTGCCCCGCGCCGTCCTCGTCGATCTCGAGCCCGGTACCATGGACGCCGTC

Allan-Perkins E, Li D-W, Schultes N, Yavuz S, LaMondia J (2020) The identification of a new species, *Diaporthe humulicola*, a pathogen causing Diaporthe leaf spot on common hop. Plant Disease 104: 2377–2390. https://doi.org/10.1094/PDIS-08-19-1770-RE

Ando Y, Masuya H, Aikawa T, Ichihara Y, Tabata M (2017) *Diaporthe toxicodendri* sp. nov., a causal fungus of the canker disease on *Toxicodendron vernicifluum* in Japan. Mycosphere 8: 1157–1167. DOI: 10.5943/mycosphere/8/5/6

Ariyawansa HA, Tsai, I, Wang J-Y, Withee P, Tanjira M, Lin S-R, Suwannarach N, Kumla J, Elgorban AM, Cheewangkoon R (2021) Molecular phylogenetic diversity and biological characterization of *Diaporthe* species associated with leaf spots of *Camellia sinensis* in Taiwan. Plants 10: 1434. https://doi.org/10.3390/plants10071434

Beluzán F, Olmo D, León M, Abad-Campos P, Armengol J (2021) First report of *Diaporthe amygdali* associated with twig canker and shoot blight of nectarine in Spain. Plant Disease 105. https://doi.org/10.1094/PDIS-10-20-2283-PDN

Chang CQ, Cheng YH, Xiang MM, Jiang ZD (2005) New species of *Phomopsis* on woody plants in Fujian Province. Mycosystema 24: 6–11.

Crous PW, Carnegie AJ, Wingfield MJ, et al. (2019) Fungal Planet description sheets: 868–950. Persoonia 42: 291–473. https://doi.org/10.3767/persoonia.2019.42.11

Crous PW, Hernández-Restrepo M, Schumacher RK, et al. (2021) New and Interesting Fungi. 4. Fungal Systematics and Evolution 7: 255–343. https://doi.org/10.3114/fuse.2021.07.13

Crous PW, Luangsa-ard JJ, Wingfield MJ, et al. (2018a) Fungal Planet description sheets: 785–867. Persoonia 41: 238–417. https://doi.org/10.3767/persoonia.2018.41.12

Crous PW, Schumacher RK, Wingfield MJ, et al. (2015a) Fungal Systematics and Evolution, FUSE 1. Sydowia 67: 81–118. DOI: 10.12905/0380.sydowia67-2015-0081

Crous PW, Shivas RG, Quaedvlieg W, et al. (2014a) Fungal Planet description sheets: 214–280. Persoonia 32: 184–306. https://doi.org/10.3767/003158514X682395

Crous PW, Summerell BA, Shivas RG, et al. (2011) Fungal Planet description sheets: 92–106. Persoonia 27: 130–162. https://doi.org/10.3767/003158511X617561

Crous PW, Summerell BA, Shivas RG, et al. (2012) Fungal Planet description sheets: 107–127. Persoonia 28: 138–182. https://doi.org/10.3767/003158512X652633

Crous PW, Wingfield MJ, Burgess TI, et al. (2016a) Fungal Planet description sheets: 469–557. Persoonia 37: 218–403. https://doi.org/10.3767/003158516X694499

Crous PW, Wingfield MJ, Burgess TI, et al. (2017) Fungal Planet description sheets: 625–715. Persoonia 39: 270–467. https://doi.org/10.3767/persoonia.2017.39.11

Crous PW, Wingfield MJ, Burgess TI, et al. (2018b) Fungal Planet description sheets: 716–784. Persoonia 40: 240–393. https://doi.org/10.3767/persoonia.2018.40.10

Crous PW, Wingfield MJ, Guarro J, et al. (2013) Fungal Planet description sheets: 154–213. Persoonia 31: 188–296. https://doi.org/10.3767/003158513X675925

Crous PW, Wingfield MJ, Le Roux JJ, et al. (2015b) Fungal Planet description sheets: 371–399. Persoonia 35: 264–327. https://doi.org/10.3767/003158515X690269

Crous PW, Wingfield MJ, Richardson DM, et al. (2016b) Fungal Planet description sheets: 400–468. Persoonia 36: 316–458. https://doi.org/10.3767/003158516X692185

Crous PW, Wingfield MJ, Schumacher RK, et al. (2014b) Fungal Planet description sheets: 281–319. Persoonia 33: 212–89. https://doi.org/10.3767/003158514X685680

Crous PW, Wingfield MJ, Schumacher RK, et al. (2020) New and Interesting Fungi. 3. Fungal Systematics and Evolution 6: 157–231. https://doi.org/10.3114/fuse.2020.06.09

da Silva RMF, Soares AM, Pádua APSL, Firmino AL, Souza-Motta CM, da Silva GA, Plautz HL Jr, Bezerra JDP, Paiva LM, Ryvarden L, Oliani LC, de Mélo MAC, Magalhães OMC, Pereira OL, Oliveira RJV, Gibertoni TB, Oliveira TGS, Svedese VM, Fan XL (2019) Mycological Diversity Description II. Acta Botanica Brasilica 33: 163–173. https://doi.org/10.1590/0102-33062018abb0411

Dayarathne MC, Jones EBG, Maharachchikumbura SSN, Devadatha B, Sarma VV, Khongphinitbunjong K, Chomnunti P, Hyde KD (2020) Morpho-molecular characterization of microfungi associated with marine based habitats. Mycosphere. 11: 1–188. DOI: 10.5943/mycosphere/11/1/1

de Silva NI, Maharachchikumbura SSN, Thambugala KM, Bhat DJ, Karunarathna SC, Tennakoon DS, Phookamsak R, Jayawardena RS, Lumyong S, Hyde KD (2021) Morphomolecular taxonomic studies reveal a high number of endophytic fungi from *Magnolia candolli* and *M. garrettii* in China and Thailand. Mycosphere 11: 163–237. DOI: 10.5943/mycosphere/12/1/3

Dissanayake AJ, Camporesi E, Hyde KD, Zhang W, Yan JY, Li XH (2017a) Molecular phylogenetic analysis reveals seven new *Diaporthe* species from Italy. Mycosphere 8: 853–877. DOI: 10.5943/mycosphere/8/5/4

Dissanayake AJ, Chen Y-Y, Liu J-K (2020) Unravelling *Diaporthe* species associated with woody hosts from karst formations (Guizhou) in China. Journal of Fungi 6: 251. https://doi.org/10.3390/jof6040251

Dissanayake AJ, Zhang W, Liu M, Hyde KD, Zhao WS, Li XH, Yan JY (2017b) *Diaporthe* species associated with peach tree dieback in Hubei, China. Mycosphere 8: 533–549. DOI: 10.5943/MYCOSPHERE/8/5/3

Doilom M, Dissanayake AJ, Wanasinghe DN, Boonmee S, Liu J-K, Bhat DJ, Taylor JE, Bahkali AH, McKenzie EHC, Hyde KD (2017) Microfungi on *Tectona grandis* (teak) in Northern Thailand. Fungal Diversity 82: 107–182. https://doi.org/10.1007/s13225-016-0368-7

Dong Z, Manawasinghe IS, Huang Y, Shu Y, Phillips AJL, Dissanayake AJ, Hyde KD, Xiang M, Luo M (2021) Endophytic *Diaporthe* associated with *Citrus grandis* cv. Tomentosa in China. Frontiers in Microbiology 11: 3621. https://doi.org/10.3389/fmicb.2020.609387

Du Z, Fan XL, Hyde KD, Yang Q, Liang Y-M, Tian C-M (2016) Phylogeny and morphology reveal two new species of *Diaporthe* from *Betula* spp. China. Phytotaxa 269: 90–102. https://doi.org/10.11646/phytotaxa.269.2.2

Fan XL, Hyde KD, Udayanga D, Wu X-Y, Tian C-M (2015) *Diaporthe rostrata*, a novel ascomycete from *Juglans mandshurica* associated with walnut dieback. Mycological Progress 14: 82. https://doi.org/10.1007/s11557-015-1104-5

Fan X, Yang Q, Bezerra JDP, Alvarez LV, Tian C-M (2018) *Diaporthe* from walnut tree (*Juglans regia*) in China, with insight of the *Diaporthe eres* complex. Mycological Progress 17: 841–853. https://doi.org/10.1007/s11557-018-1395-4

Feng X-X, Chen J-J, Wang G-R, Cao T-T, Zheng Y-L, Zhang C-L (2019) *Diaporthe sinensis*, a new fungus from *Amaranthus* sp. in China. Phytotaxa 425: 259–268. https://doi.org/10.11646/phytotaxa.425.5.1

Gao H, Pan M, Tian C, Fan X (2021) *Cytospora* and *Diaporthe* species associated with hazelnut canker and dieback in Beijing, China. Frontiers in Cellular and Infection Microbiology 11: 664366. https://doi.org/10.3389/fcimb.2021.664366

Gao YH, Su YY, Sun W (2015) *Diaporthe* species occurring on *Lithocarpus glabra* in China, with descriptions of five new species. Fungal Biology 119: 295–309. https://doi.org/10.1016/j.funbio.2014.06.006

Gao Y, Liu F, Cai L (2016) Unravelling *Diaporthe* species associated with *Camellia*. Systematics and Biodiversity 14: 102–117. https://doi.org/10.1080/14772000.2015.1101027

Gao YH, Liu F, Duan W, Crous PW, Cai L (2017) *Diaporthe* is paraphyletic. IMA Fungus 8: 153–187. https://doi.org/10.5598/imafungus.2017.08.01.11

Gao YH, Sun W, Su YY (2014) Three new species of *Phomopsis* in Gutianshan Nature Reserve in China. Mycological Progress 13: 111–121. https://doi.org/10.1007/s11557-013-0898-2

Gomes RR, Glienke C, Videira SIR, Lombard L, Groenewald JZ, Crous PW (2013) *Diaporthe*: a genus of endophytic, saprobic and plant pathogenic fungi. Persoonia 31: 1–41. https://doi.org/10.3767/003158513X666844

Guarnaccia V, Crous PW (2017) Emerging citrus diseases in Europe caused by *Diaporthe* spp. IMA Fungus 8: 317–334. https://doi.org/10.5598/imafungus.2017.08.02.07

Guarnaccia V, Crous PW (2018) Species of *Diaporthe* on *Camellia* and *Citrus* in the Azores Islands. Phytopathologia Mediterranea 57: 307−319. https://doi.org/10.14601/Phytopathol_Mediterr-23254

Guarnaccia V, Groenewald JZ, Woodhall J, Armengol J, Cinelli T, Eichmeier A, Ezra D, Fontaine F, Gramaje D, Gutierrez-Aguirregabiria A, Kaliterna J, Kiss L, Larignon P, Luque J, Mugnai L, Naor V, Raposo R, Sándor E, Váczy KZ, Crous PW (2018) *Diaporthe* diversity and pathogenicity revealed from a broad survey of grapevine diseases in Europe. Persoonia 40: 135–153. https://doi.org/10.3767/persoonia.2018.40.06

Guo YS, Crous PW, Bai Q, Fu M, Yang MM, Wang XH, Du YM, Hong N, Xu WX, Wang GP (2020) High diversity of *Diaporthe* species associated with pear shoot canker in China. Persoonia 45: 132–162. https://doi.org/10.3767/persoonia.2020.45.05

Hilário S, Amaral IA, Gonçalves MFM, Lopes A, Santos L, Alves A (2020) *Diaporthe* species associated with twig blight and dieback of *Vaccinium corymbosum* in Portugal, with description of four new species. Mycologia 112: 293–308. DOI: 10.1080/00275514.2019.1698926.

Huang F, Hou X, Dewdney MM, Fu Y, Chen G, Hyde KD, Li H (2013) *Diaporthe* species occurring on citrus in China. Fungal Diversity 61: 237–250. https://doi.org/10.1007/s13225-013-0245-6

Huang F, Udayanga D, Wang X, Hou X, Mei X, Fu Y, Hyde KD, Li H (2015) Endophytic *Diaporthe* associated with *Citrus*, a phylogenetic reassessment with seven new species from China. Fungal Biology 119: 331–347. https://doi.org/10.1016/j.funbio.2015.02.006

Huang S, Xia J, Zhang X, Sun W (2021) Morphological and phylogenetic analyses reveal three new species of *Diaporthe* from Yunnan, China. MycoKeys 78: 49–77. https://doi.org/10.3897/mycokeys.78.60878

Hu DM, Cai L, Hyde KD (2012) Three new ascomycetes from freshwater in China. Mycologia 104: 1478–1489. https://doi.org/10.3852/11-430

Hyde KD, Chaiwan N, Norphanphoun C, et al. (2018) Mycosphere notes 169–224. Mycosphere 9: 271–430. DOI: 10.5943/mycosphere/9/2/8

Hyde KD, Dong Y, Phookamsak R, et al. (2020) Fungal diversity notes 1151–1276: taxonomic and phylogenetic contributions on genera and species of fungal taxa. Fungal Diversity 100: 5–277. https://doi.org/10.1007/s13225-020-00439-5

Hyde KD, Hongsanan S, Jeewon R, et al. (2016) Fungal diversity notes 367–492, taxonomic and phylogenetic contributions to fungal taxa. Fungal Diversity 80: 1–270. https://doi.org/10.1007/s13225-016-0373-x

Hyde KD, Tennakoon DS, Jeewon R, et al. (2019) Fungal diversity notes 1036–1150: taxonomic and phylogenetic contributions on genera and species of fungal taxa. Fungal Diversity 96: 1–242. https://doi.org/10.1007/s13225-019-00429-2

Iantas J, Savi DC, Schibelbein RdS, Noriler SA, Assad BM, Dilarri G, Ferreira H, Rohr J, Thorson JS, Shaaban KA, Glienke C (2021) Endophytes of brazilian medicinal plants with activity against phytopathogens. Frontiers in Microbiology 12: 714750. https://doi.org/10.3389/fmicb.2021.714750

Jiang N, Voglmayr H, Piao C-G, Li Y (2021) Two new species of *Diaporthe* (Diaporthaceae, Diaporthales) associated with tree cankers in ﻿the Netherlands. MycoKeys 85: 31-56. https://doi.org/10.3897/mycokeys.85.73107

Lawrence DP, Travadon R, Baumgartner K (2015) Diversity of *Diaporthe* species associated with wood cankers of fruit and nut crops in northern California. Mycologia 107: 926–940. DOI: 10.3852/14-353.

Lesuthu P, Mostert L, Spies CFJ, Moyo P, Regnier T, Halleen F (2019) *Diaporthe nebulae* sp. nov. and first report of *D. cynaroidis, D. novem,* and *D. serafiniae* on grapevines in South Africa. Plant Disease 103: 808–817. https://doi.org/10.1094/PDIS-03-18-0433-RE

Li WJ, McKenzie EHC, Liu JK, D. Bhat J, Dai D-Q, Camporesi E, Tian Q, Maharachchikumbura SSN, Luo Z-L, Shang Q-J, Zhang J-F, Tangthirasunun N, Karunarathna SC, Xu J-C, Hyde KD (2020) Taxonomy and phylogeny of hyaline-spored coelomycetes. Fungal Diversity 100: 279–801. https://doi.org/10.1007/s13225-020-00440-y

Lin S, Taylor NJ, Peduto Hand F (2018) Identification and characterization of fungal pathogens causing fruit rot of deciduous holly. Plant Disease 102: 2430–2445. https://doi.org/10.1094/PDIS-02-18-0372-RE

Liu JK, Hyde KD, Jones EBG, et al. (2015) Fungal diversity notes 1–110: taxonomic and phylogenetic contributions to fungal species. Fungal Diversity 72: 1–197. https://doi.org/10.1007/s13225-015-0324-y

Lombard L, van Leeuwen GCM, Guarnaccia V, Polizzi G, Rijswick PCJ, Rosendahl KCHM, Gabler J, Crous PW (2014) *Diaporthe* species associated with *Vaccinium*, with specific reference to Europe. Phytopathologia Mediterranea 53: 287–299. DOI: 10.14601/PHYTOPATHOL_MEDITERR-14034

Long H, Zhang Q, Hao Y-Y, Shao X-Q, Wei X-X, Hyde KD, Wang Y, Zhao D-G (2019) *Diaporthe* species in south-western China. MycoKeys 57: 113–127. https://doi.org/10.3897/mycokeys.57.35448ç

Machingambi NM, Dreyer LL, Oberlander KC, Roux J, Roets F (2015) Death of endemic *Virgilia oroboides* trees in South Africa caused by *Diaporthe virgiliae* sp nov. Plant Pathology 64: 1149–1156. https://doi.org/10.1111/ppa.12341

Manawasinghe IS, Dissanayake AJ, Li X, Liu M, Wanasinghe DN, Xu J, Zhao W, Zhang W, Zhou Y, Hyde KD, Brooks S, Yan J (2019) High genetic diversity and species complexity of *Diaporthe* associated with grapevine dieback in China. Frontiers in Microbiology 10: 1936. https://doi.org/10.3389/fmicb.2019.01936

Manawasinghe IS, Jayawardena RS, Li HL, Zhou YY, Zhang W, Phillips AJL, Wanasinghe DN, Dissanayake AJ, Li XH, Li YH, Hyde KD, Yan JY (2021) Microfungi associated with *Camellia sinensis*: A case study of leaf and shoot necrosis on Tea in Fujian, China. Mycosphere 12: 430–518. DOI: 10.5943/mycosphere/12/1/6

Mapook A, Hyde KD, McKenzie EHC, Jones EBG, Bhat DJ, Jeewon R, Stadler M, Samarakoon MC, Malaithong M, Tanunchai B, Buscot F, Wubet T, Purahong W (2020) Taxonomic and phylogenetic contributions to fungi associated with the invasive weed *Chromolaena odorata* (Siam weed). Fungal Diversity 101: 1–175. https://doi.org/10.1007/s13225-020-00444-8

Marin-Felix Y, Hernández-Restrepo M, Wingfield MJ, et al. (2019) Genera of phytopathogenic fungi: GOPHY 2. Studies in Mycology 92: 47–133. https://doi.org/10.1016/j.simyco.2018.04.002

Milagres CA, Belisário R, Silva MA, Lisboa DO, Pinho DB, Furtado GQ (2018) A novel species of *Diaporthe* causing leaf spot in *Pachira glabra*. Tropical Plant Pathology 43: 460–467. https://doi.org/10.1007/s40858-018-0242-0

Mostert L, Crous PW, Kang J-C, Phillips AJL (2001) Species of *Phomopsis* and a *Libertella* sp. occurring on grapevines with specific reference to South Africa: morphological, cultural, molecular and pathological characterization. Mycologia 93: 146–167. https://doi.org/10.1080/00275514.2001.12061286

Noriler SA, Savi DC, Ponomareva L, Rodrigues R, Rohr J, Thorson JS, Glienke C, Shaaban KA (2019) Vochysiamides A and B: Two new bioactive carboxamides produced by the new species *Diaporthe vochysiae*. Fitoterapia 138: 104–273. https://doi.org/10.1016/j.fitote.2019.104273

Ozawa K, Mochizuki K, Takagi D, Ishida K, Sunada A, Ohkusu K, Kamei K, Hashimoto A, Tanaka K (2019) Identification and antifungal sensitivity of two new species of *Diaporthe* isolated. Journal of Infection and Chemotherapy 25: 96–103. https://doi.org/10.1016/j.jiac.2018.10.008

Pereira C, Ferreira B, Aucique-Perez C, Barreto R (2021) *Diaporthe rosiphthora* sp. nov.: Yet another rose dieback fungus. Crop Protection 139: 105365. https://doi.org/10.1016/j.cropro.2020.105365

Perera RH, Hyde KD, Dissanayake AJ, Jones EB, Liu JK, Wei D, Liu ZY (2018) *Diaporthe collariana* sp. nov., with prominent collarettes associated with *Magnolia champaca* fruits in Thailand. Studies in Fungi 3: 141–151. DOI: 10.5943/sif/3/1/16

Perera RH, Hyde KD, Maharachchikumbura SSN, Jones EBG, McKenzie EHC, Stadler M, Lee HB, Samarakoon MC, Ekanayaka AH, Camporesi E, Liu JK, Liu ZY (2020) Fungi on wild seeds and fruits. Mycosphere 11: 2108–2480. DOI: 10.5943/mycosphere/11/1/14

Petrović K, Riccioni L, Dordević V, Tubic SB, Miladinović J, Ceran M, Rajković D (2018) *Diaporthe pseudolongicolla* - the new pathogen on soybean seed in Serbia. Ratarstvo i povrtarstvo 55: 103–109. DOI: 10.5937/RATPOV55-18582

Phukhamsakda C, McKenzie EHC, Phillips AJL, Jones EBG, Bhat DJ, Stadler M, Bhunjun CS, Wanasinghe DN, Thongbai B, Camporesi E, Ertz D, Jayawardena RS, Perera RH, Ekanayake AH, Tibpromma S, Doilom M, Xu J, Hyde KD (2020) Microfungi associated with *Clematis* (Ranunculaceae) with an integrated approach to delimiting species boundaries. Fungal Diversity 102: 1–203. https://doi.org/10.1007/s13225-020-00448-4

Santos JM, Correia VG, Phillips AJL (2010) Primers for mating-type diagnosis in *Diaporthe* and *Phomopsis*, their use in teleomorph induction in vitro and biological species definition. Fungal Biology 114: 255–270. https://doi.org/10.1016/j.funbio.2010.01.007

Santos L, Phillips AJL, Crous PW (2017) *Diaporthe* species on Rosaceae with descriptions of *D. pyracanthae* sp. nov. and *D. malorum* sp. nov. Mycosphere 8: 485–511. DOI: 10.5943/mycosphere/8/5/2

Sun W, Huang S, Xia J, Zhang X, Li Z (2021) Morphological and molecular identification of *Diaporthe* species in south-western China, with description of eight new species. MycoKeys 77: 65–95. https://doi.org/10.3897/ mycokeys.77.59852

Tan YP, Edwards J, Grice KRE, Shivas RG (2013) Molecular phylogenetic analysis reveals six new *Diaporthe* species from Australia. Fungal Diversity 61: 251–260. https://doi.org/10.1007/s13225-013-0242-9

Tanney JB, McMullin DR, Green BD, et al. (2016) Production of antifungal and antiinsectan metabolites by the *Picea* endophyte *Diaporthe maritima* sp. nov. Fungal Biology 120: 1448–1457. https://doi.org/10.1016/j.funbio.2016.05.007

Tennakoon DS, Kuo CH, Maharachchikumbura SSN, Thambugala KM, Gentekaki E, Phillips AJL, Bhat DJ, Wanasinghe DN, de Silva NI, Promputtha I, Hyde KD (2021) Taxonomic and phylogenetic contributions to *Celtis formosana, Ficus ampelas, F. septica, Macaranga tanarius* and *Morus australis* leaf litter inhabiting microfungi. Fungal Diversity 108: 1–215. https://doi.org/10.1007/s13225-021-00474-w

Thompson SM, Tan YP, Young AJ, Neate SM, Aitken EAB, Shivas RG (2011) Stem cankers on sunflower (*Helianthus annuus*) in Australia reveal a complex of pathogenic *Diaporthe* (*Phomopsis*) species. Persoonia 27: 80–89. https://doi.org/10.3767/003158511X617110

Thompson SM, Tan YP, Shivas RG, Neate SM, Morin L, Bissett A, Aitken EAB (2015) Green and brown bridges between weeds and crops reveal novel *Diaporthe* species in Australia. Persoonia 35: 39–49. https://doi.org/10.3767/003158515X687506

Tibpromma S, Hyde KD, Bhat JD, Mortimer PE, Xu JC, Promputtha I, Doilom M, Yang JB, Tang AMC, Karunarathna SC (2018) Identification of endophytic fungi from leaves of Pandanaceae based on their morphotypes and DNA sequence data from southern Thailand. MycoKeys 33: 25–67. https://doi.org/10.3897/mycokeys.33.23670

Udayanga D, Castlebury LA, Rossman AY, Chukeatirote E, Hyde KD (2014a) Insights into the genus *Diaporthe*: phylogenetic species delimitation in the *D. eres* species complex. Fungal Diversity 67: 203–229. https://doi.org/10.1007/s13225-014-0297-2

Udayanga D, Castlebury LA, Rossman AY, Hyde KD (2014b) Species limits in *Diaporthe*: molecular re-assessment of *D. citri, D. cytosporella, D. foeniculina* and *D. rudis*. Persoonia 32: 83–101. https://doi.org/10.3767/003158514X679984

Udayanga D, Castlebury LA, Rossman AY, Chukeatirote E, Hyde KD (2015) The *Diaporthe sojae* species complex, phylogenetic re-assessment of pathogens associated with soybean, cucurbits and other field crops. Fungal Biology 119: 383–407. https://doi.org/10.1016/j.funbio.2014.10.009

Udayanga D, Liu XZ, Mckenzie EHC, Chukeatirote E, Hyde KD (2012) Multi-locus phylogeny reveals three new species of *Diaporthe* from Thailand. Cryptogamie Mycologie 33: 295–309. https://doi.org/10.7872/crym.v33.iss3.2012.295

Van Rensburg JCJ, Lamprecht SC, Groenewald JZ, Castlebury LA, Crous PW (2006) Characterization of *Phomopsis* spp. associated with die-back of rooibos (*Aspalathus linearis*) in South Africa. Studies in Mycology 55: 65–74. https://doi.org/10.3114/sim.55.1.65

Wanasinghe DN, Phukhamsakda C, Hyde KD, et al. (2018) Fungal diversity notes 709–839: taxonomic and phylogenetic contributions to fungal taxa with an emphasis on fungi on Rosaceae. Fungal Diversity 89: 1–236. https://doi.org/10.1007/s13225-018-0395-7

Wang X, Guo Y, Du Y, Yang Z, Huang X, Hong N, Xu W, Wang G (2021) Characterization of *Diaporthe* species associated with peach constriction canker, with two novel species from China. MycoKeys 80: 77–90. https:// doi.org/10.3897/mycokeys.80.63816

Wrona, CJ, Mohankumar, V, Schoeman, MH, Tan YP, Shivas RG, Jeff-Ego OS, Akinsanmi A (2020) Phomopsis husk rot of macadamia in Australia and South Africa caused by novel *Diaporthe* species. Plant Pathology 69: 911–921. https://doi.org/10.1111/ppa.13170

Yang Q, Fan XL, Du Z, Tian CM (2017a) *Diaporthe juglandicola* sp. nov. (Diaporthales, Ascomycetes) evidenced by morphological characters and phylogenetic analysis. Mycosphere 8: 817–826. DOI: 10.5943/mycosphere/8/5/3

Yang Q, Fan XL, Du Z, Liang Y-M, Tian C-M (2017b) *Diaporthe camptothecicola* sp. nov. on *Camptotheca acuminata* in China. Mycotaxon 132: 591–601. https://doi.org/10.5248/132.591

Yang Q, Fan XL, Du Z, Tian C-M (2017c) *Diaporthe* species occurring on *Senna bicapsularis* in southern China, with descriptions of two new species. Phytotaxa 302: 145–155. https://doi.org/10.11646/phytotaxa.302.2.4

Yang Q, Fan X-L, Guarnaccia V, Tian C-M (2018) High diversity of *Diaporthe* species associated with dieback diseases in China, with twelve new species described. MycoKeys 39: 97–149. https://doi.org/10.3897/mycokeys.39.26914

Yang Q, Jiang N, Tian C-M (2020) Three new *Diaporthe* species from Shaanxi Province, China. MycoKeys 67: 1–18. https://doi.org/10.3897/mycokeys.67.49483

Yang Q, Jiang N, Tian C-M (2021a) New species and records of *Diaporthe* from Jiangxi Province, China. MycoKeys 77: 41–64. https://doi.org/10.3897/mycokeys.77.59999

Yang Q, Tang J, Zhou GY (2021b) Characterization of *Diaporthe* species on *Camellia oleifera* in Hunan Province, with descriptions of two new species. MycoKeys 84: 15–33. https://doi.org/10.3897/mycokeys.84.71701

Zapata M, Palma MA, Aninat MJ, Piontelli E (2020) Polyphasic studies of new species of *Diaporthe* from native forest in Chile, with descriptions of *Diaporthe araucanorum* sp. nov., *Diaporthe foikelawen* sp. nov. and *Diaporthe patagonica* sp. nov. International Journal of Systematic and Evolutionary Microbiology 70: 3379–3390. DOI: 10.1099/ijsem.0.004183

Zhou H, Hou C-L (2019) Three new species of *Diaporthe* from China based on morphological characters and DNA sequence data analyses. Phytotaxa 422: 23. https://doi.org/10.11646/phytotaxa.422.2.3
